# Synthesis of Substituted Tetrafluorocyclobutenes and Their Ring-Opening Metathesis/Ring-Closing Metathesis to Dihydrofurans Bearing a Tetrafluoroethylene Unit

**DOI:** 10.1021/acsomega.6c02838

**Published:** 2026-05-13

**Authors:** Kateřina Kučnirová, Jaroslav Kvíčala, Josef Cvačka, Markéta Rybáčková

**Affiliations:** † Department of Organic Chemistry, 52735 University of Chemistry and Technology, Prague Technická 5, 166 28 Prague 6, Czech Republic; ‡ Institute of Organic Chemistry and Biochemistry of the Czech Academy of Sciences, Flemingovo nám. 542/2, 160 00 Prague 6, Czech Republic

## Abstract

We report the synthesis of a series of pentafluoro- and tetrafluorocyclobutenes containing alkylthio, phenylthio, alkoxy, phenoxy, and benzyloxy groups via reactions of perfluorocyclobutene with *S*- and *O*-nucleophiles. Although these substrates do not undergo ring-opening cross-metathesis with terminal alkenes, the synthesis of dihydrofurans bearing a tetrafluoroethylene unit in the side chain was achieved through a one-pot cascade reaction involving ring-opening and subsequent ring-closing metatheses of alkenyloxy tetrafluorocyclobutenes using the Hoveyda–Grubbs second-generation precatalyst.

## Graphical abstract

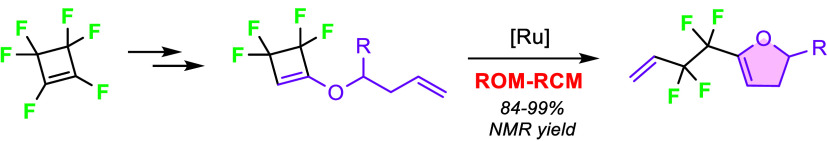

## Introduction

It is well-recognized that fluorinated compounds play an important role in medicinal chemistry and drug discovery.^1^ Dihydrofuran motifs are highly significant in naturally occurring and biologically relevant molecules.^2^ The synthetic strategies leading to dihydrofurans are constantly being developed.^3^ They can be elegantly synthesized by [3 + 2] cycloaddition between alkenes and 1,3-dicarbonyl compounds or similar multicomponent reactions.^4^ Domino reactions enable the easy construction of complex, highly functionalized molecules in a single reaction. Ring-rearrangement metathesis (RRM) is an example of such a domino transformation, where a ring-opening metathesis is followed by subsequent ring closure with an exocyclic double bond.^5^ Ring-opening metathesis, followed by ring-closing metathesis (ROM-RCM) of strained cyclic olefins, presents a simple method for the construction of multicyclic carbocycles or heterocycles, including dihydrofurans.^6^ Cyclic perfluorinated olefins have attracted considerable attention over the last decade. The two vinylic fluorine atoms show high reactivity toward carbon,^7^ sulfur,^8^ oxygen,^9^ and nitrogen^10^ nucleophiles. The vinylic fluorine atoms could also be substituted with hydrogen from sodium borohydride or lithium aluminum hydride.^7e,11^ The construction of fluorinated cycles still remains highly desirable. Recent advancements have focused on photochemical methodologies, e.g., the visible-light-induced formation of difluorocyclobutenones^12^ or the efficient access to monofluorocyclohexenes via the photocatalytic cascade cyclization of *gem*-difluoroalkene precursors.^13^ Fluorinated four-membered rings undergo several strain-release-driven transformations, including cascade reactions.^14^ There are also a few examples of ROMP of fluorinated cycloolefins.^15^ Perfluorocyclobutene (**1**) is a commercially available chemical, which can serve as a building block for the synthesis of polyfluorinated macrocyclic ethers^9c^ or Diels–Alder adducts of perfluorinated thioketones.^16^ Perfluorocyclobutene (**1**) also forms a stable adduct with 1,3-bis­(2,4,6-trimethylphenyl)­imidazolidin-2-ylidene^17^ and recently was used for the synthesis of photoswitchable fluorophores.^7g^ In our previous work, we have used perfluorocyclobutene (**1**) for the synthesis of nonsymmetrical tetrafluoroalkadienes via ROCM as the key step (Scheme 1a).^11d^

**Scheme 1:**
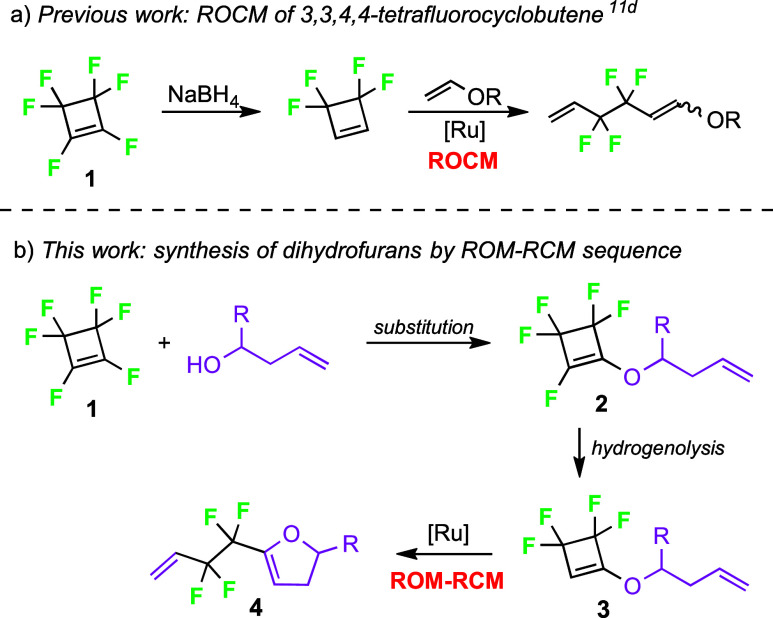
Metathesis of Fluorinated Cyclobutenes

Herein, we report the first example of ROM-RCM of substituted tetrafluorocyclobutenes **3**, obtained from perfluorocyclobutene (**1**) in a two-step procedure comprising the substitution of one vinylic fluoride by homoallyl alcohols and the hydrogenolysis of the second vinylic fluoride with lithium aluminum hydride (Scheme 1b).

## Results and Discussion

Perfluorocyclobutene (**1**) reacts readily with *C*-, *S*-, and *O*-nucleophiles. The reaction proceeds via an addition–elimination mechanism, and products of vinylic as well as allylic substitution could be formed^8c^ (Scheme 2).

**Scheme 2:**
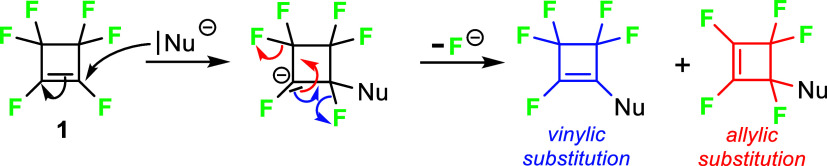
Addition–Elimination Mechanism of the Reaction of Perfluorocyclobutene (**1**) with Nucleophiles

By the reaction of perfluorocylobutene (**1**) with thiophenol and phenylmethanethiol in the presence of triethylamine, sulfides **5** were prepared (Scheme 3). The reaction proceeded with quite low chemoselectivity and mixtures of monosubstituted cyclobutenes **5a,b** with 1,2-disubstituted derivatives **6a,b** were formed even when 1 equiv of thiol and a low reaction temperature (−78 °C) were used. The byproducts from allylic substitution were also formed in small amounts. Some of the compounds have been previously described, but their full characterization was missing in most cases.^18–21^

**Scheme 3:**
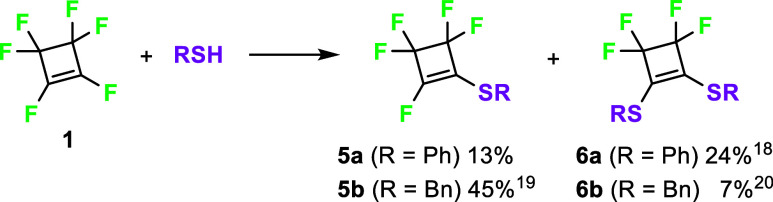
Reaction of Perfluorocyclobutene (**1**) with Thiols^a^

a Reagents and conditions: Et_3_N, Et_2_O, −78 °C, and 3–4 h.

In contrast to thiols, alcohols and phenols were considerably less reactive in the reaction with perfluorocyclobutene (**1**). The substitution took place at room temperature, and monosubstituted ethers **7** were formed almost exclusively, with only traces of dialkoxyderivatives **8** (Scheme 4).

**Scheme 4:**
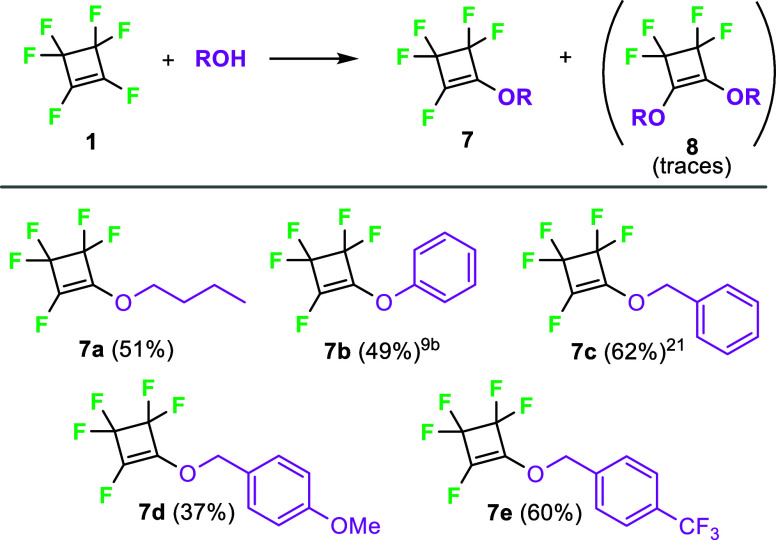
Reaction of Perfluorocyclobutene (**1**) with Alcohols and Phenols^a^

a Reagents and conditions: Et_3_N, Et_2_O, 0 °C, then rt, and 24–48 h.

The vinylic fluoride underwent hydrogenolysis by the reaction with lithium aluminum hydride, and the prepared sulfanes **5** and ethers **7** were transformed to tetrafluorocyclobutenes **9** and **10** in good yields (Scheme 5). The methoxy derivative **10d** proved to be highly unstable and rapidly decomposed.

**Scheme 5:**
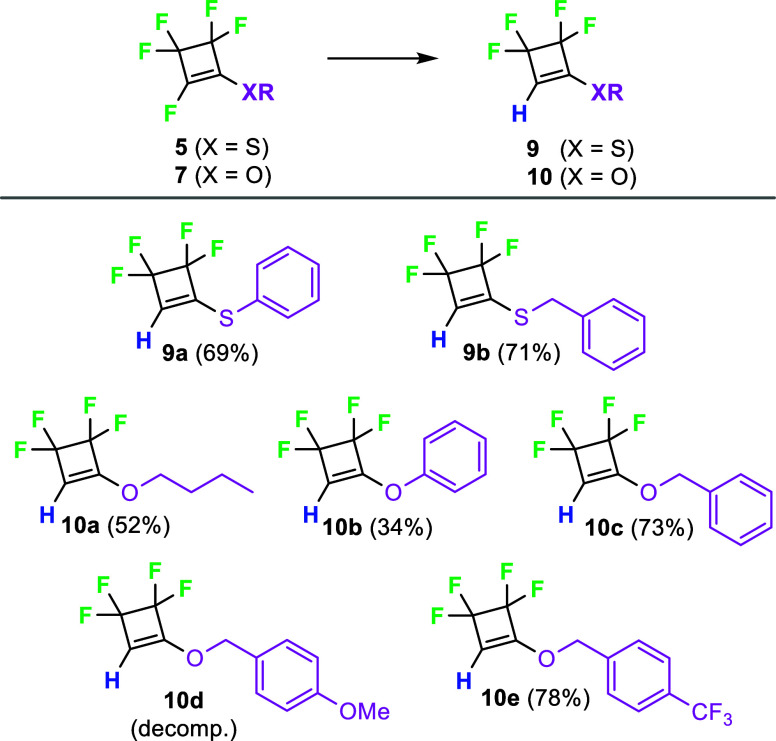
Hydrogenolysis of Pentafluorocyclobutenes **5** and **7** with Lithium Aluminum Hydride^a^

a Reagents and conditions: LiAlH_4_, Et_2_O, −78 °C, 1 h (for **9a,b**) or −78 °C, then rt, and 1–3 h (for **10a**–**e**).

The synthesized pentafluorocyclobutenes **5** and **7** as well as tetrafluorocyclobutenes **9** and **10** were subjected to the reaction with butyl vinyl ether in the presence of ruthenium precatalysts (Figure 1) in dichloromethane or toluene. However, in contrast to 3,3,4,4-tetrafluorocyclobutene (Scheme 1a),^11d^ no ring-opening cross-metathesis was observed, and all cyclobutenes were unreactive in the temperature range between 60 and 110 °C (heating of dichloromethane to this temperature required using a heavy-walled Schlenk flask fitted with a Teflon valve).

**Figure 1:**
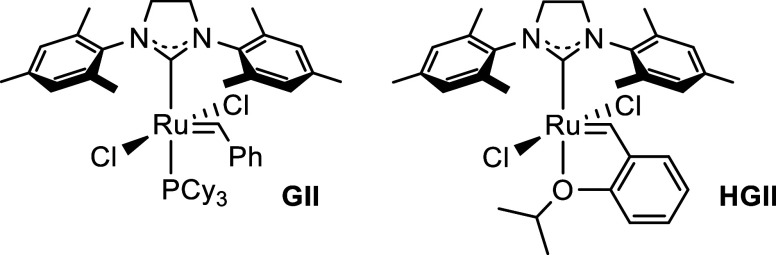
Structures of ruthenium precatalysts used in this study.

With ROM-RCM in mind, we synthesized substrates **12**, which have an alkenyloxy substituent in the side chain of the cyclobutene ring (Scheme 6). On treatment with metathesis precatalysts, these substrates should produce dihydropyrans **13**. However, the reaction of tetrafluorocyclobutene **12a** gave only very low conversion of the starting material, and no products could have been isolated. An approximately 70% conversion was observed in the case of tetrafluorocyclobutene **12b**. Nevertheless, the formation of the desired product **13b** was accompanied by the formation of another metathesis product: dihydrofuran **14b**. According to the ^19^F NMR of the crude reaction mixture, a 40:60 ratio of **13b** and **14b** was obtained. Dihydrofuran **14b** was formed after the isomerization of the double bond in the starting alkenyl alcohol. This undesired reaction pathway, in which the isomerization of the double bond occurs, is not unprecedented and has been previously described.^22^ It was not possible to separate dihydropyran **13b** from dihydrofuran **14b** and starting cyclobutene **12b** by chromatography, so the products were tentatively assigned by ^19^F NMR spectroscopy (see the SI).

**Scheme 6:**
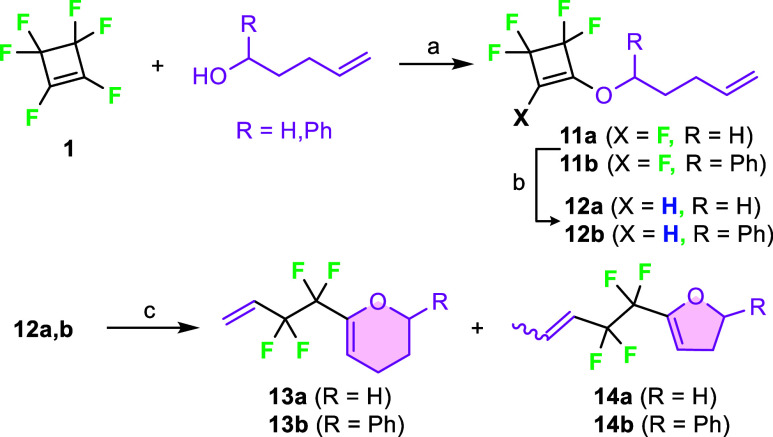
Synthesis of Tetrafluorocyclobutenes **12** and Their ROM-RCM toward Dihydropyrans **13** ^a^

a Reagents and conditions: (a) Et_3_N, Et_2_O, rt, and 48 h (for **11a**) or KOH, Et_2_O, 35 °C, and 24 h (for **11b**); (b) LiAlH_4_, Et_2_O, 0 °C, then rt, and 1 h; and (c) HGII (20 mol %), DCM, 80 °C, and 48 h.

To circumvent the problem of double-bond isomerization during metathesis, we have designed tetrafluorocyclobutene derivatives with a shorter alkenyloxy side chain and either a hydrogen atom (**3**) or an alkyl/aryl substituent (**15**) connected to the cyclobutene double bond. We have employed the same synthetic strategy, which consisted of replacing one vinylic fluoride with an alcohol, followed by hydrogenolysis of the remaining vinylic fluoride using LiAlH_4_ or nucleophilic substitution with an alkyl/aryl lithium reagent (Scheme 7).

**Scheme 7:**
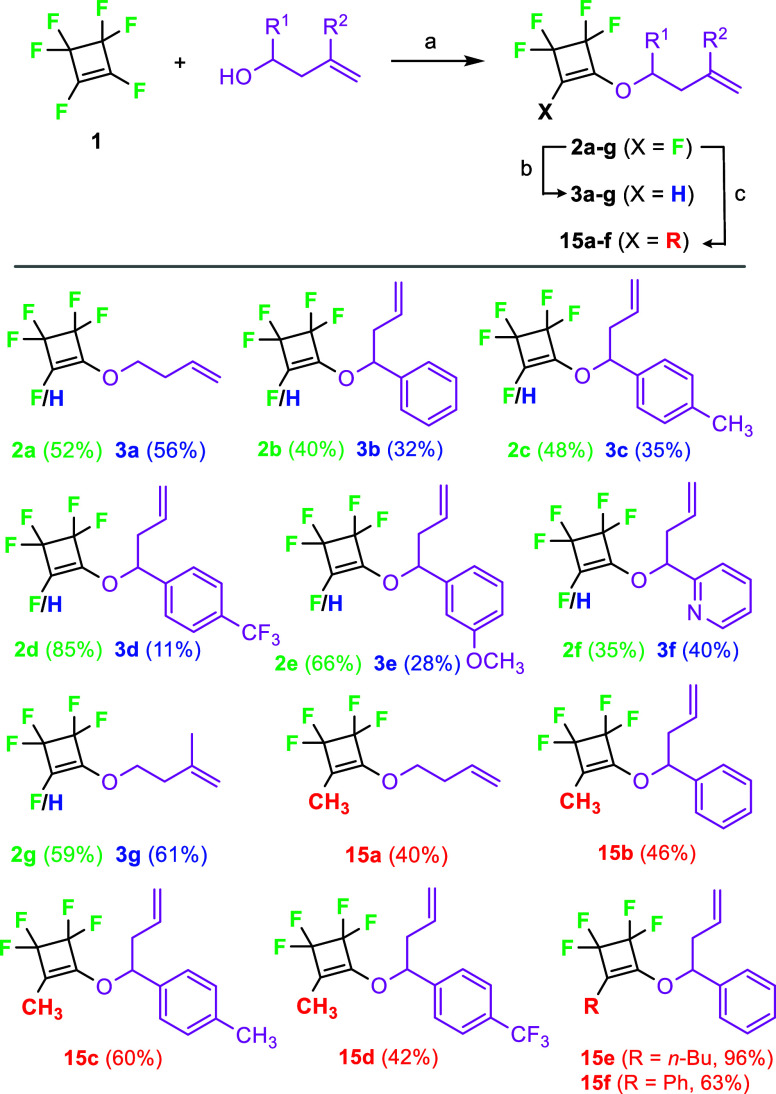
Synthesis of Pentafluorocyclobutenes **2** and Their Hydrogenolysis or Nucleophilic Substitution to Tetrafluorocyclobutenes **3** or **15** ^a^

a Reagents and conditions: (a) Et_3_N, Et_2_O, 0 °C, then rt, and 48 h (for **2a,g**) or KOH, Et_2_O, 35 °C, and 24 h (for **2b**–**f**); (b) LiAlH_4_, Et_2_O, 0 °C, then rt, and overnight; and (c) RLi, Et_2_O, 0 °C, then rt, and overnight (for **15a**–**d**) or −78 °C, then rt, and overnight (for **15e,f**).

Of the designed dienes, substrates **3g** and **15a**–**f** are particularly challenging. Metathesis of compound **3g** would form a tetrasubstituted double bond, while a tetrasubstituted double bond would break during the metathesis of dienes **15a**–**f**. Pilot experiments at ROM-RCM of tetrafluorocyclobutene **3a** using GII or HGII precatalyst were unsuccessful. The reactions proceeded with very low conversions (ca. 10%), and the formation of mixtures of products and high volatility made their identification impossible. In the case of phenyl derivative **3b**, the target dihydrofuran was formed in a 32% NMR yield using GII precatalyst at 60 °C (Table 1, entry 1). Increasing the temperature to 80 °C did not enhance the NMR yield (entry 2), and further increasing the temperature to 100 °C caused a slight decline (entry 3). Using HGII precatalyst and higher catalyst loading (20 mol %), we could enhance the NMR yield to 84% (entry 6).

**Table 1:**
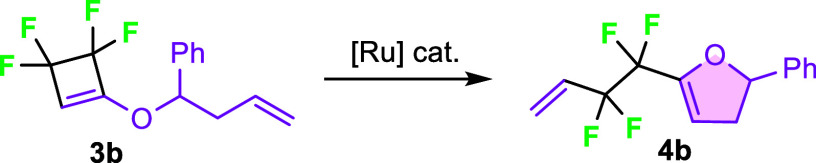
Optimization of ROM-RCM of Tetrafluorocyclobutene **3b** ^a^ ^,^ ^b^ ^,^ ^c^

entry	catalyst (mol %)	temp. (°C)	yield (%)
1	GII (10)	60	32^b^
2	GII (10)	80	30^b^
3	GII (10)	100	24^b^
4	HGII (10)	60	46^b^
5	HGII (10)	100	68^b^
6	HGII (20)	80	84^b^, 64^c^

a Reagents and conditions: GII or HGII (10–20 mol %), DCM, and 60–100 °C.

b NMR yield.

c Isolated yield.

The optimized reaction conditions (80 °C, 20 mol % of the HGII precatalyst) were used for the ROM-RCM reaction of the tetrafluorocyclobutenes **3c**–**g** and **15a**–**f** (Scheme 8). Out of substrates **3c**–**f** bearing only R^1^ substituent in the side chain (R^2^, R^3^=H), the best NMR yield was achieved with (trifluoromethyl)­phenyl derivative **3d**, while pyridine-containing substrate **3f** was completely unreactive. Cyclobutene **3g** containing a methyl group on the terminal double bond (R^1^, R^3^=H, R^2^=CH_3_) did not react under the metathesis conditions. Incorporation of a methyl group onto the cyclobutene ring caused a considerable decrease in the NMR yield of the target dihydrofurans. Very low conversion was observed for cyclobutene **15a**. Dihydrofurans **16b**–**d** were formed only in 25–32% NMR yields (compare to the unsubstituted derivatives **4b**–**d**). Moreover, complex reaction mixtures were formed; as a result, isolation of these dihydrofurans proved to be impossible. Their structures were tentatively assigned based on ^19^F NMR analysis of the reaction mixtures in analogy to **4b–d** (see SI).

**Scheme 8:**
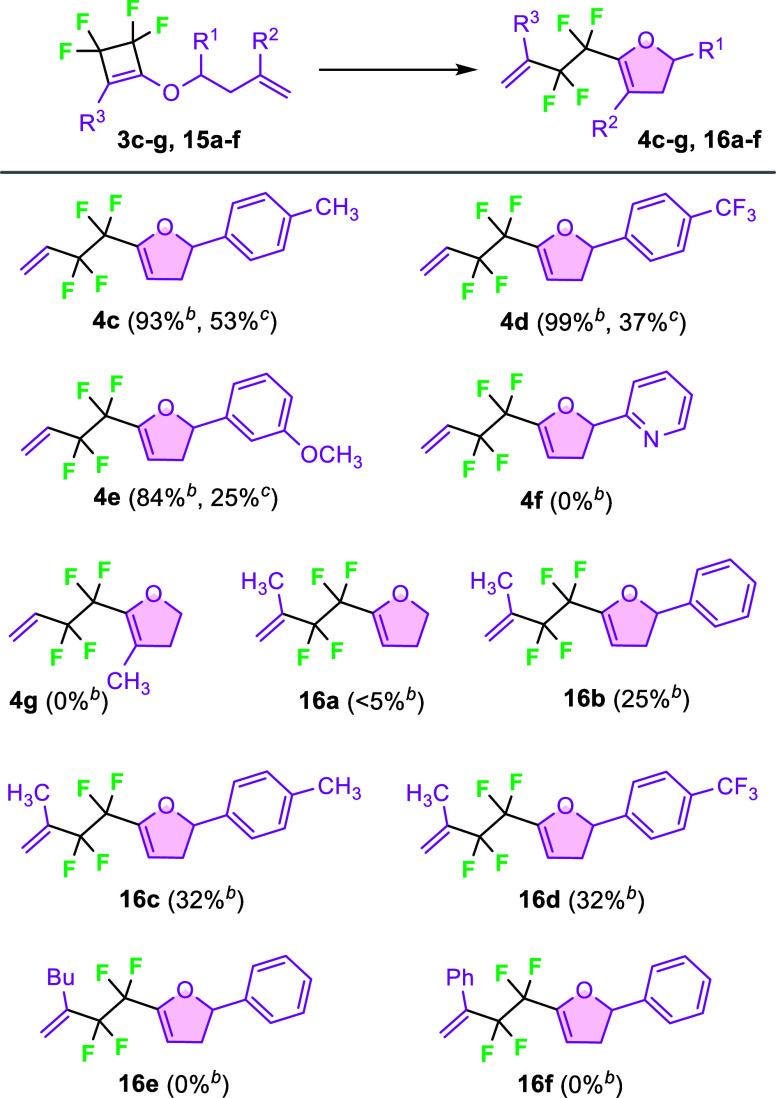
Scope of ROM-RCM of Tetrafluorocyclobutenes **3** and **15** ^a^ ^,^ ^b^ ^,^ ^c^

a Reagents and conditions: HGII (20 mol %), DCM, 80 °C, and 48 h.
b NMR yield.
c Isolated yield.

Replacing the methyl substituent with a butyl or phenyl group completely inhibited the metathesis reaction of cyclobutenes **15e,f**. While the current Ru-based protocol provides operational simplicity, highly sterically hindered tetrafluorocyclobutenes remain challenging. Future studies utilizing highly reactive Mo-based catalysts, which have recently shown remarkable success in cross-metathesis reactions of sterically demanding substrates,^23^ may provide a solution for the ROM-RCM of fluorinated cycloalkenes.

## Conclusions

In conclusion, we have developed the first synthesis of fluorinated dihydrofurans via a tandem ring-opening/ring-closing metathesis of tetrafluorocyclobutenes bearing an alkenyloxy side chain. Hexafluorocyclobutene was found to be a suitable building block for the synthesis of dihydrofurans that contain a tetrafluoroethylene unit in the side chain. Because compounds bearing the −CF_2_CF_2_– structural motif have found applications in both medicinal and materials chemistry,^24^ this strategy facilitates their synthetic availability. Future efforts will focus on expanding this methodology to access dihydropyran derivatives, which may require the evaluation of Ru hydride-quenching additives to mitigate potential alkene isomerization.^25^

## Supplementary Material



## Data Availability

The data underlying this study are available in the published article and its Supporting Information.

## Notes

Experimental details, characterization data, and NMR spectra (PDF)
