# Supplementary material for: Synthesis of Substituted Tetrafluorocyclobutenes and Their Ring-Opening Metathesis/Ring-Closing Metathesis to Dihydrofurans Bearing a Tetrafluoroethylene Unit
Source: ACS Omega. 2026 May 13;11(20):30163–8. doi: 10.1021/acsomega.6c02838 (PMC13216927; doi:10.1021/acsomega.6c02838)
Supplement: Supplementary file 1 [file ao6c02838_si_001.pdf]

Supporting Information

Synthesis of Substituted Tetrafluorocyclobutenes and Their Ring-Opening Metathesis/Ring-Closing Metathesis to Dihydrofurans Bearing Tetrafluoroethylene Unit

Kateřina Kučnirová[†], Jaroslav Kvíčala[†], Josef Cvačka[‡], Markéta Rybáčková[†]*

[†] Department of Organic Chemistry, University of Chemistry and Technology, Prague, Technická 5, 166 28 Prague 6, Czech Republic

[‡] Institute of Organic Chemistry and Biochemistry of the Czech Academy of Sciences, Flemingovo náměstí 542/2, 160 00 Prague 6, Czech Republic

Table of Contents

1. General information	S2
2. Reactions of perfluorocyclobutene (**1**) with *S*-nucleophiles	S3
3. Reactions of perfluorocyclobutene (**1**) with *O*-nucleophiles	S4
4. Synthesis of tetrafluorocyclobutenes **9**, **10**, **12**, **3** and **15**	S11
5. Procedure for the ROM-RCM sequence	S21
6. References	S25
7. Spectral data	S26

1. General information

NMR spectra were recorded with a Varian MercuryPlus spectrometer at frequencies 300 MHz for ^{1}H and 282 MHz for ^{19}F and with an Agilent 400-MR DDR2 spectrometer at frequencies 400 MHz for ^{1}H, 376 MHz for ^{19}F and 101 MHz for ^{13}C. Chemical shifts (δ) are reported in parts per million (ppm) with reference to residual signal of deuterated solvent for ^{1}H and ^{13}C NMR, and to CCl_3F as an internal standard for ^{19}F NMR. Signals are described as s = singlet, d = doublet, t = triplet, m = multiplet, bs – broad singlet. Coupling constants (*J*) are reported in Hz.

Mass spectra were recorded on LTQ Orbitrap XL (Thermo Fisher Scientific) for ESI and APCI, and on Agilent 7250A Series GC QTOF for EI and CI spectra.

Thin layer chromatography was performed using aluminum plates MERCK Kieselgel 60 F254, column chromatography was performed using silica gel MERCK Geduran Si 60, particle size 63-200 µm and basic silica gel Sigma Aldrich, particle size 230-400 µm, Ca 0.1-0.3 %).

Anhydrous solvents (diethyl ether, DCM) were purchased from VWR and dried using a solvent drying system PureSolv MD7 by Innovative Technologies.

Perfluorocyclobutene (**1**) was prepared following the procedure published in a previous study[S1]. Due to its low boiling point (0-2 °C) and high inhalation toxicity it was necessary to work with this compound with high caution and to store it in a pressure bottle.

Homoallylic alcohols (1-phenylbut-3-en-1-ol[S2], 1-(4-methylphenyl)but-3-en-1-ol[S3], 1-(4-(trifluoromethyl)phenyl)but-3-en-1-ol[S2], 1-(3-methoxyphenyl)but-3-en-1-ol[S4] and 1-(pyridin-2-yl)but-3-en-1-ol[S3]) were prepared by the reaction of the corresponding benzaldehydes with allyl magnesium bromide solution using previously published procedures and their analytical data were in accordance with the literature. 1-Phenylpent-4-en-1-ol was prepared following a previously published procedure[S5].

Two commercially available ruthenium precatalysts were used in the study (GII and HGII).

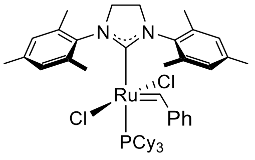

GII

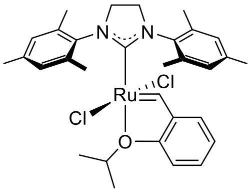

HGII

2. Reactions of perfluorocyclobutene (1) with *S*-nucleophiles

Reaction of perfluorocyclobutene (1) with thiophenol

Perfluorocyclobutene (**1**, 2.93 g, 18.1 mmol) was condensed in a 100 mL flask cooled to -78 °C and dissolved in Et_2O (40 mL). Triethylamine (2.5 mL, 18.1 mmol) was added at -78 °C and subsequently, thiophenol (1.98 g, 18.1 mmol) was added dropwise in 2 h. After stirring for 2 h at -78 °C the solvent was evaporated on a rotary vacuum evaporator and the residue was purified by column chromatography (*n*-hexane/DCM, 4:1). Pentafluorocyclobutene **5a** was obtained as a colourless liquid (0.60 g, 13%) and disubstituted tetrafluorocyclobutene **6a** was isolated as a colourless crystalline substance (1.5 g, 24%).

(2,3,3,4,4-Pentafluorocyclobut-1-en-1-yl)(phenyl)sulfane (**5a**)

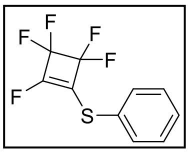

^{1}H NMR (400 MHz, $CDCl_3$): δ 7.39 – 7.51 (m, ^{Ar}C**H**, 3H), 7.59 – 7.64 (m, ^{Ar}C**H**, 2H) ppm. **^{19}F NMR** (282 MHz, $CDCl_3$): δ −115.3 (m, C**F**$_2$, 2F), −116.3 (m, C**F**$_2$, 2F), −117.3 (m, C**F**, 1F) ppm. **^{13}C NMR** (101 MHz, $CDCl_3$): δ 112.6 – 119.5 (m, **C**F$_2$, 2C), 124.8 (m, *ipso*-^{Ar}**C**, 1C), 127.8 (m, FC=**C**S, 1C), 129.8 (s, *meta*-^{Ar}**C**H, 2C), 130.5 (s, *ortho*-^{Ar}**C**H, 2C), 134.4 (s, *para*-^{Ar}**C**H, 1C), 148.9 (dtt, $^{1}J_{FC}$ = 340 Hz, $^{2}J_{FC}$ = 26 Hz, $^{3}J_{FC}$ = 23 Hz, F**C**=CS, 1C) ppm. **HRMS (EI):** Calculated for $C_{10}H_5F_5S$ $[M]^+$ 252.0032, found 252.0030.

(3,3,4,4-Tetrafluorocyclobut-1-ene-1,2-diyl)bis(phenylsulfane) (**6a**)

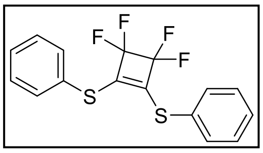

^{1}H NMR (300 MHz, $CDCl_3$)[S6]: δ 7.32 – 7.46 (m, ^{Ar}C**H**, 6H), 7.47 - 7.55 (m, ^{Ar}C**H**, 4H) ppm. **^{19}F NMR** (282 MHz, $CDCl_3$): δ −111.4 (s, C**F**$_2$, 4F) ppm. **HRMS (EI)**: Calculated for $C_{16}H_{10}F_4S_2$ $[M]^+$ 342.0160, found 342.0159.

Reaction of perfluorocyclobutene (1) with phenylmethanethiol

Perfluorocyclobutene (**1**, 2.7 g, 17 mmol) was condensed in a 100 mL flask cooled to -78 °C and dissolved in Et_2O (40 mL). Triethylamine (2.3 mL, 17 mmol) was added at -78 °C and subsequently, phenylmethanethiol (2.0 g, 17 mmol) was added dropwise in 1 h. After stirring for 2 h at -78 °C the solvent was evaporated on a rotary vacuum evaporator and the residue was purified by column chromatography (*n*-hexane/DCM, 5:1). Pentafluorocyclobutene **5b** was obtained as a colourless liquid (2.0 g, 45%) and disubstituted tetrafluorocyclobutene **6b** was isolated as a colourless crystalline substance (0.4 g, 7%).

Benzyl(2,3,3,4,4-pentafluorocyclobut-1-en-1-yl)sulfane (**5b**)

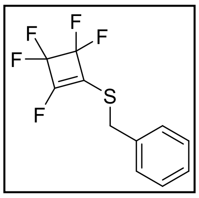

^{1}H NMR (400 MHz, $CDCl_3$): δ 4.28 (s, C**H**$_2$, 2H), 7.30 – 7.43 (m, ^{Ar}C**H**, 5H) ppm. **^{19}F NMR** (282 MHz, $CDCl_3$): δ −114.6 (m, C**F**$_2$, 2F), −116.0 (m, C**F**$_2$, 2F), −118.2 (m, C**F**, 1F) ppm. **^{13}C NMR** (101 MHz, $CDCl_3$): δ 35.4 (s, **C**H$_2$, 1C), 115.9 (tdt, $^{1}J_{FC}$ = 283 Hz, $^{2}J_{FC}$ = 12 Hz, $^{2}J_{FC}$ = 24 Hz, **C**F$_2$, 1C), 116.3 (tdt, $^{1}J_{FC}$ = 282 Hz, $^{2}J_{FC}$ = 10 Hz, $^{3}J_{FC}$ = 23 Hz, **C**F$_2$, 1C), 127.6

(m, S**C**=CF, 1C), 128.4 (s, *para*-^{Ar}**C**H, 1C), 128.8 (s, ^{Ar}**C**H, 2C), 129.0 (s, ^{Ar}**C**H, 2C), 134.6 (s, *ipso*-^{Ar}**C**, 1C), 149.7 (dtt, $^{1}J_{FC}$ = 338 Hz, $^{2}J_{FC}$ = 27 Hz, $^{3}J_{FC}$ = 24 Hz, SC=**C**F, 1C) ppm. **HRMS (EI):** Calculated for $C_{11}H_7F_5S$ $[M]^+$ 266.0189, found 266.0184.

(3,3,4,4-Tetrafluorocyclobut-1-ene-1,2-diyl)bis(benzylsulfane) (**6b**)

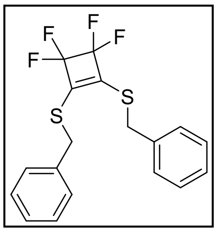

^{1}H NMR (400 MHz, $CDCl_3$): δ 4.23 (s, C**H**$_2$, 4H), 7.27 – 7.41 (m, ^{Ar}C**H**, 10H) ppm. **^{19}F NMR** (282 MHz, $CDCl_3$): δ −109.8 (s, C**F**$_2$, 4F) ppm. **^{13}C NMR** (101 MHz, $CDCl_3$): δ 35.5 (s, **C**H$_2$, 2C), 115.9 – 122.4 (m, **C**F$_2$, 2C), 128.1 (s, *para*-^{Ar}**C**H, 2C), 128.87 (s, ^{Ar}**C**H, 4C), 128.91 (s, ^{Ar}**C**H, 4C), 135.3 (s, *ipso*-^{Ar}**C**, 2C), 140.6 (m, **C**S, 2C) ppm. **HRMS (EI):** Calculated for $C_{18}H_{14}F_4S_2$ $[M]^+$ 370.0473, found 370.0470.

3. Reactions of perfluorocyclobutene (1) with *O*-nucleophiles

1-Butoxy-2,3,3,4,4-pentafluorocyclobutene (**7a**)

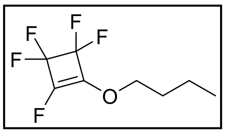

Perfluorocyclobutene (**1**, 3.18 g, 19.6 mmol) was condensed into a 100mL flask via a low-temperature addition funnel cooled to -50 °C. It was dissolved in Et_2O (30 mL) at 0 °C and subsequently butan-1-ol (1.45 g, 19.6 mmol) and triethylamine (2.7 mL, 19.6 mmol) were added. The reaction mixture was stirred at rt for 2 days, washed with water and the organic phase was dried over anhydrous $MgSO_4$. The solvent was evaporated on a rotary vacuum evaporator and the residue was dried under vacuum. Pentafluorocyclobutene **7a** was obtained as a colourless liquid (1.6 g, 51%).

^{1}H NMR (400 MHz, $CDCl_3$): δ 0.97 (t, $^{3}J_{HH}$ = 7.4 Hz, C**H**$_3$, 3H), 1.44 (m, C**H**$_2$, 2H), 1.75 (m, C**H**$_2$, 2H), 4.27 (td, $^{3}J_{HH}$ = 6.5 Hz, $^{4}J_{HH}$ = 1.5 Hz, C**H**$_2$O, 2H) ppm. **^{19}F NMR** (282 MHz, $CDCl_3$): δ −116.4 (m, C**F**$_2$, 2F), −119.5 (m, C**F**$_2$, 2F), −141.9 (m, C**F**, 1F) ppm. **^{13}C NMR** (101 MHz, $CDCl_3$): δ 13.5 (s, **C**H$_3$, 1C), 18.5 (s, **C**H$_2$, 1C), 30.9 (s, **C**H$_2$, 1C), 73.7 (s, O**C**H$_2$, 1C), 114.5 (tdt, $^{1}J_{FC}$ = 280 Hz, $^{2}J_{FC}$ = 43 Hz, $^{2}J_{FC}$ = 22 Hz, **C**F$_2$, 1C), 115.1 (tdt, $^{1}J_{FC}$ = 274 Hz, $^{2}J_{FC}$ = 19 Hz, $^{2}J_{FC}$ = 22 Hz, **C**F$_2$, 1C), 130.3 (dtt, $^{1}J_{FC}$ = 325 Hz, $^{2}J_{FC}$ = 27 Hz, $^{3}J_{FC}$ = 25 Hz, F**C**=CO, 1C), 136.7 (m, FC=**C**O, 1C) ppm. **HRMS (EI):** Calculated for $C_8H_9F_5O$ $[M]^+$ 216.0574, found 216.0565.

[(2,3,3,4,4-Pentafluorocyclobut-1-en-1-yl)oxy]benzene (**7b**)

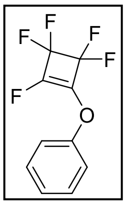

Perfluorocyclobutene (**1**, 0.96 g, 5.9 mmol) was condensed into a 50mL flask via a low-temperature addition funnel cooled to -50 °C. It was dissolved in Et_2O (10 mL) at 0 °C and subsequently phenol (0.56 g, 6.0 mmol) and triethylamine (0.84 mL, 6.0 mmol) were added. The reaction mixture was stirred at rt overnight, washed with water and the organic phase was dried over anhydrous $MgSO_4$. The solvent was evaporated on a rotary vacuum evaporator and the residue was purified by column chromatography (*n*-hexane/DCM, 3:1). Pentafluorocyclobutene **7b** was obtained as a colourless liquid (0.68 g, 49%).

^{1}H NMR (300 MHz, $CDCl_3$): δ 7.22 (m, *ortho*-^{Ar}C**H**, 2H), 7.31 (m, *para*-^{Ar}C**H**, 1H), 7.44 (m, *meta*-^{Ar}C**H**, 2H) ppm. **^{19}F NMR** (282 MHz, $CDCl_3$): δ −117.1 (m, C**F**$_2$, 2F), −119.1 (m, C**F**$_2$, 2F), −131.8 (m, C**F**, 1F) ppm. **^{13}C NMR** (101 MHz, $CDCl_3$): δ 114.6 (ttd, $^{1}J_{FC}$ = 275 Hz, $^{3}J_{FC}$ = 23 Hz, $^{3}J_{FC}$ = 19 Hz, **C**F$_2$, 1C), 115.0 (tdt, $^{1}J_{FC}$ = 280 Hz, $^{2}J_{FC}$ = 40 Hz, $^{3}J_{FC}$ = 20 Hz, **C**F$_2$, 1C), 118.4 (s, ^{Ar}**C**H, 1C), 126.7 (s, ^{Ar}**C**H, 2C), 130.2 (s, ^{Ar}**C**H, 2C), 131.7 (dtt, $^{1}J_{FC}$ = 330 Hz, $^{2}J_{FC}$ = 24 Hz, $^{3}J_{FC}$ = 24 Hz, F**C**=CO, 1C), 134.1 (m, FC=**C**O, 1C), 153.2 (s, *ipso*-^{Ar}**C**, 1C) ppm. **HRMS (EI):** Calculated for $C_8H_{10}F_5O$ $[M]^+$ 236.0261, found 236.0260.

{[(2,3,3,4,4-Pentafluorocyclobut-1-en-1-yl)oxy]methyl}benzene (**7c**)

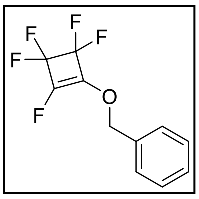

Perfluorocyclobutene (**1**, 0.96 g, 5.9 mmol) was condensed into a 50mL flask via a low-temperature addition funnel cooled to -50 °C. It was dissolved in Et_2O (10 mL) at 0 °C and subsequently benzyl alcohol (0.65 g, 6.0 mmol) and triethylamine (0.84 mL, 6.0 mmol) were added. The reaction mixture was stirred at rt overnight, washed with water and the organic phase was dried over anhydrous $MgSO_4$. The solvent was evaporated on a rotary vacuum evaporator and the residue was purified by column chromatography (*n*-hexane/DCM, 3:1). Pentafluorocyclobutene **7c** was obtained as a colourless liquid (0.92 g, 62%).

^{1}H NMR (300 MHz, $CDCl_3$): δ 5.27 (s, OC**H**$_2$, 2H), 7.33-7.50 (m, ^{Ar}C**H**, 5H) ppm. **^{19}F NMR** (282 MHz, $CDCl_3$): δ −116.5 (m, C**F**$_2$, 2F), −119.3 (m, C**F**$_2$, 2F), −139.9 (m, C**F**, 1F) ppm. **^{13}C NMR** (101 MHz, $CDCl_3$): δ 75.1 (s, O**C**H$_2$, 1C), 114.5 (tdt, $^{1}J_{FC}$ = 280 Hz, $^{2}J_{FC}$ = 43 Hz, $^{2}J_{FC}$ = 23 Hz, **C**F$_2$, 1C), 115.0 (tdt, $^{1}J_{FC}$ = 275 Hz, $^{2}J_{FC}$ = 19 Hz, $^{2}J_{FC}$ = 22 Hz, **C**F$_2$, 1C), 128.1 (s, ^{Ar}**C**H, 2C), 128.9 (s, ^{Ar}**C**H, 2C), 129.5 (s, *para*-^{Ar}**C**H, 1C), 133.5 (s, *ipso*-^{Ar}**C**, 1C), 136.3 (m, O**C**=CF, 1C), 130.9 (dtt, $^{1}J_{FC}$ = 327 Hz, $^{2}J_{FC}$ = 27 Hz, $^{3}J_{FC}$ = 24 Hz, OC=**C**F, 1C) ppm. **HRMS (EI):** Calculated for $C_{11}H_7F_5O$ $[M]^+$ 250.0417, found 250.0416.

1-Methoxy-4-{[(2,3,3,4,4-pentafluorocyclobut-1-en-1-yl)oxy]methyl}benzene (**7d**)

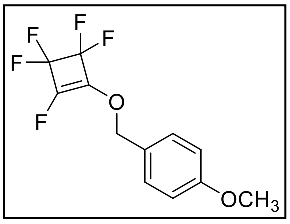

Perfluorocyclobutene (**1**, 3.00 g, 18.5 mmol) was condensed into a 50mL flask via a low-temperature addition funnel cooled to -50 °C. It was dissolved in Et_2O (10 mL) at 0 °C and subsequently (4-methoxyphenyl)methanol (2.56 g, 18.5 mmol) and triethylamine (2.6 mL, 18.5 mmol) were added. The reaction mixture was stirred at rt overnight, washed with water and the organic phase was dried over anhydrous $MgSO_4$. The solvent was evaporated on a rotary vacuum evaporator and the residue was purified by column chromatography (*n*-hexane/EtOAc, 8:1). Pentafluorocyclobutene **7d** was obtained as a colourless liquid (1.9 g, 37%).

^{1}H NMR (400 MHz, $CDCl_3$): δ 3.82 (s, C**H**$_3$O, 3H), 5.18 (s, C**H**$_2$, 2H), 6.93 (m, ^{Ar}C**H**, 2H), 7.31 (m, ^{Ar}C**H**, 2H) ppm. **^{19}F NMR** (376 MHz, $CDCl_3$): δ −116.4 (m, C**F**$_2$, 2F), −119.3 (m, C**F**$_2$, 2F), −140.0 (m, C**F**, 1F) ppm. **^{13}C NMR** (101 MHz, $CDCl_3$, ^{1}H and ^{19}F decoupling): δ 55.4 (s, **C**H$_3$O, 1C), 75.2 (s, **C**H$_2$, 1C), 114.4 (s, ^{Ar}**C**H, 2C), 114.6 (s, **C**F$_2$, 1C), 115.1 (s, **C**F$_2$, 1C), 125.6 (s, *ipso*-^{Ar}**C**, 1C), 130.3 (s, ^{Ar}**C**H, 2C), 130.8 (s, F**C**=CO, 1C),

136.4 (s, FC=**C**O, 1C), 160.7 (s, ^{Ar}**C**-OCH_3, 1C) ppm. **HRMS (CI):** Calculated for $C_{12}H_{10}F_5O_2$ $[M+H]^+$ 281.0595, found 281.0590.

1-{[(2,3,3,4,4-Pentafluorocyclobut-1-en-1-yl)oxy]methyl}-4-(trifluoromethyl)benzene (**7e**)

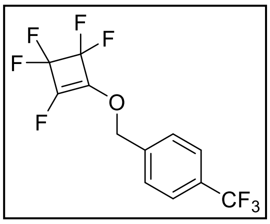

Perfluorocyclobutene (**1**, 1.80 g, 11.1 mmol) was condensed into a 50mL flask via a low-temperature addition funnel cooled to -50 °C. It was dissolved in Et_2O (10 mL) at 0 °C and subsequently (4-(trifluoromethyl)phenyl)methanol (1.96 g, 11.1 mmol) and triethylamine (1.55 mL, 11.1 mmol) were added. The reaction mixture was stirred at rt overnight, washed with water and the organic phase was dried over anhydrous $MgSO_4$. The solvent was evaporated on a rotary vacuum evaporator and the residue was purified by column chromatography (*n*-hexane). Pentafluorocyclobutene **7e** was obtained as a colourless liquid (2.12 g, 60%).

^{1}**H NMR** (400 MHz, $CDCl_3$): δ 5.31 (s, C**H**$_2$, 2H), 7.50 (m, ^{Ar}C**H**, 2H), 7.69 (m, ^{Ar}C**H**, 2H) ppm. ^{19}**F NMR** (376 MHz, $CDCl_3$): δ −63.3 (s, C**F**$_3$, 3F), −116.6 (m, C**F**$_2$, 2F), −119.2 (m, C**F**$_2$, 2F), −139.3 (m, C**F**, 1F) ppm. ^{13}**C NMR** (101 MHz, $CDCl_3$, ^{1}H and ^{19}F decoupling): δ 74.0 (s, **C**H$_2$, 1C), 114.5 (s, **C**F$_2$, 1C), 115.0 (s, **C**F$_2$, 1C), 123.8 (s, **C**F$_3$, 1C), 126.1 (s, ^{Ar}**C**H, 2C), 128.0 (s, ^{Ar}**C**H, 2C), 131.4 (s, F**C**=CO, 1C), 131.7 (s, ^{Ar}**C**-CF_3, 1C), 136.0 (s, FC=**C**O, 1C), 137.4 (s, *ipso*-^{Ar}C, 1C) ppm. **HRMS (CI):** Calculated for $C_{12}H_6F_7O$ $[M-F]^+$ 299.0301, found 299.0302.

1,3,3,4,4-Pentafluoro-2-[(pent-4-en-1-yl)oxy]cyclobutene (**11a**)

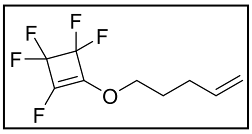

Perfluorocyclobutene (**1**, 1.65 g, 10 mmol) was condensed into a 100mL flask via a low-temperature addition funnel cooled to -50 °C. It was dissolved in Et_2O (20 mL) at 0 °C and subsequently pent-4-en-1-ol (0.90 g, 10 mmol) and triethylamine (1.4 mL, 10 mmol) were added. The reaction mixture was stirred at rt for 2 days, washed with water (3 × 10 mL) and the organic phase was dried over anhydrous Na_2SO_4. The solvent was evaporated on a rotary vacuum evaporator and the residue was dried under vacuum. Pentafluorocyclobutene **11a** was obtained as a yellowish liquid (0.80 g, 35%).

^{1}**H NMR** (400 MHz, $CDCl_3$): δ 1.88 (m, C**H**$_2$, 2H), 2.19 (m, C**H**$_2$CH=CH$_2$, 2H), 4.28 (td, $^3J_{HH}$ = 6.4 Hz, $^4J_{HH}$ = 1.4 Hz, C**H**$_2$O, 2H), 5.05 (dm, $^3J_{HH}$ = 10.4 Hz, CH=CH**H**^{cis}, 1H), 5.08 (dm, $^3J_{HH}$ = 16.9 Hz, CH=CH**H**^{trans}, 1H), 5.78 (ddt, $^3J_{HH}$ = 17.0 Hz, $^3J_{HH}$ = 10.2 Hz, $^3J_{HH}$ = 6.7 Hz, C**H**=CH$_2$, 1H) ppm. ^{19}**F NMR** (282 MHz, $CDCl_3$): δ −116.4 (m, C**F**$_2$, 2F), −119.4 (m, C**F**$_2$, 2F), -141.7 (m, C**F**, 1F) ppm. ^{13}**C NMR** (101 MHz, $CDCl_3$): δ 28.0 (s, **C**H$_2$, 1C), 29.2 (s, **C**H$_2$CH=CH$_2$, 1C), 73.0 (s, O**C**H$_2$, 1C), 114.5 (tdt, $^1J_{FC}$ = 280 Hz, $^2J_{FC}$ = 43 Hz, $^3J_{FC}$ = 23 Hz, **C**F$_2$, 1C), 115.0 (tdt, $^1J_{FC}$ = 274 Hz, $^2J_{FC}$ = 19 Hz, $^2J_{FC}$ = 22 Hz, **C**F$_2$, 1C), 116.1 (s, **C**H$_2$=CH, 1C), 130.4 (dtt, $^1J_{FC}$ = 325 Hz, $^2J_{FC}$ = 27 Hz, $^3J_{FC}$ = 25 Hz, **C**F, 1C), 136.4 (s, **C**H=CH$_2$, 1C), 136.6 (m, **C**-OR, 1C) ppm. **HRMS (EI):** Calculated for $C_9H_9F_5O$ $[M]^+$ 228.0574, found 228.0565.

{1-[(2,3,3,4,4-Pentafluorocyclobut-1-en-1-yl)oxy]pent-4-en-1-yl}benzene (**11b**)

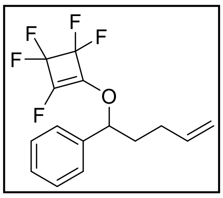

Perfluorocyclobutene (**1**, 0.49 g, 3.0 mmol) was condensed into a 25mL flask via a low-temperature addition funnel cooled to -50 °C. It was dissolved in Et_2O (5 mL) and subsequently 1-phenylpent-4-en-1-ol (0.41 g, 2.5 mmol) and KOH (0.28 g, 5.0 mmol) were added. The reaction mixture was stirred at 35°C for 24 h, then cooled to rt, washed with water (3 × 20 mL) and the organic phase was dried over anhydrous Na_2SO_4. The solvent was evaporated on a rotary vacuum evaporator and the residue was purified by column chromatography (*n*-hexane/EtOAc, 9:1). Pentafluorocyclobutene **11b** was obtained as a colourless liquid (0.21 g, 28%).

^{1}H NMR (400 MHz, $CDCl_3$): δ 1.85-1.98 (m, C**H$_2$**, 1H), 2.09-2.21 (m, C**H$_2$**, 3H), 5.01-5.08 (m, CH=C**H$_2$**, 2H), 5.19 (m, C**H**O, 1H), 5.78 (m, C**H**=CH$_2$, 1H), 7.27-7.33 (m, ArC**H**, 2H), 7.34-7.43 (m, ArC**H**, 3H) ppm. **^{19}F NMR** (376 MHz, $CDCl_3$): δ (−115.8)-(−117.2) (m, C**F$_2$**, 2F), −119.9 (m, C**F$_2$**, 2F), -138.9 (m, C**F**, 1F) ppm. **^{13}C NMR** (100 MHz, $CDCl_3$, ^{1}H and ^{19}F decoupling): δ 29.5 (s, **C**H$_2$, 1C), 36.4 (s, **C**H$_2$, 1C), 86.0 (s, O**C**H, 1C), 114.6 (s, **C**F$_2$, 1C), 115.0 (s, **C**F$_2$, 1C), 116.3 (s, **C**H$_2$=CH, 1C), 126.2 (s, Ar**C**H, 2C), 129.0 (s, Ar**C**H, 2C), 129.3 (s, *para*-Ar**C**H, 1C), 130.6 (s, **C**F, 1C), 135.8 (s, CF=**C**O, 1C), 136.6 (s, **C**H=CH$_2$, 1C), 138.2 (s, *ipso*-Ar**C**, 1C) ppm. **HRMS (CI):** Calculated for $C_{15}H_{14}F_5O$ $[M+H]^+$ 305.0959, found 305.0948.

1-[(But-3-en-1-yl)oxy]-2,3,3,4,4-pentafluorocyclobutene (**2a**)

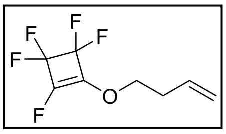

Perfluorocyclobutene (**1**, 3.0 g, 18 mmol) was condensed into a 100mL flask via a low-temperature addition funnel cooled to -50 °C. It was dissolved in Et_2O (20 mL) at 0 °C and subsequently but-3-en-1-ol (1.4 g, 18 mmol) and triethylamine (2.6 mL, 18 mmol) were added. The reaction mixture was stirred at rt for 2 days, washed with water and the organic phase was dried over anhydrous Na_2SO_4. The solvent was evaporated on a rotary vacuum evaporator and the residue was dried under vacuum. Pentafluorocyclobutene **2a** was obtained as a yellowish liquid (2.0 g, 52%).

^{1}H NMR (400 MHz, $CDCl_3$): δ 2.52 (dtm, $^{3}J_{HH}$ = 6.8 Hz, $^{3}J_{HH}$ = 6.7 Hz, C**H$_2$**CH=CH$_2$, 2H), 4.30 (dt, $^{3}J_{HH}$ = 6.7 Hz, $^{4}J_{HH}$ = 1.0 Hz, C**H$_2$**O, 2H), 5.17 (dm, $^{3}J_{HH}$ = 10.3 Hz, CH=CH**H**^{cis}, 1C), 5.18 (dm, $^{3}J_{HH}$ = 17.1 Hz, CH=CH**H**^{trans}, 1H), 5.79 (ddt, $^{3}J_{HH}$ = 17.7 Hz, $^{3}J_{HH}$ = 10.3 Hz, $^{3}J_{HH}$ = 6.8 Hz, C**H**=CH$_2$, 1H) ppm. **^{19}F NMR** (282 MHz, $CDCl_3$): δ −116.5 (m, C**F$_2$**, 2F), −119.3 (m, C**F$_2$**, 2F), −141.3 (m, C**F**, 1F) ppm. **^{13}C NMR** (101 MHz, $CDCl_3$): δ 33.4 (s, **C**H$_2$CH=CH$_2$, 1C), 72.8 (s, O**C**H$_2$, 1C), 114.6 (ttd, $^{1}J_{FC}$ = 280 Hz, $^{2}J_{FC}$ = 22 Hz, $^{3}J_{FC}$ = 20 Hz, **C**F$_2$, 1C), 115.2 (tdt, $^{1}J_{FC}$ = 275 Hz, $^{2}J_{FC}$ = 42 Hz, $^{2}J_{FC}$ = 23 Hz, **C**F$_2$, 1C), 118.9 (s, C**H**=CH$_2$, 1C), 130.7 (dtt, $^{1}J_{FC}$ = 326 Hz, $^{2}J_{FC}$ = 27 Hz, $^{3}J_{FC}$ = 25 Hz, **C**F, 1C), 132.0 (s, CH=C**H$_2$**, 1C), 136.6 (m, **C**-OR, 1C) ppm. **HRMS (CI):** Calculated for $C_8H_8F_5O$ $[M+H]^+$ 215.0495, found 215.0497.

{1-[(2,3,3,4,4-Pentafluorocyclobut-1-en-1-yl)oxy]but-3-en-1-yl}benzene (**2b**)

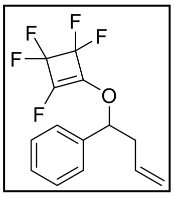

Perfluorocyclobutene (**1**, 2.0 g, 12.4 mmol) was condensed into a 50mL flask via a low-temperature addition funnel cooled to -50 °C. It was dissolved in Et_2O (20 mL) and subsequently 1-phenylbut-3-en-1-ol (1.84 g, 12.4 mmol) and KOH (1.40 g, 24.8 mmol) were added. The reaction mixture was stirred at 35 °C for 24 h, then cooled to rt, washed with water (3 × 20 mL) and the organic phase was dried over anhydrous Na_2SO_4. The solvent was evaporated on a rotary vacuum evaporator and the residue was purified by column chromatography (*n*-hexane/EtOAc, 9:1 → 4:1). Pentafluorocyclobutene **2b** was obtained as a yellowish liquid (1.44 g, 40%).

^{1}H NMR (400 MHz, $CDCl_3$): δ 2.63 (m, C**H$_2$**CH=CH$_2$, 1H), 2.80 (m, C**H$_2$**CH=CH$_2$, 1H), 5.12 – 5.19 (m, CH=C**H$_2$**, 2H), 5.23 (m, OC**H**CH$_2$, 1H), 5.71 (m, C**H**=CH$_2$, 1H), 7.30 – 7.35 (m, *ortho*-^{Ar}C**H**, 2H), 7.38 – 7.45 (m, ^{Ar}C**H**, 3H) ppm. **^{19}F NMR** (282 MHz, $CDCl_3$): δ −116.6 (m, C**F$_2$**, 2F), −119.9 (m, C**F**$_2$, 2F), −138.3 (m, C**F**, 1F) ppm. **^{13}C NMR** (101 MHz, $CDCl_3$): δ 41.6 (s, **C**H$_2$CH=CH$_2$, 1C), 85.9 (s, **C**HO, 1C), 114,5 (tdt, $^1J_{FC}$ = 274 Hz, $^3J_{FC}$ = 20 Hz, $^2J_{FC}$ = 23 Hz, **C**F$_2$, 1C), 114.9 (tdt, $^1J_{FC}$ = 280 Hz, $^2J_{FC}$ = 43 Hz, $^2J_{FC}$ = 23 Hz, **C**F$_2$, 1C), 119.4 (s, CH=**C**H$_2$, 1C), 126.1 (s, *ortho*-^{Ar}**C**H, 2C), 128.8 (s, *meta*-^{Ar}**C**H, 2C), 129.2 (s, *para*-^{Ar}**C**H, 1C), 130.8 (dm, $^1J_{FC}$ = 353 Hz, **C**F=COR, 1C), 131.7 (s, **C**H=CH$_2$, 1C), 135.6 (m, CF=**C**OR, 1C), 137.6 (s, *ipso*-^{Ar}**C**, 1C) ppm. **HRMS (CI):** Calculated for $C_{14}H_{11}F_5O$ $[M]^+$ 290.0730, found 290.0729.

1-{1-[(Perfluorocyclobut-1-en-1-yl)oxy]but-3-en-1-yl}-4-methylbenzene (**2c**)

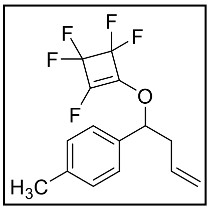

Perfluorocyclobutene (**1**, 1.0 g, 6.2 mmol) was condensed into a 50mL flask via a low-temperature addition funnel cooled to -50 °C. It was dissolved in Et_2O (20 mL) and subsequently 1-(4-methylphenyl)but-3-en-1-ol (1.0 g, 6.2 mmol) and KOH (0.70 g, 12 mmol) were added. The reaction mixture was stirred at 35 °C for 24 h, then cooled to rt, washed with water (3 × 20 mL) and the organic phase was dried over anhydrous Na_2SO_4. The solvent was evaporated on a rotary vacuum evaporator and the residue was purified by column chromatography (*n*-hexane/EtOAc, 4:1). Pentafluorocyclobutene **2c** was obtained as a colourless liquid (0.90 g, 48%).

^{1}H NMR (400 MHz, $CDCl_3$): δ 2.37 (s, C**H$_3$**, 3H), 2.60 (m, C**H$_2$**, 1H), 2.78 (m, C**H$_2$**, 1H), 5.10 – 5.23 (m, CH=C**H$_2$** a OC**H**, 3H), 5.70 (m, C**H**=CH$_2$, 1H), 7.21 (s, ^{Ar}C**H**, 4H) ppm. **^{19}F NMR** (376 MHz, $CDCl_3$): δ (−115.7) - (−117.3) (m, C**F**$_2$, 2F), (−119.7) - (−120.1) (m, C**F**$_2$, 2F), −138.3 (m, C**F**, 1F) ppm. **^{13}C NMR** (101 MHz, ^{1}H and ^{19}F decoupling, $CDCl_3$): δ 21.4 (s, **C**H$_3$, 1C), 41.7 (s, **C**H$_2$CH=CH$_2$, 1C), 86.0 (s, **C**HO, 1C), 114.6 (s, **C**F$_2$, 1C), 115.1 (s, **C**F$_2$, 1C), 119.4 (s, CH=**C**H$_2$, 1C), 126.3 (s, ^{Ar}**C**H, 2C), 129.7 (s, ^{Ar}**C**H, 2C), 130.6 (s, **C**F=COR, 1C), 132.1 (s, **C**H=CH$_2$, 1C), 134.8 (s, *ipso*-^{Ar}**C**, 1C), 135.8 (m, CF=**C**OR, 1C), 139.3 (s, **C**-CH$_3$, 1C) ppm. **HRMS (CI):** Calculated for $C_{15}H_{14}F_5O$ $[M+H]^+$ 305.0959, found 305.0951.

1-{1-[(Perfluorocyclobut-1-en-1-yl)oxy]but-3-en-1-yl}-4-(trifluoromethyl)-benzene (**2d**)

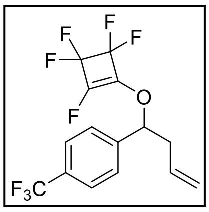

Perfluorocyclobutene (**1**, 1.0 g, 6.2 mmol) was condensed into a 50mL flask via a low-temperature addition funnel cooled to -50 °C. It was dissolved in Et_2O (20 mL) and subsequently 1-(4-(trifluoromethyl)phenyl)but-3-en-1-ol (1.34 g, 6.2 mmol) and KOH (700 mg, 12.4 mmol) were added. The reaction mixture was stirred at 35 °C for 24 h, then cooled to rt, washed with water (3 × 20 mL) and the organic phase was dried over anhydrous Na_2SO_4. The solvent was evaporated on a rotary vacuum evaporator and the residue was dried under vacuum. Pentafluorocyclobutene **2d** was obtained as a yellowish liquid (1.9 g, 85%).

^{1}H NMR (400 MHz, $CDCl_3$): δ 2.12 – 2.22 (m, C**H**$_2$CH=CH$_2$, 1H), 2.28 – 2.39 (m, C**H**$_2$CH=CH$_2$, 1H), 4.67 – 4.76 (m, CH=C**H$_2$**, 2H), 4.83 (t, $^3J_{HH}$ = 6.6 Hz, OC**H**CH$_2$, 1H), 5.24 (m, C**H**=CH$_2$, 1H), 6.99 (m, ^{Ar}C**H**, 2H), 7.23 (m, ^{Ar}C**H**, 2H) ppm. **^{19}F NMR** (376 MHz, $CDCl_3$): δ −63.3 (s, C**F**$_3$, 3F), (−115.9) - (−117.4) (m, C**F**$_2$, 2F), (−119.4) - (−119.8) (m, C**F**$_2$, 2F), −138.3 (m, C**F**, 1F) ppm. **^{13}C NMR** (101 MHz, $CDCl_3$): δ 41.5 (s, **C**H$_2$CH=CH$_2$, 1C), 84.9 (s, **C**HO, 1C), 110.9 – 118.0 (m, **C**F$_2$, 2C), 120.1 (s, CH=**C**H$_2$, 1C), 123.8 (q, $^1J_{FC}$ = 272 Hz, **C**F$_3$, 1C), 125.9 (q, $^2J_{FC}$ = 4 Hz, **C**-CF$_3$, 1C), 126.5 (s, *ortho*-^{Ar}**C**H, 2C), 130.9 (s, **C**H=CH$_2$, 1C), 131.0 (dm, $^1J_{FC}$ = 326 Hz, **C**F=COR, 1C), 131.4 (q, $^3J_{FC}$ = 33 Hz, *meta*-^{Ar}**C**H, 2C), 135.4 (m, CF=**C**OR, 1C), 141.5 (s, *ipso*-^{Ar}**C**, 1C) ppm. **HRMS (CI):** Calculated for $C_{15}H_{11}F_8O$ $[M+H]^+$ 359.0677, found 359.0663.

1-Methoxy-3-{1-[(perfluorocyclobut-1-en-1-yl)oxy]but-3-en-1-yl}benzene (**2e**)

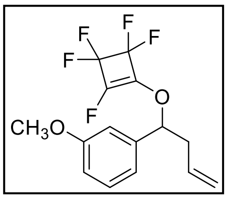

Perfluorocyclobutene (**1**, 2.00 g, 12.4 mmol) was condensed into a 50mL flask via a low-temperature addition funnel cooled to -50 °C. It was dissolved in Et_2O (20 mL) and subsequently 1-(3-methoxyphenyl)but-3-en-1-ol (2.22 g, 12.4 mmol) and KOH (1.40 g, 24.8 mmol) were added. The reaction mixture was stirred at 35 °C for 24 h, then cooled to rt, washed with water (3 × 20 mL) and the organic phase was dried over anhydrous Na_2SO_4. The solvent was evaporated on a rotary vacuum evaporator and the residue was purified by column chromatography (*n*-hexane/EtOAc, 4:1). Pentafluorocyclobutene **2e** was obtained as a colourless liquid (2.6 g, 66%).

^{1}H NMR (400 MHz, $CDCl_3$): δ 2.61 (m, C**H**$_2$CH=CH$_2$, 1H), 2.77 (m, C**H**$_2$CH=CH$_2$, 1H), 3.82 (s, C**H**$_3$O, 3H), 5.12 – 5.19 (m, CH=C**H$_2$**, 2H), 5.20 (dd, $^3J_{HH}$ = 10.0 Hz, $^3J_{HH}$ = 5.7 Hz, OC**H**, 1H), 5.71 (m, C**H**=CH$_2$, 1H), 6.84 (m, ^{Ar}C**H**, 1H), 6.87 – 6.94 (m, ^{Ar}C**H**, 2H), 7.31 (m, ^{Ar}C**H**, 1H) ppm. **^{19}F NMR** (376 MHz, $CDCl_3$): δ (−115.8) - (−117.2) (m, C**F**$_2$, 2F), −119.9 (m, C**F**$_2$, 2F), −138.6 (m, C**F**, 1F) ppm. **^{13}C NMR** (101 MHz, $CDCl_3$): δ 41.5 (s, **C**H$_2$, 1C), 55.3 (s, **C**H$_3$O, 1C), 85.7 (s, **C**HO, 1C), 110.0 – 119.2 (m, **C**F$_2$, 2C), 111.6 (s, ^{Ar}**C**H, 1C), 114.5 (s, ^{Ar}**C**H, 1C), 118.4 (s, ^{Ar}**C**H, 1C), 119.3 (s, CH=**C**H$_2$, 1C), 129.9 (s, ^{Ar}**C**H, 1C), 130.6 (dm, $^1J_{FC}$ = 326 Hz, **C**F, 1C), 131.7 (s, **C**H=CH$_2$, 1C), 135.6 (m, **C**O, 1C), 139.2 (s, ^{Ar}**C**, 1C), 159.8 (s, ^{Ar}**C**-OCH$_3$, 1C) ppm. **HRMS (EI):** Calculated for $C_{15}H_{13}F_5O_2$ $[M]^+$ 320.0830, found 320.0837.

2-{1-[(Perfluorocyclobut-1-en-1-yl)oxy]but-3-en-1-yl}pyridine (**2f**)

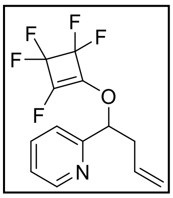

Perfluorocyclobutene (**1**, 1.0 g, 6.2 mmol) was condensed into a 50mL flask via a low-temperature addition funnel cooled to -50 °C. It was dissolved in Et_2O (20 mL) and subsequently 1-(pyridin-2-yl)but-3-en-1-ol (0.93 g, 6.2 mmol) and KOH (700 mg, 12.4 mmol) were added. The reaction mixture was stirred at 35 °C for 24 h, then cooled to rt, washed with water (3 × 20 mL) and the organic phase was dried over anhydrous Na_2SO_4. The solvent was evaporated on a rotary vacuum evaporator and the residue was purified by column chromatography (*n*-hexane/EtOAc, 4:1). Pentafluorocyclobutene **2f** was obtained as a colourless liquid (0.64 g, 35%).

^{1}H NMR (400 MHz, $CDCl_3$): δ 2.79 (m, C**H**$_2$CH=CH$_2$, 2H), 5.09 – 5.20 (m, CH=C**H$_2$**, 2H), 5.38 (t, $^3J_{HH}$ = 6.1 Hz, OC**H**CH$_2$, 1H), 5.75 (m, C**H**=CH$_2$, 1H), 7.28 (m, ^{Ar}C**H**, 1H), 7.34 (m, ^{Ar}C**H**, 1H), 7.76 (m, ^{Ar}C**H**, 1H), 8.61 (m, ^{Ar}C**H**, 1H) ppm. **^{19}F NMR** (376 MHz, $CDCl_3$): δ −116.6 (m, C**F**$_2$, 2F), −119.6 (m, C**F**$_2$, 2F), −138.0 (m, C**F**, 1F) ppm. **^{13}C NMR** (101 MHz, $CDCl_3$): δ 39.9 (s, **C**H$_2$CH=CH$_2$, 1C), 85.7 (s, **C**HO, 1C), 110.9 – 118.3 (m, **C**F$_2$, 2C), 119.6 (s, CH=**C**H$_2$, 1C), 120.3 (s, ^{Ar}**C**H, 1C), 123.8 (s, ^{Ar}**C**H, 1C), 130.8 (dm, $^1J_{FC}$ = 327 Hz, **C**F, 1C), 131.3 (s, **C**H=CH$_2$, 1C), 137.2 (s, ^{Ar}**C**, 1C), 135.6 (m, **C**O, 1C), 149.6 (s, ^{Ar}**C**H, 1C), 156.7 (s, ^{Ar}**C**, 1C) ppm. **HRMS (ESI):** Calculated for $C_{13}H_{11}F_5NO$ $[M+H]^+$ 292.0755, found 292.0755.

1,3,3,4,4-Pentafluoro-2-[(3-methylbut-3-en-1-yl)oxy]cyclobutene (**2g**)

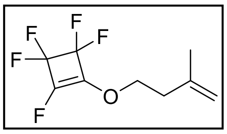

Perfluorocyclobutene (**1**, 4.9 g, 30 mmol) was condensed into a 100mL flask via a low-temperature addition funnel cooled to -50 °C. It was dissolved in Et_2O (20 mL) at 0 °C and subsequently 3-methylbut-3-en-1-ol (3.3 g, 39 mmol) and triethylamine (5.4 mL, 39 mmol) were added. The reaction mixture was stirred at rt for 2 days, washed with water (3 × 10 mL) and the organic phase was dried over anhydrous Na_2SO_4. The solvent was evaporated on a rotary vacuum evaporator and the residue was purified by column chromatography (*n*-hexane/EtOAc, 4:1). Pentafluorocyclobutene **2g** was obtained as a colourless liquid (5.3 g, 59%).

^{1}H NMR (300 MHz, $CDCl_3$): δ 1.78 (s, CH$_3$, 3H), 2.47 (m, C**H**$_2$C=CH$_2$, 2H), 4.36 (m, C**H**$_2$O, 2H), 4.78 (m, C=C**H**H, 1H), 4.89 (m, C=C**H**H, 1H) ppm. **^{19}F NMR** (282 MHz, $CDCl_3$): δ −116.4 (m, CF$_2$, 2F), −119.3 (m, CF$_2$, 2F), -141.4 (m, CF, 1F) ppm. **^{13}C NMR** (101 MHz, $CDCl_3$): δ 22.3 (s, **C**H$_3$, 1H), 36.9 (s, **C**H$_2$C=CH$_2$, 1C), 71.9 (s, O**C**H$_2$, 1C), 113.5 (s, C=**C**H$_2$, 1C), 114.5 (tdt, $^1J_{FC}$ = 281 Hz, $^2J_{FC}$ = 43 Hz, $^2J_{FC}$ = 23 Hz, **C**F$_2$CF, 1C), 115.5 (tdt, $^1J_{FC}$ = 274 Hz, $^2J_{FC}$ = 22 Hz, $^3J_{FC}$ = 19 Hz, **C**F$_2$CO, 1C), 130.5 (dtt, $^1J_{FC}$ = 325 Hz, $^2J_{FC}$ = 27 Hz, $^3J_{FC}$ = 25 Hz, **C**F=CO, 1C), 136.5 (m, **C**-OR, 1C), 139.8 (s, **C**=CH$_2$, 1C) ppm. **HRMS (CI):** Calculated for $C_9H_{10}F_5O$ $[M+H]^+$ 229.0652, found 229.0654.

4. Synthesis of tetrafluorocyclobutenes 9, 10, 12, 3 and 15

Phenyl(3,3,4,4-tetrafluorocyclobut-1-en-1-yl)sulfane (**9a**)

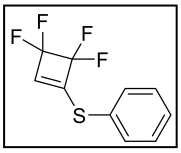

Pentafluorocyclobutene **5a** (0.40 g, 1.6 mmol) was dissolved in dry Et_2O (10 mL) under an inert atmosphere. The solution was cooled to -78 °C and $LiAlH_4$ solution (4M in Et_2O, 0.40 mL, 1.6 mmol) was slowly added. The reaction mixture was stirred at -78 °C for 1 h and then quenched with water (10 mL). It was extracted with Et_2O (3 × 10 mL), the combined organic layers were washed with brine (10 mL) and dried over anhydrous $MgSO_4$. The solvent was evaporated on a rotary vacuum evaporator and the residue was dried under vacuum. Tetrafluorocyclobutene **9a** was obtained as colourless liquid (0.26 g, 69%).

^{1}H NMR (400 MHz, $CDCl_3$): δ 6.05 (tt, $^3J_{HF}$ = 10.8 Hz, $^4J_{HF}$ = 1.7 Hz, **H**C=CS, 1H), 7.42 – 7.50 (m, ^{Ar}C**H**, 3H), 7.56 – 7.61 (m, ^{Ar}C**H**, 2H) ppm. **^{19}F NMR** (282 MHz, $CDCl_3$): δ −109.5 (m, C**F**$_2$, 2F), −113.6 (m, C**F**$_2$, 2F) ppm. **^{13}C NMR** (101 MHz, $CDCl_3$): δ 118.0 (tt, $^1J_{FC}$ = 282 Hz, $^2J_{FC}$ = 24 Hz, **C**F$_2$, 1C), 118.4 (tt, $^1J_{FC}$ = 290 Hz, $^2J_{FC}$ = 25 Hz, **C**F$_2$, 1C), 128.9 (tt, $^2J_{FC}$ = 26 Hz, $^3J_{FC}$ = 13 Hz, H**C**=CS, 1C), 130.1 (s, ^{Ar}**C**H, 2C), 130.5 (s, *para*-^{Ar}CH, 1C), 134.2 (s, ^{Ar}**C**H, 2C), 125.9 (s, *ipso*-^{Ar}**C**, 1C), 154.8 (tt, $^2J_{FC}$ = 24 Hz, $^3J_{FC}$ = 16 Hz, HC=**C**S, 1C) ppm. **HRMS (CI):** Calculated for $C_{10}H_6F_4S$ $[M]^+$ 234.0126, found 234.0124.

Benzyl(3,3,4,4-tetrafluorocyclobut-1-en-1-yl)sulfane (**9b**)

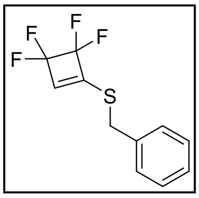

Pentafluorocyclobutene **5b** (1.0 g, 3.8 mmol) was dissolved in anhydrous Et_2O (30 mL) under an inert atmosphere. The solution was cooled to -78 °C and $LiAlH_4$ solution (4M in Et_2O, 0.95 mL, 3.8 mmol) was slowly added. The reaction mixture was stirred at -78 °C for 1 h and then quenched with water (10 mL). It was extracted with Et_2O (3 × 20 mL), the combined organic layers were washed with brine (10 mL) and dried over anhydrous $MgSO_4$. The solvent was evaporated on a rotary vacuum evaporator and the residue was dried under vacuum. Tetrafluorocyclobutene **9b** was obtained as colourless liquid (0.66 g, 71%).

^{1}H NMR (400 MHz, $CDCl_3$): δ 4.16 (s, C**H**$_2$, 2H), 6.29 (tt, $^3J_{HF}$ = 10.7 Hz, $^4J_{HF}$ = 1.8 Hz, **H**C=CS, 1H), 7.28 – 7.44 (m, ^{Ar}C**H**, 5H) ppm. **^{19}F NMR** (282 MHz, $CDCl_3$): δ −108.9 (m, C**F**$_2$, 2F), −112.8 (m, C**F**$_2$, 2F) ppm. **^{13}C NMR** (101 MHz, $CDCl_3$): δ 35.9 (s, **C**H$_2$, 1C), 118.5 (tt, $^1J_{FC}$ = 281 Hz, $^2J_{FC}$ = 24 Hz, **C**F$_2$, 1C), 119.1 (tt, $^1J_{FC}$ = 291 Hz, $^2J_{FC}$ = 25 Hz, **C**F$_2$, 1C), 128.3 (s, *para*-^{Ar}**C**H, 1C), 128.8 (s, ^{Ar}**C**H, 2C), 128.8 (m, HC=**C**S, 1C), 129.1 (s, ^{Ar}**C**H, 2C), 134.2 (s, *ipso*-^{Ar}**C**, 1C), 153.8 (tt, $^2J_{FC}$ = 24 Hz, $^3J_{FC}$ = 16 Hz, H**C**=CS, 1C) ppm. **HRMS (CI):** Calculated for $C_{11}H_9F_4S$ $[M+H]^+$ 249.0361, found 249.0358.

1-Butoxy-3,3,4,4-tetrafluorocyclobutene (**10a**)

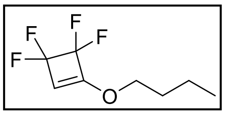

Pentafluorocyclobutene **7a** (1.0 g, 4.6 mmol) was dissolved in dry Et_2O (30 mL) under an inert atmosphere. The solution was cooled to -78 °C and $LiAlH_4$ solution (4M in Et_2O, 1.2 mL, 4.8 mmol) was slowly added. The reaction mixture was stirred at rt for 3 h and then quenched with water (10 mL). It was extracted with Et_2O and the organic phase

was dried over anhydrous $MgSO_4$. The solvent was evaporated on a rotary vacuum evaporator and the residue was dried under vacuum. Tetrafluorocyclobutene **10a** was obtained as colourless liquid (0.47 g, 52%).

^{1}H NMR (400 MHz, $CDCl_3$)**:** δ 0.96 (t, $^3J_{HH}$ = 7.4 Hz, C**H**$_3$, 3H), 1.45 (m, C**H**$_2$, 2H), 1.75 (m, C**H**$_2$, 2H), 4.05 (t, $^3J_{HH}$ = 6.5 Hz, C**H**$_2$O, 2H), 5.40 (tt, $^3J_{HF}$ = 10.3 Hz, $^4J_{HF}$ = 2.3 Hz, **H**C=CO, 1H) ppm. **^{19}F NMR** (282 MHz, $CDCl_3$): δ −109.2 (m, C**F**$_2$, 2F), −118.5 (m, C**F**$_2$, 2F) ppm. **^{13}C NMR** (101 MHz, $CDCl_3$): δ 13.5 (s, **C**H$_3$, 1C), 18.7 (s, **C**H$_2$, 1C), 30.3 (s, **C**H$_2$, 1C), 72.1 (s, O**C**H$_2$, 1C), 106.5 (tt, $^2J_{FC}$ = 27 Hz, $^3J_{FC}$ = 14 Hz, H**C**=CO, 1C), 117.5 (tt, $^1J_{FC}$ = 275 Hz, $^2J_{FC}$ = 24 Hz, **C**F$_2$, 1C), 117.7 (tt, $^1J_{FC}$ = 287 Hz, $^2J_{FC}$ = 25 Hz, **C**F$_2$, 1C), 159.5 (tt, $^2J_{FC}$ = 24 Hz, $^3J_{FC}$ = 20 Hz, HC=**C**O, 1C) ppm. **HRMS (EI):** Calculated for $C_8H_{10}F_4O$ $[M]^+$ 198.0668, found 198.0670.

[(3,3,4,4-Tetrafluorocyclobut-1-en-1-yl)oxy]benzene (**10b**)

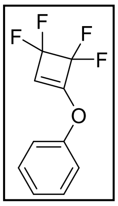

Pentafluorocyclobutene **7b** (0.46 g, 2.1 mmol) was dissolved in dry Et_2O (15 mL) under an inert atmosphere. The solution was cooled to -78 °C and $LiAlH_4$ solution (2M in THF, 1.1 mL, 2.1 mmol) was slowly added. The reaction mixture was stirred at rt for 1 h and then quenched with water (10 mL). It was extracted with Et_2O and the organic phase was dried over anhydrous $MgSO_4$. The solvent was evaporated on a rotary vacuum evaporator and the residue was purified by column chromatography (*n*-hexane/DCM, 4:1). Tetrafluorocyclobutene **10b** was obtained as colourless liquid (0.16 g, 34%).

^{1}H NMR (400 MHz, $CDCl_3$): δ 5.45 (tt, $^3J_{HF}$ = 10.0 Hz, $^4J_{HF}$ = 2.0 Hz, **H**C=CO, 1H), 7.22 (m, *ortho*-^{Ar}C**H**, 2H), 7.33 (m, *para*-^{Ar}C**H**, 1H), 7.46 (m, *meta*-^{Ar}C**H**, 2H) ppm. **^{19}F NMR** (282 MHz, $CDCl_3$): δ −110.1 (m, C**F**$_2$, 2F), −118.3 (m, C**F**$_2$, 2F) ppm. **^{13}C NMR** (101 MHz, $CDCl_3$): δ 109.8 (tt, $^2J_{FC}$ = 27 Hz, $^3J_{FC}$ = 13 Hz, H**C**=CO, 1C), 117.3 (tt, $^1J_{FC}$ = 276 Hz, $^2J_{FC}$ = 23 Hz, **C**F$_2$, 1C), 117.4 (tt, $^1J_{FC}$ = 287 Hz, $^2J_{FC}$ = 25 Hz, **C**F$_2$, 1C), 119.3 (s, *ortho*-^{Ar}**C**H, 2C), 126.8 (s, *para*-^{Ar}**C**H, 1C), 130.3 (s, *meta*-^{Ar}**C**H, 2C), 153.1 (s, *ipso*-^{Ar}**C**, 1C), 157.7 (tt, $^2J_{FC}$ = 24 Hz, $^3J_{FC}$ = 21 Hz, HC=**C**O, 1C) ppm. **HRMS (CI):** Calculated for $C_{10}H_7F_4O$ $[M+H]^+$ 219.0432, found 219.0433.

{[(3,3,4,4-Tetrafluorocyclobut-1-en-1-yl)oxy]methyl}benzene (**10c**)

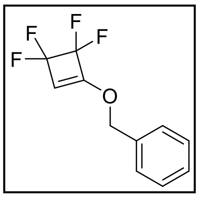

Pentafluorocyclobutene **7c** (0.2 g, 0.8 mmol) was dissolved in dry Et_2O (5 mL) under an inert atmosphere. The solution was cooled to -78 °C and $LiAlH_4$ solution (2M in THF, 0.4 mL, 0.8 mmol) was slowly added. The reaction mixture was stirred at rt for 1 h and then quenched with water (10 mL). It was extracted with Et_2O and the organic phase was dried over anhydrous $MgSO_4$. The solvent was evaporated on a rotary vacuum evaporator and the residue was dried under vacuum. Tetrafluorocyclobutene **10c** was obtained as colourless liquid (0.14 g, 73%).

^{1}H NMR (400 MHz, $CDCl_3$): δ 5.08 (s, C**H**$_2$, 2H), 5.46 (tt, $^3J_{HF}$ = 10.2 Hz, $^4J_{HF}$ = 2.4 Hz, **H**C=CO, 1H), 7.34 – 7.47 (m, ^{Ar}C**H**, 5H) ppm. **^{19}F NMR** (282 MHz, $CDCl_3$): δ −109.4 (m, C**F**$_2$, 2F), −118.3 (m, C**F**$_2$, 2F) ppm. **^{13}C NMR** (101 MHz, $CDCl_3$): δ 73.9 (s, O**C**H$_2$, 1C), 107.7 (tt, $^2J_{FC}$ = 27 Hz, $^3J_{FC}$ = 14 Hz, $^2J_{FC}$ = 14 Hz, H**C**=CO, 1C), 117.57 (tt, $^1J_{FC}$ = 275 Hz, $^2J_{FC}$ = 23 Hz, **C**F$_2$, 1C), 117.59 (tt, $^1J_{FC}$ = 287 Hz, $^2J_{FC}$ = 25 Hz, **C**F$_2$, 1C), 128.0 (s, *ortho*-^{Ar}**C**H, 2C), 128.9 (s, *meta*-^{Ar}**C**H, 2C), 129.3 (s, *para*-^{Ar}**C**H, 1C), 133.3 (s, *ipso*-^{Ar}**C**, 1C), 159.1 (tt, $^2J_{FC}$ = 24 Hz, $^3J_{FC}$ = 20 Hz, HC=**C**O, 1C) ppm. **HRMS (ESI):** Calculated for $C_{11}H_7F_4O$ [M-H]$^-$ 231.0439, found 231.0445.

1-Methoxy-4-{[(3,3,4,4-tetrafluorocyclobut-1-en-1-yl)oxy]methyl}benzene (**10d**)

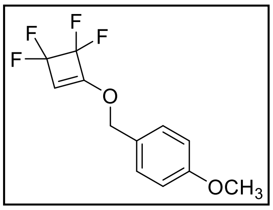

Pentafluorocyclobutene **7d** (0.28 g, 1.0 mmol) was dissolved in dry Et_2O (5 mL) under an inert atmosphere. The solution was cooled to -78 °C and $LiAlH_4$ solution (4M in Et_2O, 0.25 mL, 1.0 mmol) was slowly added. The reaction mixture was stirred at rt for 2 h and then quenched with water (10 mL). It was extracted with Et_2O and the organic phase was dried over anhydrous $MgSO_4$. The solvent was evaporated on a rotary vacuum evaporator and the residue was purified by column chromatography (*n*-hexane/EtOAc, 9:1). Tetrafluorocyclobutene **10d** was obtained as colourless liquid (0.16 g), however, it proved to be highly unstable and in a few minutes its complete decomposition was observed. Therefore only ^{1}H and ^{19}F NMR spectra could be obtained.

^{1}H NMR (400 MHz, $CDCl_3$): δ 3.83 (s, C**H**$_3$O, 3H), 5.01 (s, C**H**$_2$, 2H), 5.44 (tt, $^3J_{HF}$ = 10.2 Hz, $^4J_{HF}$ = 2.3 Hz, **H**C=CO, 1H), 6.93 (m, ^{Ar}C**H**, 2H), 7.30 (m, ^{Ar}C**H**, 2H) ppm. **^{19}F NMR** (376 MHz, $CDCl_3$): δ −109.2 (m, C**F**$_2$, 2F), −118.2 (m, C**F**$_2$, 2F) ppm.

1-{[(2,3,3,4,4-Pentafluorocyclobut-1-en-1-yl)oxy]methyl}-4-(trifluoromethyl)benzene (**10e**)

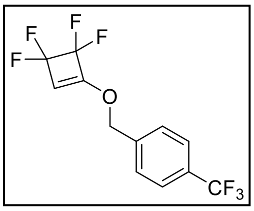

Pentafluorocyclobutene **7e** (0.32 g, 1.0 mmol) was dissolved in dry Et_2O (5 mL) under an inert atmosphere. The solution was cooled to -78 °C and $LiAlH_4$ solution (4M in Et_2O, 0.25 mL, 1.0 mmol) was slowly added. The reaction mixture was stirred at rt for 2 h and then quenched with water (10 mL). It was extracted with Et_2O and the organic phase was dried over anhydrous $MgSO_4$. The solvent was evaporated on a rotary vacuum evaporator and the residue was purified by column chromatography (*n*-hexane/EtOAc, 9:1). Tetrafluorocyclobutene **10e** was obtained as colourless liquid (0.23 g, 78%).

^{1}H NMR (400 MHz, $CDCl_3$): δ 5.13 (s, C**H**$_2$, 2H), 5.48 (tt, $^3J_{HF}$ = 10.2 Hz, $^4J_{HF}$ = 2.2 Hz, **H**C=CO, 1H), 7.48 (m, ^{Ar}C**H**, 2H), 7.68 (m, ^{Ar}C**H**, 2H) ppm. **^{19}F NMR** (376 MHz, $CDCl_3$): δ −63.3 (s, C**F**$_3$, 3F), −109.6 (m, C**F**$_2$, 2F), −118.2 (m, C**F**$_2$, 2F) ppm. **^{13}C NMR** (101 MHz, $CDCl_3$, ^{1}H and ^{19}F decoupling): δ 72.7 (s, **C**H$_2$, 1C), 108.3 (s, H**C**=CO, 1C), 117.5 (s, **C**F$_2$, 1C), 117.6 (s, **C**F$_2$, 1C), 123.9 (s, **C**F$_3$, 1C), 126.0 (s, ^{Ar}**C**H, 2C), 127.9 (s, ^{Ar}**C**H, 2C), 131.5 (s, ^{Ar}**C**-CF$_3$, 1C), 137.3 (s, *ipso*-^{Ar}C, 1C), 158.9 (s, FC=**C**O, 1C) ppm. **HRMS (EI):** Calculated for $C_{12}H_7F_7O$ [M]$^+$ 300.0380, found 300.0369.

5-[(3,3,4,4-Tetrafluorcyclobut-1-en-1-yl)oxy]pent-1-en (**12a**)

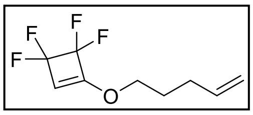

Pentafluorocyclobutene **11a** (0.50 g, 2.2 mmol) was dissolved in dry Et_2O (20 mL) under an inert atmosphere. The solution was cooled to 0 °C and $LiAlH_4$ solution (4M in Et_2O, 0.55 mL, 2.2 mmol) was slowly added. The reaction mixture was stirred at rt for 1 h and then quenched with water (10 mL). The aqueous phase was separated and washed with Et_2O (3 × 10 mL). The combined organic layers were washed with brine (10 mL) and dried over anhydrous Na_2SO_4. The solvent was evaporated on a rotary vacuum evaporator. Tetrafluorocyclobutene **12a** was obtained as a colourless liquid (0.26 g, 56%).

^{1}H NMR (400 MHz, $CDCl_3$): δ 1.88 (m, C**H**$_2$, 2H), 2.19 (m, C**H**$_2$, 2H), 4.05 (t, $^3J_{HH}$ = 6.4 Hz, C**H**$_2$O, 2H), 5.06 (dm, $^3J_{HH}$ = 17.3 Hz, CH=CH**H**^{trans}, 1H), 5.06 (dm, $^3J_{HH}$ = 10.4 Hz, CH=CH**H**^{cis}, 1H), 5.40 (tt, $^3J_{HF}$ = 10.3 Hz, $^4J_{HF}$ = 2.3 Hz, C**H**=COR), 5.79 (ddt, $^3J_{HH}$ = 17.3 Hz, $^3J_{HH}$ = 10.2 Hz, $^3J_{HH}$ = 6.7 Hz, C**H**=CH$_2$, 1H) ppm. **^{19}F NMR** (282 MHz, $CDCl_3$): δ −108.8 (m, C**F**$_2$, 2F), −117.9 (m, C**F**$_2$, 2F) ppm. **^{13}C NMR** (101 MHz, $CDCl_3$): δ 27.4 (s, **C**H$_2$, 1C), 29.4 (s, **C**H$_2$, 1C), 71.4 (s, O**C**H$_2$, 1C), 106.9 (tt, $^2J_{FC}$ = 27 Hz, $^3J_{FC}$ = 14 Hz, **C**H=COR, 1C), 116.1 (s, **C**H$_2$=CH, 1C), 117.5 (tt, $^1J_{FC}$ = 287 Hz, $^2J_{FC}$ = 25 Hz, **C**F$_2$, 1C), 117.7 (tt, $^1J_{FC}$ = 275 Hz, $^2J_{FC}$ = 23 Hz, **C**F$_2$, 1C), 136.6 (s, **C**H=CH$_2$, 1C), 159.4 (tt, J_{FC} = 24 Hz, J_{FC} = 20 Hz, **C**-OR, 1C) ppm. **HRMS (EI):** Calculated for $C_9H_{10}F_4O$ $[M]^+$ 210.0668, found 210.0669.

{1-[(3,3,4,4-Tetrafluorocyclobut-1-en-1-yl)oxy]pent-4-en-1-yl}benzene (**12b**)

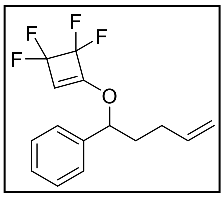

Pentafluorocyclobutene **11b** (0.15 g, 0.50 mmol) was dissolved in dry Et_2O (5 mL) under an inert atmosphere. The solution was cooled to 0 °C and $LiAlH_4$ solution (4M in Et_2O, 0.13 mL, 0.50 mmol) was slowly added. The reaction mixture was stirred at rt for 1 h and then quenched with water (5 mL). The aqueous phase was separated and washed with Et_2O (3 × 5 mL). The combined organic layers were washed with brine (10 mL) and dried over anhydrous Na_2SO_4. The solvent was evaporated on a rotary vacuum evaporator. Tetrafluorocyclobutene **12b** was obtained as a yellowish liquid (0.12 g, 84%).

^{1}H NMR (400 MHz, $CDCl_3$): δ 1.86-1.98 (m, C**H**$_2$, 1H), 2.10-2.20 (m, C**H**$_2$, 3H), 4.99 (m, C**H**O, 1H), 5.02-5.08 (m, CH=C**H**$_2$, 2H), 5.10 (tt, $^3J_{HF}$ = 9.9 Hz, $^4J_{HF}$ = 2.4 Hz, H**C**=CO, 1H), 5.79 (m, C**H**=CH$_2$, 1H), 7.27-7.29 (m, ArC**H**, 1H), 7.33-7.42 (m, ArC**H**, 4H) ppm. **^{19}F NMR** (376 MHz, $CDCl_3$): δ (−108.6)-(−109.9) (m, C**F**$_2$, 2F), (−117.9)-(−119.4) (m, CF$_2$, 2F) ppm. **^{13}C NMR** (101 MHz, $CDCl_3$, ^{1}H and ^{19}F decoupling): δ 29.4 (s, **C**H$_2$, 1C), 36.4 (s, **C**H$_2$, 1C), 84.8 (s, O**C**H, 1C), 108.3 (s, H**C**=CO, 1C), 116.2 (s, CH=**C**H$_2$, 1C), 117.8 (s, **C**F$_2$, 1C), 117.9 (s, **C**F$_2$, 1C), 126.1 (s, Ar**C**H, 2C), 129.0 (s, Ar**C**H, 1C), 129.1 (s, Ar**C**H, 2C), 136.7 (s, **C**H=CH$_2$, 1C), 138.1 (s, *ipso*-Ar**C**, 1C), 158.4 (s, HC=**C**O, 1C) ppm. **HRMS (EI):** Calculated for $C_{15}H_{14}F_4O$ $[M]^+$ 286.0975, found 286.0963.

1-(But-3-en-1-yloxy)-3,3,4,4-tetrafluorocyclobutene (**3a**)

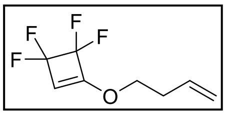

Pentafluorocyclobutene **2a** (1.0 g, 4.7 mmol) was dissolved in dry Et_2O (20 mL) under an inert atmosphere. The solution was cooled to 0 °C and $LiAlH_4$ solution (4M in Et_2O, 1.2 mL, 4.7 mmol) was slowly added. The reaction mixture was stirred at rt overnight and then quenched with water (5 mL). The aqueous phase was separated and washed with Et_2O (3 × 10 mL). The combined organic layers were washed with brine (10 mL) and dried over anhydrous $MgSO_4$. The solvent was carefully evaporated on a rotary vacuum evaporator (700 mbar, 40 °C). Tetrafluorocyclobutene **3a** was obtained as colourless liquid (0.51 g, 56%).

^{1}H NMR (400 MHz, $CDCl_3$): δ 2.53 (t, $^3J_{HH}$ = 6.6 Hz, C**H**$_2$CH=CH$_2$, 2H), 4.09 (t, $^3J_{HH}$ = 6.6 Hz, C**H$_2$**O, 2H), 5.15 (dm, $^3J_{HH}$ = 10.3 Hz, CH=CH**H**^{cis}, 1C), 5.17 (dm, $^3J_{HH}$ = 17.1 Hz, CH=CH**H**^{trans}, 1H), 5.42 (tt, $^3J_{HF}$ = 10.3 Hz, $^4J_{HF}$ = 2.3 Hz, **H**C=COR, 1H), 5.79 (ddt, $^3J_{HH}$ = 17.7 Hz, $^3J_{HH}$ = 10.3 Hz, $^3J_{HH}$ = 6.8 Hz, C**H**=CH$_2$, 1H) ppm. **^{19}F NMR** (282 MHz, $CDCl_3$): δ −109.3 (m, C**F**$_2$, 2F), −118.3 (m, C**F**$_2$, 2F) ppm. **^{13}C NMR** (101 MHz, $CDCl_3$): δ 32.6 (s, **C**H$_2$CH=CH$_2$, 1C), 71.3 (s, O**C**H$_2$, 1C), 106.9 (tt, $^2J_{FC}$ = 27 Hz, $^3J_{FC}$ = 14 Hz, **C**H=COR, 1C), 117.5 (tt, $^1J_{FC}$ = 287 Hz, $^2J_{FC}$ = 25 Hz, **C**F$_2$, 1C), 117.6 (tt, $^1J_{FC}$ = 275 Hz, $^2J_{FC}$ = 23 Hz, **C**F$_2$, 1C), 118.4 (s, CH=**C**H$_2$, 1C), 132.3 (s, **C**H=CH$_2$, 1C), 159.3 (tt, $^2J_{FC}$ = 23 Hz, $^3J_{FC}$ = 17 Hz, CH=**C**OR, 1C) ppm. **HRMS (CI):** Calculated for $C_8H_9F_4O$ $[M+H]^+$ 197.0590, found 197.0586.

1-{1-[(3,3,4,4-Tetrafluorocyclobut-1-en-1-yl)oxy]but-3-en-1-yl}benzene (**3b**)

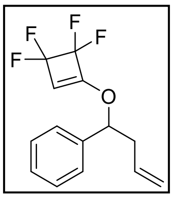

Pentafluorocyclobutene **2b** (0,29 g, 1.0 mmol) was dissolved in dry Et_2O (5 mL) under an inert atmosphere. The solution was cooled to 0 °C and $LiAlH_4$ solution (4M in Et_2O, 0.25 mL, 1.0 mmol) was slowly added. The reaction mixture was stirred at rt overnight and then quenched with water (5 mL). The aqueous phase was separated, washed with Et_2O (2 × 5 mL) and dried over anhydrous Na_2SO_4. The solvent was evaporated on a rotary vacuum evaporator and purified by column chromatography (*n*-hexane/EtOAc, 9:1). Tetrafluorocyclobutene **3b** was obtained as a yellowish liquid (87 mg, 32%).

^{1}H NMR (400 MHz, $CDCl_3$): δ 2.62 (m, C**H**$_2$CH=CH$_2$, 1H), 2.79 (m, C**H**$_2$CH=CH$_2$, 1H), 5.01 (dd, $^3J_{HH}$ = 7.5 Hz, $^3J_{HH}$ = 5.9 Hz, OC**H**CH$_2$, 1H), 5.11 (m, C**H**=COR, 1H), 5.11 (m, CH=CH**H**, 1H), 5.15 (m, CH=C**H**H, 1H), 5.70 (m, C**H**=CH$_2$, 1H), 7.28 (m, *ortho*-^{Ar}C**H**, 2H), 7.35-7.41 (m, ^{Ar}C**H**, 3H) ppm. **^{19}F NMR** (282 MHz, $CDCl_3$): δ −109.3 (m, C**F**$_2$, 2F), −118.6 (m, C**F**$_2$, 2F) ppm. **^{13}C NMR** (101 MHz, $CDCl_3$): δ 41.5 (s, **C**H$_2$CH=CH$_2$, 1C), 84.8 (s, **C**HO, 1C), 108.4 (tt, $^2J_{FC}$ = 27 Hz, $^3J_{FC}$ = 14 Hz, **C**H=COR, 1C), 117.6 (tt, $^1J_{FC}$ = 274 Hz, $^2J_{FC}$ = 23 Hz, **C**F$_2$, 1C), 117.7 (tt, $^1J_{FC}$ = 287 Hz, $^2J_{FC}$ = 25 Hz, **C**F$_2$, 1C), 119.1 (s, CH=**C**H$_2$, 1C), 126.1 (s, *ortho*-^{Ar}**C**H, 2C), 129.1 (s, *meta*- a *para*-^{Ar}**C**H, 3C), 131.93 (s, **C**H=CH$_2$, 1C), 137.5 (s, *ipso*-^{Ar}**C**, 1C), 158.2 (tt, $^2J_{FC}$ = 24 Hz, $^3J_{FC}$ = 20 Hz, CH=**C**OR, 1C) ppm. **HRMS (EI):** Calculated for $C_{14}H_{12}F_4O$ $[M]^+$ 272.0819, found 272.0815.

1-Methyl-4-{1-[(3,3,4,4-tetrafluorocyclobut-1-en-1-yl)oxy]but-3-en-1-yl}benzene (3c)

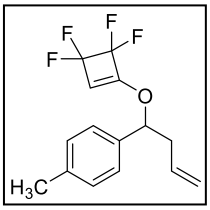

Pentafluorocyclobutene **2c** (0.30 g, 1.0 mmol) was dissolved in dry Et_2O (5 mL) under an inert atmosphere. The solution was cooled to 0 °C and $LiAlH_4$ solution (4M in Et_2O, 0.25 mL, 1.0 mmol) was slowly added. The reaction mixture was stirred at rt overnight and then quenched with water (5 mL). The aqueous phase was separated, washed with Et_2O (2 × 5 mL) and dried over anhydrous Na_2SO_4. The solvent was evaporated on a rotary vacuum evaporator and purified by column chromatography (*n*-hexane/EtOAc, 9:1). Tetrafluorocyclobutene **3b** was obtained as a yellowish liquid (0.10 g, 35%).

^{1}H NMR (400 MHz, $CDCl_3$): δ 2.36 (s, C**H**$_3$, 3H), 2.59 (m, C**H**$_2$, 1H), 2.77 (m, C**H**$_2$, 1H), 4.97 (dd, $^3J_{HH}$ = 7.5 Hz, $^3J_{HH}$ = 6.0 Hz, C**H**O, 1H), 5.07 – 5.16 (m, CF_2C**H** and CH=C**H$_2$**, 3H), 5.70 (m, C**H**=CH_2, 1H), 7.16 (m, ^{Ar}C**H**, 2H), 7.20 (m, ^{Ar}C**H**, 2H) ppm. **^{19}F NMR** (376 MHz, $CDCl_3$): δ (−108.5) - (−109.9) (m, C**F**$_2$, 2F), (−117.8) - (−119.4) (m, C**F**$_2$, 2F) ppm. **^{13}C NMR** (101 MHz, $CDCl_3$): δ 21.2 (s, **C**H$_3$, 1C), 41.4 (s, **C**H$_2$, 1C), 84.8 (s, **C**HO, 1C), 108.3 (tt, $^3J_{FC}$ = 27 Hz, $^2J_{FC}$ = 14 Hz, CF_2**C**H, 1C), 117.7 (tt, $^1J_{FC}$ = 275 Hz, $^2J_{FC}$ = 23 Hz, **C**F$_2$, 1C), 117.8 (tt, $^1J_{FC}$ = 287 Hz, $^2J_{FC}$ = 25 Hz, **C**F$_2$, 1C), 119.0 (s, CH=**C**H$_2$, 1C), 126.1 (s, ^{Ar}**C**H, 2C), 129.6 (s, ^{Ar}**C**H, 2C), 132.1 (s, **C**H=CH_2, 1C), 134.5 (s, ^{Ar}**C**, 1C), 138.9 (s, ^{Ar}**C**CH$_3$, 1C), 158.2 (tt, $^2J_{FC}$ = 24 Hz, $^3J_{FC}$ = 20 Hz, **C**O, 1C) ppm. **HRMS (EI):** Calculated for $C_{15}H_{14}F_4O$ $[M]^+$ 286.0975, found 286.0970.

4-{1-[(3,3,4,4-Tetrafluorocyclobut-1-en-1-yl)oxy]but-3-en-1-yl}-1-trifluoromethylbenzen (3d)

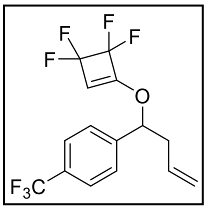

Pentafluorocyclobutene **2d** (1.0 g, 2.8 mmol) was dissolved in dry Et_2O (20 mL) under an inert atmosphere. The solution was cooled to 0 °C and $LiAlH_4$ solution (4M in Et_2O, 4.2 mL, 4.2 mmol) was slowly added. The reaction mixture was stirred at rt overnight and then quenched with water (10 mL). The aqueous phase was separated, washed with Et_2O (2 × 10 mL) and dried over anhydrous Na_2SO_4. The solvent was evaporated on a rotary vacuum evaporator and purified by column chromatography (*n*-hexane/EtOAc, 4:1). Tetrafluorocyclobutene **3d** was obtained as a colourless liquid (0.11 g, 11%).

^{1}H NMR (400 MHz, $CDCl_3$): δ 2.36 (m, C**H**$_2$, 1H), 2.79 (m, C**H**$_2$, 1H), 5.03 – 5.23 (m, CF_2C**H** and C**H**O and CH=C**H$_2$**, 4H), 5.69 (m, C**H=**CH_2, 1H), 7.41 (m, ^{Ar}C**H**, 2H), 7.67 (m, ^{Ar}C**H**, 2H) ppm. **^{19}F NMR** (376 MHz, $CDCl_3$): δ −63.3 (s, C**F**$_3$, 3F), (−108.8) - (−110.2) (m, C**F**$_2$, 2F), (−117.7) - (−119.2) (m, C**F**$_2$, 2F) ppm. **^{13}C NMR** (101 MHz, $CDCl_3$): δ 41.3 (s, **C**H$_2$CH=CH_2, 1C), 83.8 (s, **C**HO, 1C), 108.7 (m, CF_2**C**H, 1C), 114.0 – 121.5 (m, **C**F$_2$, 2C), 119.9 (s, CH=**C**H$_2$, 1C), 123.8 (m, **C**F$_3$, 1C), 126.0 (s, ^{Ar}**C**H, 2C), 126.4 (s, ^{Ar}**C**H, 2C), 131.1 (s, **C**H=CH_2, 1C), 131.2 (q, $^2J_{FC}$ = 23 Hz, CF_3**C**, 1C), 141.4 (s, ^{Ar}**C**, 1C), 158.0 (m, **C**O, 1C) ppm. **HRMS (CI):** Calculated for $C_{15}H_{12}F_7O$ $[M+H]^+$ 341.0771, found 341.0784.

1-Methoxy-3-{1-[(3,3,4,4-tetrafluorocyclobut-1-en-1-yl)oxy]but-3-en-1-yl}benzene (**3e**)

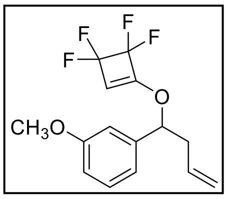

Pentafluorocyclobutene **2e** (0.32 g, 1.0 mmol) was dissolved in dry Et_2O (5 mL) under an inert atmosphere. The solution was cooled to 0 °C and $LiAlH_4$ solution (4M in Et_2O, 0.25 mL, 1.0 mmol) was slowly added. The reaction mixture was stirred at rt overnight and then quenched with water (5 mL). The aqueous phase was separated, washed with Et_2O (2 × 5 mL) and dried over anhydrous Na_2SO_4. The solvent was evaporated on a rotary vacuum evaporator and purified by column chromatography (*n*-hexane/EtOAc, 4:1). Tetrafluorocyclobutene **3e** was obtained as a yellowish liquid (86 mg, 28%).

^{1}H NMR (400 MHz, $CDCl_3$): δ 2.60 (m, C**H**$_2$, 1H), 2.77 (m, C**H**$_2$, 1H), 3.81 (s, C**H**$_3$O, 3H), 4.97 (dd, $^3J_{HH}$ = 7.6 Hz, $^3J_{HH}$ = 5.8 Hz, C**H**O, 1H), 5.09 – 5.20 (m, CF_2C**H** a CH=C**H$_2$**, 3H), 5.71 (m, C**H**=CH_2, 1H), 6.80 (m, ^{Ar}C**H**, 1H), 6.85 (m, ^{Ar}C**H**, 1H), 6.88 (m, ^{Ar}C**H**, 1H), 7.30 (m, ^{Ar}C**H**, 1H) ppm. **^{19}F NMR** (376 MHz, $CDCl_3$): δ (−108.6) - (−109.9) (m, C**F**$_2$, 2F), (−117.9) - (−119.3) (m, C**F**$_2$, 2F) ppm. **^{13}C NMR** (101 MHz, $CDCl_3$): δ 41.4 (s, **C**H_2, 1C), 55.3 (s, **C**H_3O, 1C), 84.7 (s, **C**HO, 1C), 108.4 (m, CF_2**C**H, 1C), 111.4 – 121.0 (m, **C**F_2, 2C), 111.7 (s, ^{Ar}**C**H, 1C), 114.2 (s, ^{Ar}**C**H, 1C), 118.3 (s, ^{Ar}**C**H, 1C), 119.1 (s, CH=**C**H_2, 1C), 130.1 (s, ^{Ar}**C**H, 1C), 132.0 (s, **C**H=CH_2, 1C), 139.1 (s, ^{Ar}**C**, 1C), 158.2 (m, **C**O, 1C), 160.0 (s, ^{Ar}**C**OCH_3, 1C) ppm. **HRMS (EI):** Calculated for $C_{15}H_{14}F_4O_2$ $[M]^+$ 302.0924, found 302.0933.

2-{1-[(3,3,4,4-Tetrafluorocyclobut-1-en-1-yl)oxy]but-3-en-1-yl}pyridine (**3f**)

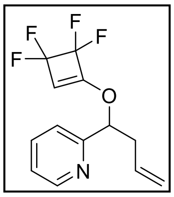

Pentafluorocyclobutene **2f** (0.29 g, 1.0 mmol) was dissolved in dry Et_2O (5 mL) under an inert atmosphere. The solution was cooled to 0 °C and $LiAlH_4$ solution (4M in Et_2O, 0.25 mL, 1.0 mmol) was slowly added. The reaction mixture was stirred at rt overnight and then quenched with water (5 mL). The aqueous phase was separated, washed with Et_2O (2 × 5 mL) and dried over anhydrous Na_2SO_4. The solvent was evaporated on a rotary vacuum evaporator and purified by column chromatography (*n*-hexane/EtOAc, 4:1 → 2:1). Tetrafluorocyclobutene **3f** was obtained as a colourless liquid (0.11 g, 40%).

^{1}H NMR (400 MHz, $CDCl_3$): δ 2.76 (m, C**H**$_2$, 2H), 5.07 – 5.14 (m, CH=C**H$_2$**, 2H), 5.16 (t, $^3J_{HH}$ = 6.5 Hz, C**H**O, 1H), 5.23 (tt, $^3J_{HH}$ = 10.3 Hz, $^3J_{HH}$ = 2.3 Hz, CF_2C**H**, 1H), 5.73 (m, C**H**=CH_2, 1H), 7.26 (m, ^{Ar}C**H**, 1H), 7.32 (m, ^{Ar}C**H**, 1H), 7.74 (m, ^{Ar}C**H**, 1H), 8.56 (m, ^{Ar}C**H**, 1H) ppm. **^{19}F NMR** (376 MHz, $CDCl_3$): δ (−108.9) - (−110.1) (m, C**F**$_2$, 2F), (−117.9) - (−119.3) (m, C**F**$_2$, 2F) ppm. **^{13}C NMR** (101 MHz, $CDCl_3$): δ 39.6 (s, **C**$H_2CH=CH_2$, 1C), 85.1 (s, **C**HO, 1C), 108.8 (tt, $^3J_{FC}$ = 27 Hz, $^2J_{FC}$ = 13 Hz, CF_2**C**H, 1C), 117.4 (tt, $^1J_{FC}$ = 275 Hz, $^2J_{FC}$ = 23 Hz, **C**F_2, 1C), 117.8 (tt, $^1J_{FC}$ = 288 Hz, $^2J_{FC}$ = 25 Hz, **C**F_2, 1C), 119.3 (s, CH=**C**H_2, 1C), 120.3 (s, ^{Ar}**C**H, 1C), 123.6 (s, ^{Ar}**C**H, 1C), 131.6 (s, **C**H=CH_2, 1C), 137.3 (s, ^{Ar}**C**H, 1C), 149.4 (s, ^{Ar}**C**H, 1C), 157.0 (s, ^{Ar}**C**, 1C), 158.3 (m, **C**O, 1C) ppm. **HRMS (ESI):** Calculated for $C_{13}H_{12}F_4NO$ $[M+H]^+$ 274.0850, found 274.0849.

3,3,4,4-Tetrafluoro-1-[(3-methylbut-3-en-1-yl)oxy]cyclobutene (**3g**)

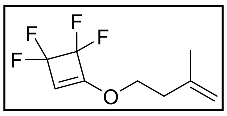

Pentafluorocyclobutene **2g** (0.50 g, 2.2 mmol) was dissolved in dry Et_2O (15 mL) under an inert atmosphere. The solution was cooled to 0 °C and $LiAlH_4$ solution (2M in THF, 1.3 mL, 2.5 mmol) was slowly added. The reaction mixture was stirred at rt for 1 h and then quenched with water (10 mL). The aqueous phase was separated and washed with Et_2O (3 × 10 mL). The combined organic layers were washed with brine (10 mL) and dried over anhydrous Na_2SO_4. The solvent was evaporated on a rotary vacuum evaporator. Tetrafluorocyclobutene **3g** was obtained as a yellowish liquid (0.28 g, 61%).

^{1}H NMR (299,97 MHz, $CDCl_3$): δ 1.77 (s, C**H**$_3$, 3H), 2.48 (t, $^3J_{HH}$ = 6.5 Hz, C**H**$_2$CCH$_3$, 2H), 4.15 (t, $^3J_{HH}$ = 6.5 Hz, OC**H**$_2$, 2H), 4.77 (m, CH=C**H**H, 1H), 4.87 (m, CH=CH**H**, 1H), 5.43 (tt, $^3J_{HF}$ = 10.3 Hz, $^4J_{HF}$ = 2.2 Hz, **H**C=COR, 1H) ppm. **^{19}F NMR** (282 MHz, $CDCl_3$): δ −109.3 (m, C**F**$_2$, 2F), −118.3 (m, C**F**$_2$, 2F) ppm. **^{13}C NMR** (101 MHz, $CDCl_3$): δ 22.5 (s, **C**H$_3$, 1H), 36.1 (s, **C**H$_2$C=CH$_2$, 1C), 70.5 (s, O**C**H$_2$, 1C), 106.7 (tt, $^2J_{FC}$ = 27 Hz, $^3J_{FC}$ = 15 Hz, **C**H=COR, 1C), 113.2 (s, C=**C**H$_2$, 1C), 117.5 (tt, $^1J_{FC}$ = 287 Hz, $^2J_{FC}$ = 25 Hz, **C**F$_2$, 1C), 117.7 (tt, $^1J_{FC}$ = 275 Hz, $^2J_{FC}$ = 23 Hz, **C**F$_2$, 1C), 140.2 (s, **C**=CH$_2$, 1C), 159.3 (tt, $^2J_{FC}$ = 24 Hz, $^3J_{FC}$ = 20 Hz, **C**-OR, 1C) ppm. **HRMS (EI):** Calculated for $C_9H_{10}F_4O$ $[M]^+$ 210.0668, found 210.0664.

1-[(But-3-en-1-yl)oxy]-3,3,4,4-tetrafluoro-2-methylcyclobutene (**15a**)

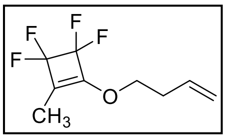

Pentafluorocyclobutene (**2a**, 0.90 g, 4.2 mmol) was dissolved in dry Et_2O (10 mL) under an inert atmosphere. The solution was cooled to 0 °C and CH_3Li solution (1.6M in Et_2O, 2.6 mL, 4.2 mmol) was added dropwise. The reaction mixture was stirred at rt for 1 h and then quenched with water (10 mL). The aqueous phase was separated and washed with Et_2O (3 × 10 mL). The combined organic layers were dried over anhydrous Na_2SO_4, the solvent was evaporated on a rotary vacuum evaporator and the residue was purified by column chromatography (*n*-hexane/DCM, 2:1) Tetrafluorocyclobutene **15a** was obtained as a colourless liquid (0.34 g, 40%).

^{1}H NMR (400 MHz, $CDCl_3$): δ 1.80 (tm, $^4J_{HF}$ = 4.1 Hz, C**H**$_3$, 3H), 2.49 (m, C**H**$_2$, 2H), 4.20 (t, $^3J_{HH}$ = 6.7 Hz, OC**H**$_2$, 2H), 5.13 (dm, $^3J_{HH}$ = 10.3 Hz, CH=CH**H**^{cis}, 1H), 5.16 (dm, $^3J_{HH}$ = 16.8 Hz, CH=CH**H**^{trans}, 1H), 5.80 (m, C**H**=CH$_2$, 1H) ppm. **^{19}F NMR** (376 MHz, $CDCl_3$): δ −115.4 (m, C**F**$_2$, 2F), −116.0 (m, C**F**$_2$, 2F) ppm. **^{13}C NMR** (101 MHz, $CDCl_3$, ^{1}H and ^{19}F decoupling): δ 6.3 (s, **C**H$_3$, 1C), 33.7 (s, **C**H$_2$, 1C), 70.7 (s, O**C**H$_2$, 1C), 117.7 (s, **C**F$_2$, 2C), 118.3 (s, CH=**C**H$_2$, 1C), 119.0 (s, CH$_3$**C**=CO, 1C), 132.7 (s, **C**H=CH$_2$, 1C), 152.6 (s, CH$_3$C=**C**O, 1C) ppm. **HRMS (EI):** Calculated for $C_9H_{10}F_4O$ $[M]^+$ 210.0662, found 210.0656.

{1-[(3,3,4,4-Tetrafluoro-2-methylcyclobut-1-en-1-yl)oxy]but-3-en-1-yl}benzene (**15b**)

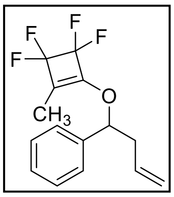

Pentafluorocyclobutene (**2b**, 1.02 g, 3.40 mmol) was dissolved in dry Et_2O (15 mL) under an inert atmosphere. The solution was cooled to 0 °C and CH_3Li solution (1.6M in Et_2O, 2.2 mL, 3.4 mmol) was added dropwise. The reaction mixture was stirred at rt overnight and then quenched with water (10 mL). The aqueous phase was separated and washed with Et_2O (3 × 10 mL). The combined organic layers were dried over anhydrous Na_2SO_4, the solvent was evaporated on a rotary vacuum evaporator and the residue was purified by column chromatography (*n*-hexane). Tetrafluorocyclobutene **15b** was obtained as a colourless liquid (0.45 g, 46%).

^{1}H NMR (400 MHz, $CDCl_3$): δ 1.62 (s, C**H**$_3$, 3H), 2.58 (m, C**H**$_2$, 1H), 2.71 (m, C**H**$_2$, 1H), 5.07-5.21 (m, OC**H** and CH=C**H**$_2$, 3H), 5.72 (m, C**H**=CH$_2$, 1H), 7.26-7.44 (m, ArC**H**, 5H) ppm. **^{19}F NMR** (376 MHz, $CDCl_3$): δ (−115.0)-(−117.0) (m, C**F**$_2$, 4F) ppm. **^{13}C NMR** (101 MHz, $CDCl_3$, 1H and 19F decoupling): δ 6.4 (s, **C**H$_3$, 1C), 42.1 (s, **C**H$_2$, 1C), 83.4 (s, **C**HO, 1C), 117.6 (s, **C**F$_2$, 1C), 118.1 (s, **C**F$_2$, 1C), 119.0 (s, C=**C**H$_2$, 1C), 119.6 (s, CH$_3$**C**=CO, 1C), 125.7 (s, Ar**C**H, 2C), 128.8 (s, Ar**C**H, 1C), 132.3 (s, **C**H=CH$_2$, 1C), 132.3 (s, Ar**C**H, 2C), 138.9 (s, *ipso*-Ar**C**, 1C), 151.9 (m, **C**O, 1C) ppm. **HRMS (CI):** Calculated for $C_{15}H_{15}F_4O$ $[M+H]^+$ 287.1054, found 287.1054.

1-Methyl-4-{1-[(3,3,4,4-tetrafluoro-2-methylcyclobut-1-en-1-yl)oxy]but-3-en-1-yl}benzene (**15c**)

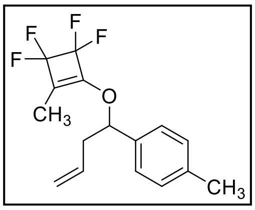

Pentafluorocyclobutene (**2c**, 0.30 g, 1.0 mmol) was dissolved in dry Et_2O (5 mL) under an inert atmosphere. The solution was cooled to 0 °C and CH_3Li solution (1.6M in Et_2O, 0.63 mL, 1.0 mmol) was added dropwise. The reaction mixture was stirred at rt overnight and then quenched with water (10 mL). The aqueous phase was separated and washed with Et_2O (3 × 10 mL). The combined organic layers were dried over anhydrous Na_2SO_4, the solvent was evaporated on a rotary vacuum evaporator and the residue was purified by column chromatography (*n*-hexane). Tetrafluorocyclobutene **15c** was obtained as a colourless liquid (0.18 g, 60%).

^{1}H NMR (400 MHz, $CDCl_3$): δ 1.60 (m, C**H**$_3$C=CO, 3H), 2.35 (s, C**H**$_3$, 3H), 2.55 (m, C**H**$_2$, 1H), 2.70 (m, C**H**$_2$, 1H), 5.05-5.20 (m, OC**H** a CH=C**H**$_2$, 3H), 5.71 (m, C**H**=CH$_2$, 1H), 7.17 (s, ArC**H**, 4H) ppm. **^{19}F NMR** (376 MHz, $CDCl_3$): δ (−115.0)-(−116.9) (m, C**F**$_2$, 4F) ppm. **^{13}C NMR** (101 MHz, $CDCl_3$, ^{1}H and ^{19}F decoupling): δ 6.5 (s, **C**H$_3$C=CO, 1C), 21.3 (s, **C**H$_3$, 1C), 42.1 (s, **C**H$_2$, 1C), 83.5 (s, O**C**H, 1C), 117.7 (s, **C**F$_2$, 1C), 118.3 (s, **C**F$_2$, 1C), 118.8 (s, CH=**C**H$_2$, 1C), 119.6 (s, CH$_3$**C**=CO, 1C), 125.8 (s, Ar**C**H, 2C), 129.5 (s, Ar**C**H, 2C), 132.5 (s, **C**H=CH$_2$, 1C), 136.1 (s, *ipso*-Ar**C**, 1C), 138.6 (s, CH$_3$-**C**Ar, 1C), 152.1 (s, CH$_3$C=**C**O, 1C) ppm. **HRMS (EI):** Calculated for $C_{16}H_{16}F_4O$ $[M]^+$ 300.1132, found 300.1133.

1-{1-[(3,3,4,4-Tetrafluoro-2-methylcyclobut-1-en-1-yl)oxy]but-3-en-1-yl}-4-(trifluoromethyl)benzene (**15d**)

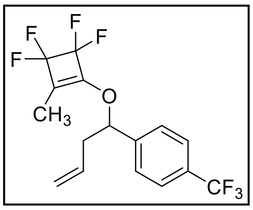

Pentafluorocyclobutene (**2d**, 0.36 g, 1.0 mmol) was dissolved in dry Et_2O (5 mL) under an inert atmosphere. The solution was cooled to 0 °C and CH_3Li solution (1.6M in Et_2O, 0.63 mL, 1.0 mmol) was added dropwise. The reaction mixture was stirred at rt overnight and then quenched with water (5 mL). The aqueous phase was separated and washed with Et_2O (3 × 10 mL). The combined organic layers were dried over anhydrous Na_2SO_4, the solvent was evaporated on a rotary vacuum evaporator and the residue was purified by column chromatography (*n*-hexane → EtOAc). Tetrafluorocyclobutene **15d** was obtained as a colourless liquid (0.15 g, 42%).

^{1}H NMR (400 MHz, $CDCl_3$): δ 1.66 (m, C**H**$_3$C=CO, 3H), 2.58 (m, C**H**$_2$, 1H), 2.72 (m, C**H**$_2$, 1H), 5.23 (m, OC**H**, 1H), 5.06-5.17 (m, CH=C**H**$_2$, 2H), 5.70 (m, C**H**=CH$_2$, 1H), 7.42 (m, ArC**H**, 2H), 7.65 (m, ArC**H**, 2H) ppm. **^{19}F NMR** (376 MHz, $CDCl_3$): δ −63.2 (s, C**F**$_3$, 3F), −115.3 (m, C**F**$_2$, 2F), (−116.2)-(−116.5) (m, C**F**$_2$, 2F) ppm. **^{13}C NMR** (101 MHz, $CDCl_3$, ^{1}H and ^{19}F decoupling): δ 6.4 (s, **C**H$_3$C=CO, 1C), 41.9 (s, **C**H$_2$, 1C), 82.6 (s, **C**HO, 1C), 117.7 (s, **C**F$_2$, 1C), 118.0 (s, **C**F$_2$, 1C), 119.7 (s, CH=**C**H$_2$, 1C), 120.6 (s, CH$_3$**C**=CO, 1C), 124.0 (s, **C**F$_3$, 1C), 125.9 (s, Ar**C**H, 2C), 126.3 (s, Ar**C**H, 2C), 131.0 (s, CF$_3$**C**, 1C), 131.6 (s, **C**H=CH$_2$, 1C), 142.9 (s, *ipso*-Ar**C**, 1C), 151.7 (s, CH$_3$C=**C**O, 1C) ppm. **HRMS (EI):** Calculated for $C_{16}H_{13}F_7O$ $[M]^+$ 354.0849, found 354.0843.

{1-[(2-Butyl-3,3,4,4-tetrafluorocyclobut-1-en-1-yl)oxy]but-3-en-1-yl}benzene (**15e**)

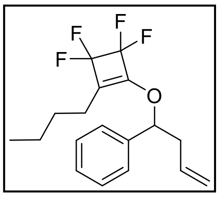

Pentafluorocyclobutene (**2b**, 0.15 g, 0.50 mmol) was dissolved in dry Et_2O (5 mL) under an inert atmosphere. The solution was cooled to -78 °C and *n*-BuLi solution (2.5M in hexanes, 0.20 mL, 0.50 mmol) was added dropwise. The reaction mixture was stirred at rt overnight and then quenched with water (10 mL). The aqueous phase was separated and washed with Et_2O (3 × 10 mL). The combined organic layers were dried over anhydrous Na_2SO_4 and solvents were evaporated on a rotary vacuum evaporator. Tetrafluorocyclobutene **15e** was obtained as a colourless liquid (0.16 g, 96%).

^{1}H NMR (400 MHz, $CDCl_3$): δ 0.86 (t, $^3J_{HH}$ = 7.2 Hz, C**H**$_3$, 3H), 1.27 (m, C**H**$_2$, 2H), 1.43 (m, C**H**$_2$, 2H), 2.07 (m, C**H**$_2$, 2H), 2.57 (m, OCHC**H**$_2$, 1H), 2.57 (m, OCHC**H**$_2$, 1H), 5.08-5.19 (m, OC**H** and CH=C**H**$_2$, 3H), 5.73 (m, C**H**=CH$_2$, 1H), 7.27-7.31 (m, ArC**H**, 2H), 7.32-7.42 (m, ArC**H**, 3H) ppm. **^{19}F NMR** (376 MHz, $CDCl_3$): δ −113.8 (m, C**F**$_2$, 2F), −114.7 (m, C**F**$_2$, 2F) ppm. **^{13}C NMR (**101 MHz, $CDCl_3$, ^{1}H and ^{19}F decoupling): δ 13.7 (s, **C**H$_3$, 1C), 22.2 (s, **C**H$_2$, 1C), 22.6 (s, **C**H$_2$, 1C), 28.9 (s, **C**H$_2$, 1C), 42.2 (s, **C**H$_2$, 1C), 83.6 (s, **C**HO, 1C), 117.9 (s, **C**F$_2$, 1C), 118.5 (s, **C**F$_2$, 1C), 118.9 (s, CH=**C**H$_2$, 1C), 124.6 (s, Bu**C**=CO, 1C), 125.9 (s, Ar**C**H, 2C), 128.7 (s, Ar**C**H, 1C), 128.8 (s, Ar**C**H, 2C), 132.5 (s, **C**H=CH$_2$, 1C), 139.0 (s, *ipso*-Ar**C**, 1C), 151.7 (m, BuC=**C**O, 1C) ppm. **HRMS (EI):** Calculated for $C_{18}H_{20}F_4O$ $[M]^+$ 328.1445, found 328.1448.

{3,3,4,4-Tetrafluoro-2-[(1-phenylbut-3-en-1-yl)oxy]cyclobut-1-en-1-yl)}benzene (**15f**)

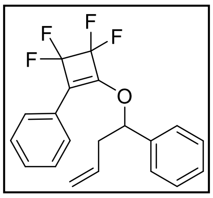

Pentafluorocyclobutene (**2b**, 0.15 g, 0.50 mmol) was dissolved in dry Et_2O (5 mL) under an inert atmosphere. The solution was cooled to -78 °C and PhLi solution (1.8M in Bu_2O, 0.28 mL, 0.50 mmol) was added dropwise. The reaction mixture was stirred at rt overnight and then quenched with water (10 mL). The aqueous phase was separated and washed with Et_2O (3 × 10 mL). The combined organic layers were dried over anhydrous Na_2SO_4, solvents were evaporated on a rotary vacuum evaporator and the residue was purified by column chromatography (*n*-hexane). Tetrafluorocyclobutene **15f** was obtained as a colourless liquid (96 mg, 63%).

^{1}H NMR (400 MHz, $CDCl_3$): δ 2.70 (m, C**H**$_2$, 1H), 2.85 (m, C**H**$_2$, 1H), 5.12- 5.22 (m, CH=C**H**$_2$, 2H), 5.33 (dd, $^3J_{HH}$ = 7.6 Hz, $^3J_{HH}$ = 5.6 Hz, C**H**O, 1H), 5.77 (m, C**H**=CH$_2$, 1H), 7.32-7.48 (m, ArC**H**, 8H), 7.60 (m, ArC**H**, 2H) ppm. **^{19}F NMR** (376 MHz, $CDCl_3$): δ (−109.7)-(−111.0) (m, C**F**$_2$, 2F), (−113.2)-(−114.7) (m, C**F**$_2$, 2F) ppm. **^{13}C NMR** (101 MHz, $CDCl_3$): δ 42.1 (s, **C**H$_2$, 1C), 85.1 (s, O**C**H, 1C), 114.5-121.2 (m, **C**F$_2$, 2C), 119.2 (m, CH=**C**H$_2$, 1C), 125.2 (s, Ar**C**H, 2C), 126.9 (m, Ph**C**=CO, 1C), 127.6 (bs, Ar**C**H, 2C), 128.8 (s, Ar**C**, 1C), 128.8 (s, Ar**C**H, 2C), 128.9 (s, Ar**C**H, 1C), 129.0 (bs, Ar**C**H, 2C), 129.5 (bs, Ar**C**H, 1C), 132.3 (s, **C**H=CH$_2$, 1C), 138.8 (s, Ar**C**, 1C), 150.0 (m, PhC=**C**O, 1C) ppm. **HRMS (EI):** Calculated for $C_{20}H_{16}F_4O$ $[M]^+$ 348.1132, found 348.1122.

5. General procedure for the ROM-RCM reaction sequence

General procedure: A solution of **HGII** precatalyst (16 mg, 24 µmol) in anhydrous, degassed DCM (4 mL) was transferred to a heavy-walled Schlenk flask fitted with a teflon valve. Cyclobutene **3b-f** (0.12 mmol) was added via syringe and then the reaction mixture was heated to 80 °C for two days. Conversion was monitored by ^{19}F NMR analysis using an external standard (DMSO-*d*$_6$). The solvent was then evaporated on a rotary vacuum evaporator and the residue was purified by column chromatography (Ca-silica gel, eluent *n*-hexane/EtOAc, 20:1 or 40:1). NMR yields were determined from the crude reaction mixture using an external standard - a coaxial capillary tube with DMSO-*d*$_6$ inserted into the sample tube. Residual signal of CHD_2SOCD_3 was then used as an integration reference.

CAUTION: Heating dichloromethane to 80-100 °C in a closed vessel generates an internal pressure of up to 6 bar. To prevent the risk of explosion, heavy-walled vessels must be used. The reaction should be performed behind a blast shield in a properly ventilated fume hood.

2-Phenyl-5-(1,1,2,2-tetrafluorobut-3-en-1-yl)-2,3-dihydrofuran (**4b**)

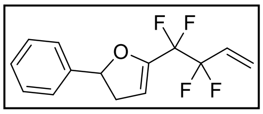

According to the general procedure, dihydrofuran **4b** was synthesized from tetrafluorocyclobutene **3b** as a yellowish liquid (21 mg, isolated yield 64%, NMR yield 84%).

^{1}H NMR (400 MHz, $CDCl_3$): δ 2.76 (m, C**H**$_2$, 1H), 3.24 (m, C**H**$_2$, 1H), 5.48 (m, C**H**, 1H), 5.71 (dd, $^{3}J_{HH}$ = 10.9 Hz, $^{3}J_{HH}$ = 8.6 Hz, OC**H**, 1H), 5.69 (dm, $^{3}J_{HH}$ = 10.8 Hz, CH=CH$^{\mathbf{cis}}$H^{trans}, 1H), 5.89 (dm, $^{3}J_{HH}$ = 17.1 Hz, CH=CH^{cis}**H**$^{\mathbf{trans}}$, 1H), 6.02 (m, C**H**=CH^{cis}H^{trans}, 1H), 7.26 – 7.41 (m, ^{Ar}C**H**, 5H) ppm. **^{19}F NMR** (376 MHz, $CDCl_3$): δ −115.1 (m, C**F**$_2$, 2F), −116.7 (m, C**F**$_2$, 2F) ppm. **^{13}C NMR** (101 MHz, $CDCl_3$): δ 38.5 (s, **C**H$_2$, 1C), 84.3 (s, O**C**H, 1C), 103.7 (m, **C**H=C, 1C), 110.6 – 119.1 (m, **C**F$_2$, 2C), 124.1 (t, $^{3}J_{FC}$ = 9 Hz, CH=**C**H$_2$, 1C), 125.5 (s, ^{Ar}**C**H, 2C), 126.5 (t, $^{2}J_{FC}$ = 24 Hz, **C**H=CH$_2$, 1C), 128.1 (s, ^{Ar}**C**H, 1C), 128.7 (s, ^{Ar}**C**H, 2C), 141.7 (s, ^{Ar}**C**, 1C), 146.9 (t, $^{2}J_{FC}$ = 40 Hz, CH=**C**, 1C) ppm. **HRMS (EI):** Calculated for $C_{14}H_{12}F_4O$ $[M]^+$ 272.0819, found 272.0822.

2-(4-Methylphenyl)-5-(1,1,2,2-tetrafluorobut-3-en-1-yl)-2,3-dihydrofuran (**4c**)

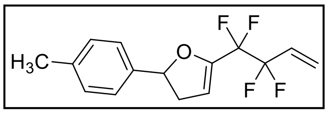

According to the general procedure, dihydrofuran **4c** was synthesized from tetrafluorocyclobutene **3c** as a yellowish liquid (18 mg, isolated yield 53%, NMR yield 93%).

^{1}H NMR (400 MHz, $CDCl_3$): δ 2.35 (s, C**H**$_3$, 3H), 2.76 (m, C**H**$_2$, 1H), 3.21 (m, C**H**$_2$, 1H), 5.48 (m, C=C**H**, 1H**)**, 5.68 (dd, $^{3}J_{HH}$ = 10.6 Hz, $^{3}J_{HH}$ = 8.5 Hz, OC**H**, 1H), 5.68 (dm, $^{3}J_{HH}$ = 10.9 Hz, CH=C**H**^{cis}H^{trans}, 1H), 5.89 (dm, $^{3}J_{HH}$ = 8.5 Hz, CH=CH$^{\mathbf{cis}}$**H**^{trans}, 1H), 6.02 (m, C**H**=CH^{cis}H^{trans}, 1H), 7.18 (m, ^{Ar}C**H**, 2H), 7.25 (m, ^{Ar}C**H**, 2H) ppm. **^{19}F NMR** (376 MHz, $CDCl_3$): δ −115.2 (m, C**F**$_2$, 2F), (−115.9) - (−117.6) (m, C**F**$_2$, 2F) ppm. **^{13}C NMR** (101 MHz, $CDCl_3$): δ 21.2 (s, **C**H$_3$, 1C), 38.4 (s, **C**H$_2$, 1C), 84.3 (s, O**C**H, 1C), 103.7 (tm, $^{3}J_{FC}$ = 5 Hz, **C**H=C, 1C), 111.7 (tt, $^{1}J_{FC}$ = 250 Hz, $^{2}J_{FC}$ = 35 Hz, **C**F$_2$, 1C), 114.6 (tt, $^{1}J_{FC}$ = 251 Hz, $^{2}J_{FC}$ = 33 Hz, **C**F$_2$, 1C), 124.1 (t, $^{3}J_{FC}$ = 10 Hz, CH=**C**H$_2$, 1C), 125.6 (s, ^{Ar}**C**H, 2C), 126.5 (t, $^{2}J_{FC}$ = 24 Hz, **C**H=CH$_2$, 1C), 129.3 (s, ^{Ar}**C**H, 2C), 137.9 (s, CH$_3$**C**, 1C), 138.7 (s, ^{Ar}**C**, 1C), 146.9 (t, $^{2}J_{FC}$ = 30 Hz, O**C**CF$_2$, 1C) ppm. **HRMS (EI):** Calculated for $C_{15}H_{14}F_4O$ $[M]^+$ 286.0975, found 286.0982.

5-(1,1,2,2-Tetrafluorobut-3-en-1-yl)-2-(4-(trifluoromethyl)phenyl)-2,3-dihydrofuran (**4d**)

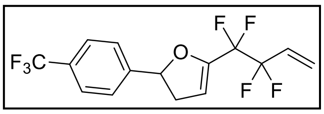

According to the general procedure, dihydrofuran **4d** was synthesized from tetrafluorocyclobutene **3d** as a yellowish liquid (15 mg, isolated yield 37%, NMR yield 99%).

^{1}H NMR (400 MHz, $CDCl_3$): δ 2.73 (m, C**H**$_2$, 1H), 3.31 (m, C**H**$_2$, 1H), 5.50 (m, C**H**, 1H), 5.78 (dd, $^{3}J_{HH}$ = 10.0 Hz, $^{3}J_{HH}$ = 8.1 Hz, OC**H**, 1H), 5.72 (dm, $^{3}J_{HH}$ = 10.9 Hz, CH=C**H**$^{\mathbf{cis}}$H^{trans}, 1H), 5.72 (dm, $^{3}J_{HH}$ = 17.3 Hz, CH=CH^{cis}**H**$^{\mathbf{trans}}$, 1H), 5.97 – 6.14 (m, C**H**=CH^{cis}H^{trans}, 1H), 7.46 (m, ^{Ar}C**H**, 2H), 7.64 (m, ^{Ar}C**H**, 2H) ppm. **^{19}F NMR** (376 MHz, $CDCl_3$): δ −63.1 (s, C**F**$_3$, 3F), −114.8 (m, C**F**$_2$, 2F), −116.4 (m, C**F**$_2$, 2F) ppm. **^{13}C NMR** (101 MHz, $CDCl_3$): δ 38.5 (s, **C**H$_2$, 1C), 83.2 (s, O**C**H, 1C), 103.7 (t, $^{3}J_{FC}$ = 5 Hz, **C**H=C, 1C), 111.7 (tt, $^{1}J_{FC}$ =

249 Hz, $^{2}J_{FC}$ = 37 Hz, **C**F_2, 1C), 114.6 (tt, $^{1}J_{FC}$ = 249 Hz, $^{2}J_{FC}$ = 33 Hz, **C**F_2, 1C), 124.0 (q, $^{3}J_{FC}$ = 272 Hz, **C**F_3, 1C), 124.3 (t, $^{3}J_{FC}$ = 9 Hz, CH=**C**H_2, 1C), 125.6 (s, ^{Ar}**C**H, 2C), 125.7 (t, $^{4}J_{FC}$ = 4 Hz, ^{Ar}**C**H, 2C), 126.3 (t, $^{2}J_{FC}$ = 24 Hz, **C**H=CH_2, 1C), 130.2 (q, $^{3}J_{FC}$ = 33 Hz, CF_3**C**, 1C), 145.8 (s, ^{Ar}**C**, 1C), 147.0 (t, $^{2}J_{FC}$ = 31 Hz, O**C**CF_2, 1C) ppm. **HRMS (CI):** Calculated for $C_{15}H_{12}F_7O$ $[M+H]^+$ 341.0771, found 341.0770.

2-(3-Methoxyphenyl)-5-(1,1,2,2-tetrafluorobut-3-en-1-yl)-2,3-dihydrofuran (**4e**)

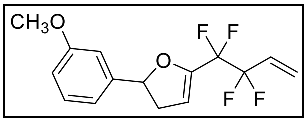

According to the general procedure, dihydrofuran **4e** was synthesized from tetrafluorocyclobutene **3e** as a yellowish liquid (9 mg, isolated yield 25%, NMR yield 84%).

^{1}H NMR (400 MHz, $CDCl_3$): δ 2.74 (m, C**H**$_2$, 1H), 3.23 (m, C**H**$_2$, 1H), 3.80 (s, C**H**$_3$O, 3H), 5.47 (m, C=C**H**, 1H), 5.65 – 5.72 (m, OC**H** a CH=C**H**^{cis}H^{trans}, 2H), 5.90 (dm, $^{3}J_{HH}$ = 17.3 Hz, CH=CH^{cis}**H**^{trans}, 1H), 6.01 (m, C**H**=CH^{cis}H^{trans}, 1H), 6.88 – 6.92 (m, ^{Ar}C**H**, 2H), 6.84 (m, ^{Ar}C**H**, 1H), 7.26 (m, ^{Ar}C**H**, 1H) ppm. **^{19}F NMR** (376 MHz, $CDCl_3$): δ −115.0 (m, C**F**$_2$, 2F), −116.6 (m, C**F**$_2$, 2F) ppm. **^{13}C NMR** (101 MHz, $CDCl_3$): δ 38.5 (s, **C**H_2, 1C), 55.2 (s, **C**H_3O, 1C), 84.0 (s, O**C**H, 1C), 103.7 (m, **C**H=C, 1C), 111.0-119.0 (m, **C**F_2, 2C), 110.7 (s, ^{Ar}**C**H, 1C), 113.6 (s, ^{Ar}**C**H. 1C), 117.6 (s, ^{Ar}**C**H. 1C), 124.1 (t, $^{3}J_{FC}$ = 9 Hz, CH=**C**H_2, 1C), 126.5 (t, $^{2}J_{FC}$ = 24 Hz, **C**H=CH_2, 1C), 129.8 (s, ^{Ar}**C**H, 1C), 143.5 (s, ^{Ar}**C**, 1C), 146.9 (m, O**C**CF_2, 1C), 159.9 (s, ^{Ar}**C**OCH_3, 1C) ppm. **HRMS (EI):** Calculated for $C_{15}H_{14}F_4O_2$ $[M]^+$ 302.0924, found 302.0931.

Mixture of 2-phenyl-6-(1,1,2,2-tetrafluorobut-3-en-1-yl)-3,4-dihydro-2*H*-pyran (**13b**) and 2-phenyl-5-(1,1,2,2-tetrafluoropent-3-en-1-yl)-2,3-dihydrofuran (**14b**)

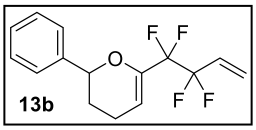

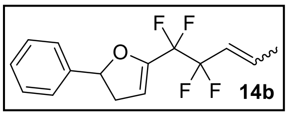

According to the general procedure, 7 mg of the complex mixture of starting tetrafluorocyclobutene **12b**, dihydropyran **13b** and dihydrofuran **14b** in ca. 3:3:4 ratio was obtained after purification by column chromatography (eluent *n*-hexane/EtOAc 100:0 → 95:5) from 15 mg of starting cyclobutene **12b**. Although signals of vinylic hydrogens and CH_2 groups could be recognized in the ^{1}H NMR spectrum, the spectrum was too complex to enable full correct assignment. The composition of the crude product was hence estimated based on ^{19}F NMR spectrum.

13b: **^{19}F NMR** (376 MHz, $CDCl_3$): δ −111.8 (m, C**F**$_2$, 2F), -115.8 (dm, $^{3}J_{HF}$ = 38.2 Hz, C**F**$_2$, 2F).

14b: **^{19}F NMR** (376 MHz, $CDCl_3$): δ −114.5 (dm, $^{3}J_{HF}$ = 11.0 Hz, C**F**$_2$, 2F), -116.1 (dm, $^{3}J_{HF}$ = 21.6 Hz, C**F**$_2$, 2F). **HRMS (ESI):** Calculated for $C_{15}H_{15}F_4O$ $[M+H]^+$ 287.1054, found 287.1056; calcd. for $C_{15}H_{14}F_4NaO$ $[M+Na]^+$ 309.0873, found 309.0875.

2-Phenyl-5-(1,1,2,2-tetrafluoro-3-methylbut-3-en-1-yl)-2,3-dihydrofuran (**16b**)

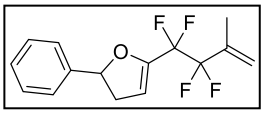

According to the general procedure, a complex mixture containing starting tetrafluorocyclobutene **15b** and dihydrofuran **16b** in ca. 1:2 ratio was obtained after purification by column chromatography (eluent *n*-hexane/EtOAc 98:2) from 34 mg of starting cyclobutene **15b**. Although signals of vinylic hydrogens and CH_2 groups could be

recognized in the ^{1}H NMR spectrum, the spectrum was too complex to enable full correct assignment. The composition of the crude product was hence estimated based on ^{19}F NMR spectrum.

^{19}F NMR (376 MHz, $CDCl_3$): δ −113.8 (m, C**F**$_2$, 2F), -114.5 (m, C**F**$_2$, 2F). **HRMS (CI):** Calculated for $C_{15}H_{15}F_4O$ $[M+H]^+$ 287.1054, found 287.1048.

2-(4-Methylphenyl)-5-(1,1,2,2-tetrafluoro-3-methylbut-3-en-1-yl)- 2,3-dihydrofuran (**16c**)

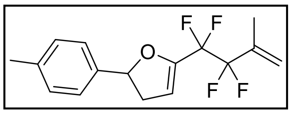

According to the general procedure, a complex mixture containing starting tetrafluorocyclobutene **15c** and dihydrofuran **16c** in ca. 7:3 ratio was obtained after purification by column chromatography (eluent *n*-hexane/EtOAc 98:2) from 36 mg of starting cyclobutene **15c**. Although signals of vinylic hydrogens and CH_2 groups could be recognized in the ^{1}H NMR spectrum, the spectrum was too complex to enable full correct assignment. The composition of the crude product was hence estimated based on ^{19}F NMR spectrum.

^{19}F NMR (376 MHz, $CDCl_3$): δ −113.9 (m, C**F**$_2$, 2F), -114.7 (m, C**F**$_2$, 2F).

5-(1,1,2,2-Tetrafluoro-3-methylbut-3-en-1-yl)-2-[4-(trifluoromethyl)phenyl]-2,3-dihydrofuran (**16d**)

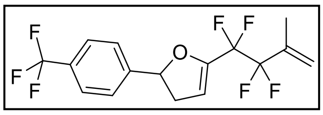

According to the general procedure, a complex mixture containing starting tetrafluorocyclobutene **15d** and dihydrofuran **16d** in ca. 2:1 ratio was obtained after purification by column chromatography (eluent hexane/EtOAc 98:2) from 43 mg of starting cyclobutene **15d**. Although signals of vinylic hydrogens and CH_2 groups could be recognized in the ^{1}H NMR spectrum, the spectrum was too complex to enable full correct assignment. The composition of the crude product was hence estimated based on ^{19}F NMR spectrum.

^{19}F NMR (376 MHz, $CDCl_3$): δ -62.6 (s, C**F**$_3$, 3F), −113.7 (m, C**F**$_2$, 2F), -114.6 (m, C**F**$_2$, 2F).

6. References:

[S1] Kučnirová, K.; Kvíčala, J.; Svoboda, M.; Cvačka, J.; Čejka, J.; Rybáčková, M. Non-Symmetrical Tetrafluoroalkadienes Synthesized by ROCM of 3,3,4,4-Tetrafluorocyclobutene. *Chem. Eur. J.* **2023**, *29*, e202300435.

[S2] Staniland, S.; Adams, R. W.; McDouall, J. J. W.; Maffucci, I.; Contini, A.; Grainger, D. M.; Turner, N. J.; Clayden, J. Biocatalytic Dynamic Kinetic Resolution for the Synthesis of Atropisomeric Biaryl *N*-Oxide Lewis Base Catalysts. *Angew. Chem., Int. Ed.* **2016**, *55*, 10755.

[S3] Masuda, R.; Yasukawa, T.; Yamashita, Y.; Kobayashi, S. Nitrogen-Doped Carbon-Incarcerated Zinc Electrodes as Heterogeneous Catalysts for Electrochemical Allylation of Carbonyl Compounds. *J. Org. Chem.* **2022**, *87*, 3453.

[S4] Dey, P.; Koli, M.; Goswani, D.; Sharma, A.; Chattopadhyay, S. [bmim][Br] as an Inexpensive and Efficient Medium for the Barbier-Type Allylation Reaction Using a Catalytic Amount of Indium: Mechanistic Studies. *Eur. J. Org. Chem.* **2018**, 1333.

[S5] Hamaguchi, T.; Takahashi, Y.; Tsuji, H.; Kawatsura, M. Nickel-Catalyzed Hydroarylation of in Situ Generated 1,3-Dienes with Arylboronic Acids Using a Secondary Homoallyl Carbonate as a Surrogate for the 1,3-Diene and Hydride Source. *Org. Lett.* **2020**, *22*, 1124.

[S6] Timperley, C. M. Fluoroalkene chemistry: Part 2. Reactions of thiols with some toxic 1,2-dichlorinated polyfluorocycloalkenes. *J. Fluorine Chem.* **2004**, *125*, 685.

7. Spectral data:

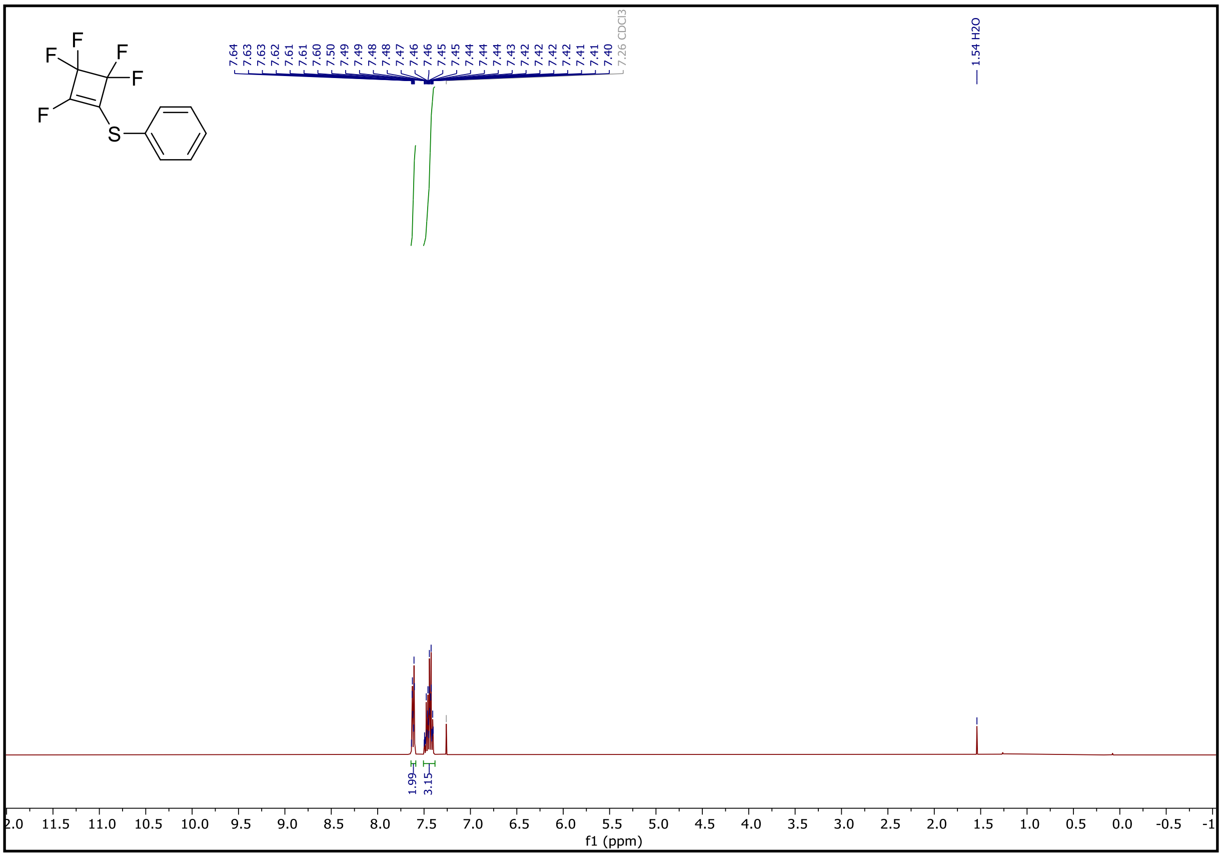

Figure S1: ^{1}H NMR spectrum of pentafluorocyclobutene **5a** ($CDCl_3$, 400 MHz)

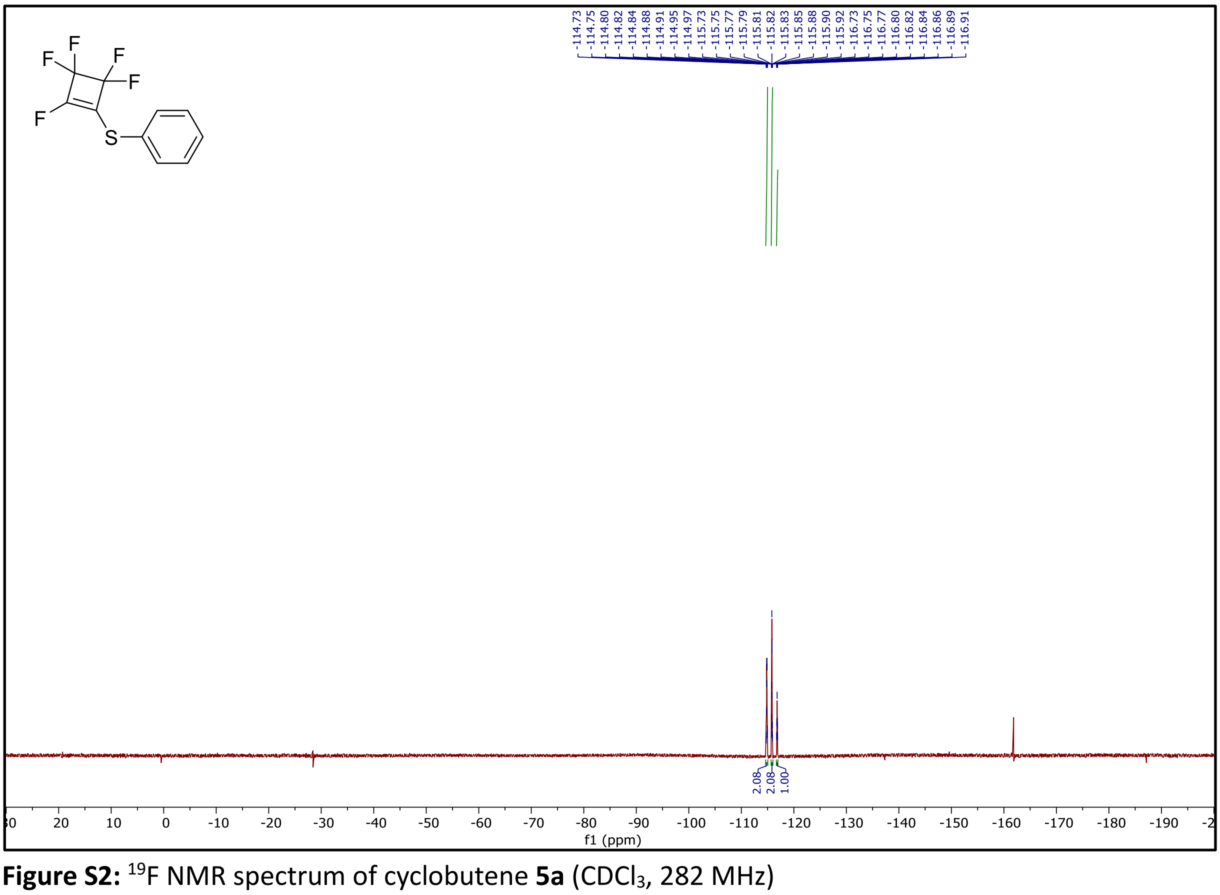

Figure S2: ^{19}F NMR spectrum of cyclobutene **5a** ($CDCl_3$, 282 MHz)

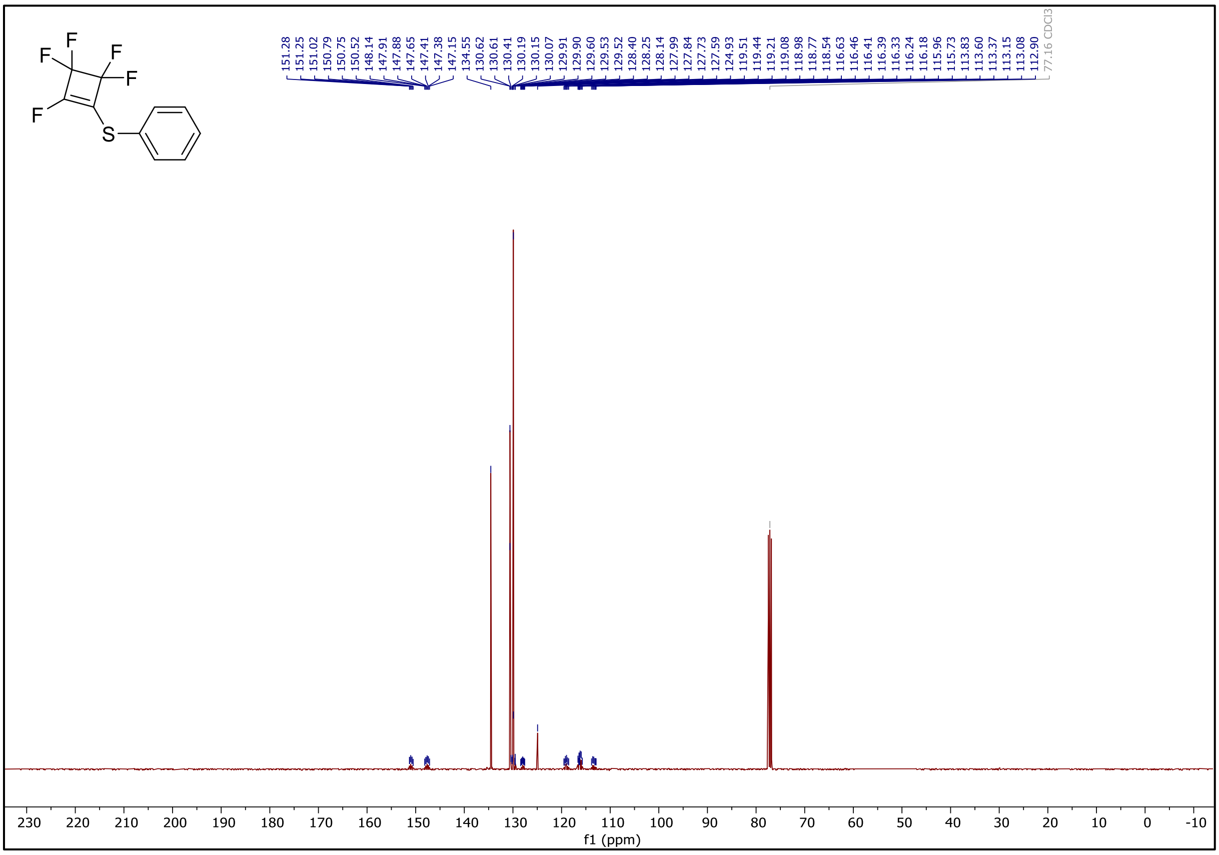

Figure S3: ^{13}C NMR spectrum of cyclobutene **5a** ($CDCl_3$, 101 MHz)

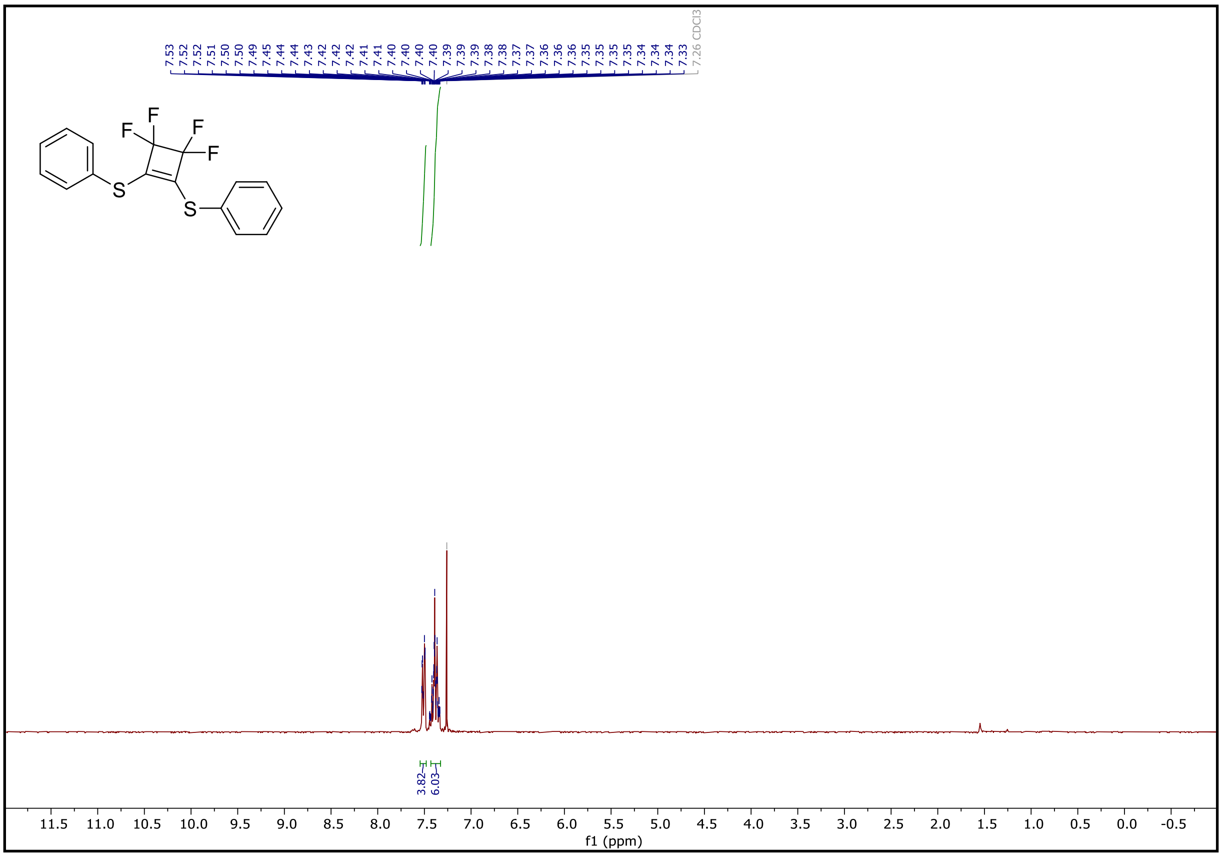

Figure S4: ^{1}H NMR of tetrafluorocyclobutene **6a** ($CDCl_3$, 300 MHz)

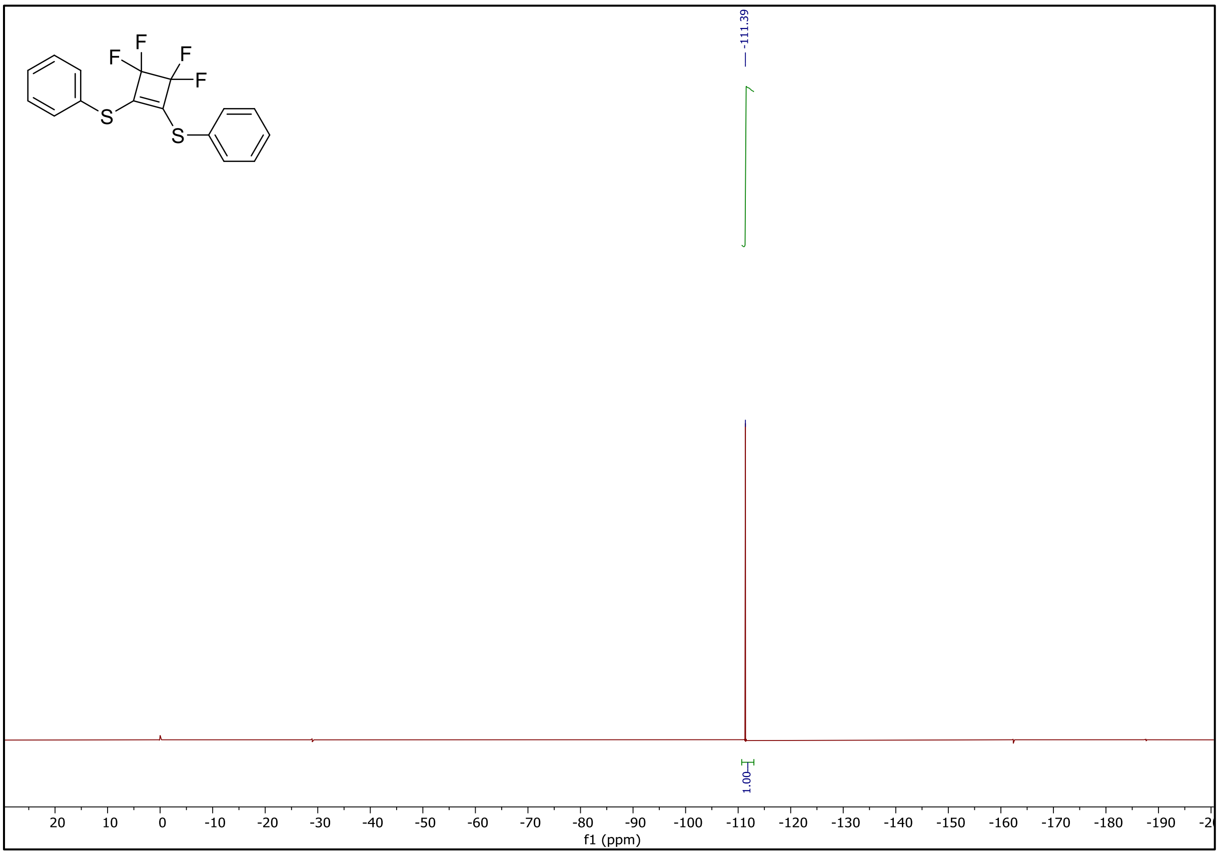

Figure S5: ^{19}F NMR spectrum of tetrafluorocyclobutene **6a** ($CDCl_3$, 282 MHz)

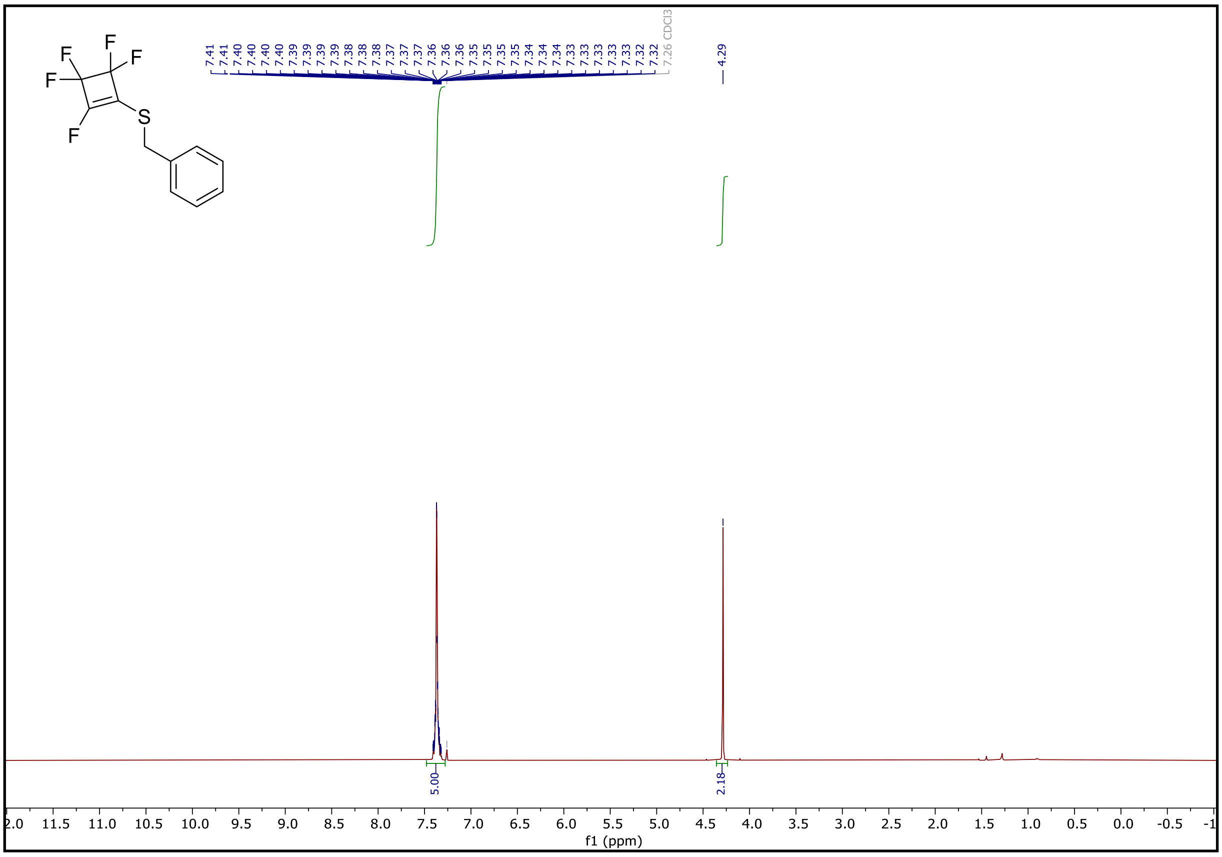

Figure S6: ^{1}H NMR spectrum of pentafluorocyclobutene **5b** ($CDCl_3$, 400 MHz)

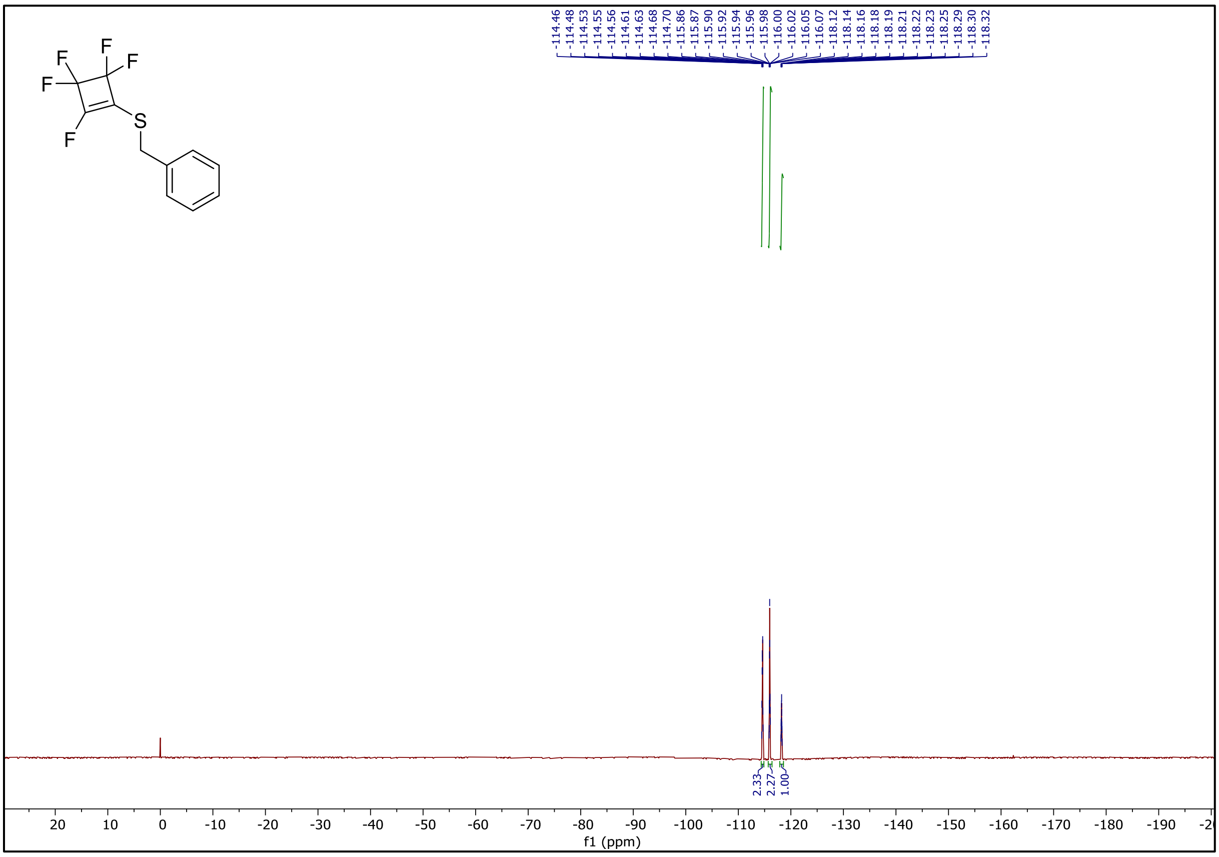

Figure S7: ^{19}F NMR spectrum of pentafluorocyclobutene **5b** ($CDCl_3$, 282 MHz)

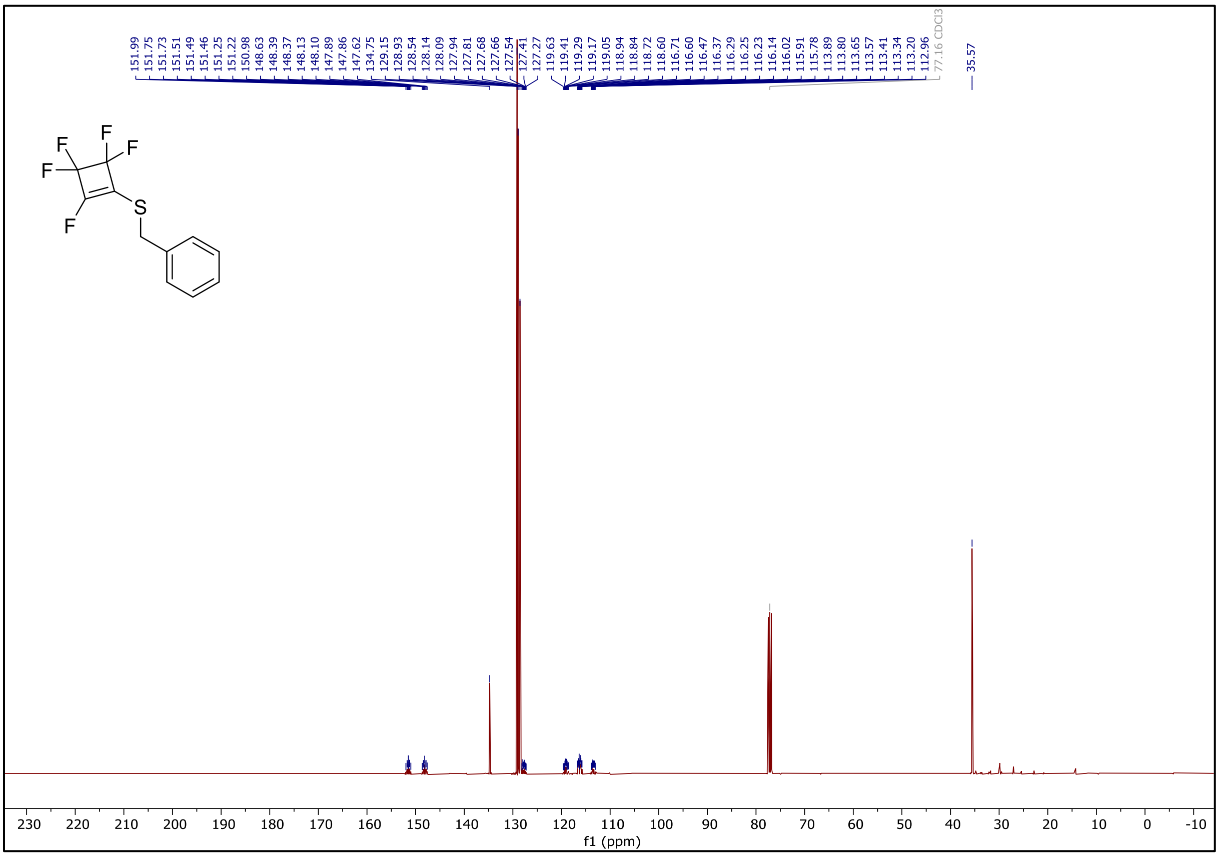

Figure S8: ^{13}C NMR spectrum of pentafluorocyclobutene **5b** ($CDCl_3$, 101 MHz)

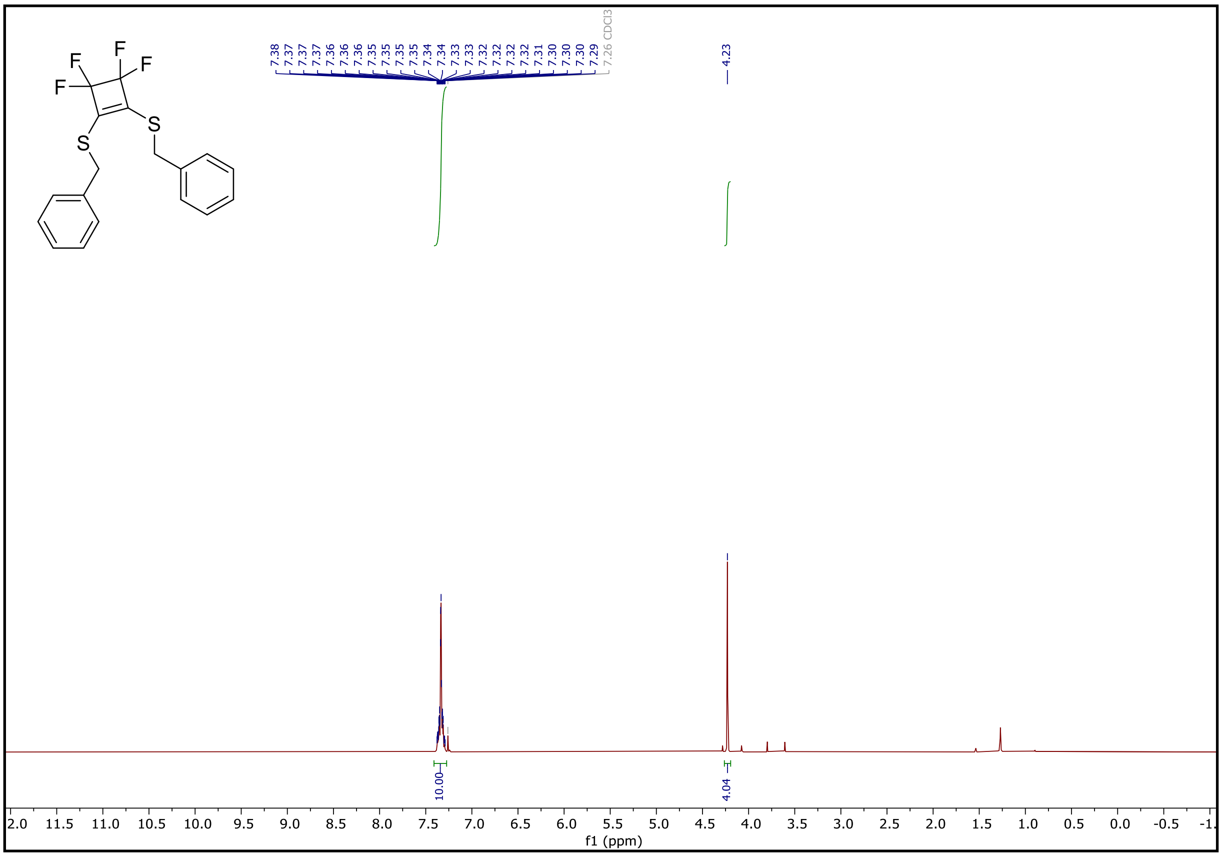

Figure S9: ^{1}H NMR spectrum of tetrafluorocyclobutene **6b** ($CDCl_3$, 400 MHz)

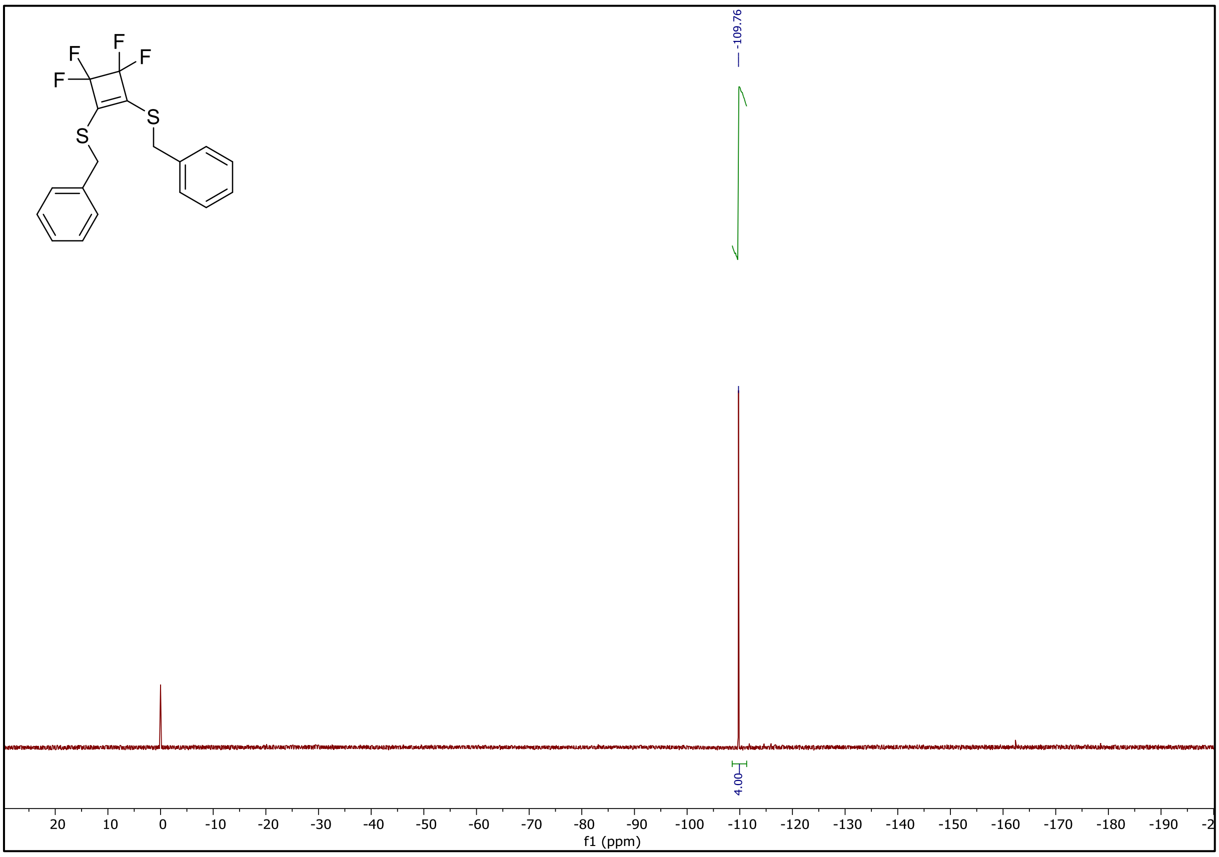

Figure S10: ^{19}F NMR spectrum of tetrafluorocyclobutene **6b** ($CDCl_3$, 282 MHz)

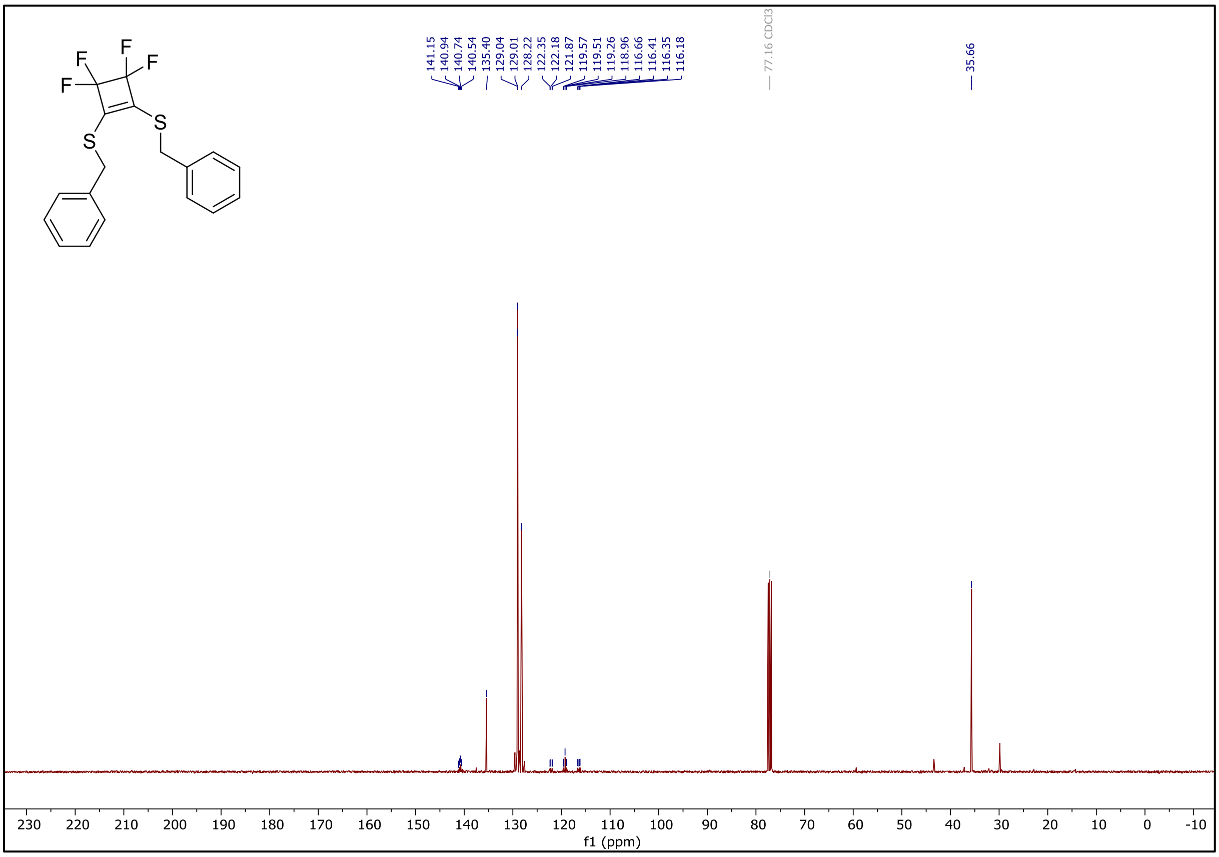

Figure S11: ^{13}C NMR spectrum of tetrafluorocyclobutene **6b** ($CDCl_3$, 101 MHz)

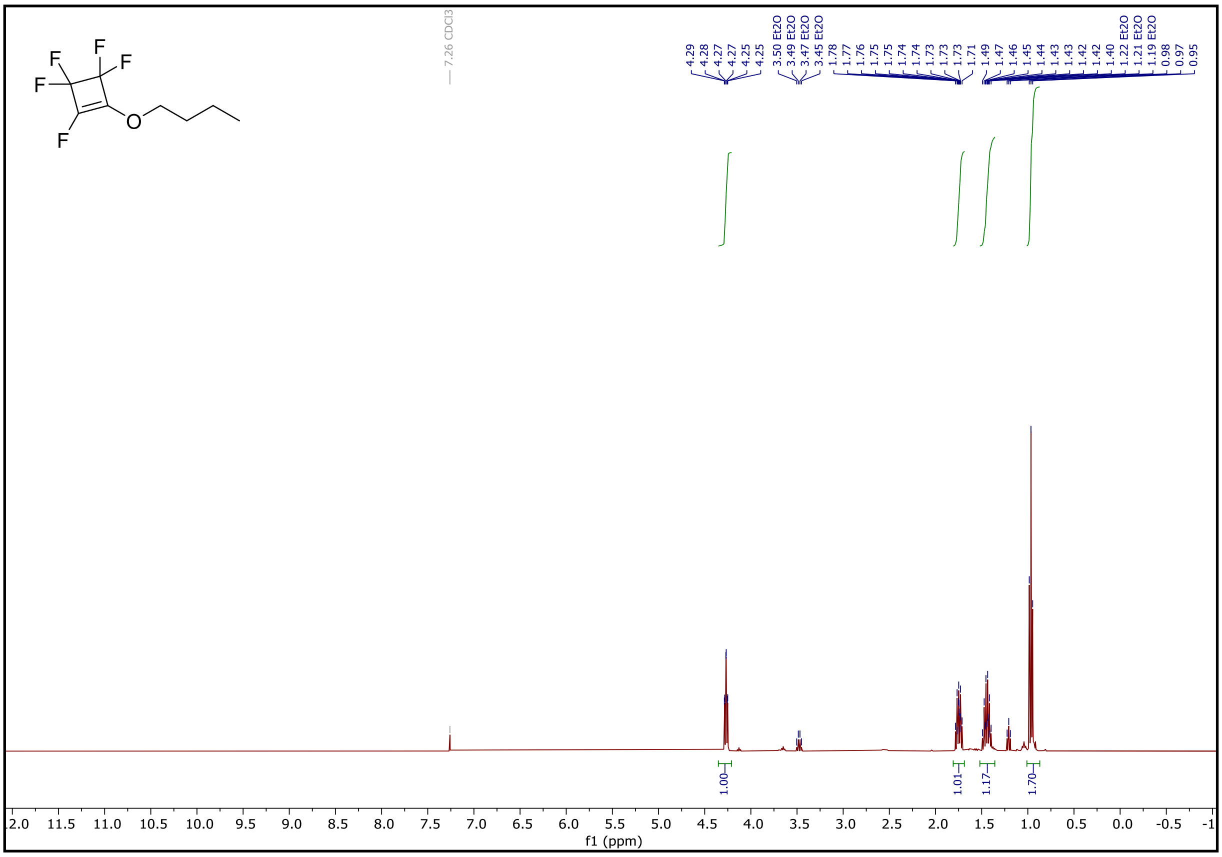

Figure S12: ^{1}H NMR spectrum of pentafluorocyclobutene **7a** ($CDCl_3$, 400 MHz)

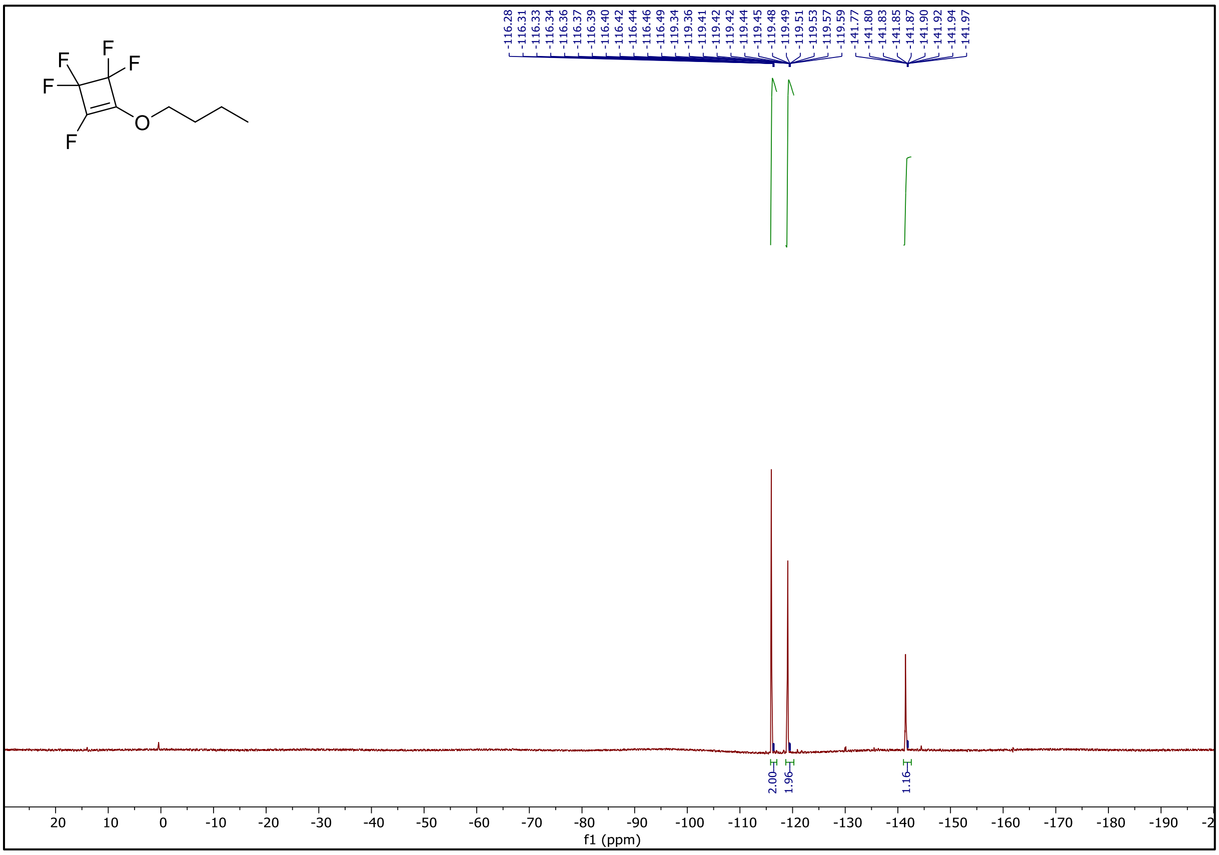

Figure S13: ^{19}F NMR spectrum of pentafluorocyclobutene **7a** ($CDCl_3$, 282 MHz)

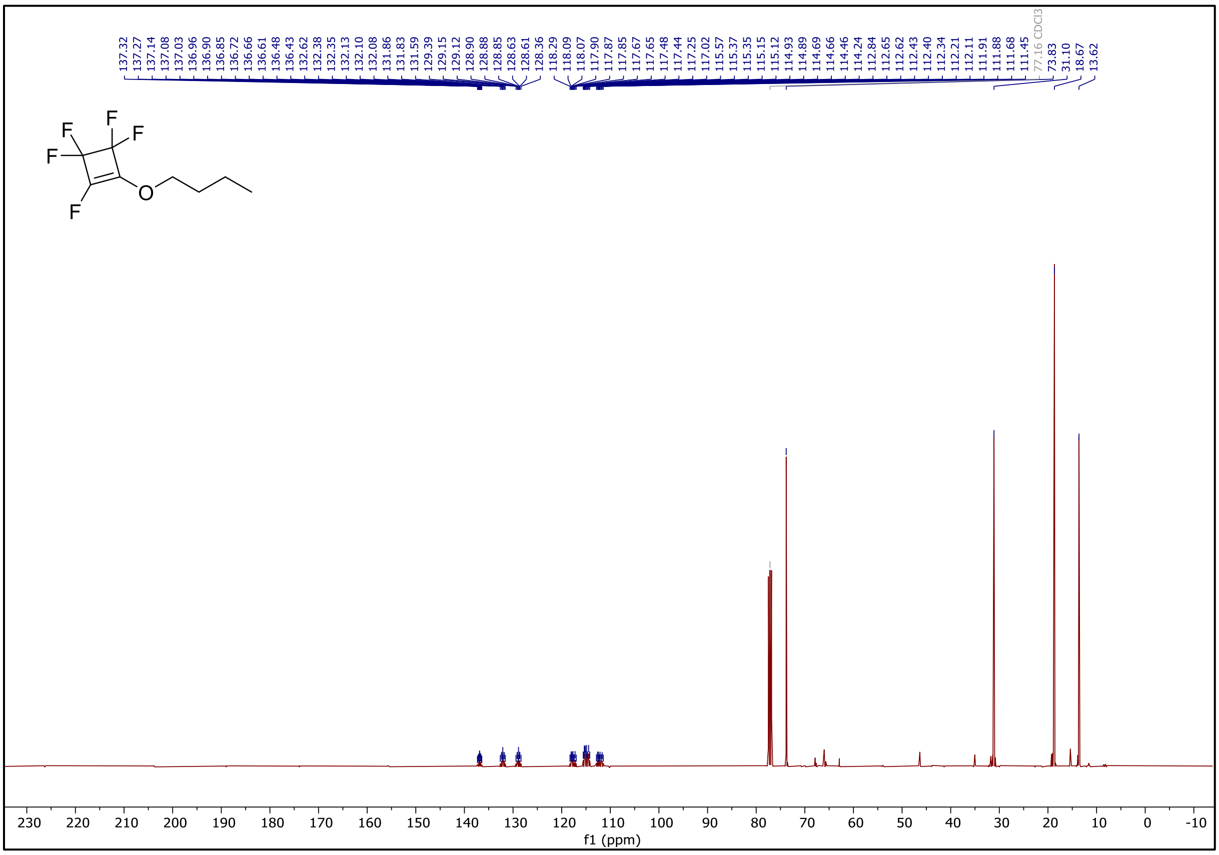

Figure S14: ^{13}C NMR spectrum of pentafluorocyclobutene **7a** ($CDCl_3$, 101 MHz)

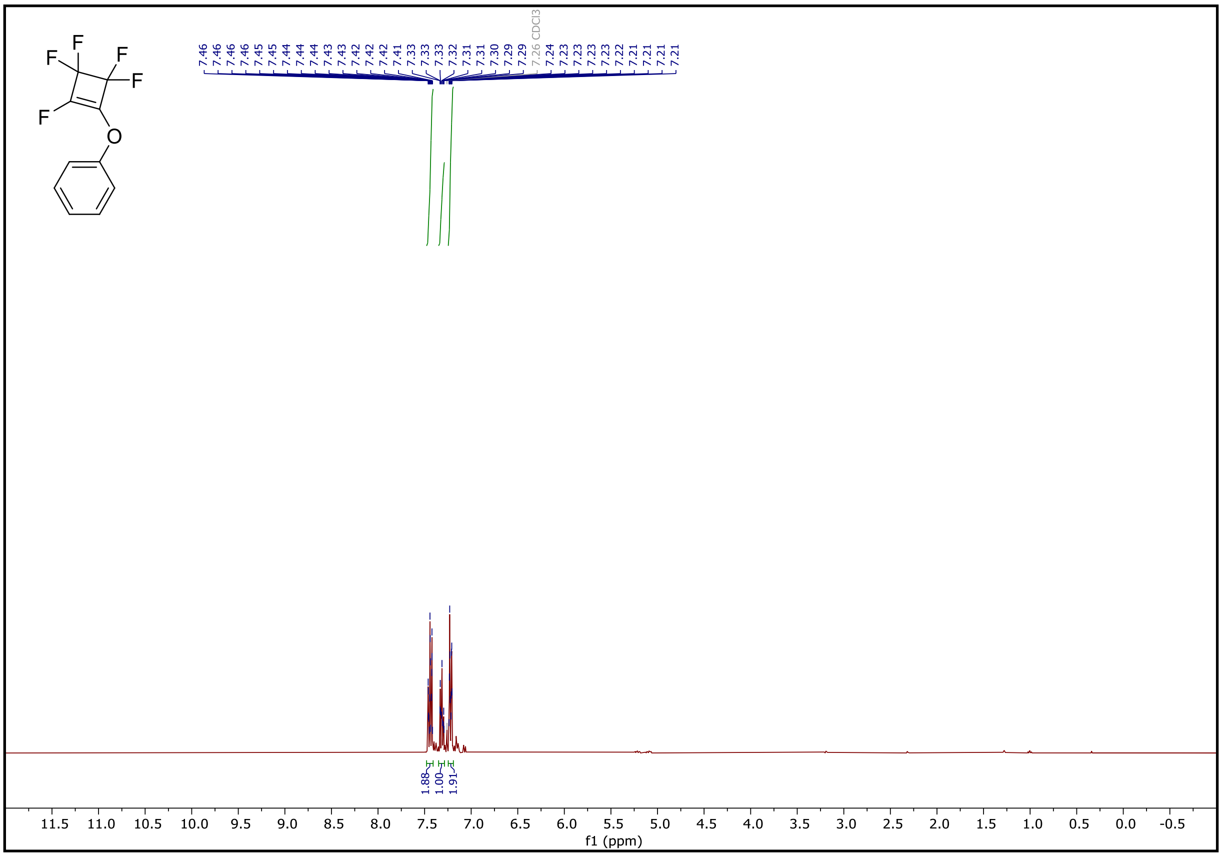

Figure S15: ^{1}H NMR spectrum of pentafluorocyclobutene **7b** ($CDCl_3$, 300 MHz)

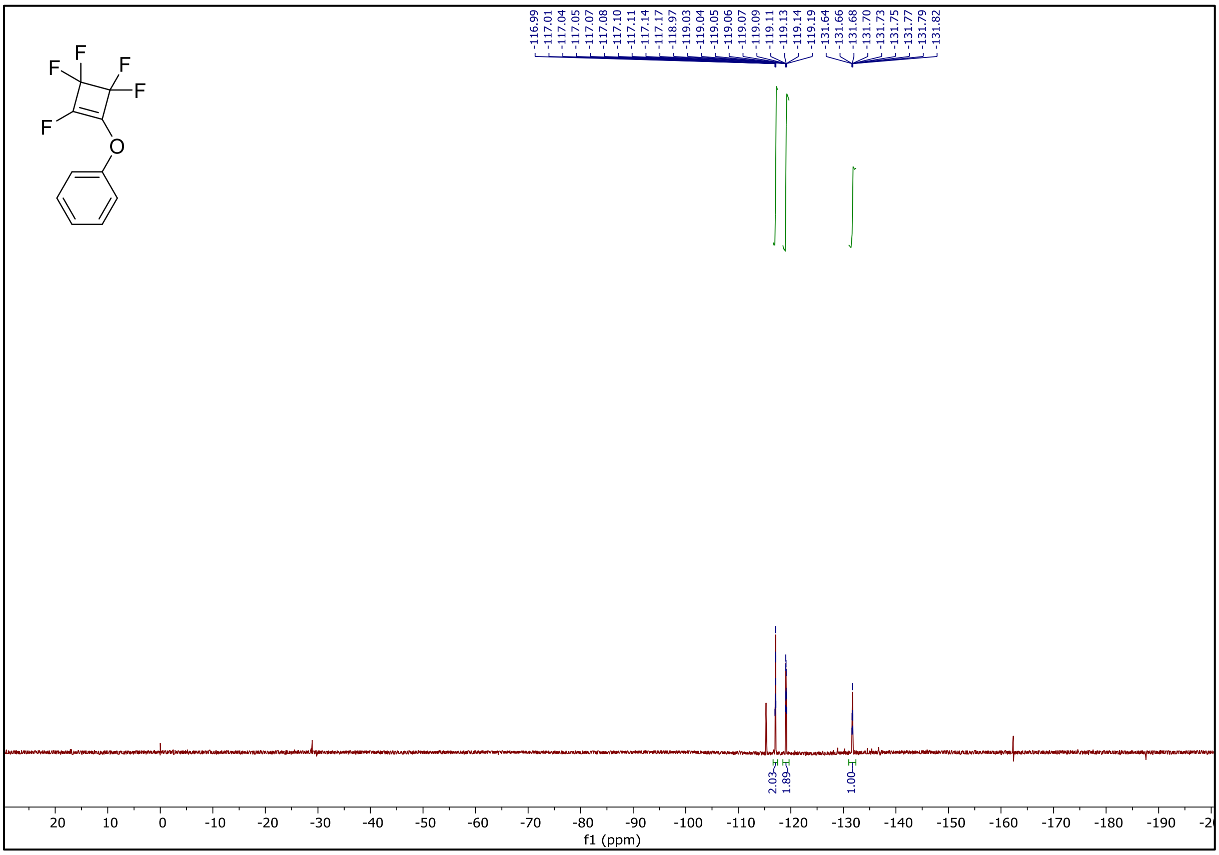

Figure S16: ^{19}F NMR spectrum of pentafluorocyclobutene **7b** ($CDCl_3$, 282 MHz)

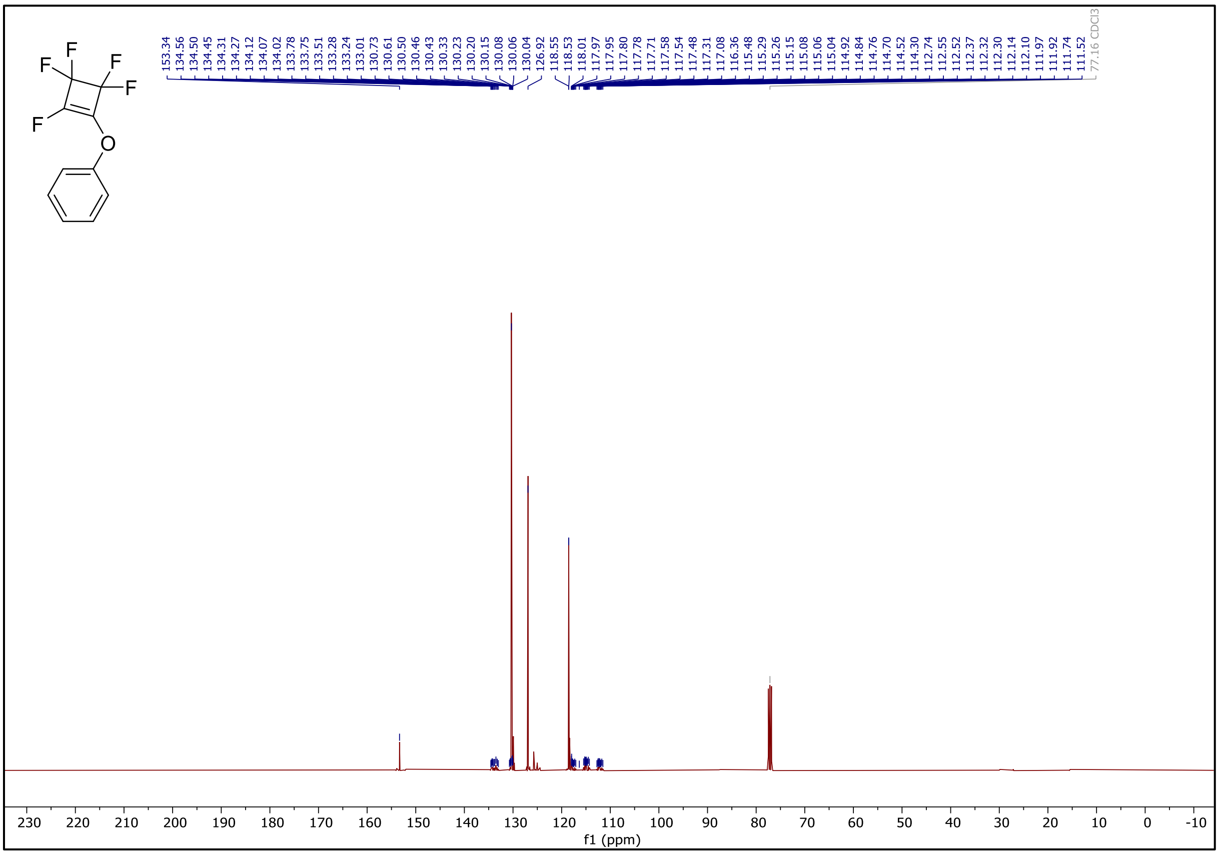

Figure S17: ^{13}C NMR spectrum of pentafluorocyclobutene **7b** ($CDCl_3$, 101 MHz)

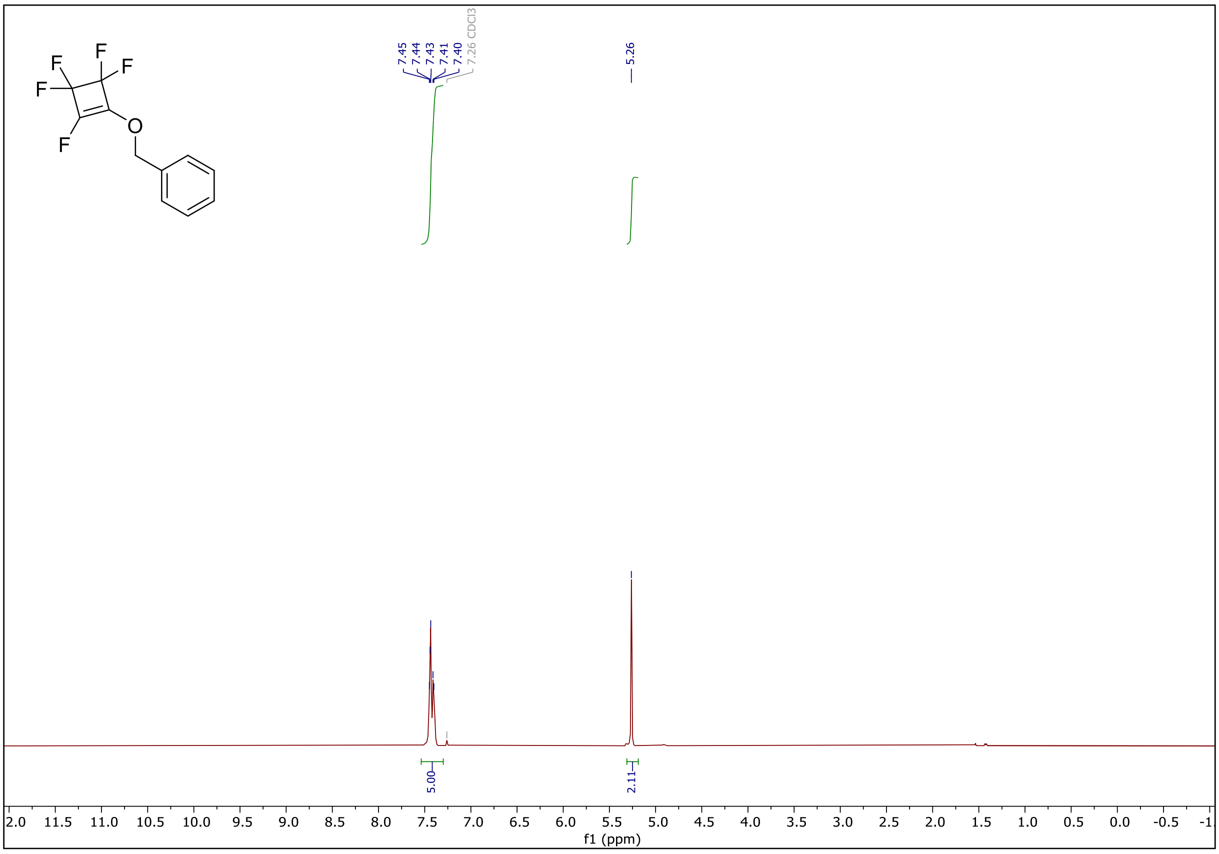

Figure S18: ^{1}H NMR spectrum of pentafluorocyclobutene **7c** ($CDCl_3$, 300 MHz)

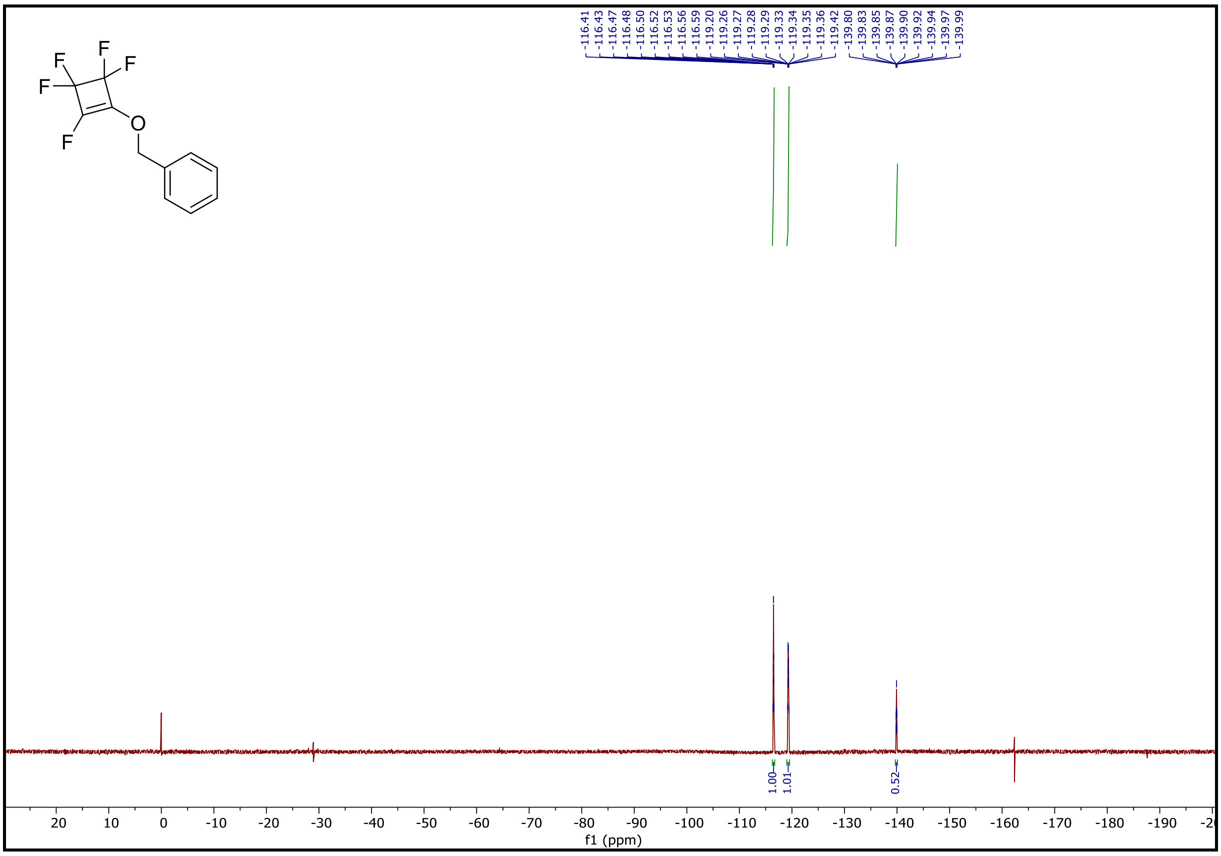

Figure S19: ^{19}F NMR spectrum of pentafluorocyclobutene **7c** ($CDCl_3$, 282 MHz)

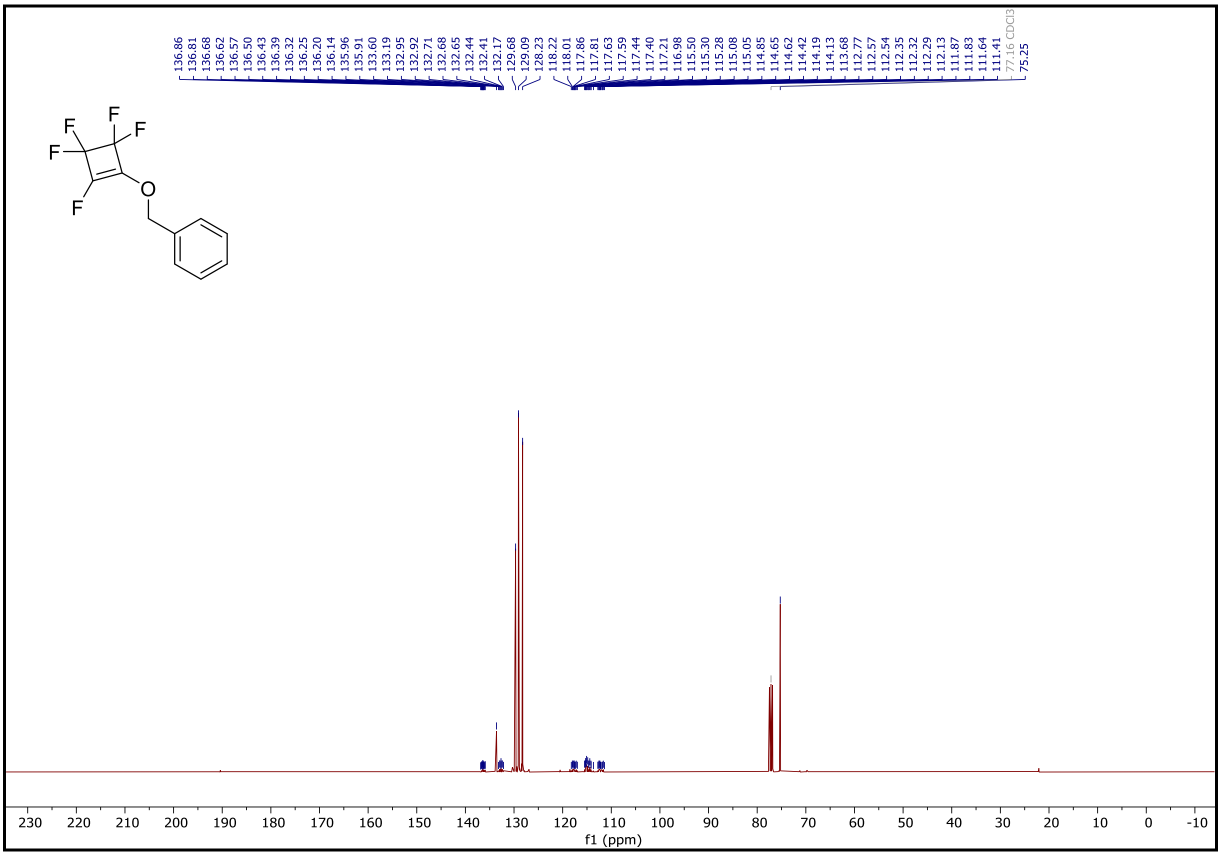

Figure S20: ^{13}C NMR spectrum of pentafluorocyclobutene **7c** ($CDCl_3$, 101 MHz)

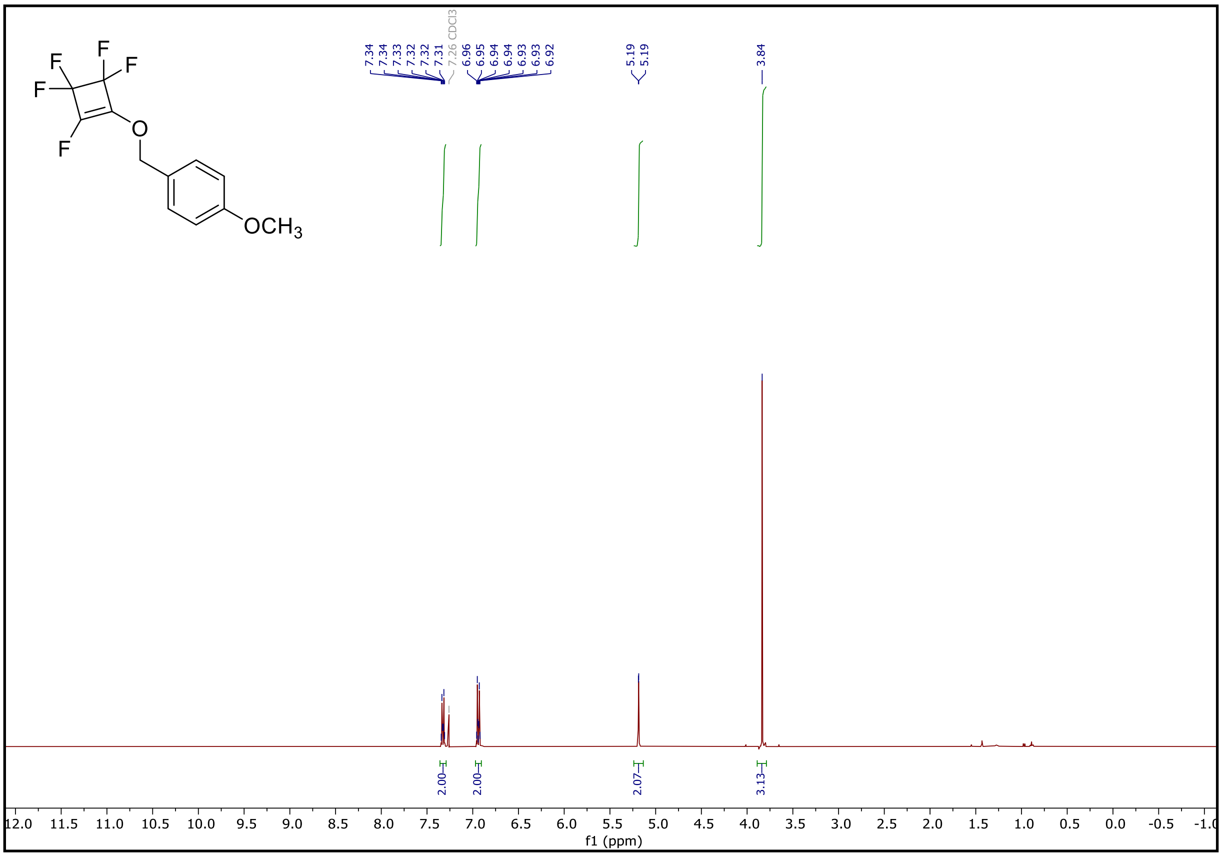

Figure S21: ^{1}H NMR spectrum of pentafluorocyclobutene **7d** ($CDCl_3$, 400 MHz)

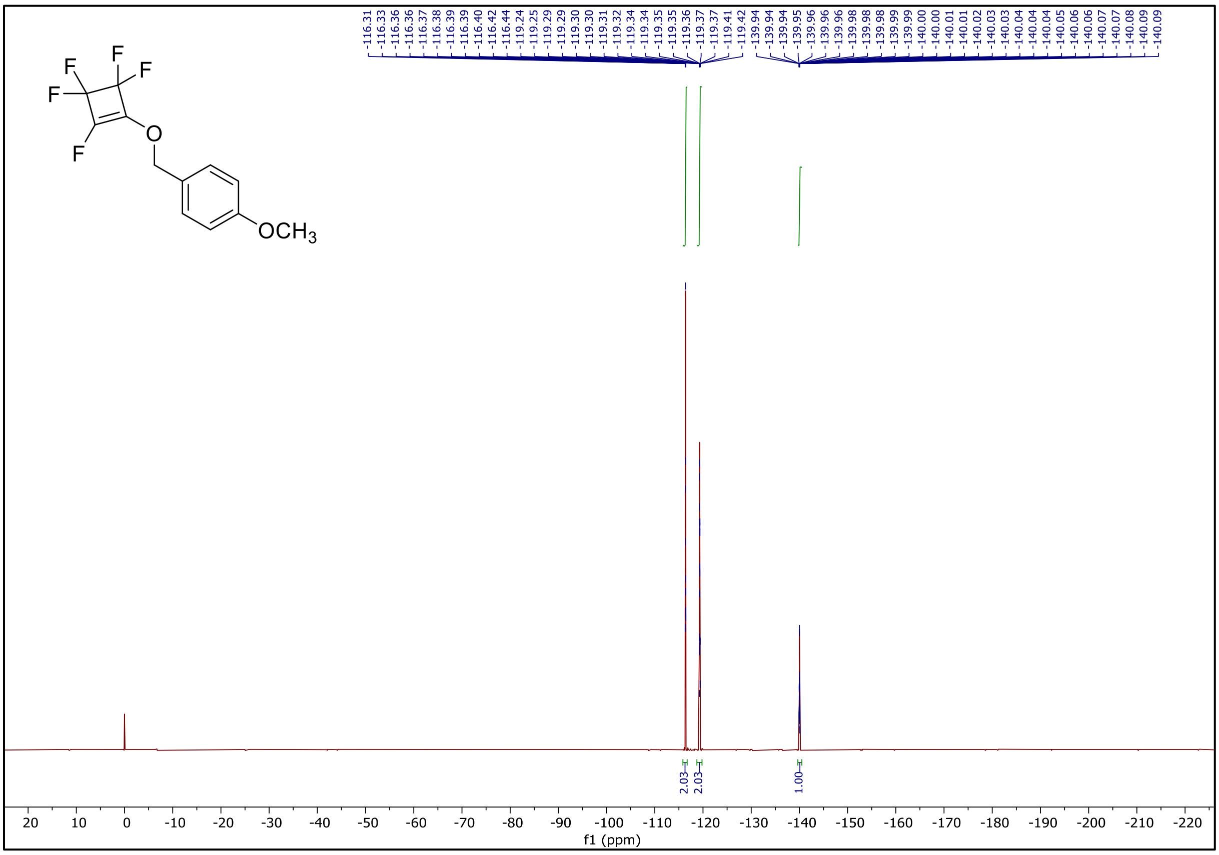

Figure S22: ^{19}F NMR spectrum of pentafluorocyclobutene **7d** ($CDCl_3$, 376 MHz)

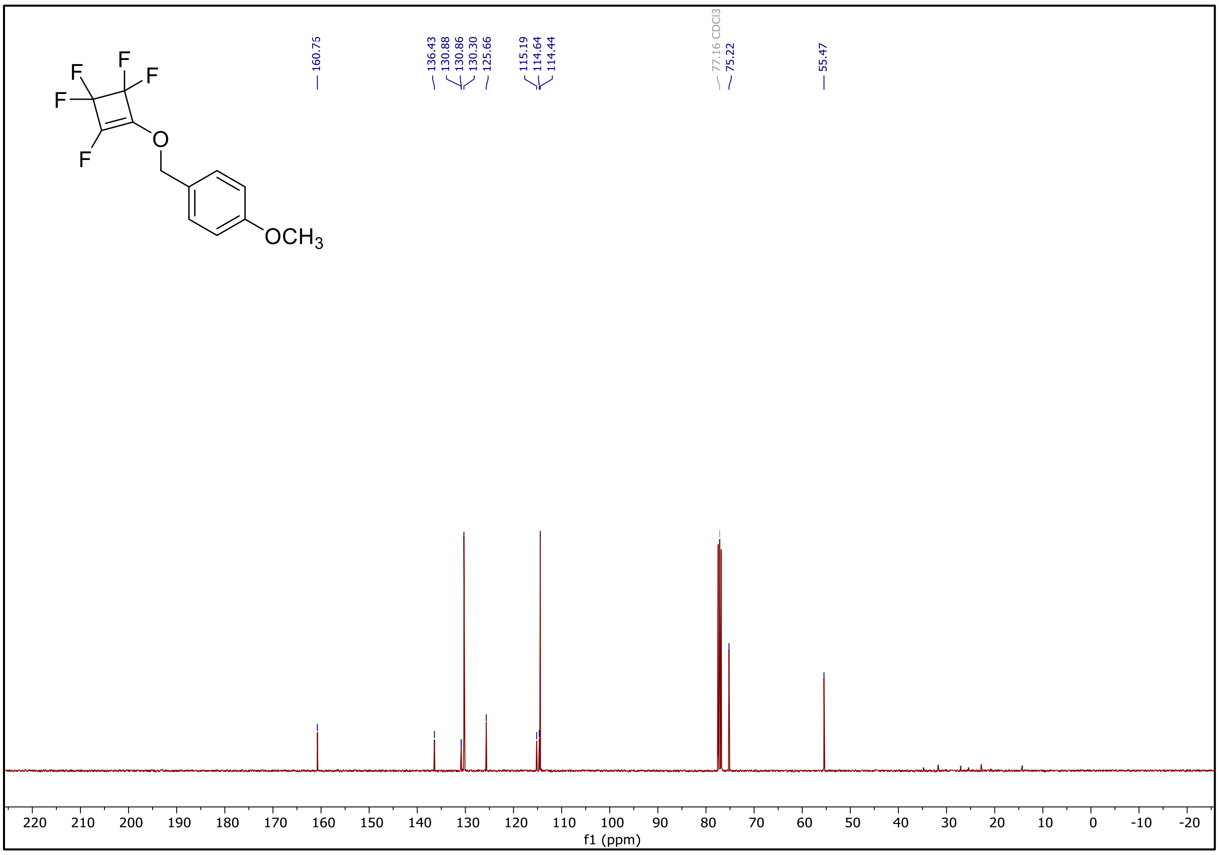

Figure S23: ^{13}C NMR spectrum of pentafluorocyclobutene **7d** ($CDCl_3$, 101 MHz)

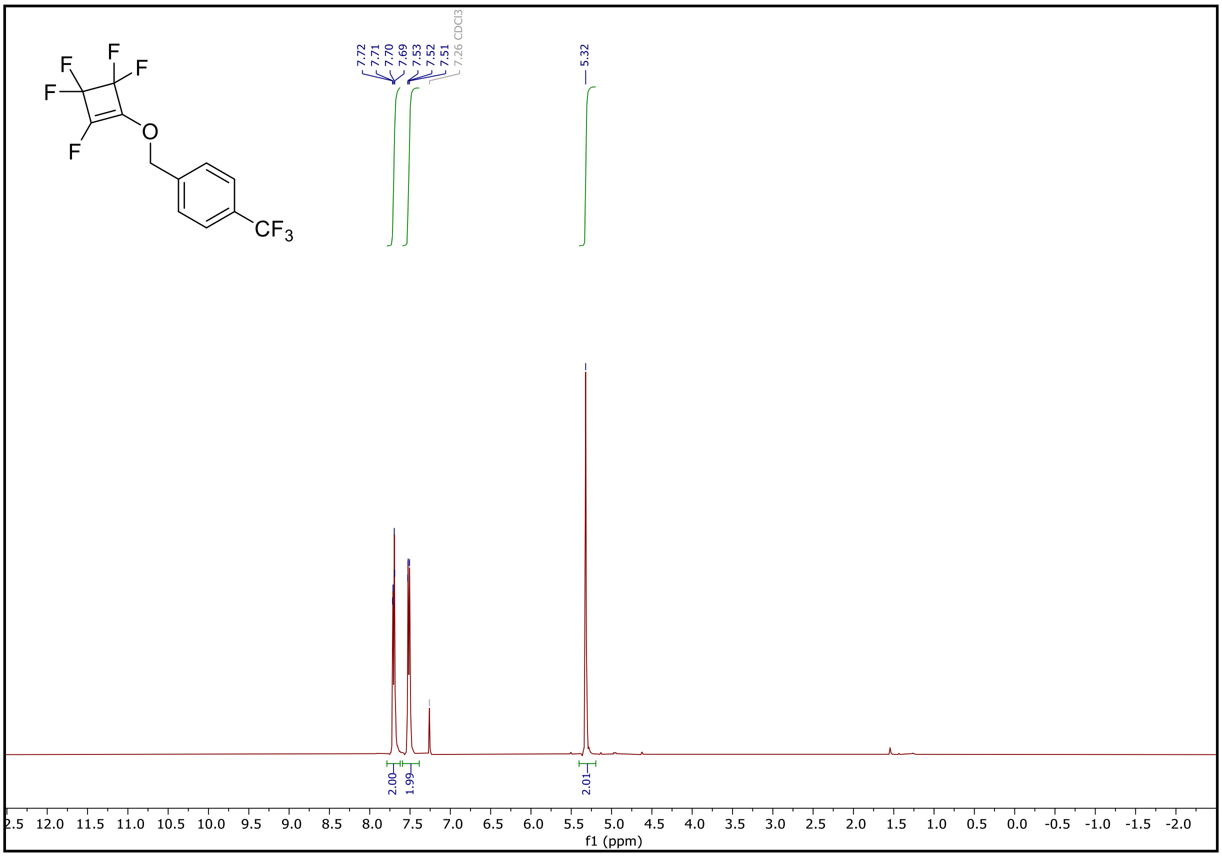

Figure S24: ^{1}H NMR spectrum of pentafluorocyclobutene **7e** ($CDCl_3$, 400 MHz)

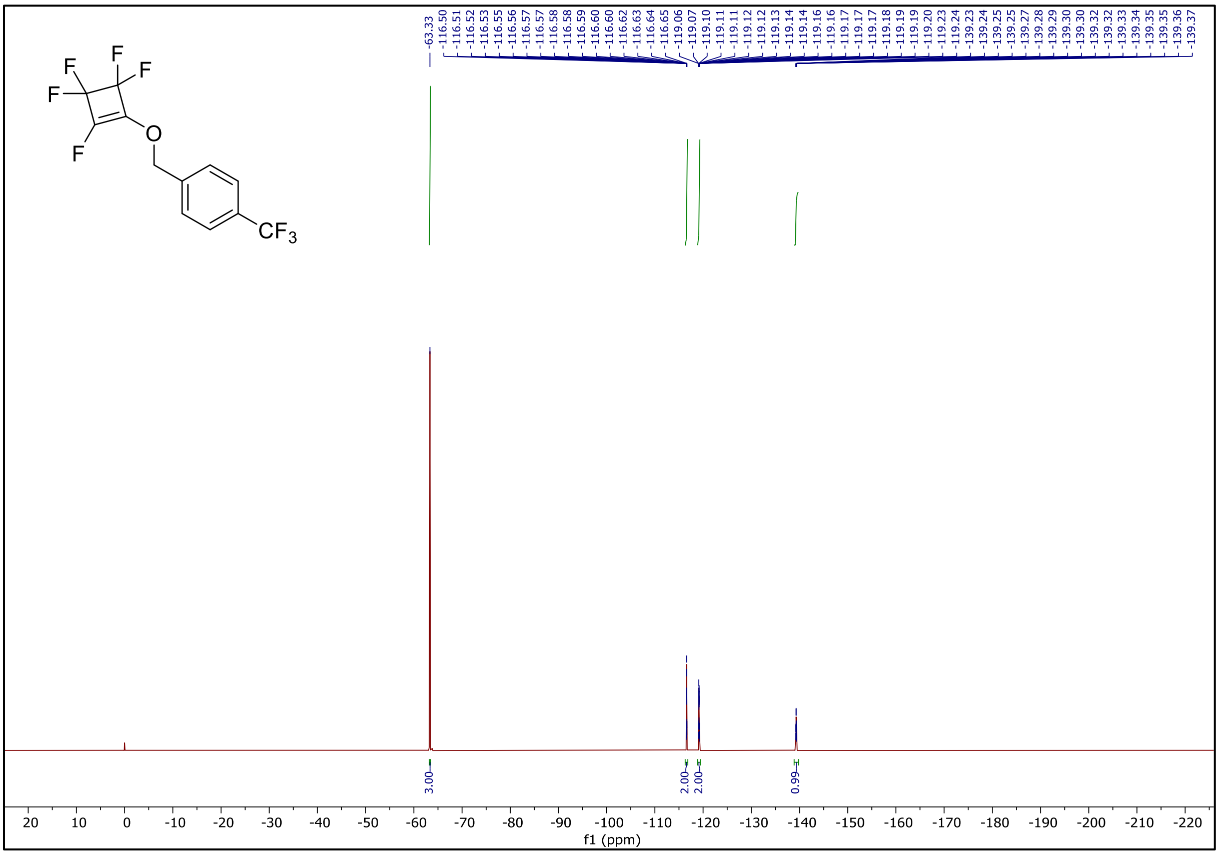

Figure S25: ^{19}F NMR spectrum of pentafluorocyclobutene **7e** ($CDCl_3$, 376 MHz)

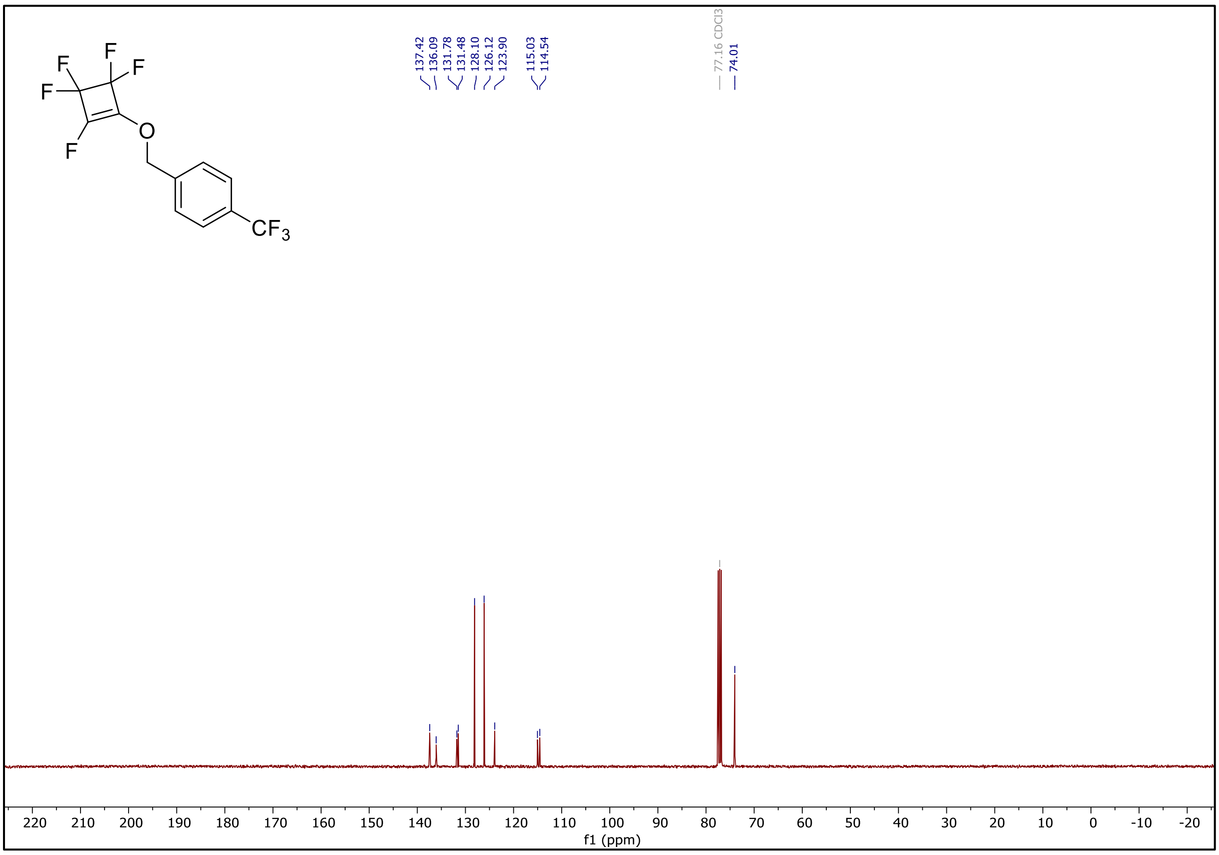

Figure S26: ^{13}C NMR spectrum of pentafluorocyclobutene **7e** ($CDCl_3$, 101 MHz, ^{1}H and ^{19}F decoupling)

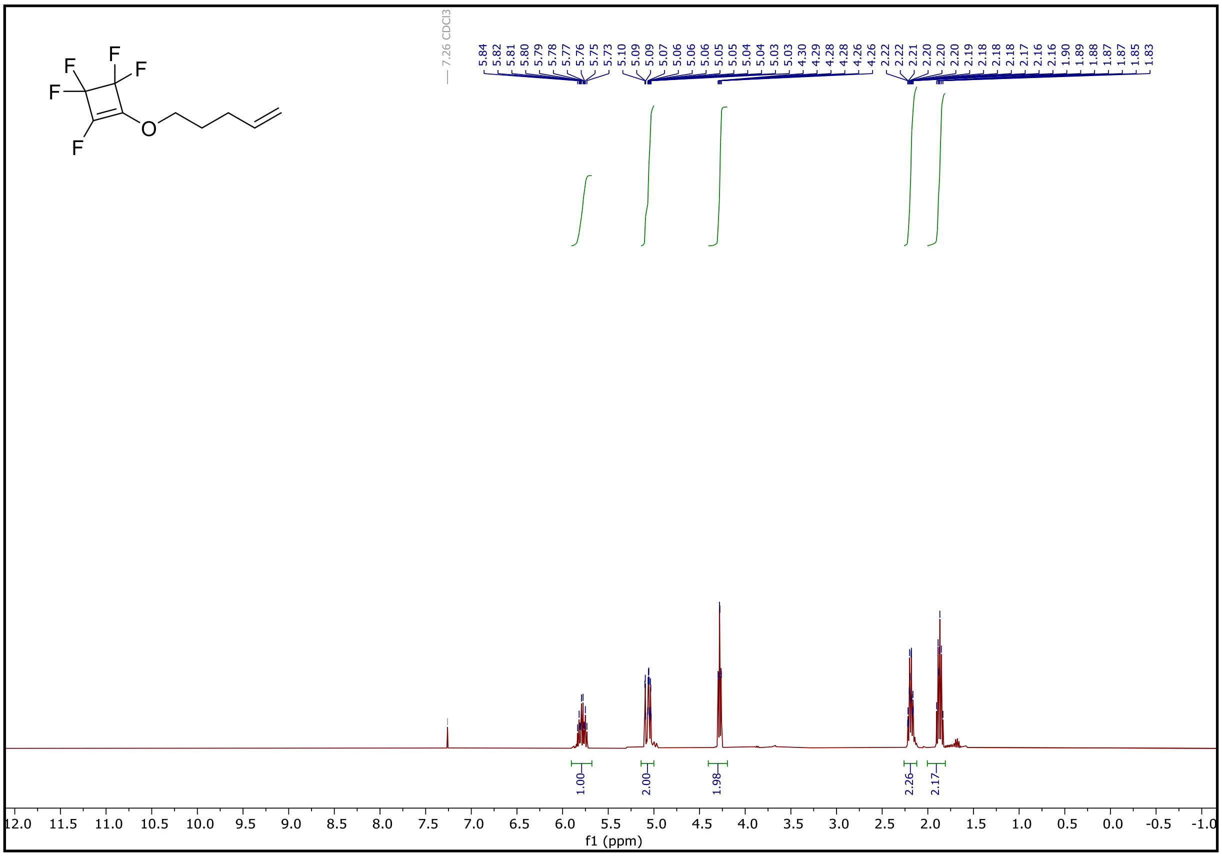

Figure S27: ^{1}H NMR spectrum of pentafluorocyclobutene **11a** ($CDCl_3$, 400 MHz)

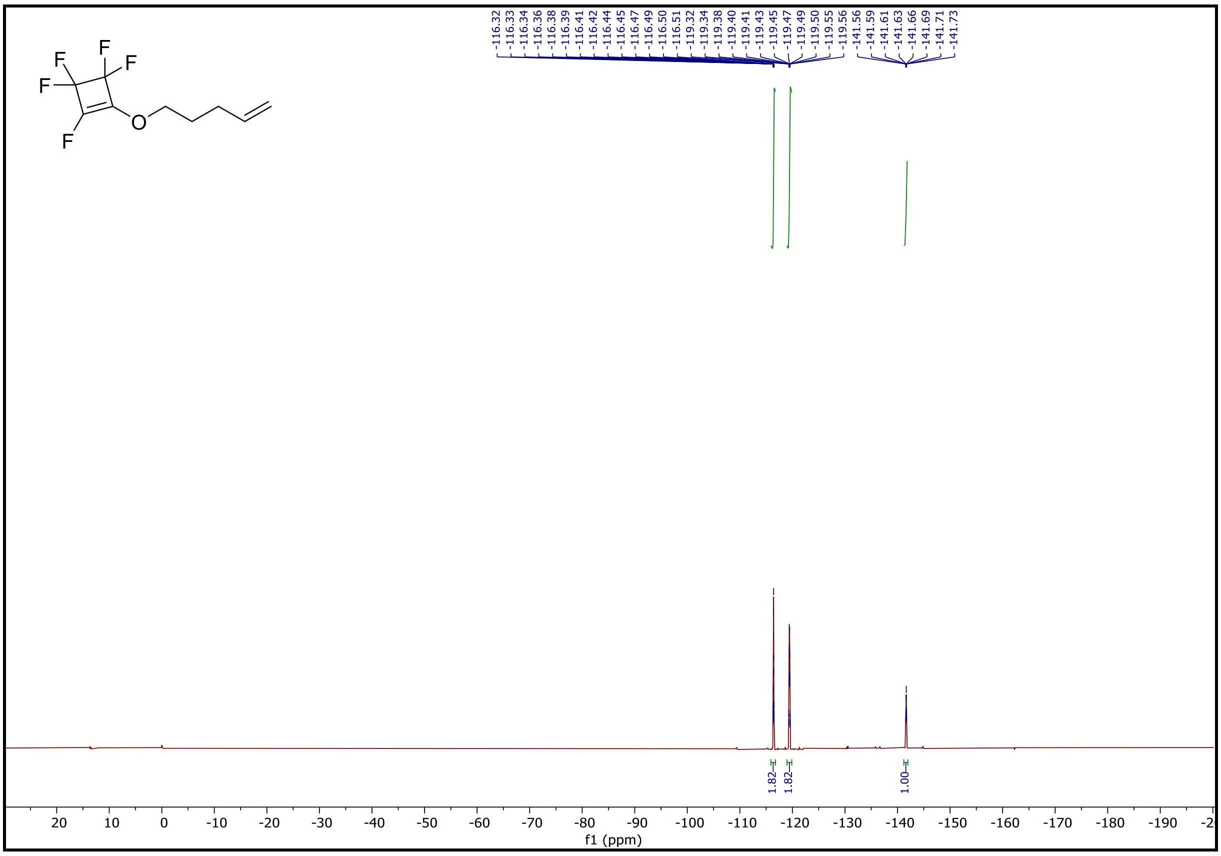

Figure S28: ^{19}F NMR spectrum of pentafluorocyclobutene **11a** ($CDCl_3$, 282 MHz)

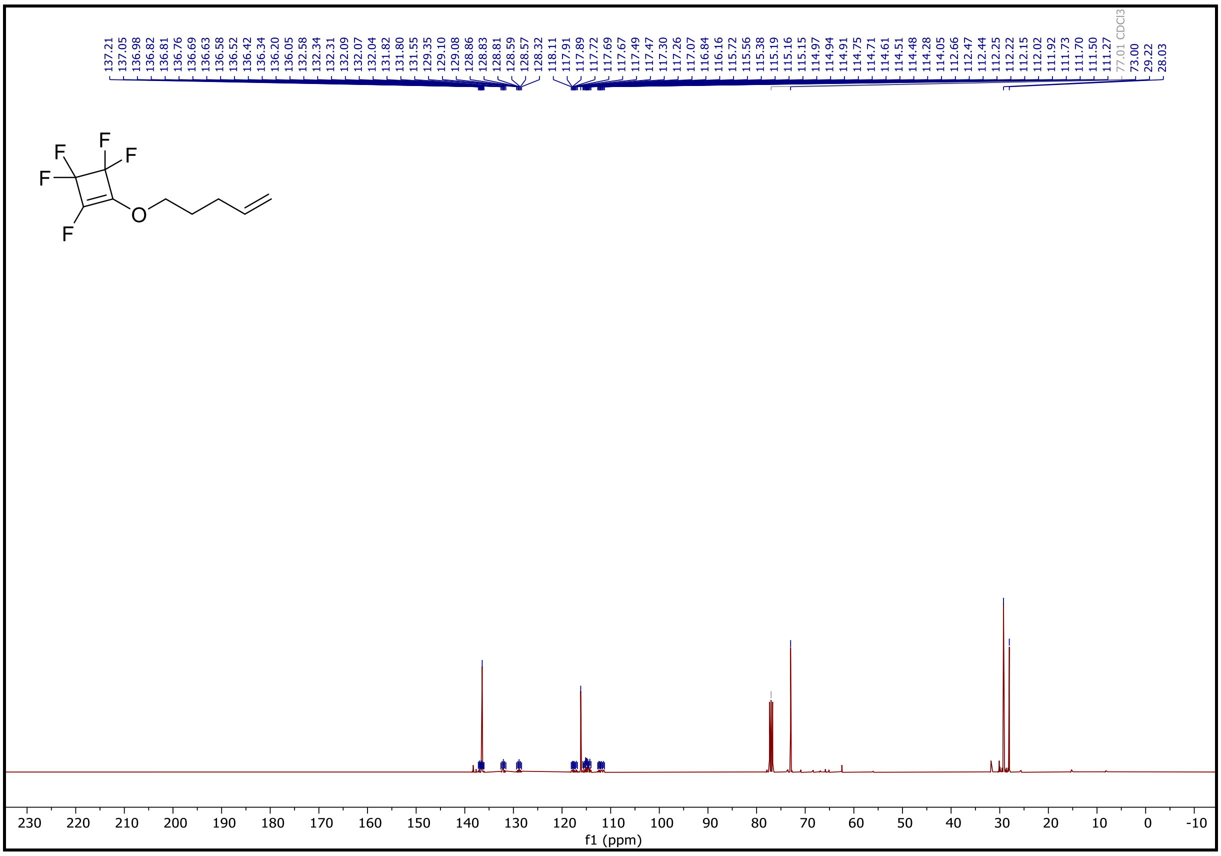

Figure S29: ^{13}C NMR spectrum of pentafluorocyclobutene **11a** ($CDCl_3$, 101 MHz)

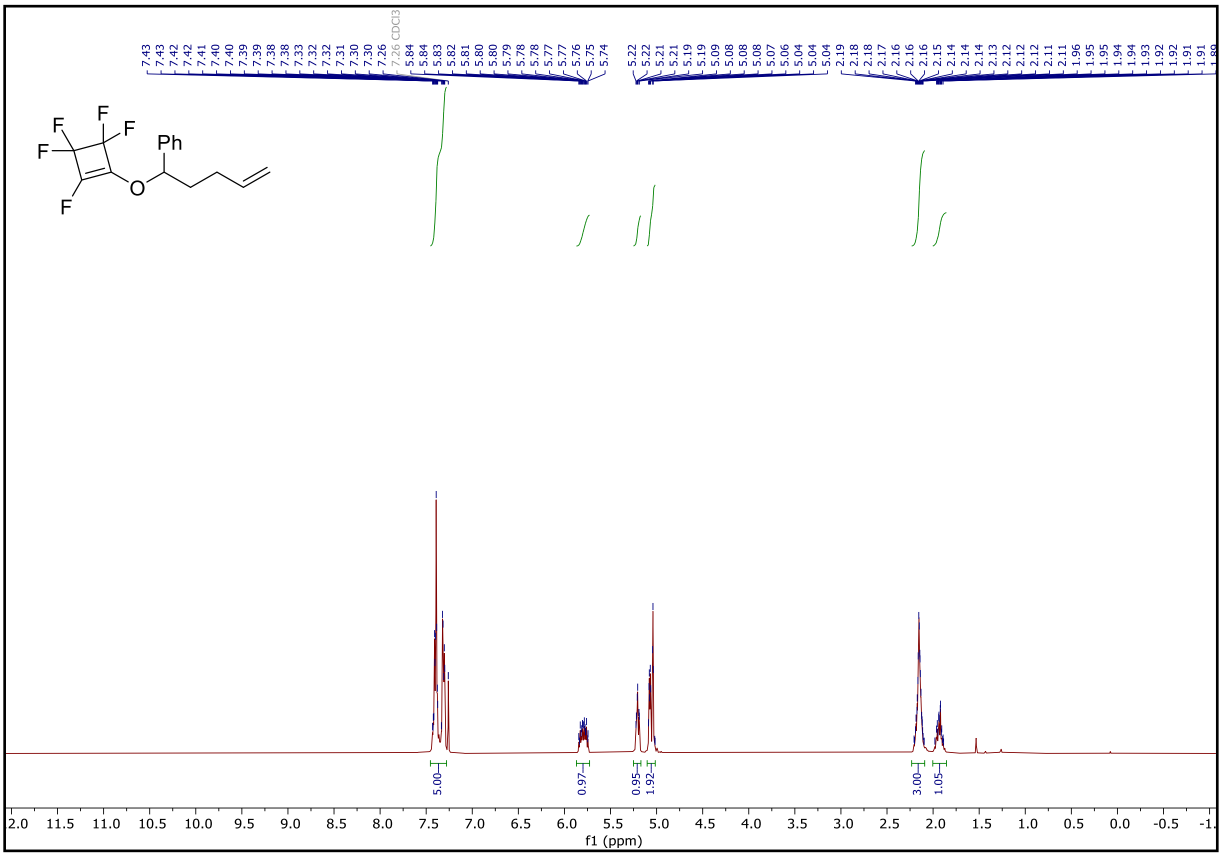

Figure S30: ^{1}H NMR spectrum of pentafluorocyclobutene **11b** ($CDCl_3$, 400 MHz)

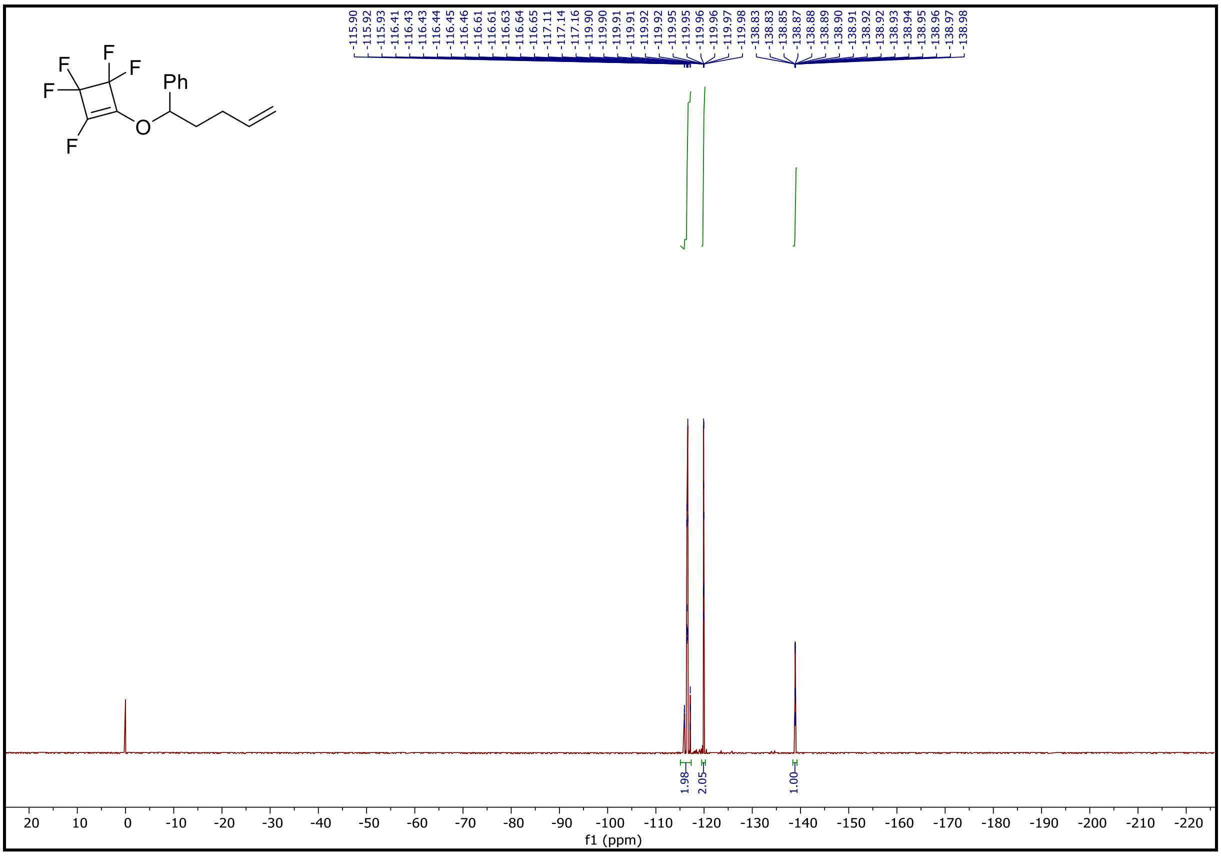

Figure S31: ^{19}F NMR spectrum of pentafluorocyclobutene **11a** ($CDCl_3$, 376 MHz)

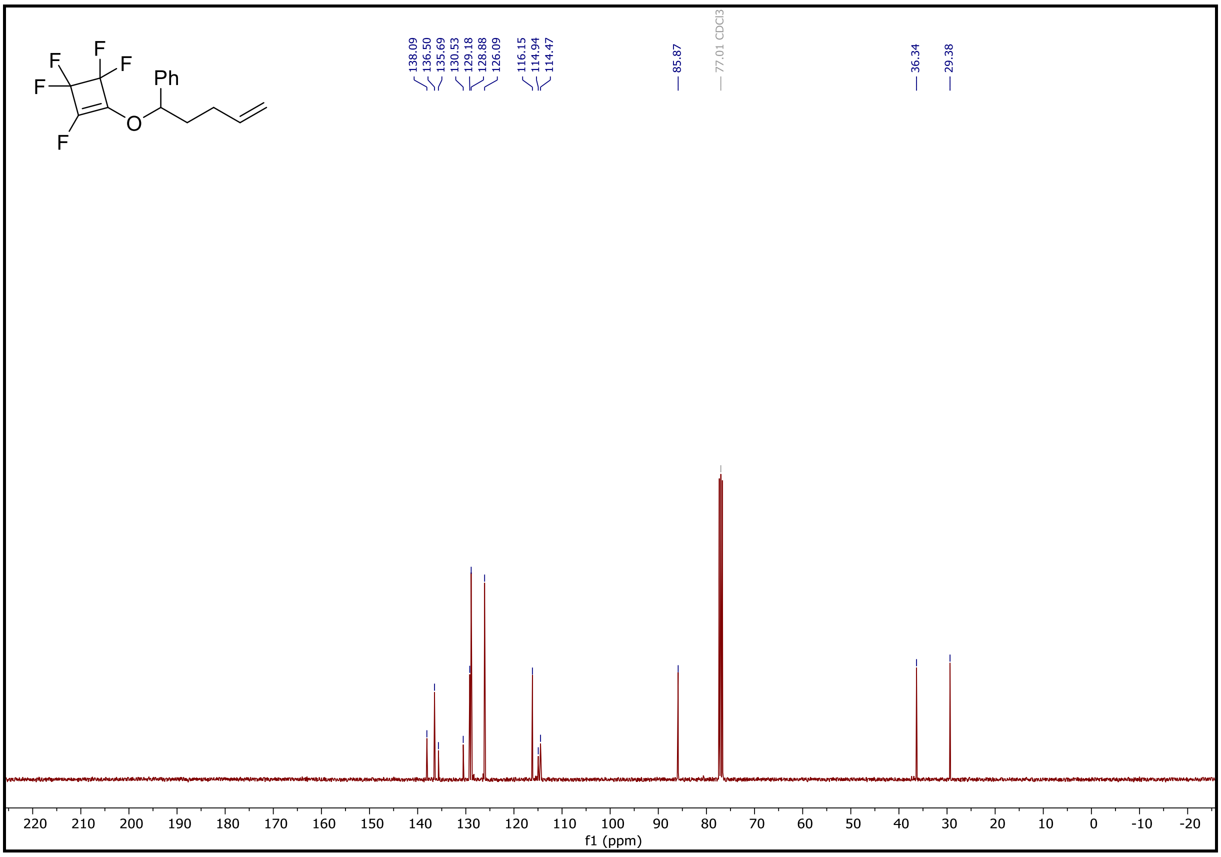

Figure S32: ^{13}C NMR spectrum of pentafluorocyclobutene **11b** ($CDCl_3$,101 MHz, ^{1}H and ^{19}F decoupling)

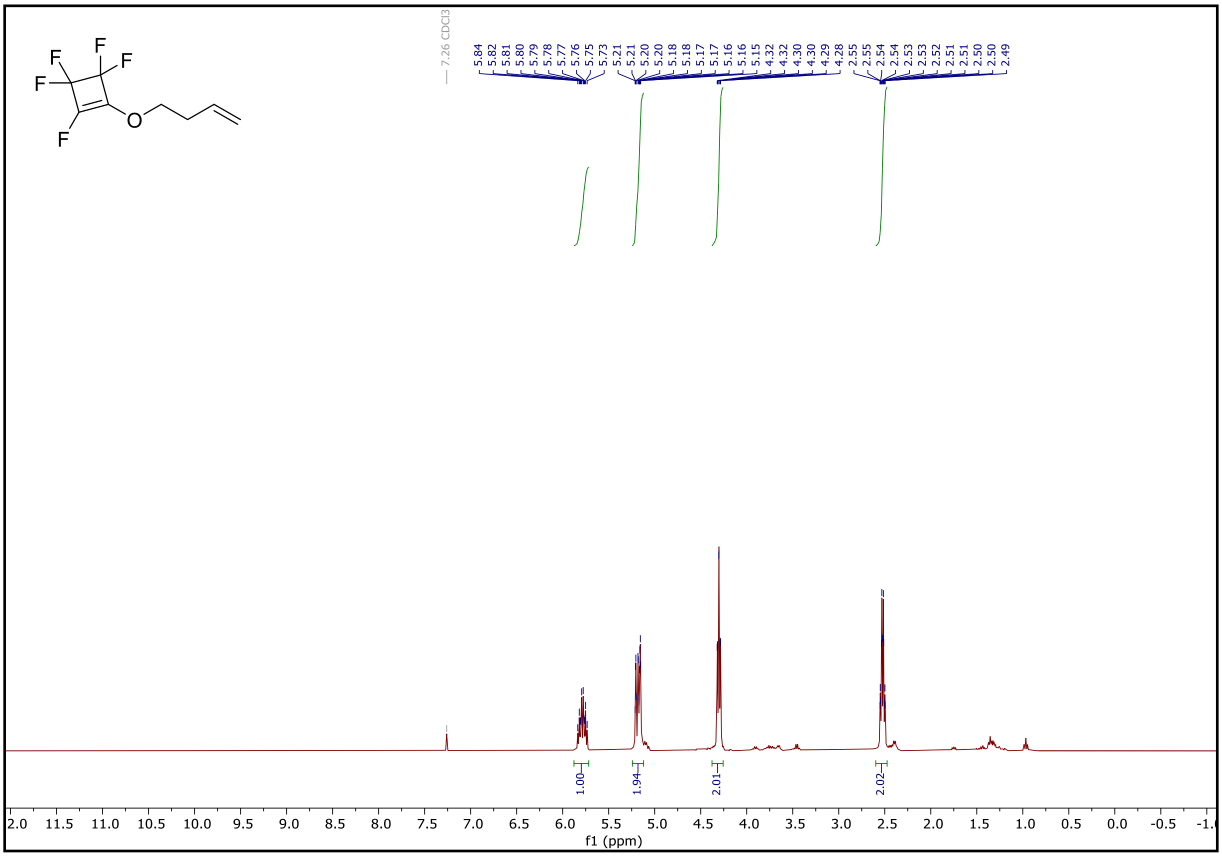

Figure S33: ^{1}H NMR spectrum of pentafluorocyclobutene **2a** ($CDCl_3$, 400 MHz)

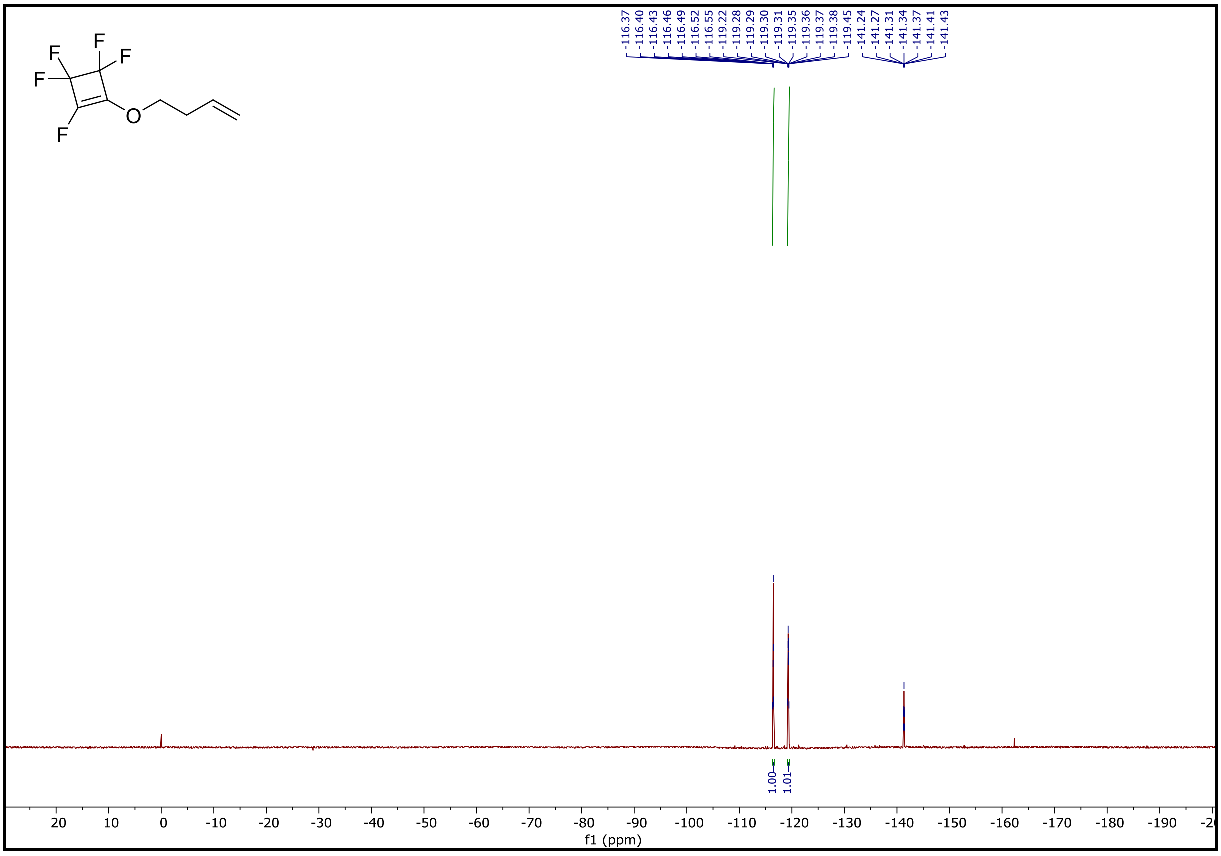

Figure S34: ^{19}F NMR spectrum of pentafluorocyclobutene **2a** ($CDCl_3$, 282 MHz)

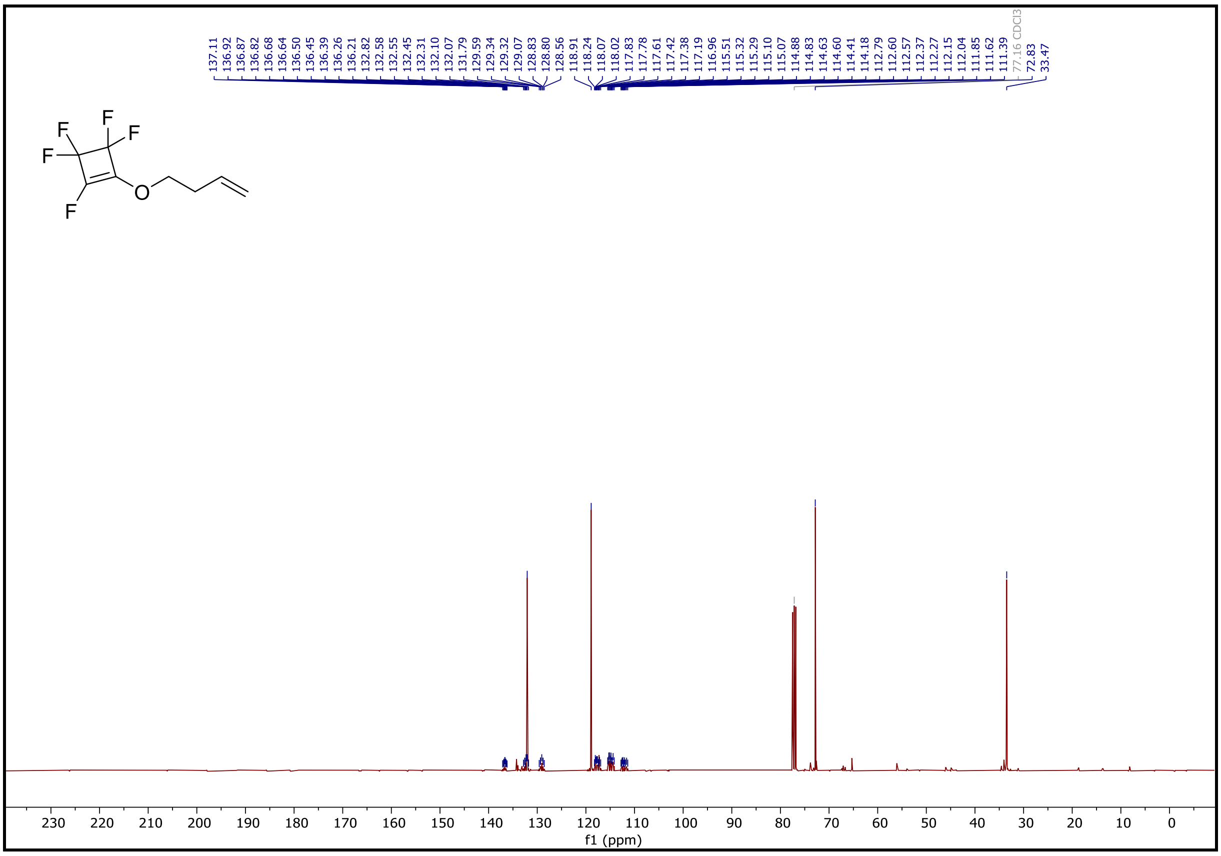

Figure S35: ^{13}C NMR spectrum of pentafluorocyclobutene **2a** ($CDCl_3$, 101 MHz)

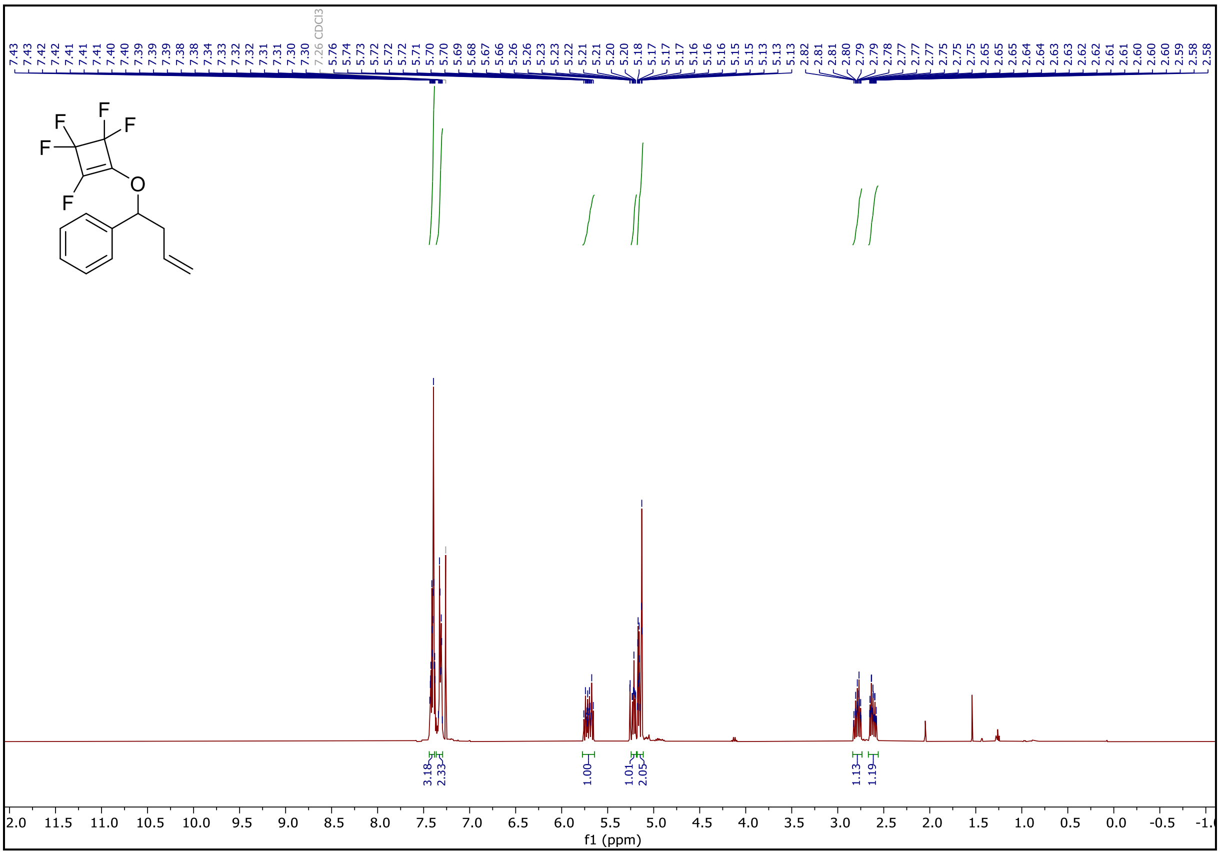

Figure S36: ^{1}H NMR spectrum of pentafluorocyclobutene **2b** ($CDCl_3$, 400 MHz)

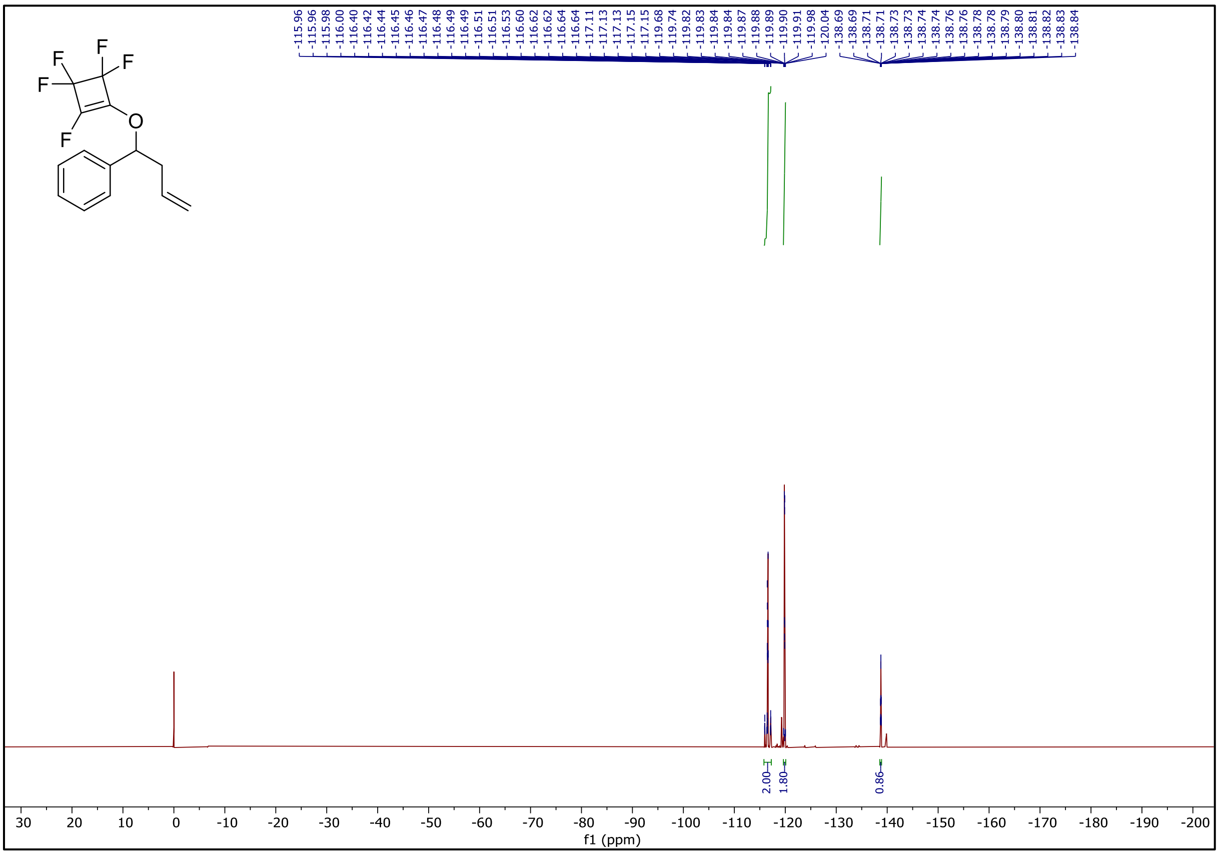

Figure S37: ^{19}F NMR spectrum of pentafluorocyclobutene **2b** ($CDCl_3$, 282 MHz)

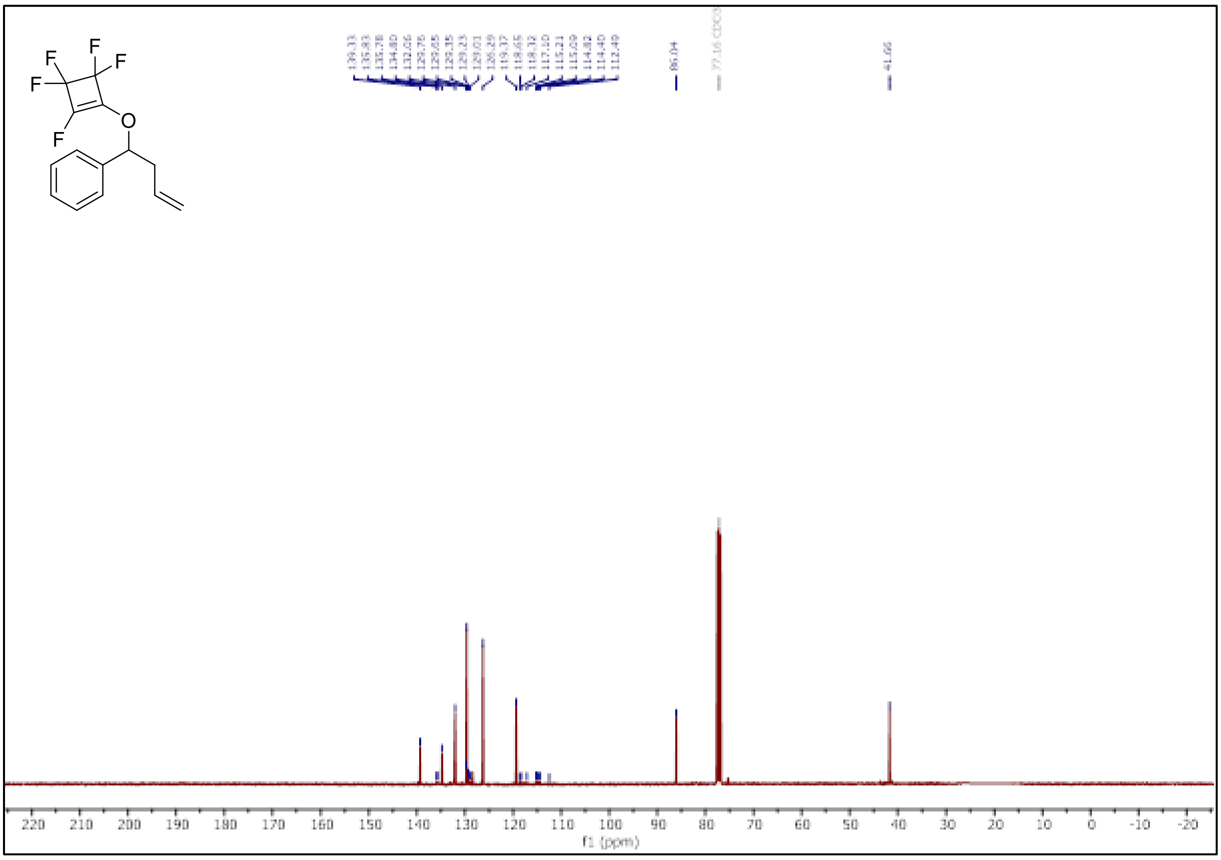

Figure S38: ^{13}C NMR spectrum of pentafluorocyclobutene **2b** ($CDCl_3$, 101 MHz)

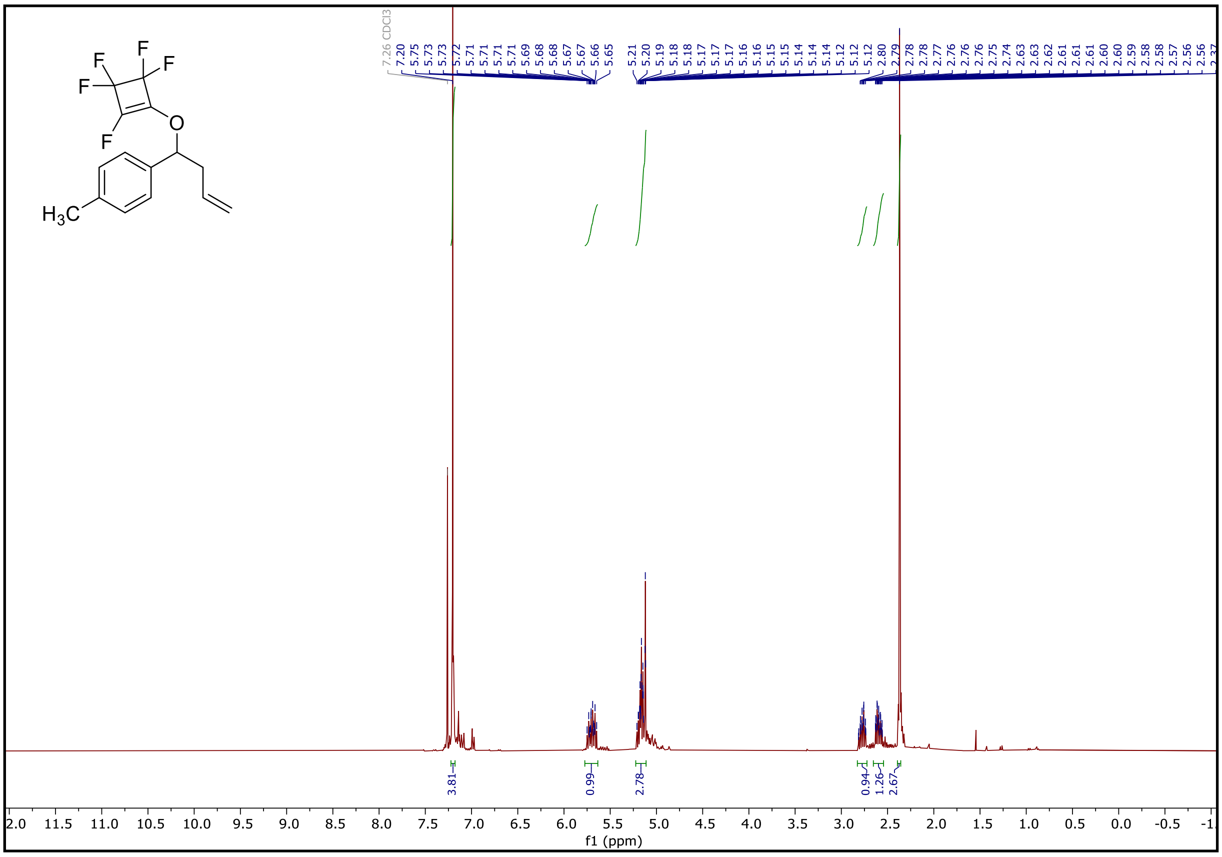

Figure S39: ^{1}H NMR spectrum of pentafluorocyclobutene **2c** ($CDCl_3$, 400 MHz)

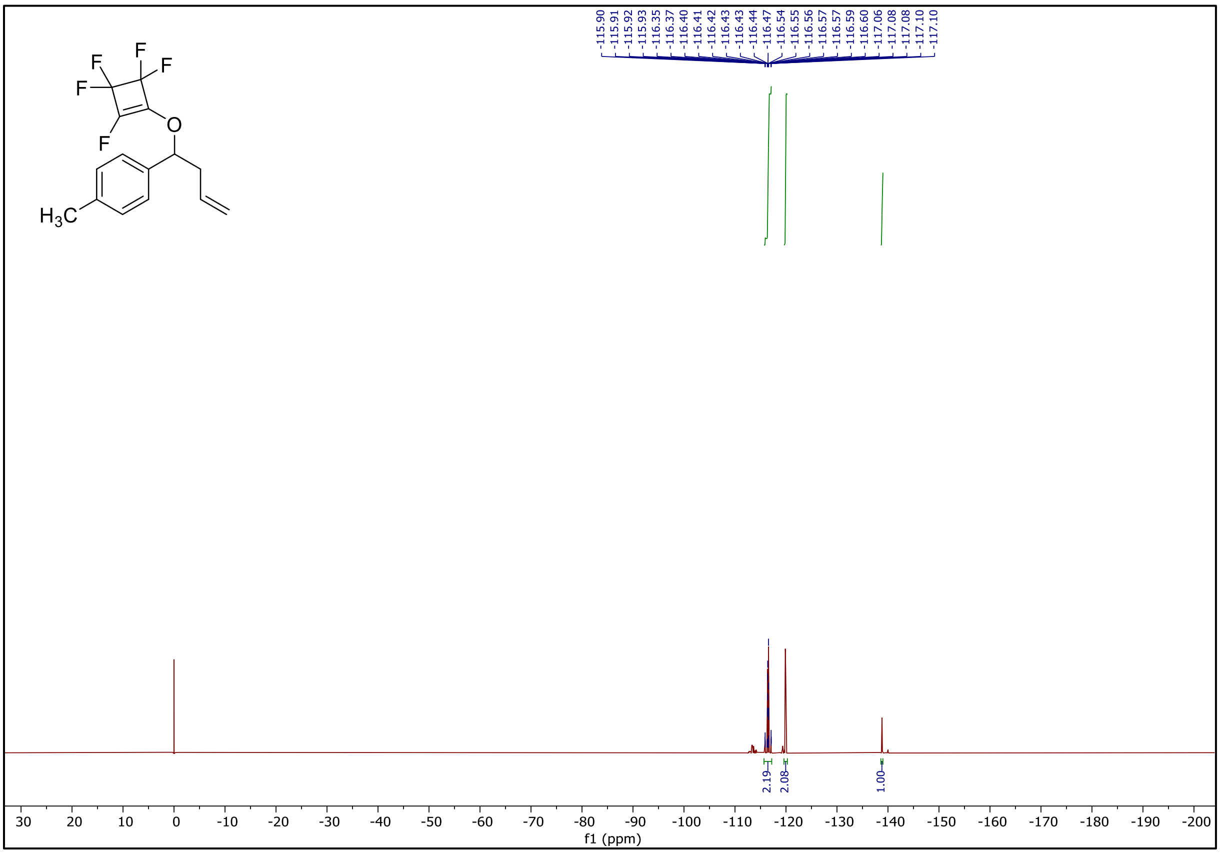

Figure S40: ^{19}F NMR spectrum of pentafluorocyclobutene **2c** ($CDCl_3$, 376 MHz)

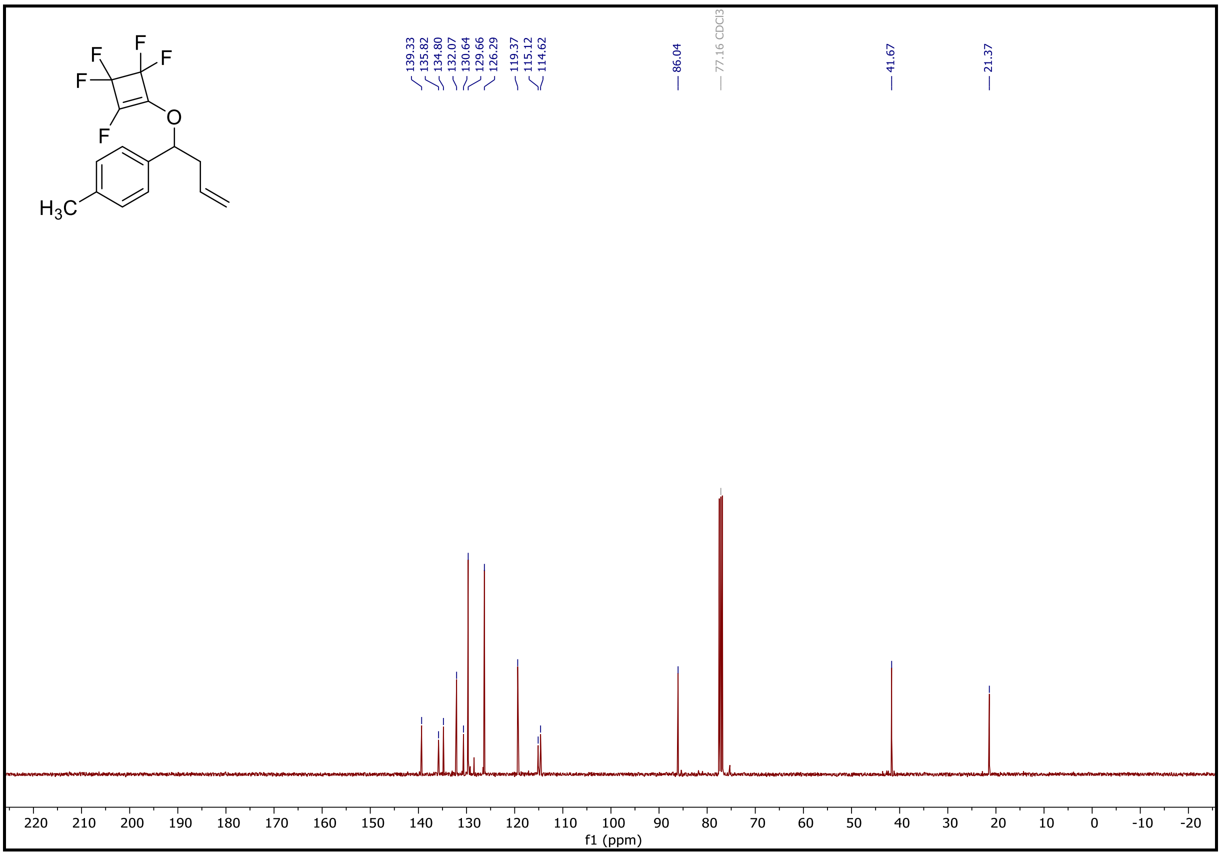

Figure S41: ^{13}C NMR spectrum of pentafluorocyclobutene **2c** ($CDCl_3$, 101 MHz, ^{1}H and ^{19}F decoupling)

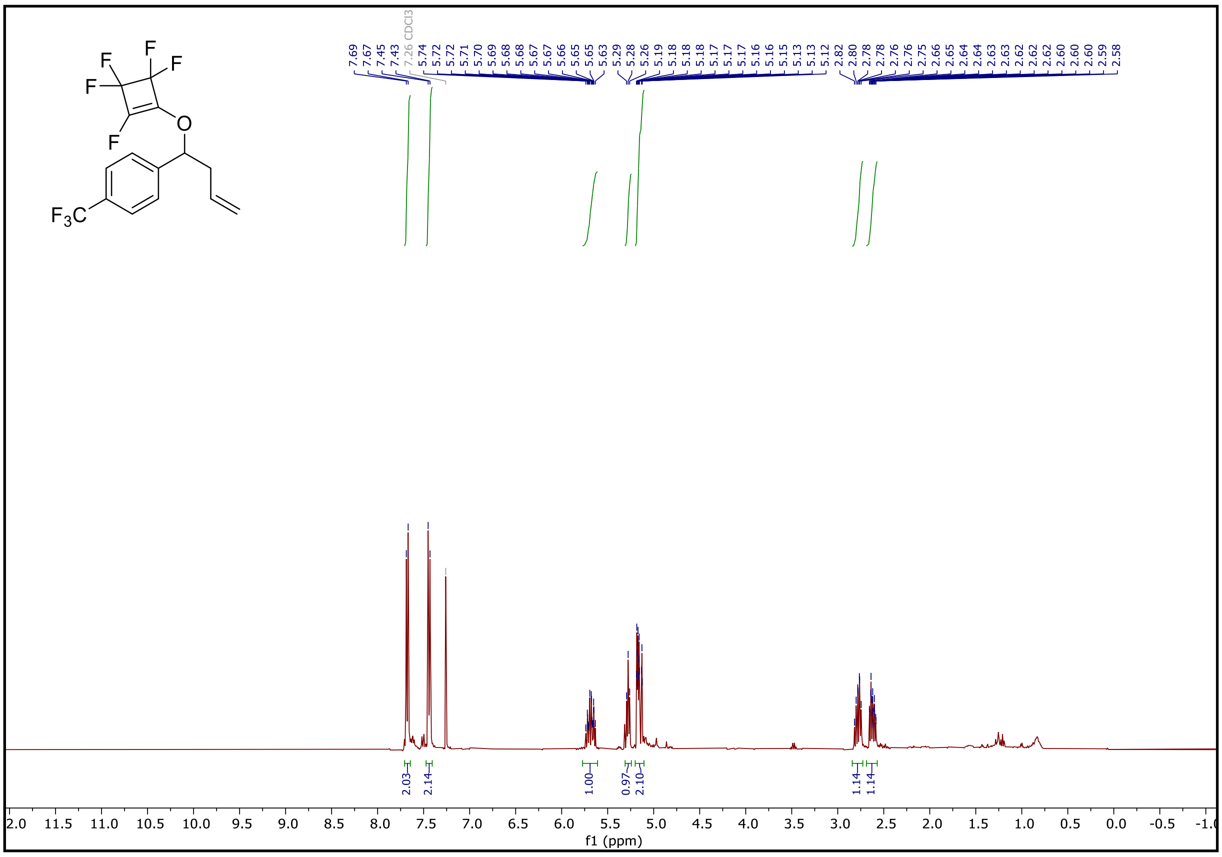

Figure S42: ^{1}H NMR spectrum of pentafluorocyclobutene **2d** ($CDCl_3$, 400 MHz)

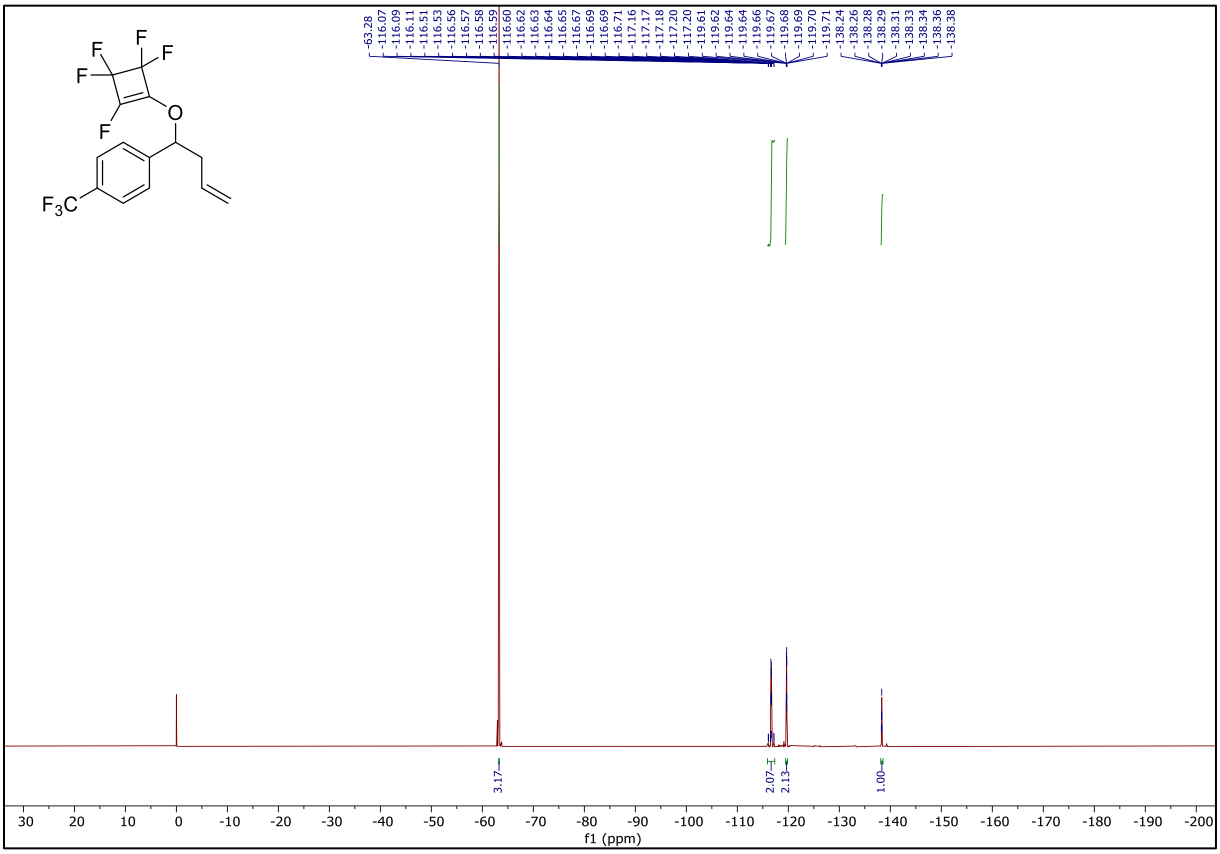

Figure S43: ^{19}F NMR spectrum of pentafluorocyclobutene **2d** ($CDCl_3$, 376 MHz)

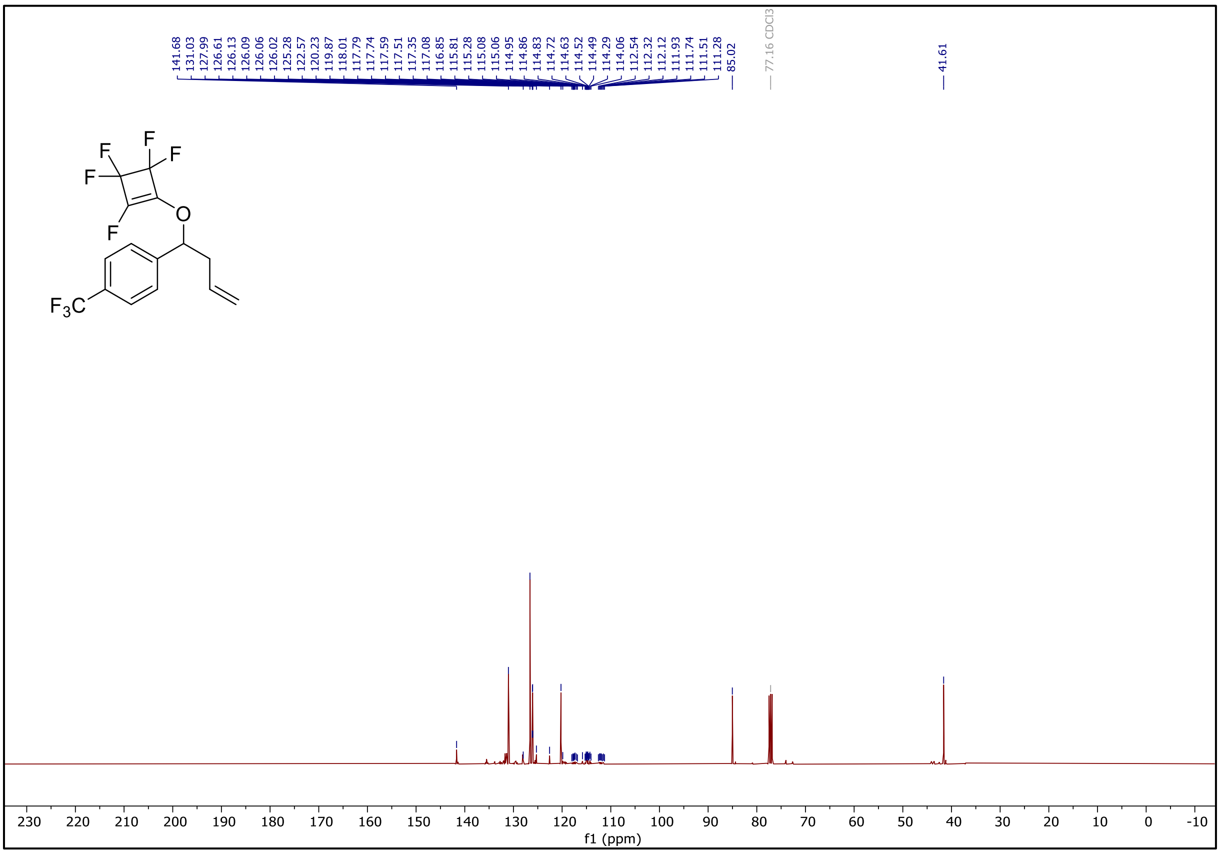

Figure S44: ^{13}C NMR spectrum of pentafluorocyclobutene **2d** ($CDCl_3$, 101 MHz)

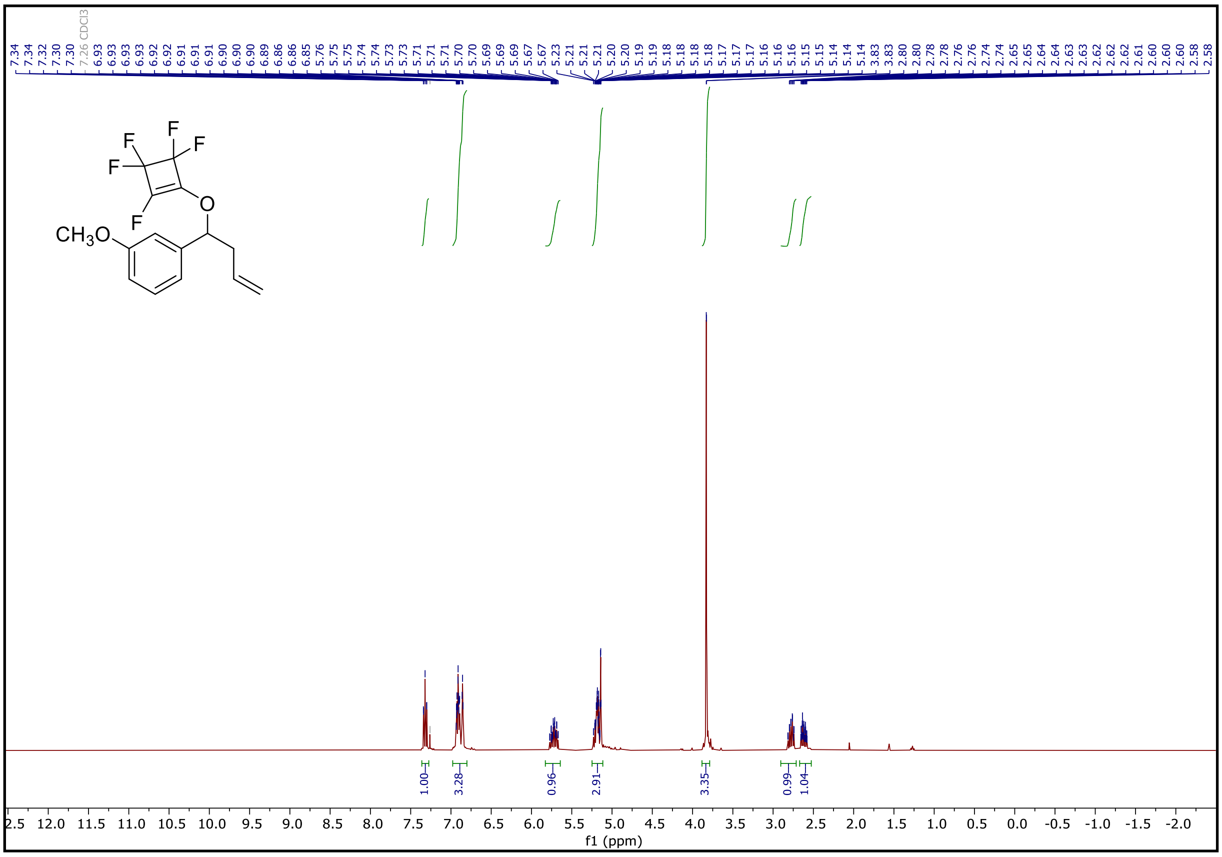

Figure S45: ^{1}H NMR spectrum of pentafluorocyclobutene **2e** ($CDCl_3$, 400 MHz)

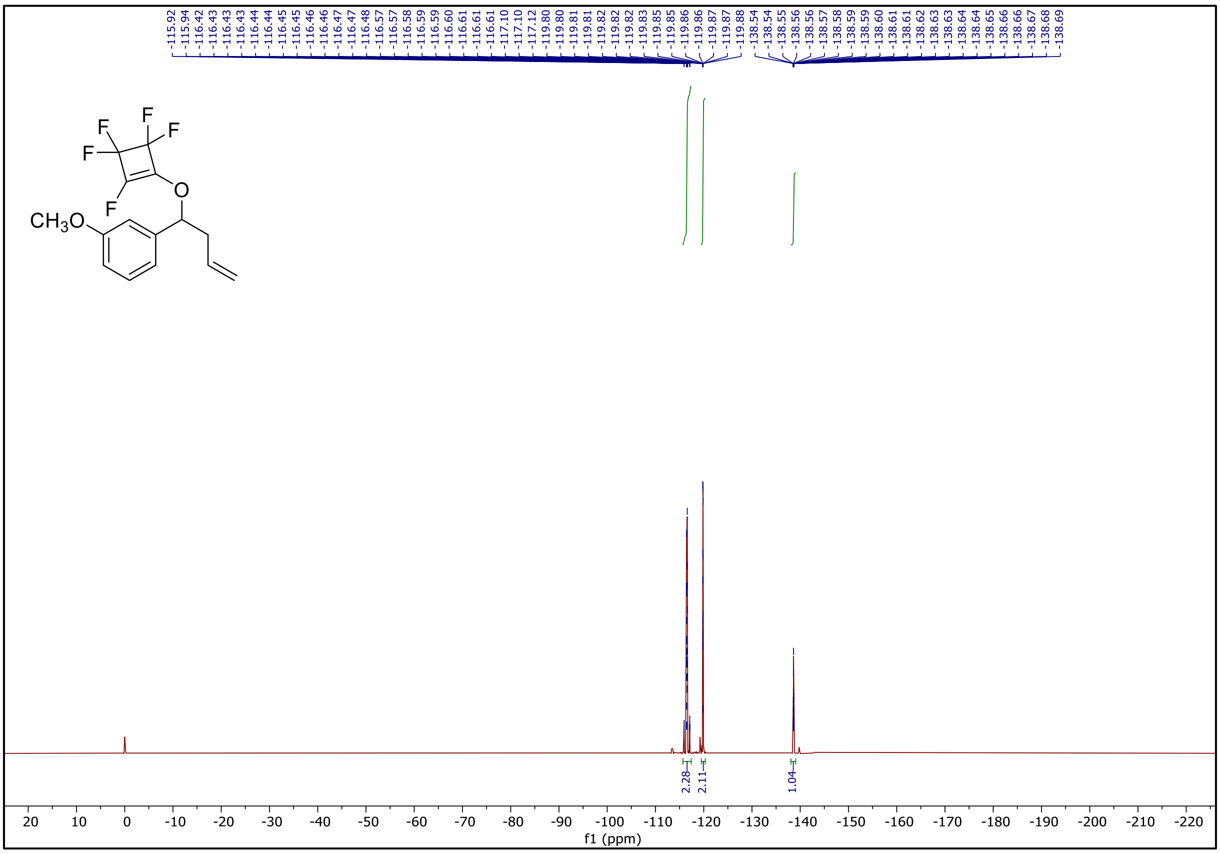

Figure S46: ^{19}F NMR spectrum of pentafluorocyclobutene **2e** ($CDCl_3$, 376 MHz)

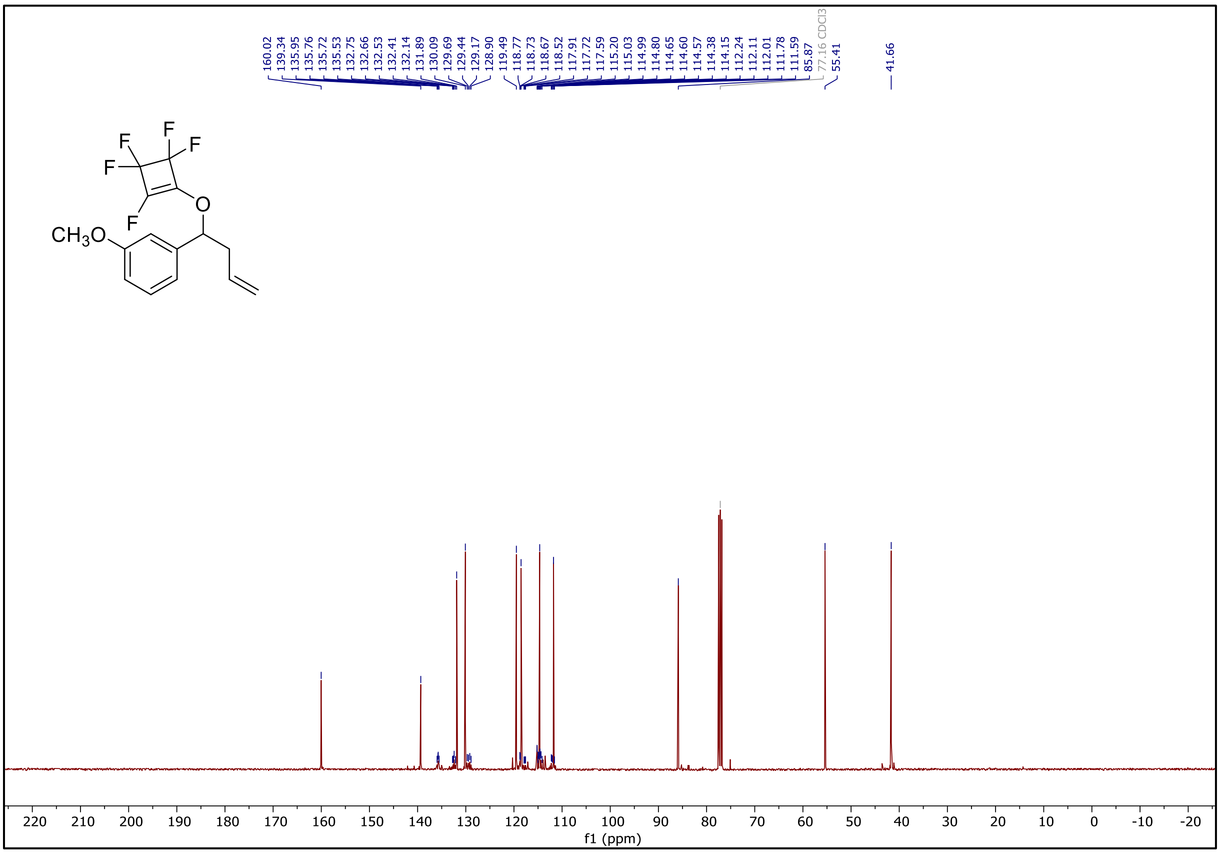

Figure S47: ^{13}C NMR spectrum of pentafluorocyclobutene **2e** ($CDCl_3$, 101 MHz)

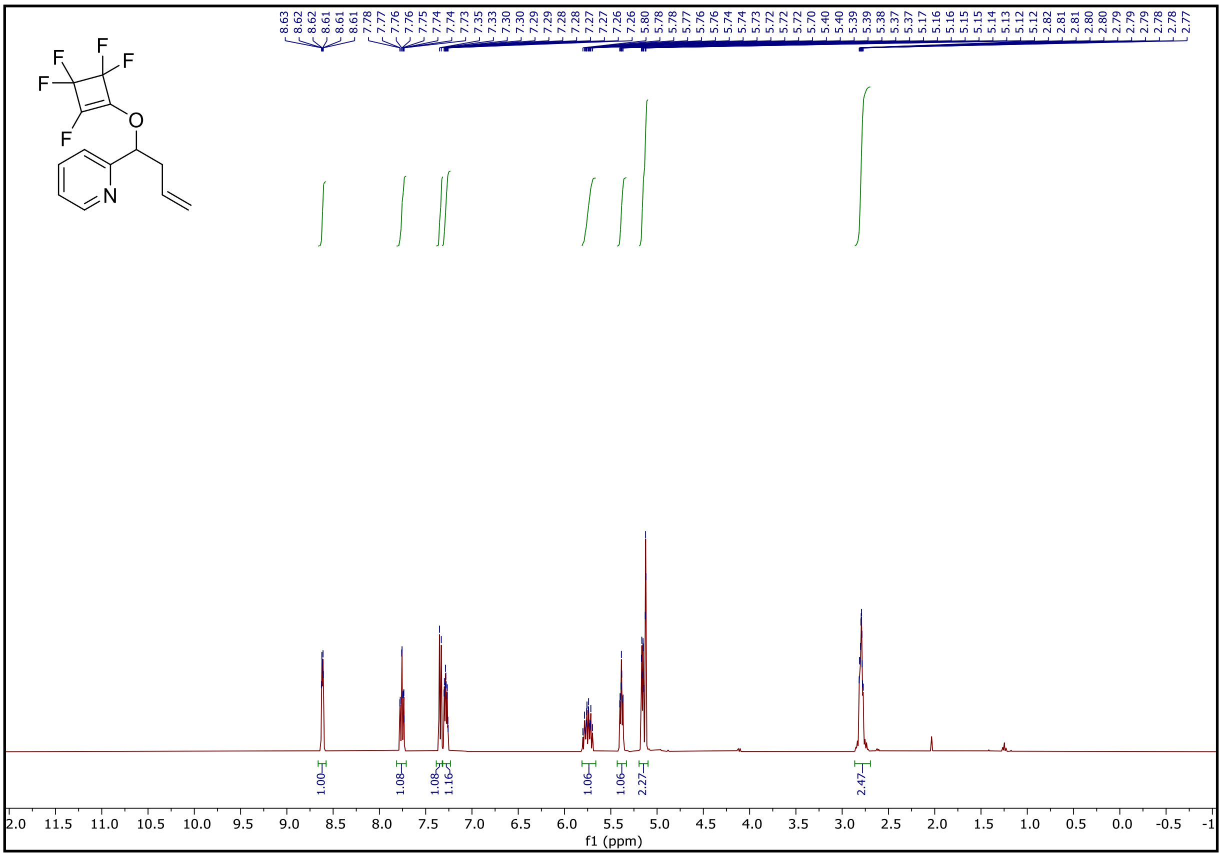

Figure S48: ^{1}H NMR spectrum of pentafluorocyclobutene **2f** ($CDCl_3$, 400 MHz)

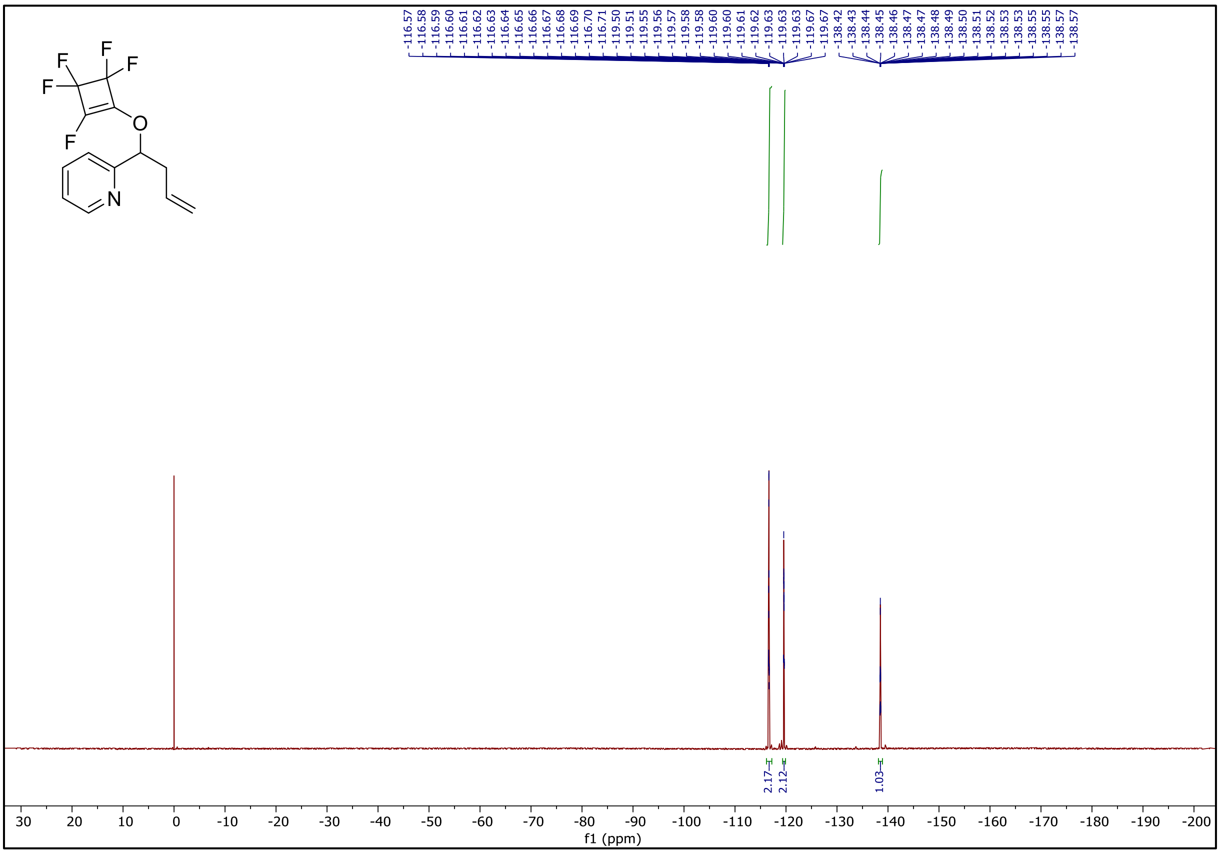

Figure S49: ^{19}F NMR spectrum of pentafluorocyclobutene **2f** ($CDCl_3$, 376 MHz)

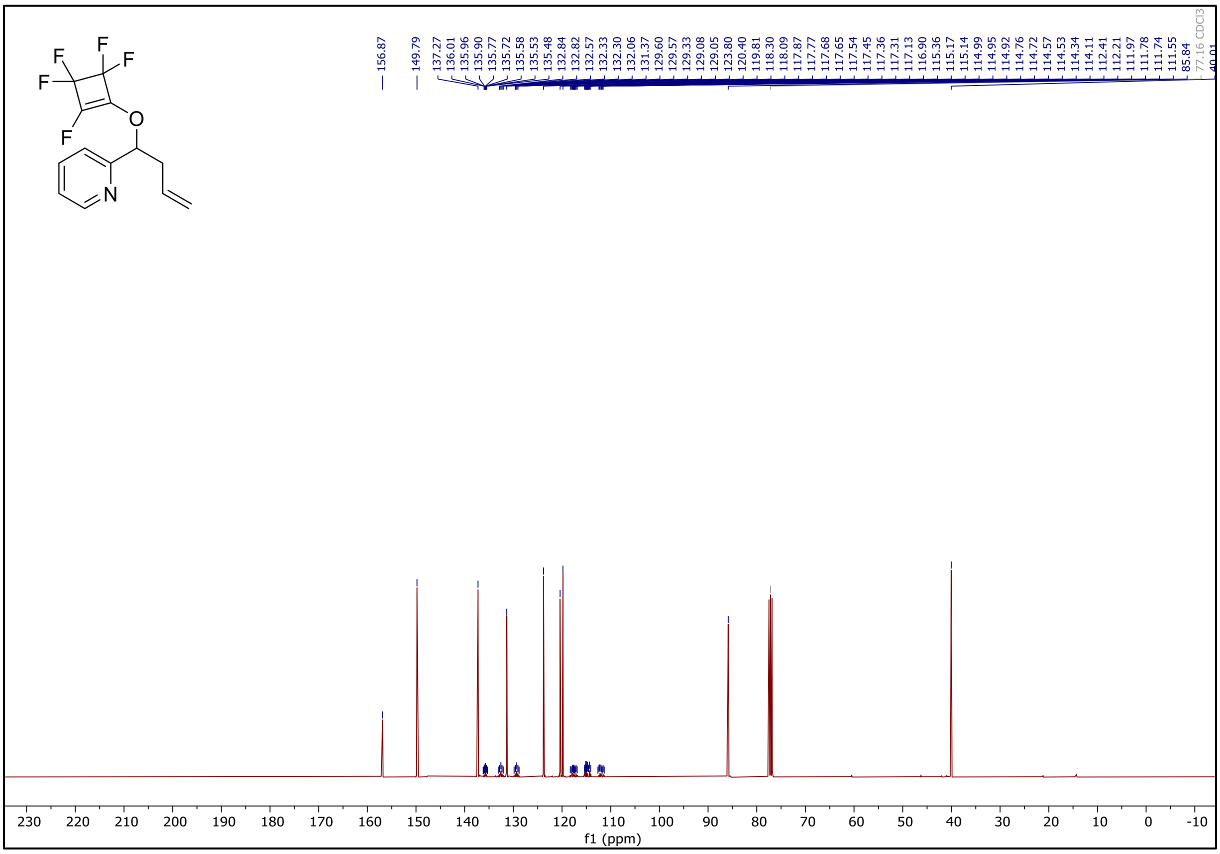

Figure S50: ^{13}C NMR spectrum of pentafluorocyclobutene **2f** ($CDCl_3$, 101 MHz)

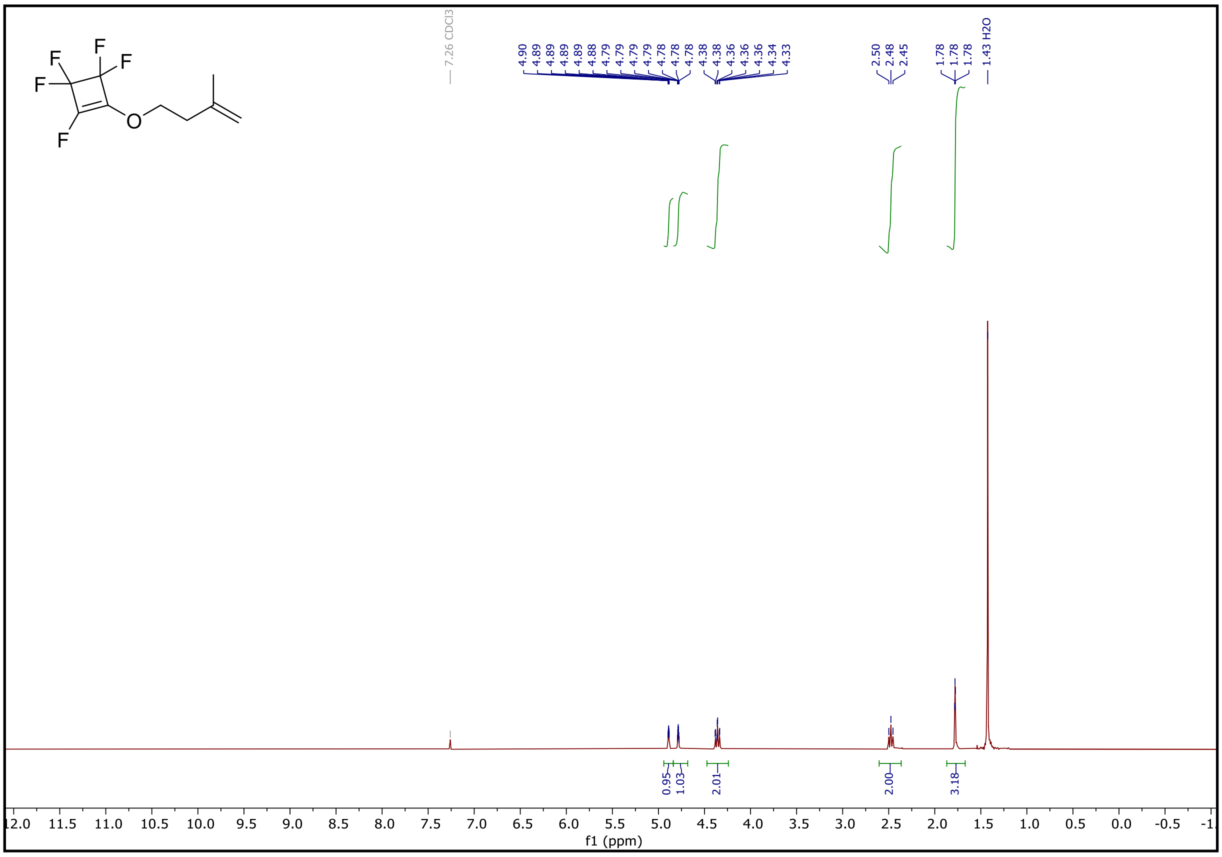

Figure S51: ^{1}H NMR spectrum of pentafluorocyclobutene **2g** ($CDCl_3$, 400 MHz)

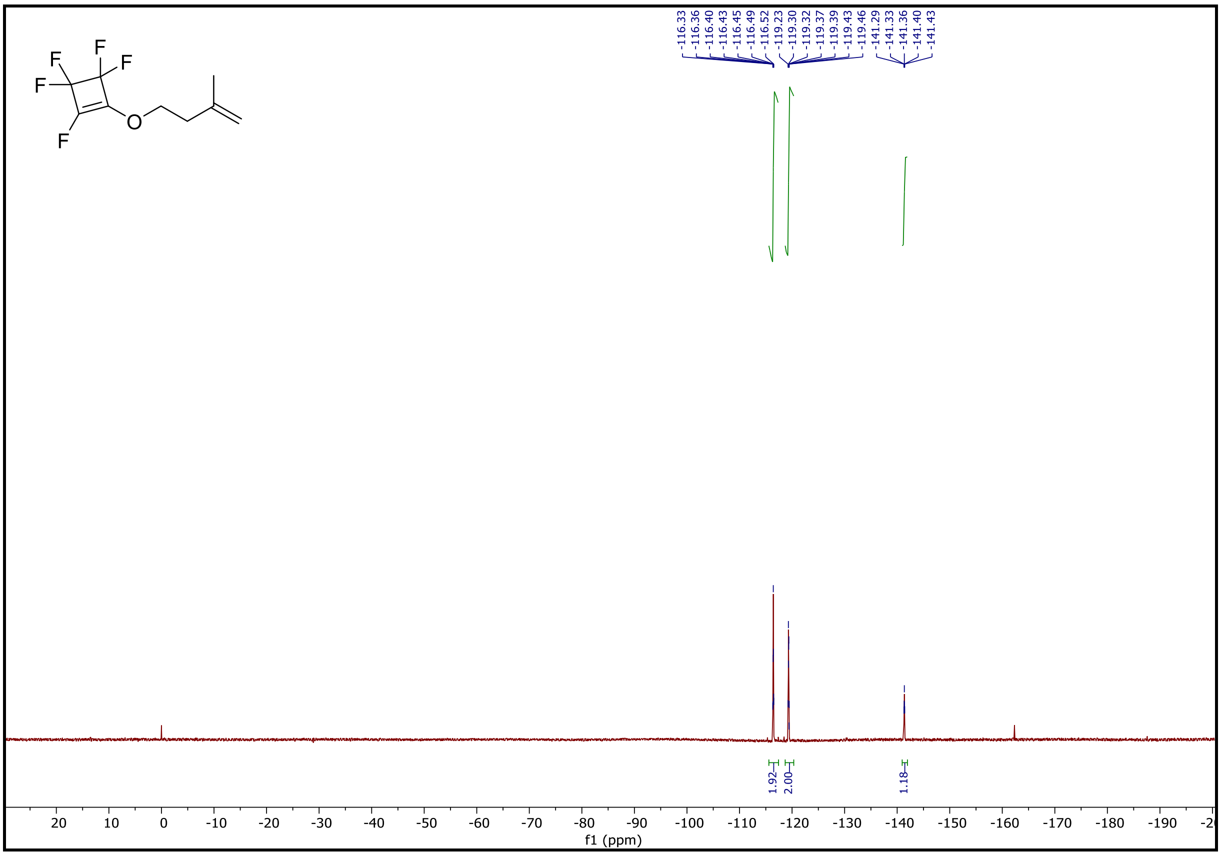

Figure S52: ^{19}F NMR spectrum of pentafluorocyclobutene **2g** ($CDCl_3$, 282 MHz)

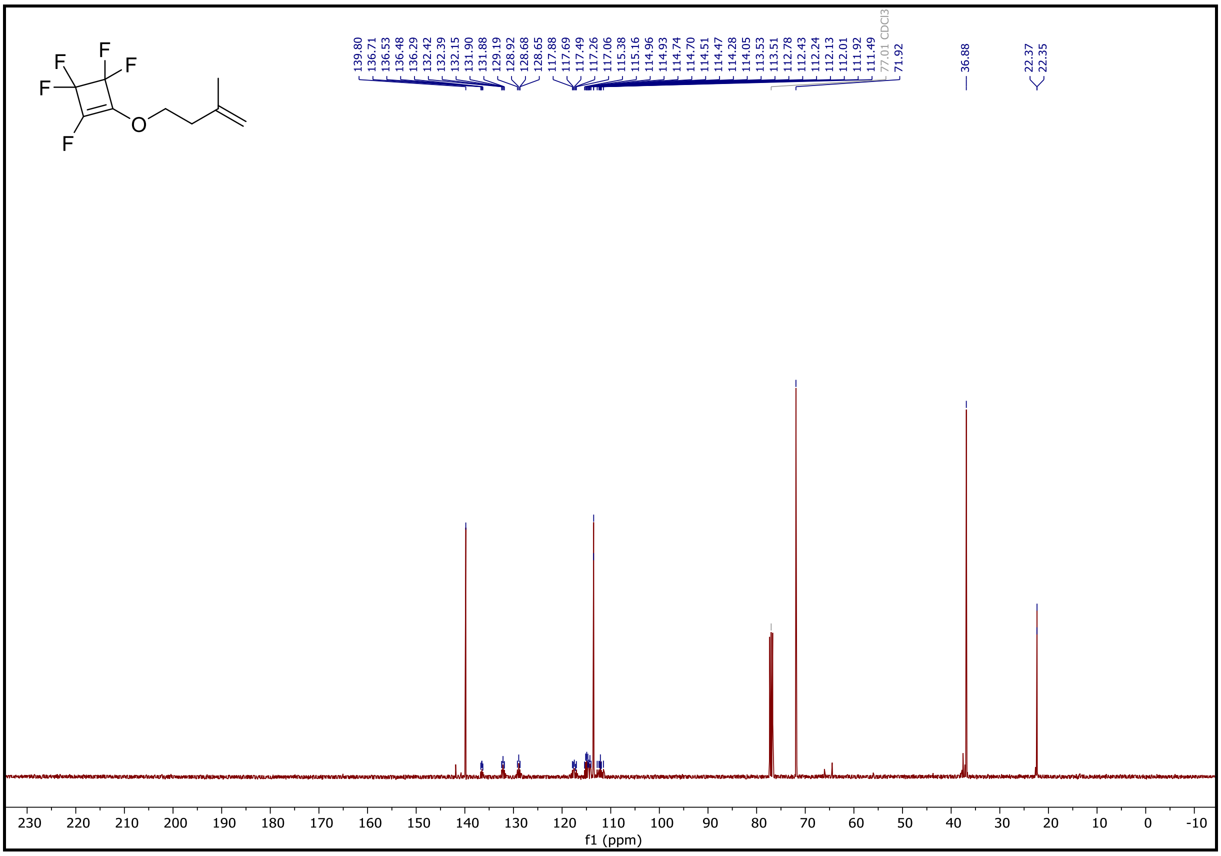

Figure S53: ^{13}C NMR spectrum of pentafluorocyclobutene **2g** ($CDCl_3$, 101 MHz)

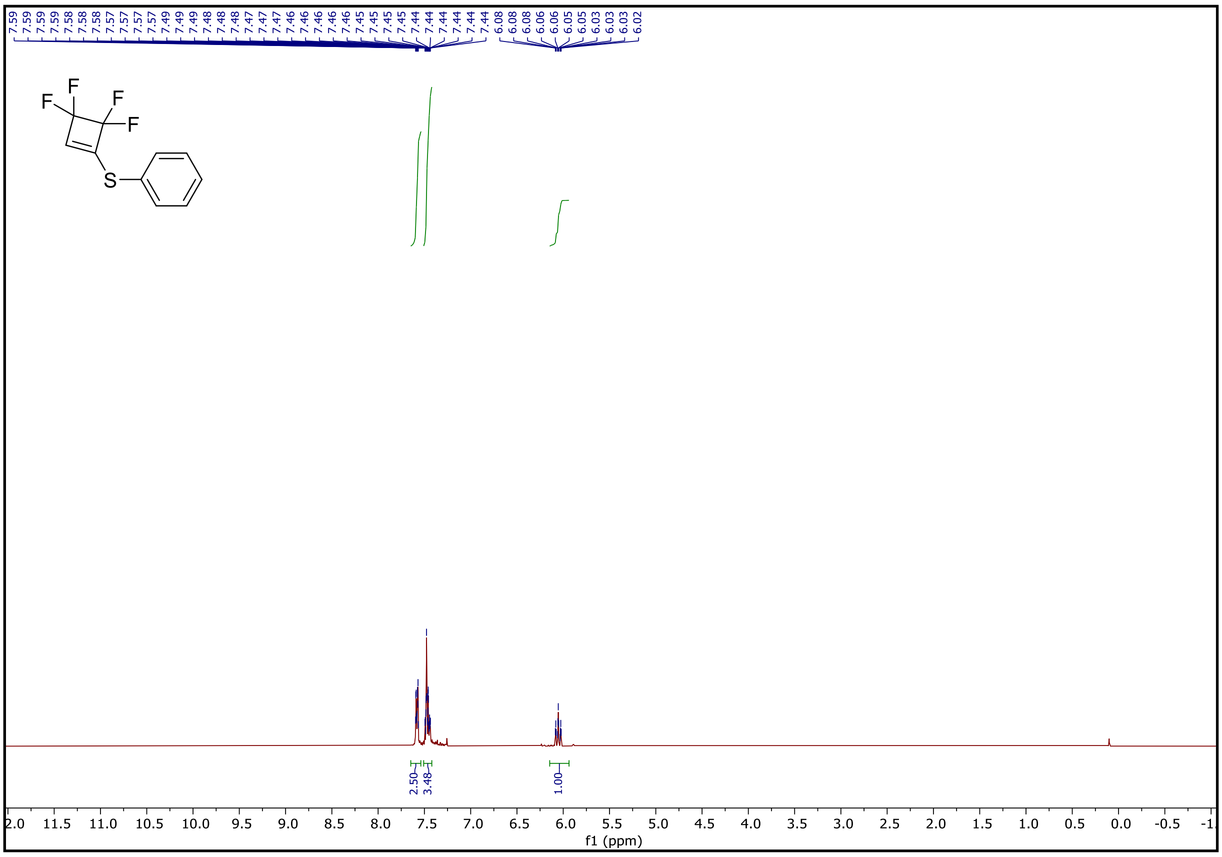

Figure S54: ^{1}H NMR spectrum of tetrafluorocyclobutene **9a** ($CDCl_3$, 400 MHz)

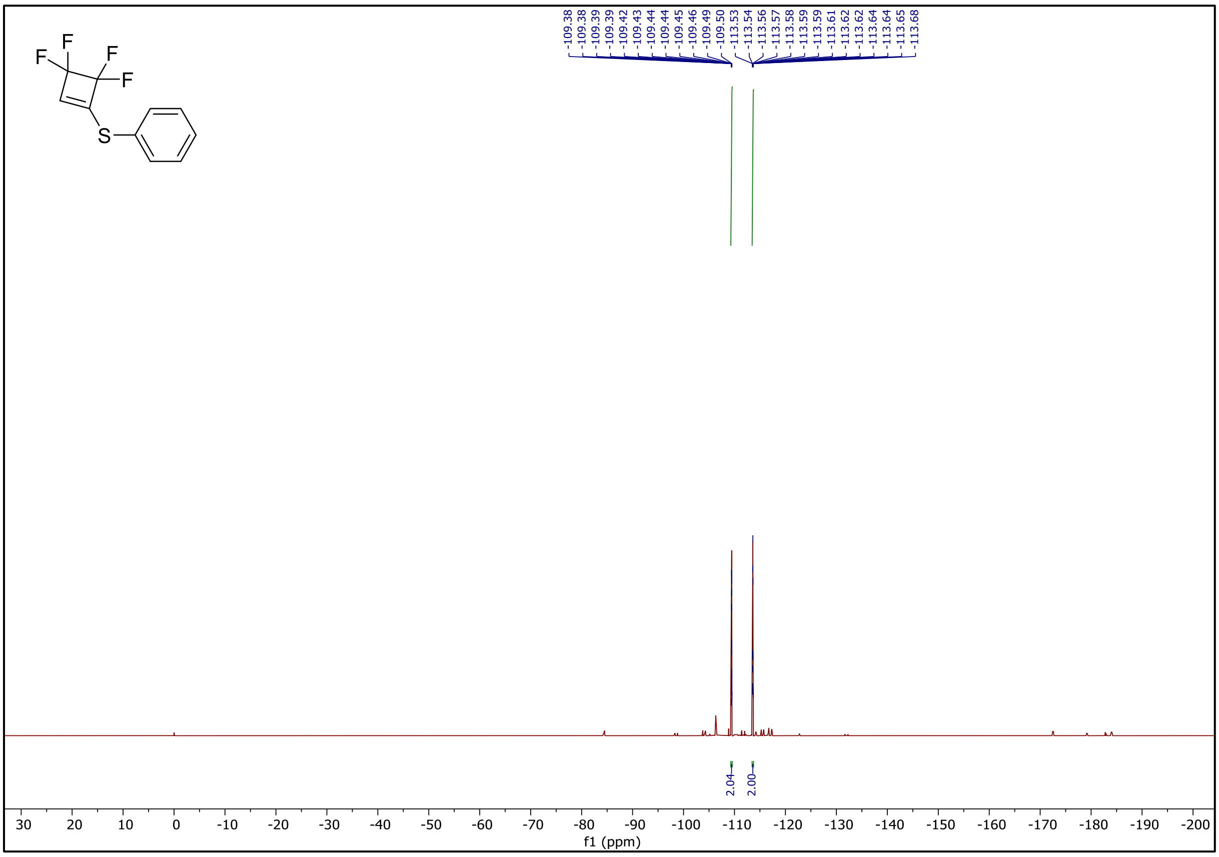

Figure S55: ^{19}F NMR spectrum of tetrafluorocyclobutene **9a** ($CDCl_3$, 282 MHz)

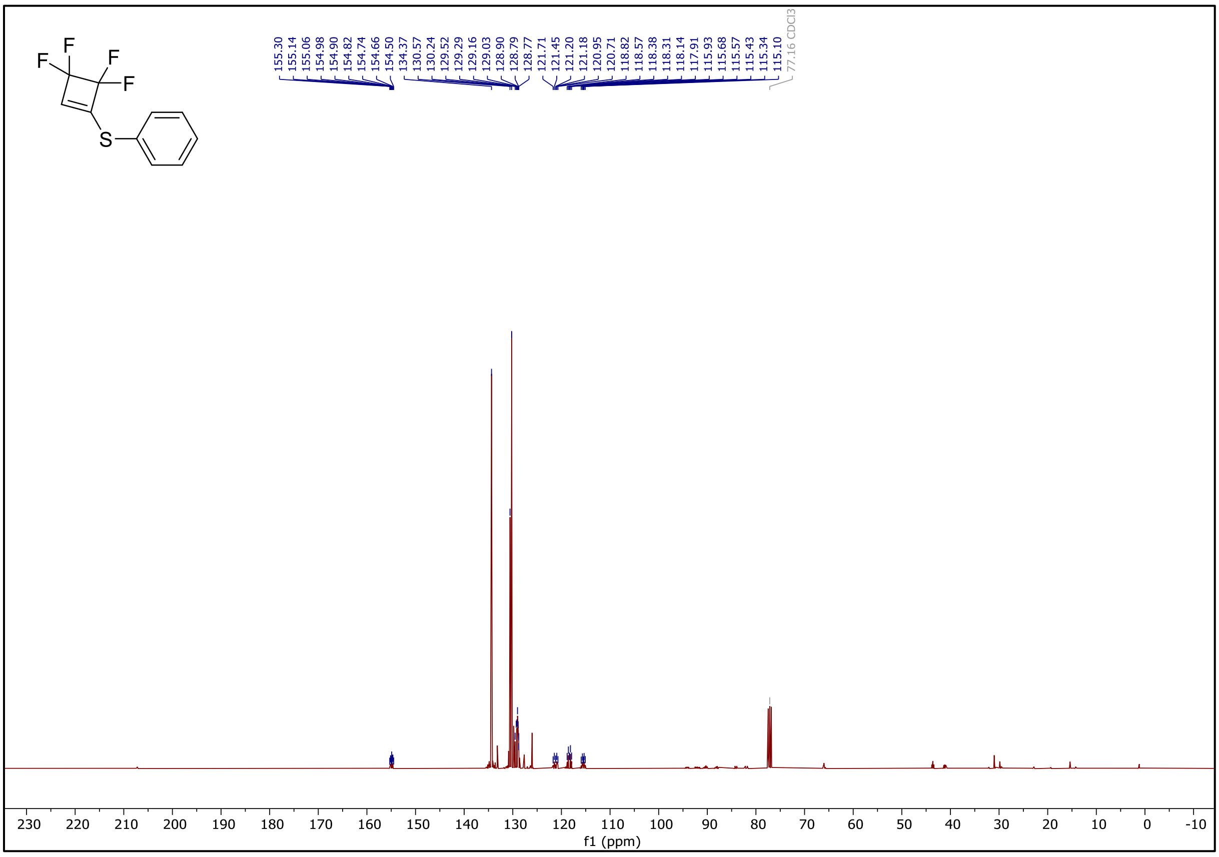

Figure S56: ^{13}C NMR spectrum of tetrafluorocyclobutene **9a** ($CDCl_3$, 101 MHz)

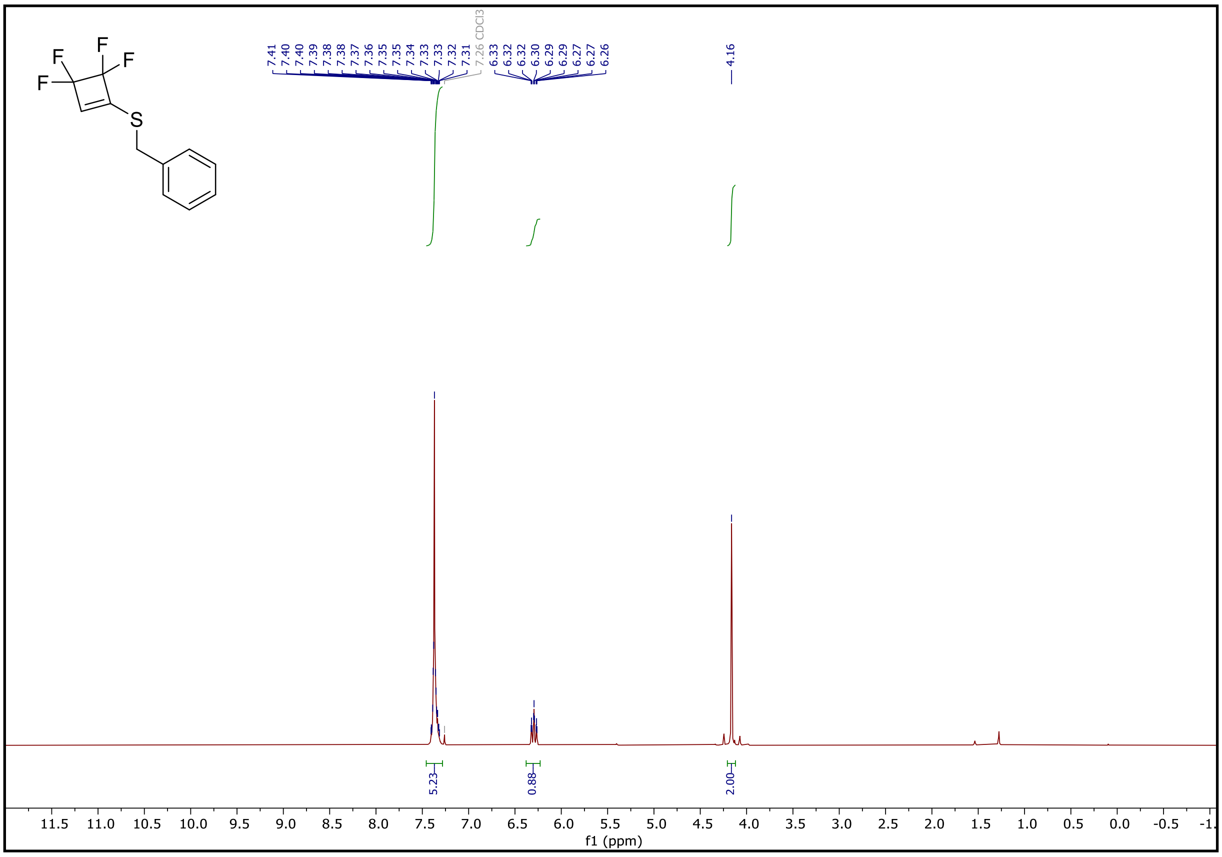

Figure S57: ^{1}H NMR spectrum of tetrafluorocyclobutene **9b** ($CDCl_3$, 400 MHz)

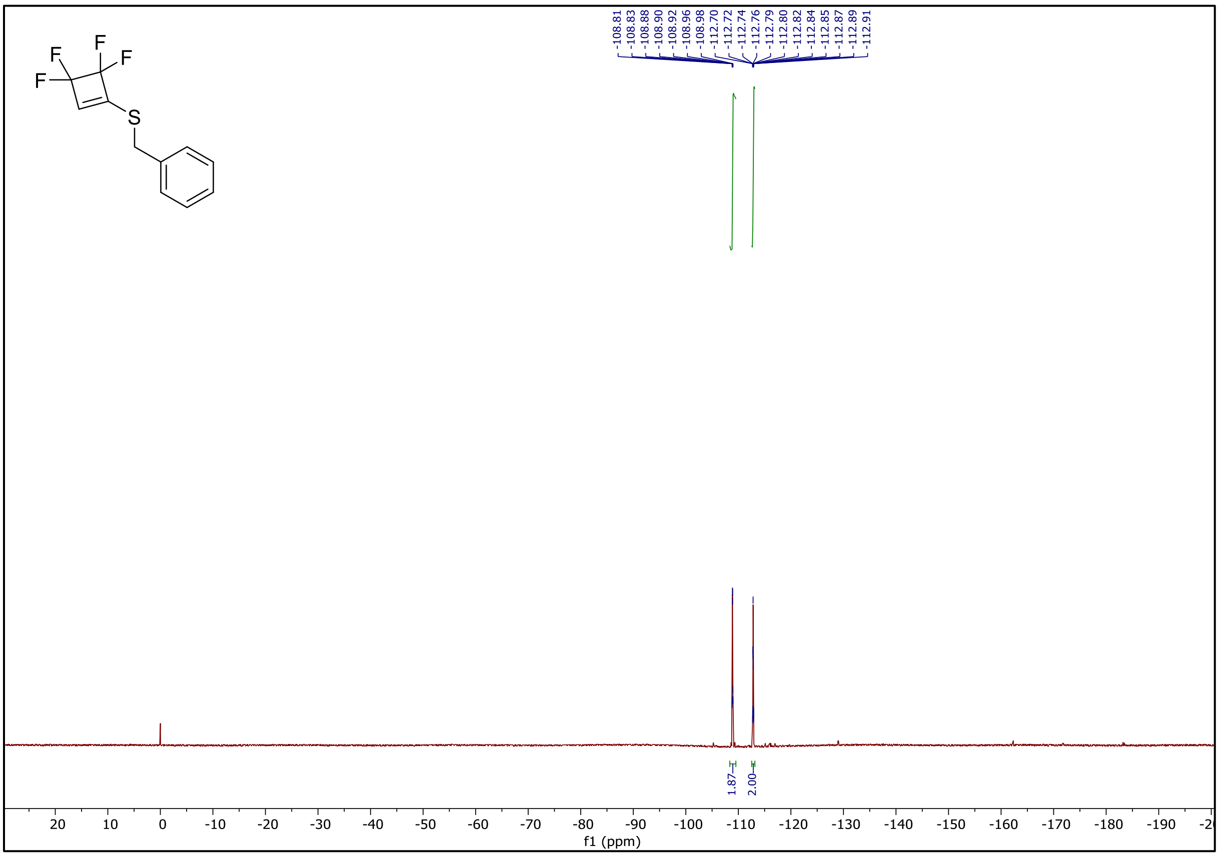

Figure S58: ^{19}F NMR spectrum of tetrafluorocyclobutene **9b** ($CDCl_3$, 282 MHz)

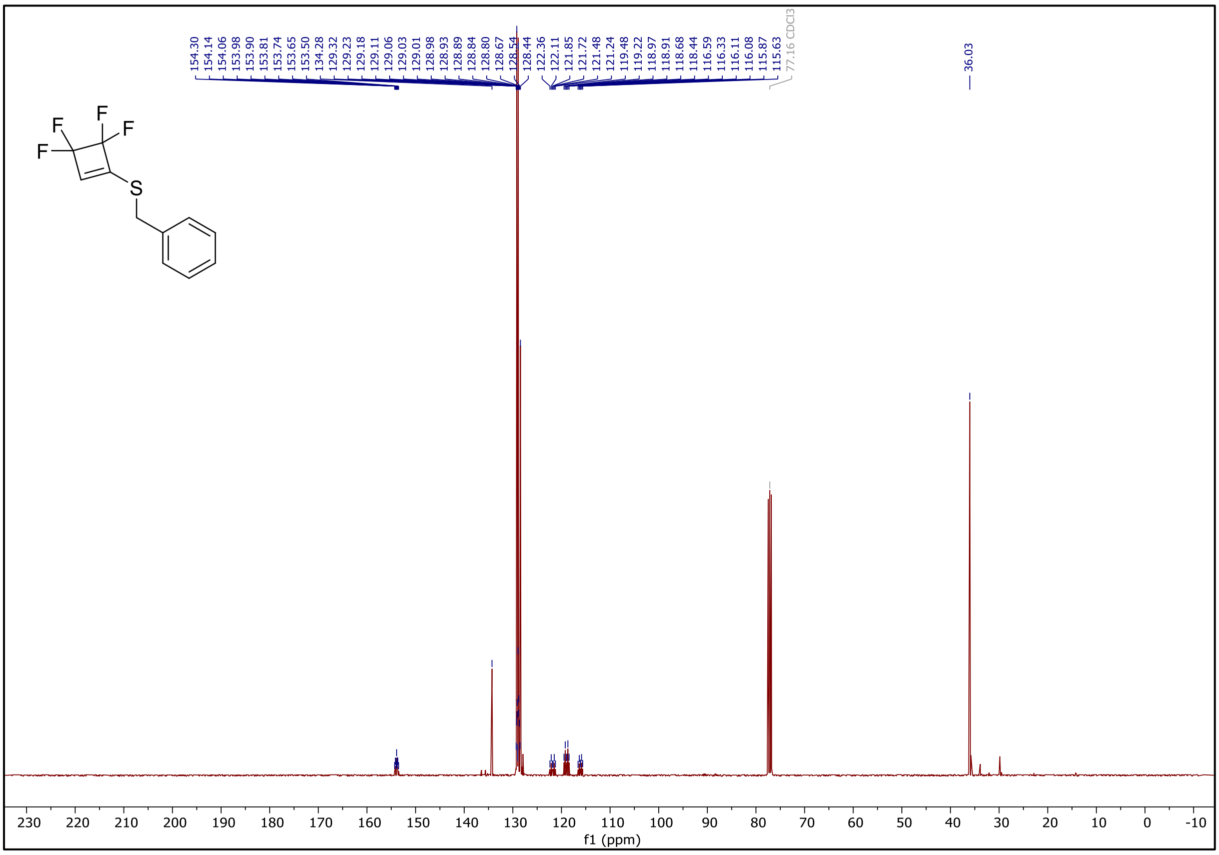

Figure S59: ^{13}C NMR spectrum of tetrafluorocyclobutene **9b** ($CDCl_3$, 101 MHz)

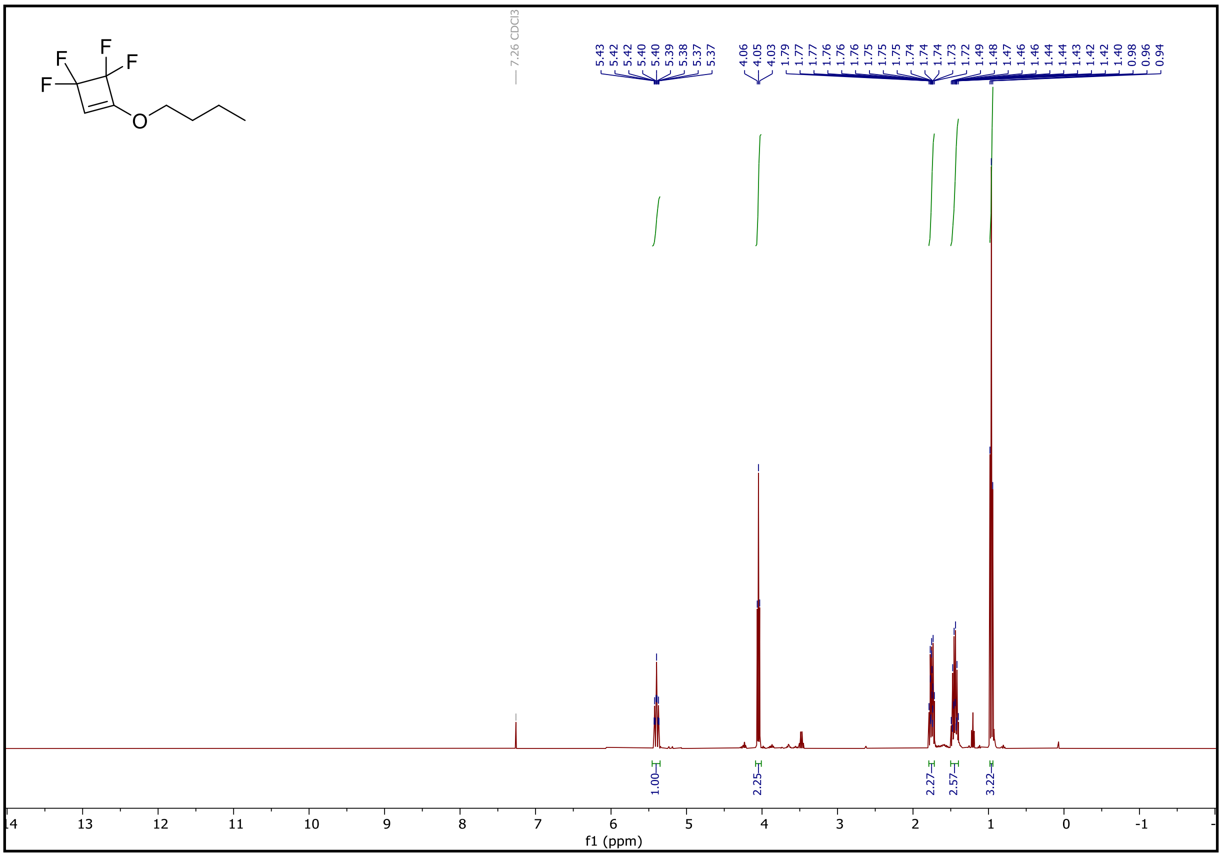

Figure S60: ^{1}H NMR spectrum of tetrafluorocyclobutene **10a** ($CDCl_3$, 400 MHz)

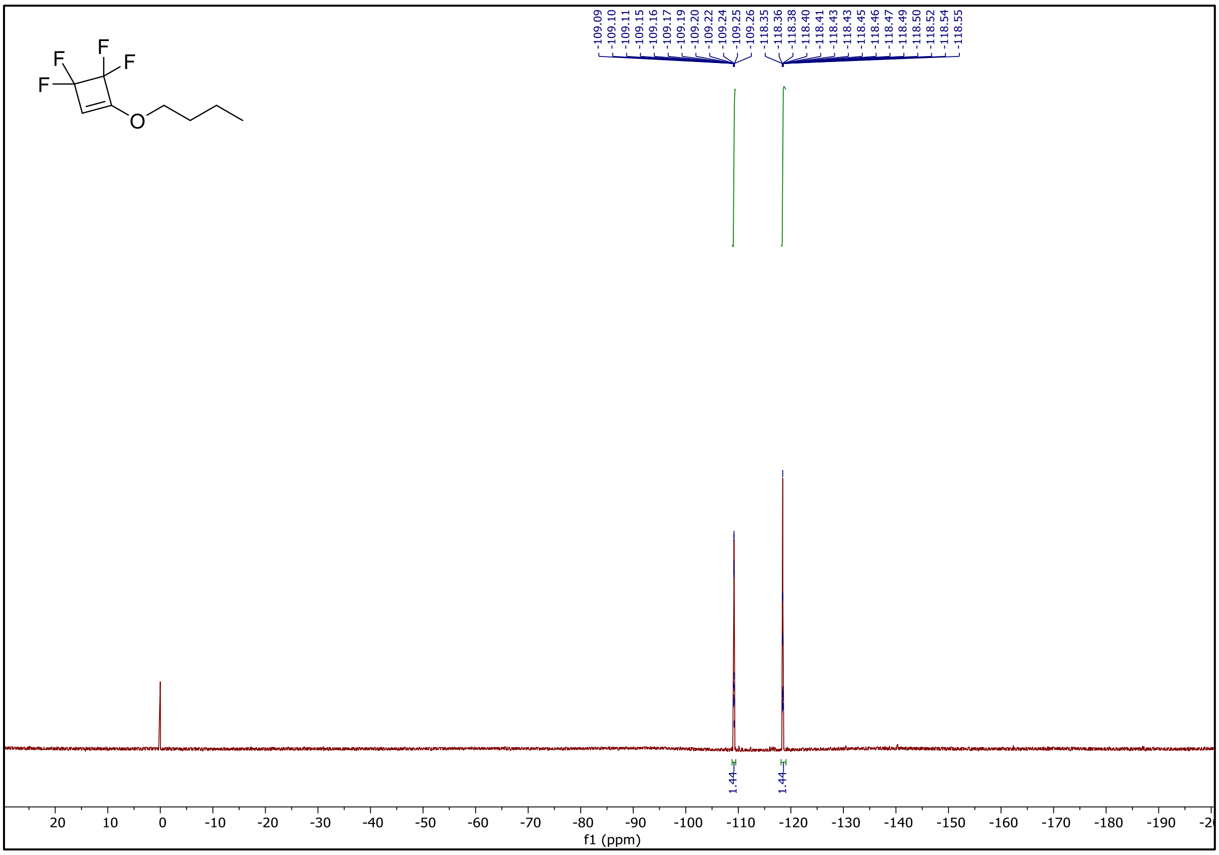

Figure S61: ^{19}F NMR spectrum of tetrafluorocyclobutene **10a** ($CDCl_3$, 282 MHz)

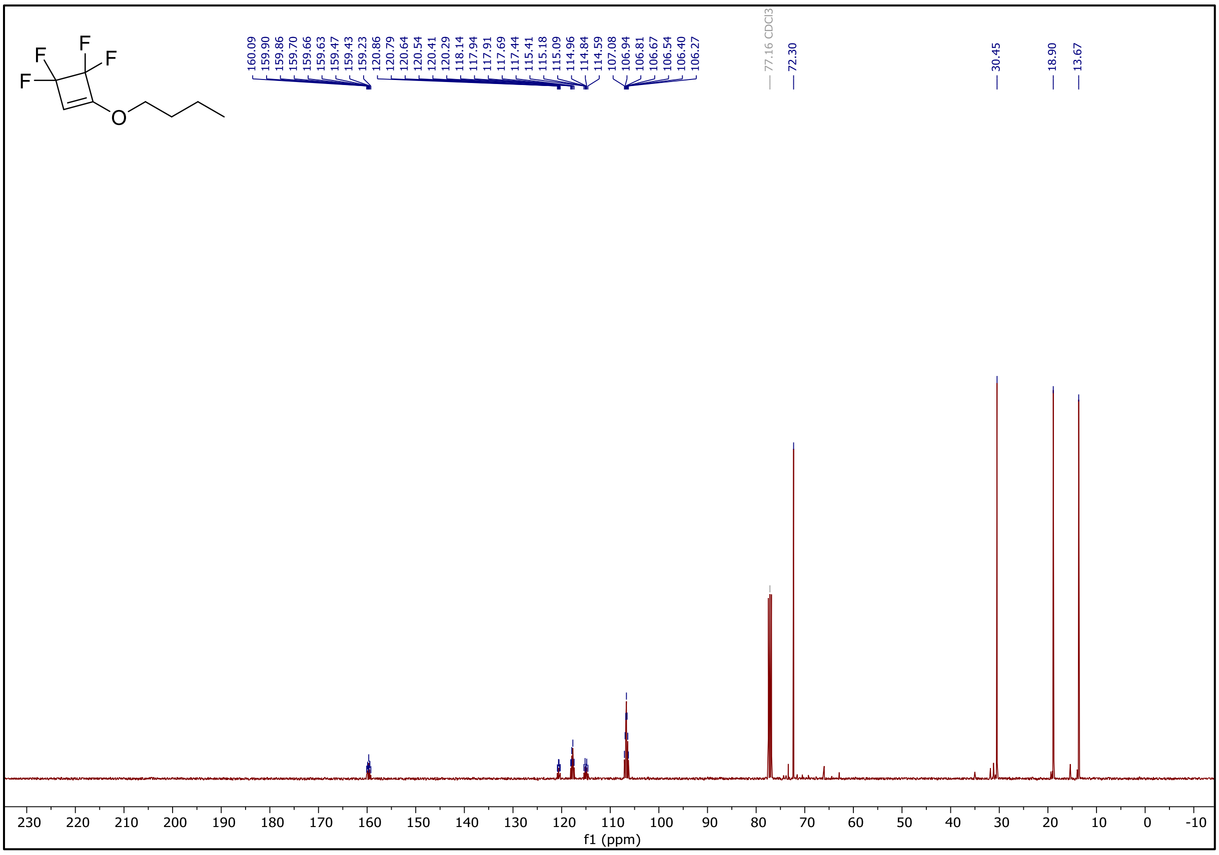

Figure S62: ^{13}C NMR spectrum of tetrafluorocyclobutene **10a** ($CDCl_3$, 101 MHz)

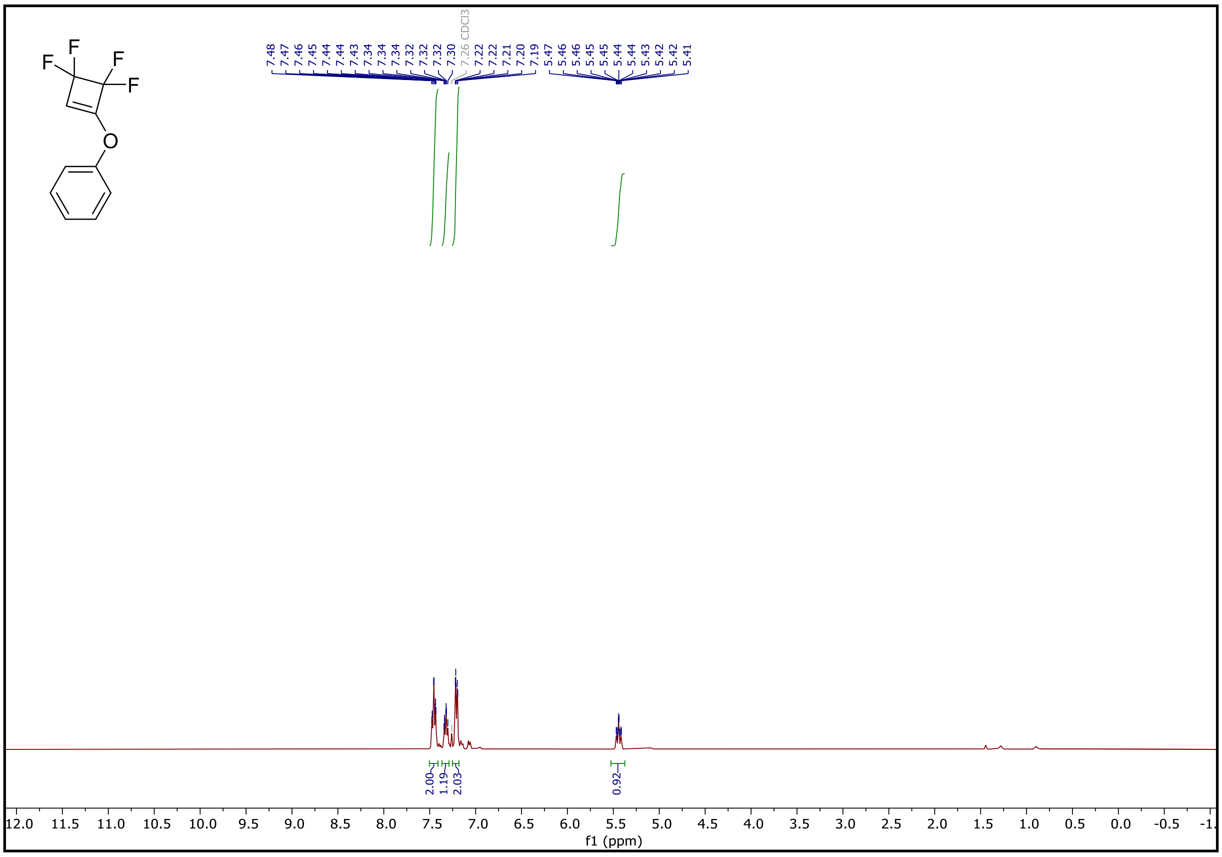

Figure S63: ^{1}H NMR spectrum of tetrafluorocyclobutene **10b** ($CDCl_3$, 400 MHz)

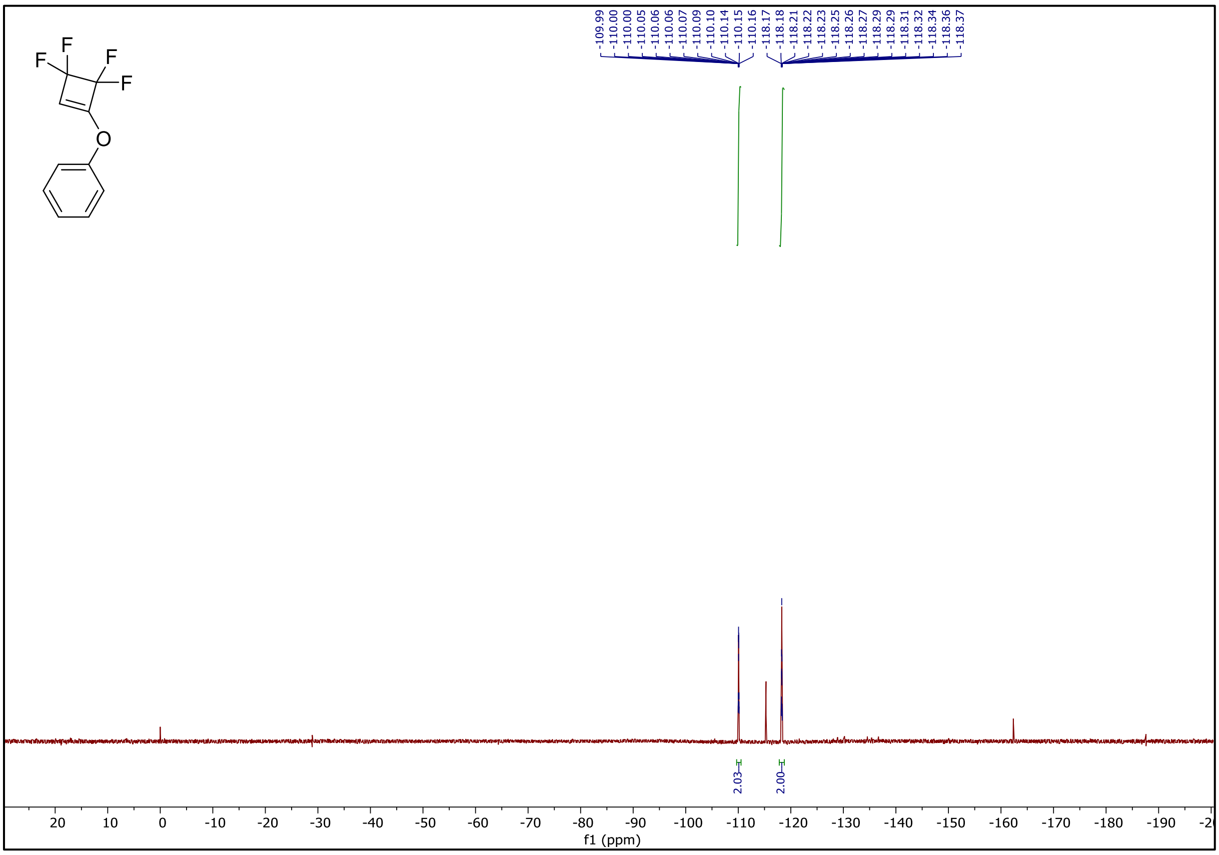

Figure S64: ^{19}F NMR spectrum of tetrafluorocyclobutene **10b** ($CDCl_3$, 282 MHz)

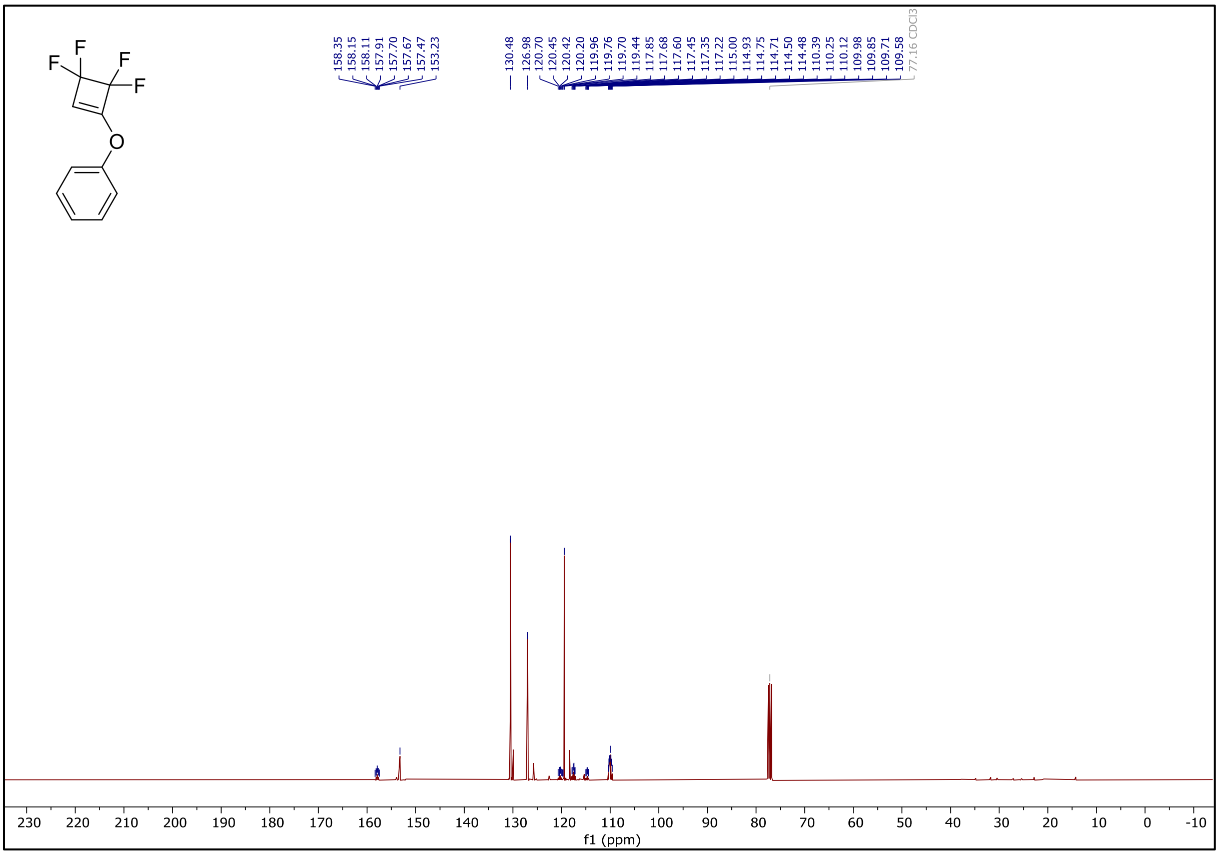

Figure S65: ^{13}C NMR spectrum of tetrafluorocyclobutene **10b** ($CDCl_3$, 101 MHz)

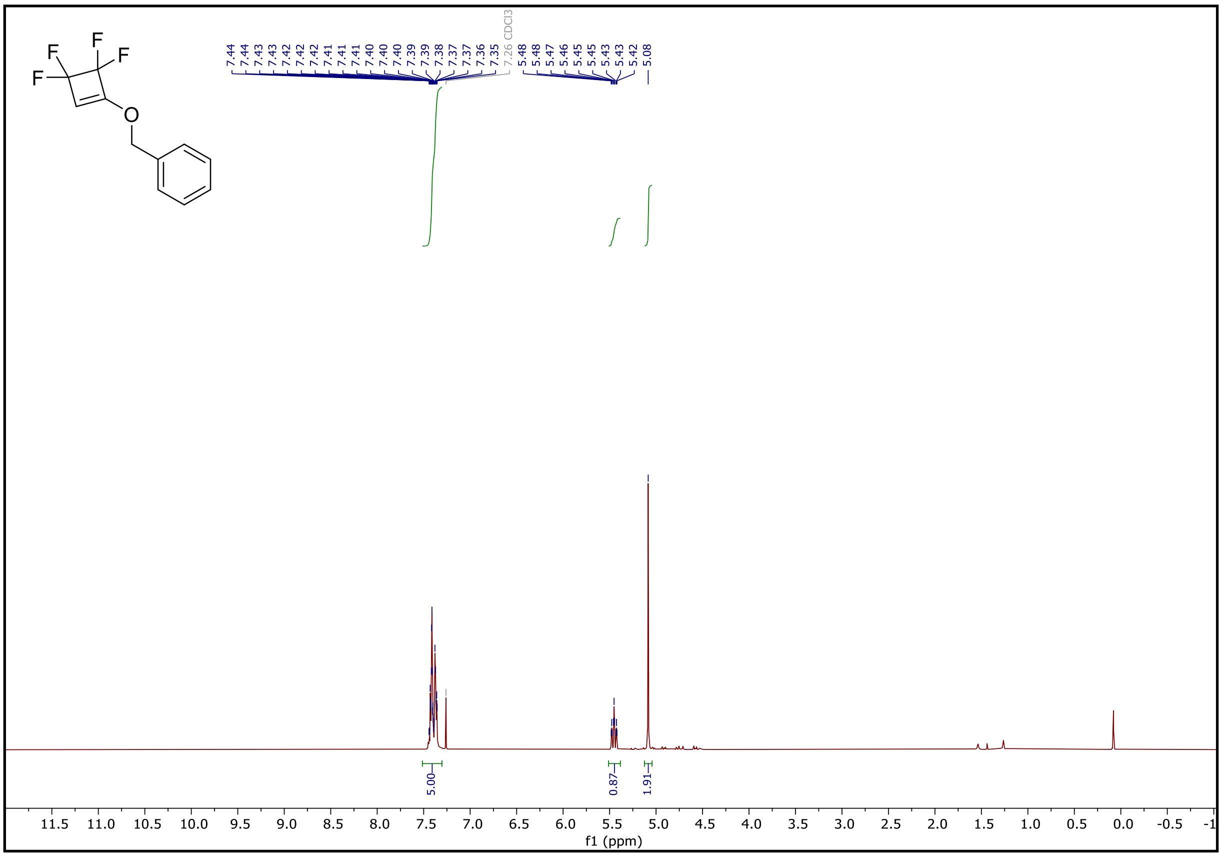

Figure S66: ^{1}H NMR spectrum of tetrafluorocyclobutene **10c** ($CDCl_3$, 400 MHz)

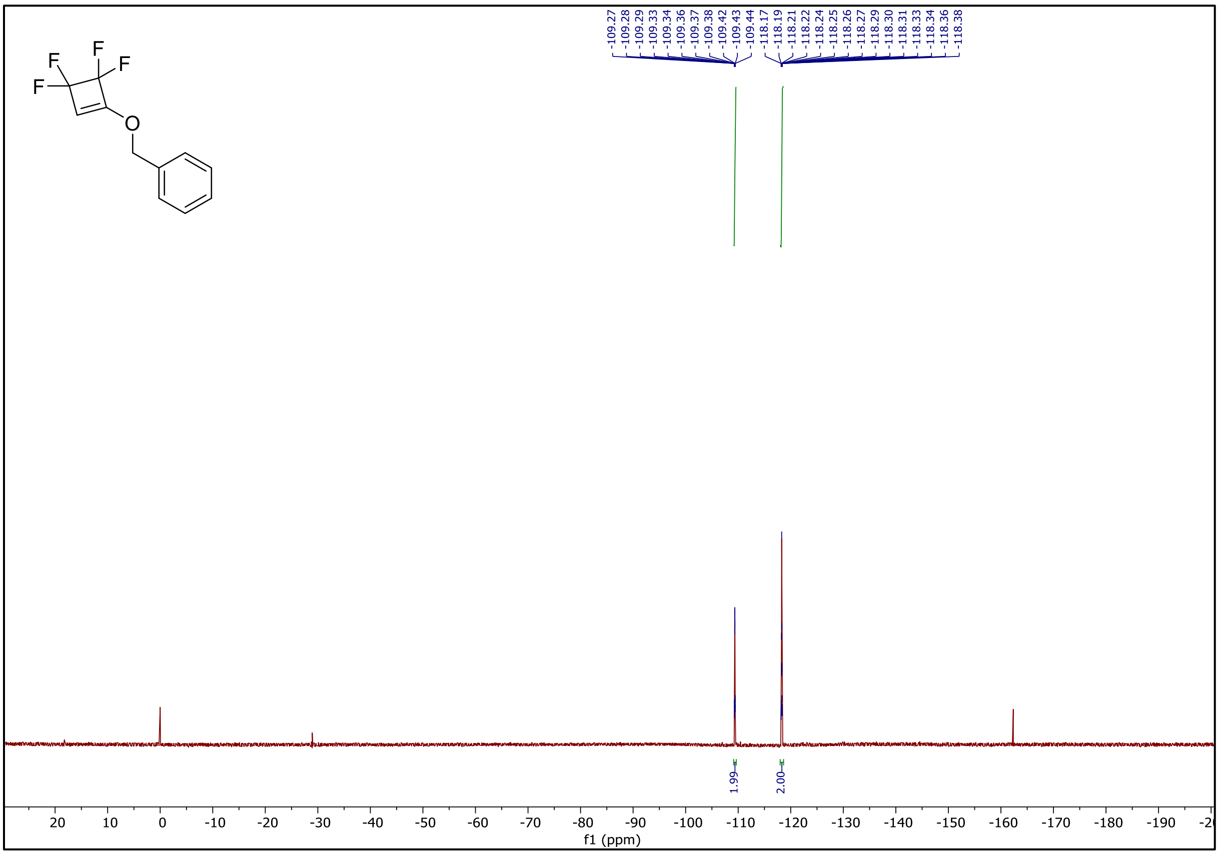

Figure S67: ^{19}F NMR spectrum of tetrafluorocyclobutene **10c** ($CDCl_3$, 282 MHz)

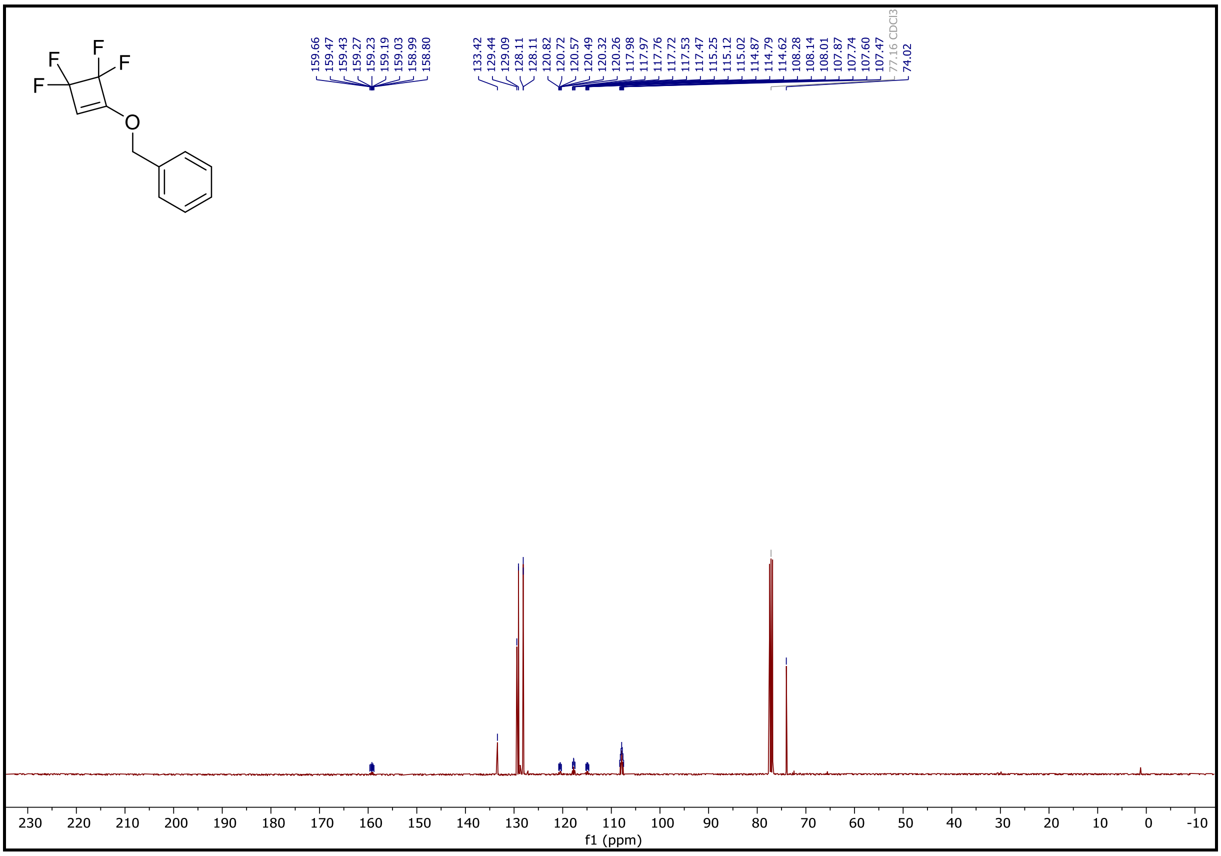

Figure S68: ^{13}C NMR spectrum of tetrafluorocyclobutene **10c** ($CDCl_3$, 101 MHz)

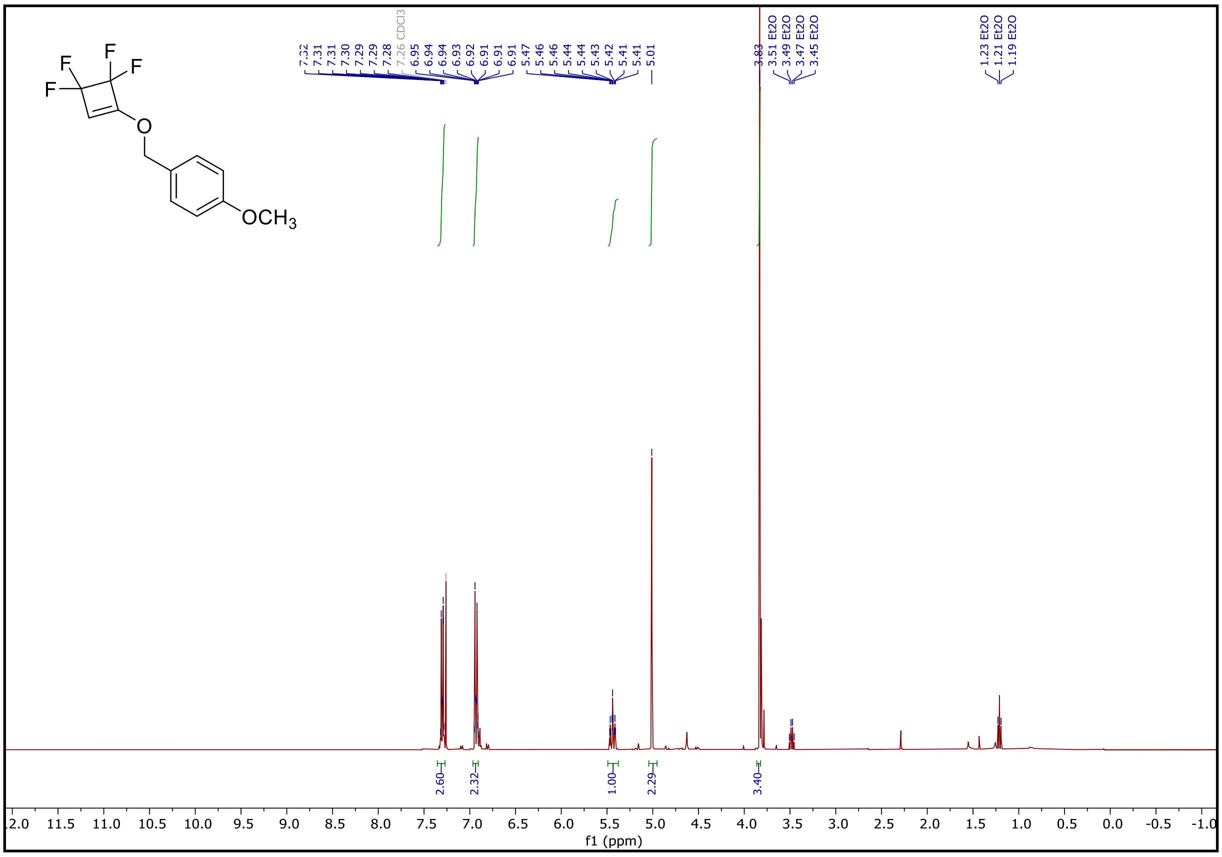

Figure S69: ^{1}H NMR spectrum of tetrafluorocyclobutene **10d** ($CDCl_3$, 400 MHz)

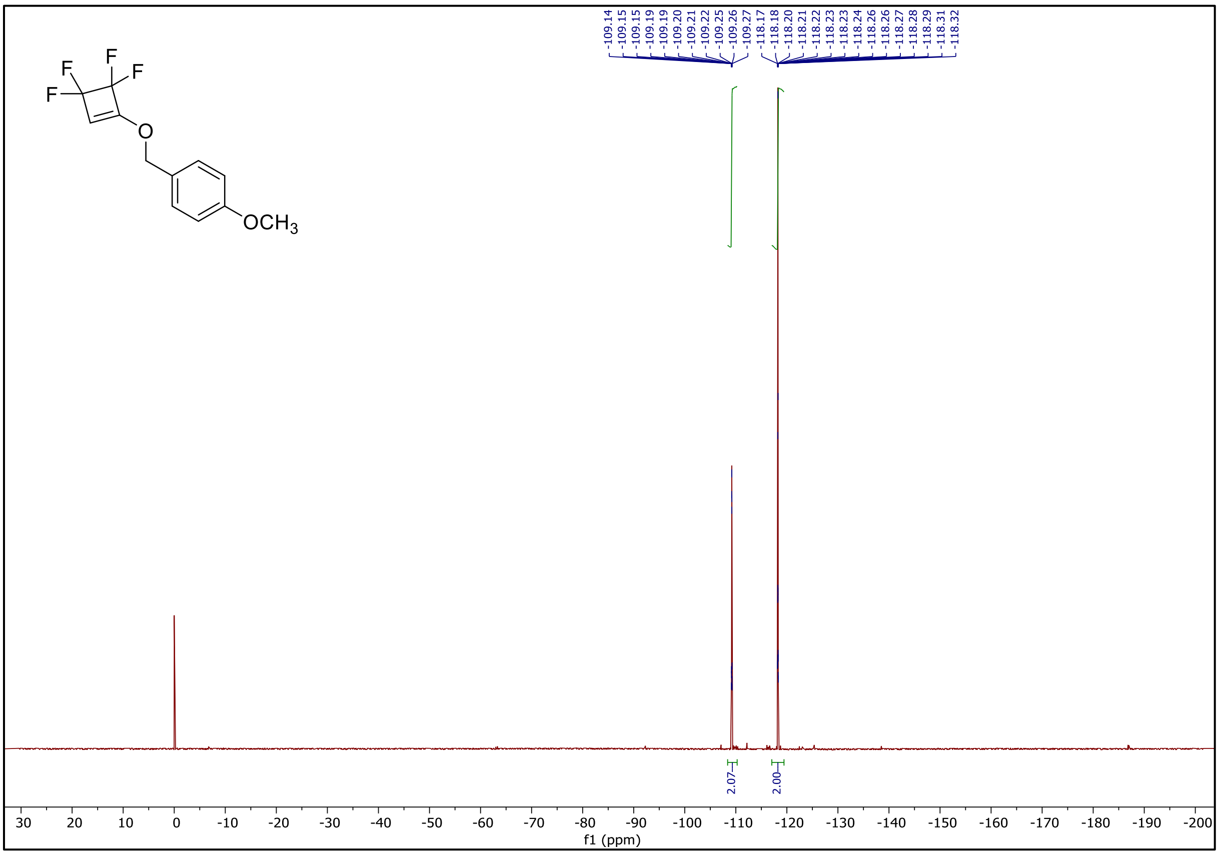

Figure S70: ^{19}F NMR spectrum of tetrafluorocyclobutene **10d** ($CDCl_3$, 376 MHz)

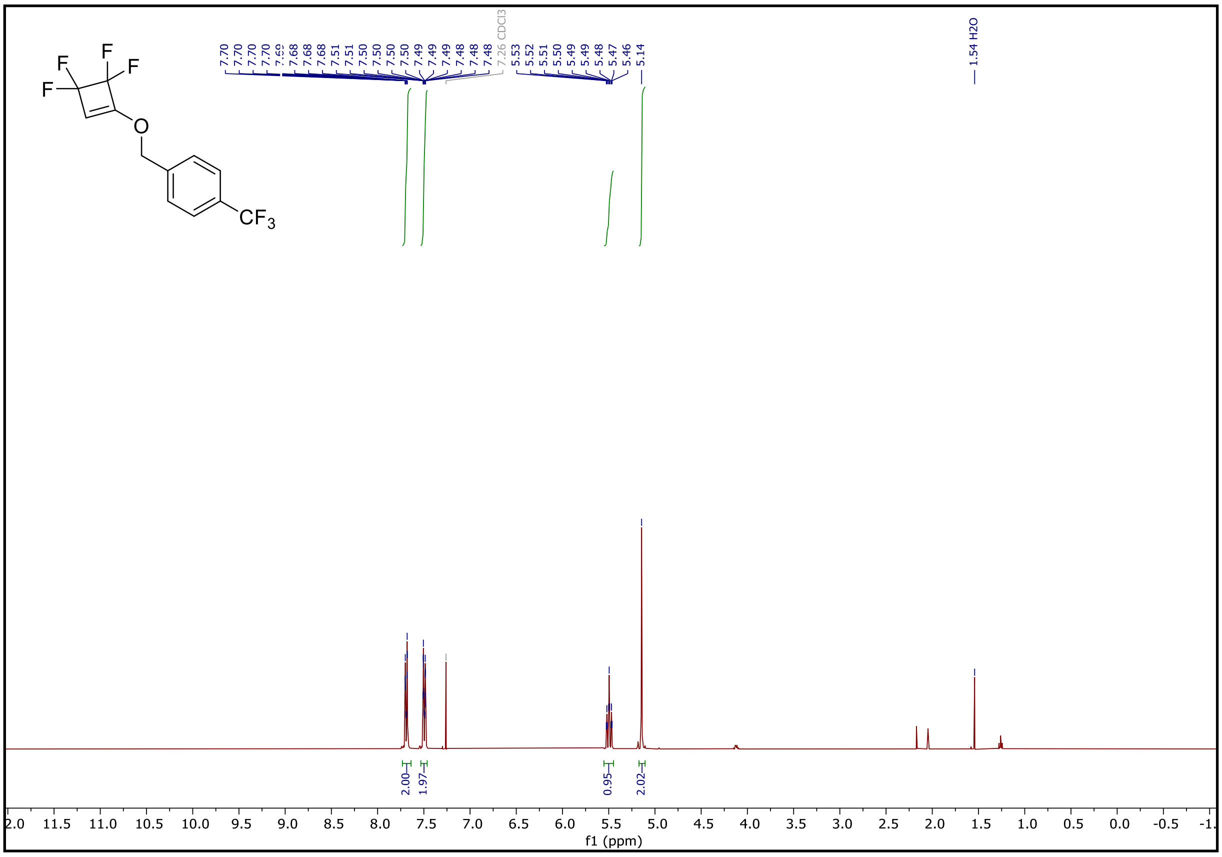

Figure S71: ^{1}H NMR spectrum of tetrafluorocyclobutene **10e** ($CDCl_3$, 400 MHz)

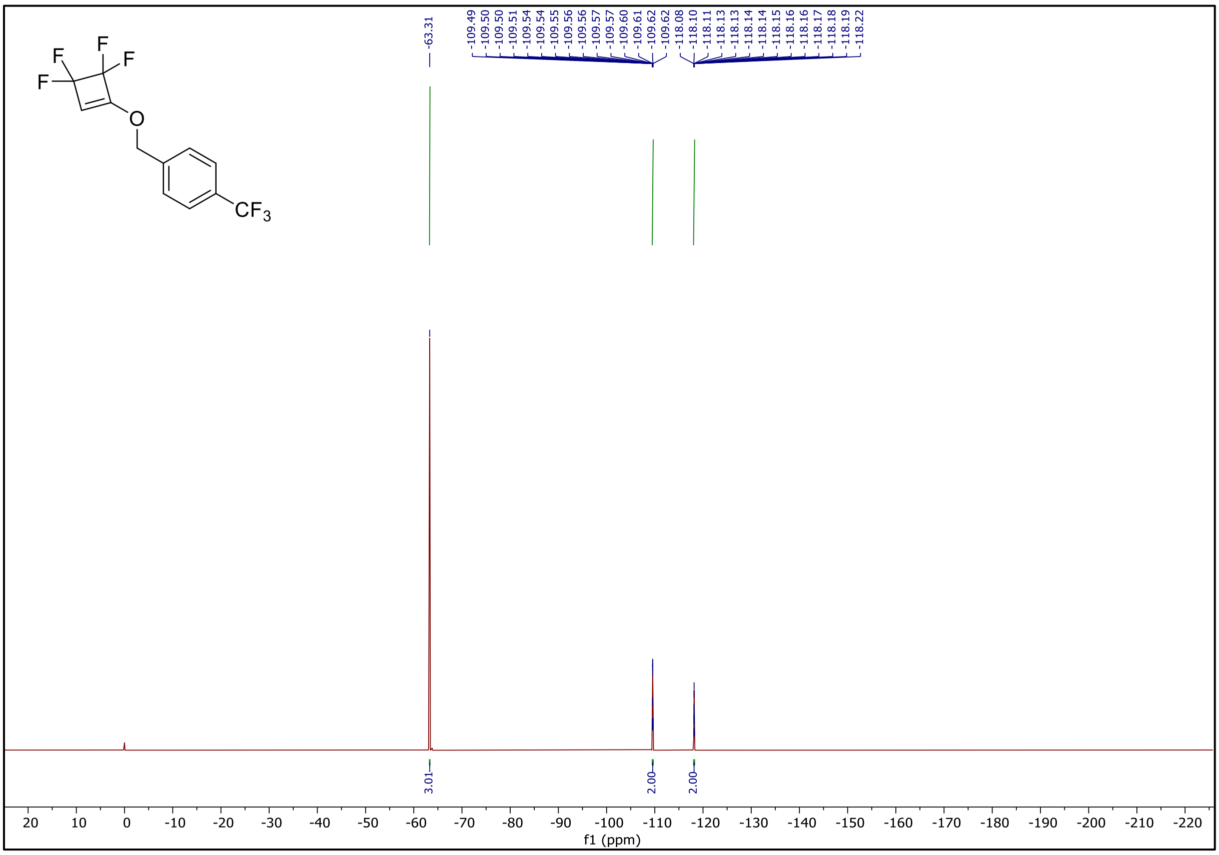

Figure S72: ^{19}F NMR spectrum of tetrafluorocyclobutene **10e** ($CDCl_3$, 376 MHz)

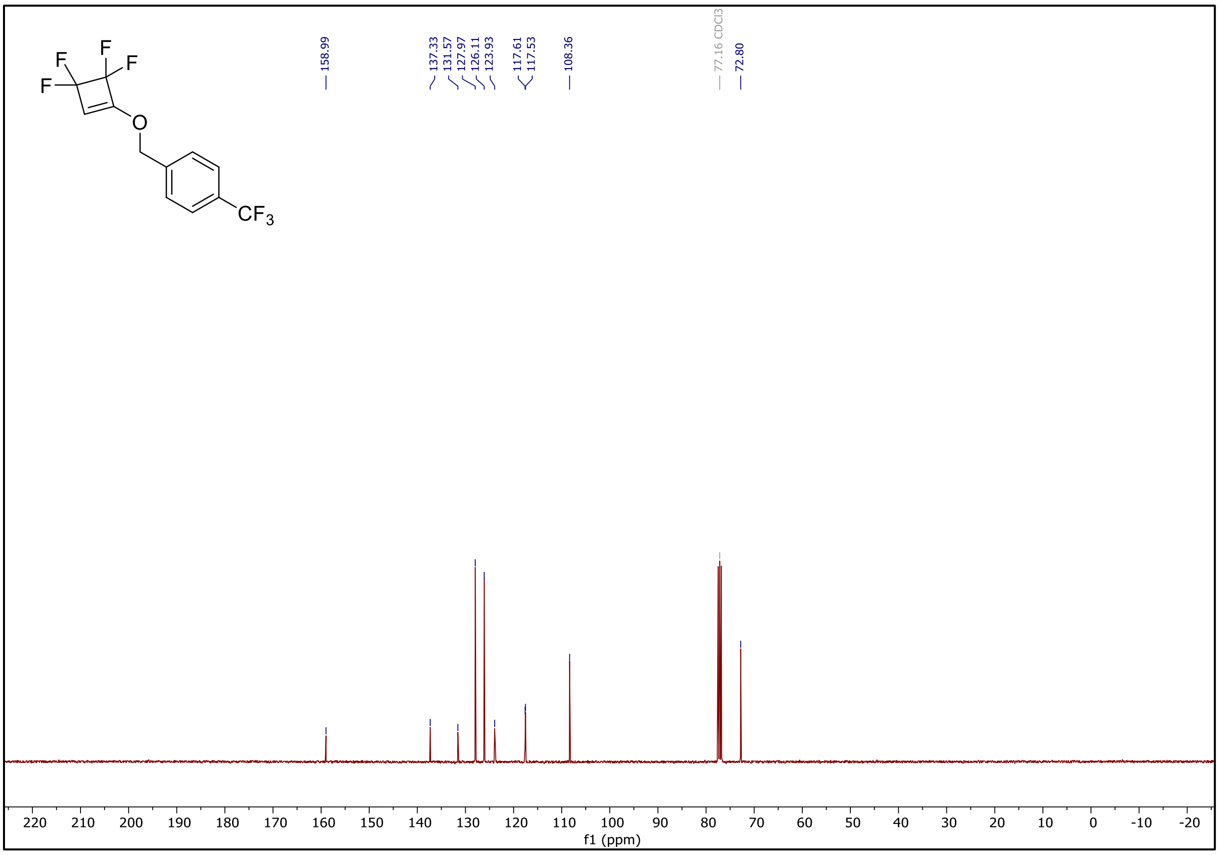

Figure S73: ^{13}C NMR spectrum of tetrafluorocyclobutene **10e** ($CDCl_3$, 101 MHz, ^{1}H and ^{19}F decoupling)

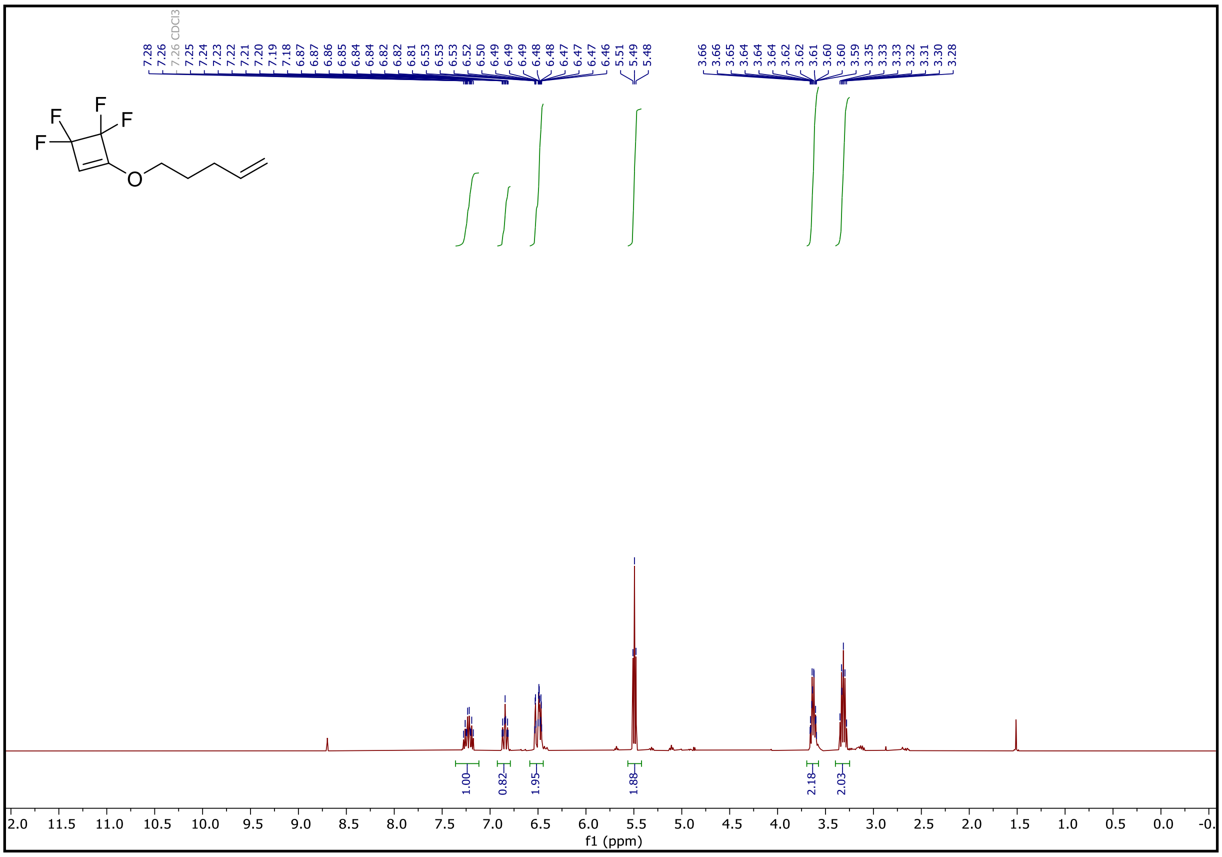

Figure S74: ^{1}H NMR spectrum of tetrafluorocyclobutene **12a** ($CDCl_3$, 400 MHz)

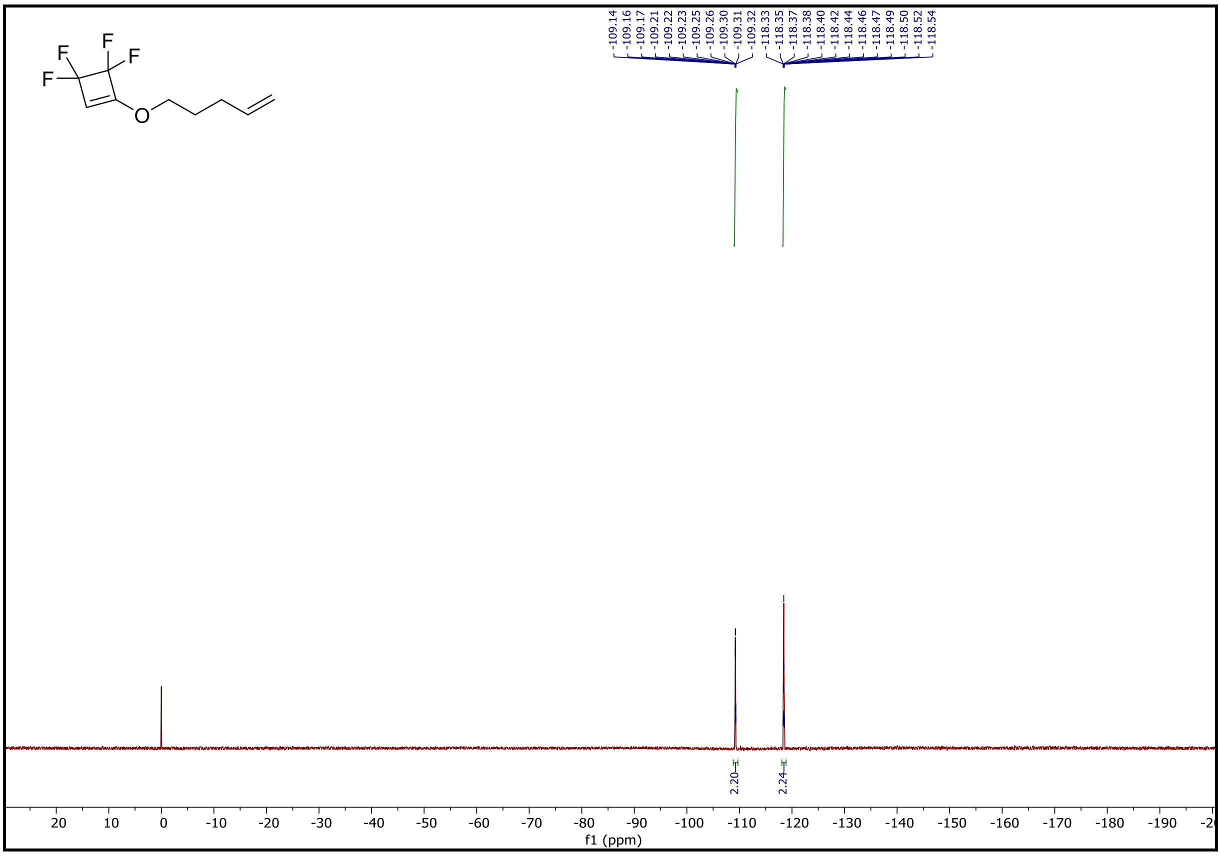

Figure S75: ^{19}F NMR spectrum of tetrafluorocyclobutene **12a** ($CDCl_3$, 282 MHz)

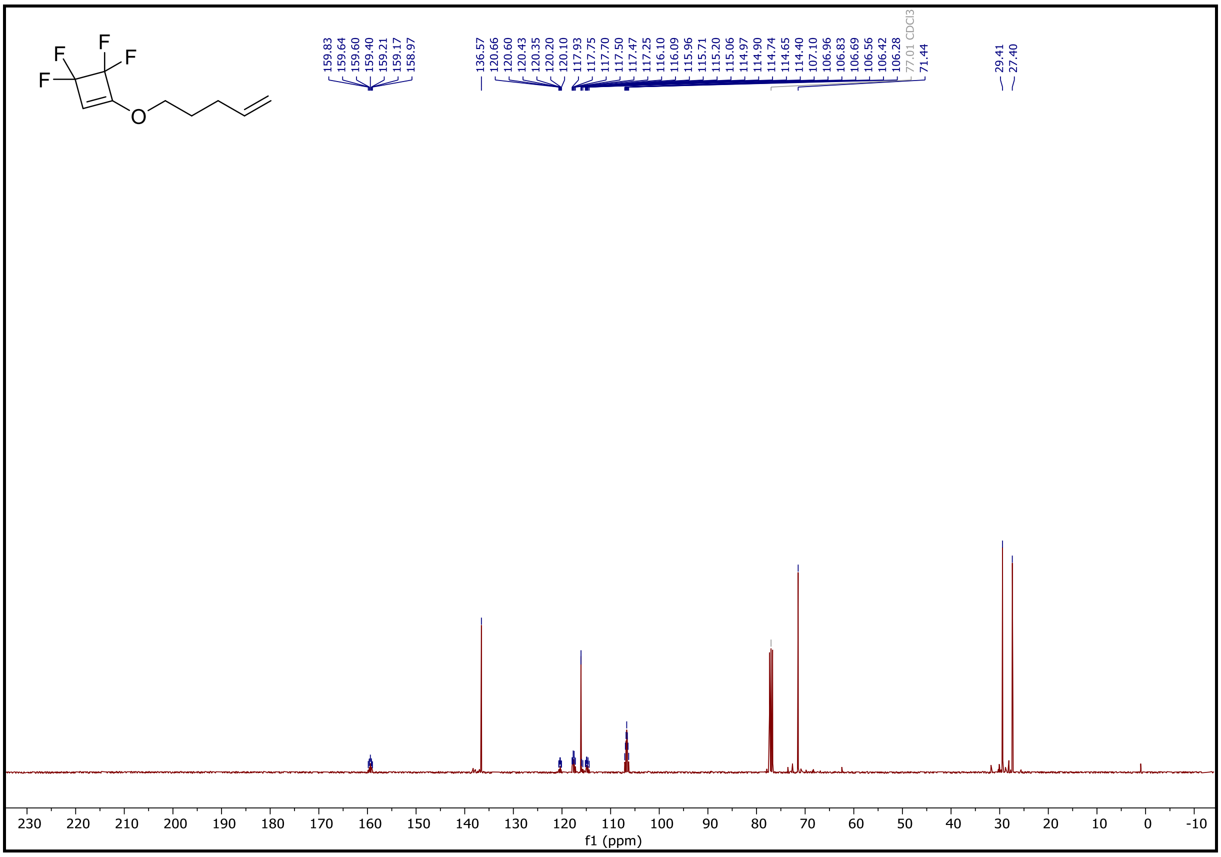

Figure S76: ^{13}C NMR spectrum of tetrafluorocyclobutene **12a** ($CDCl_3$, 101 MHz)

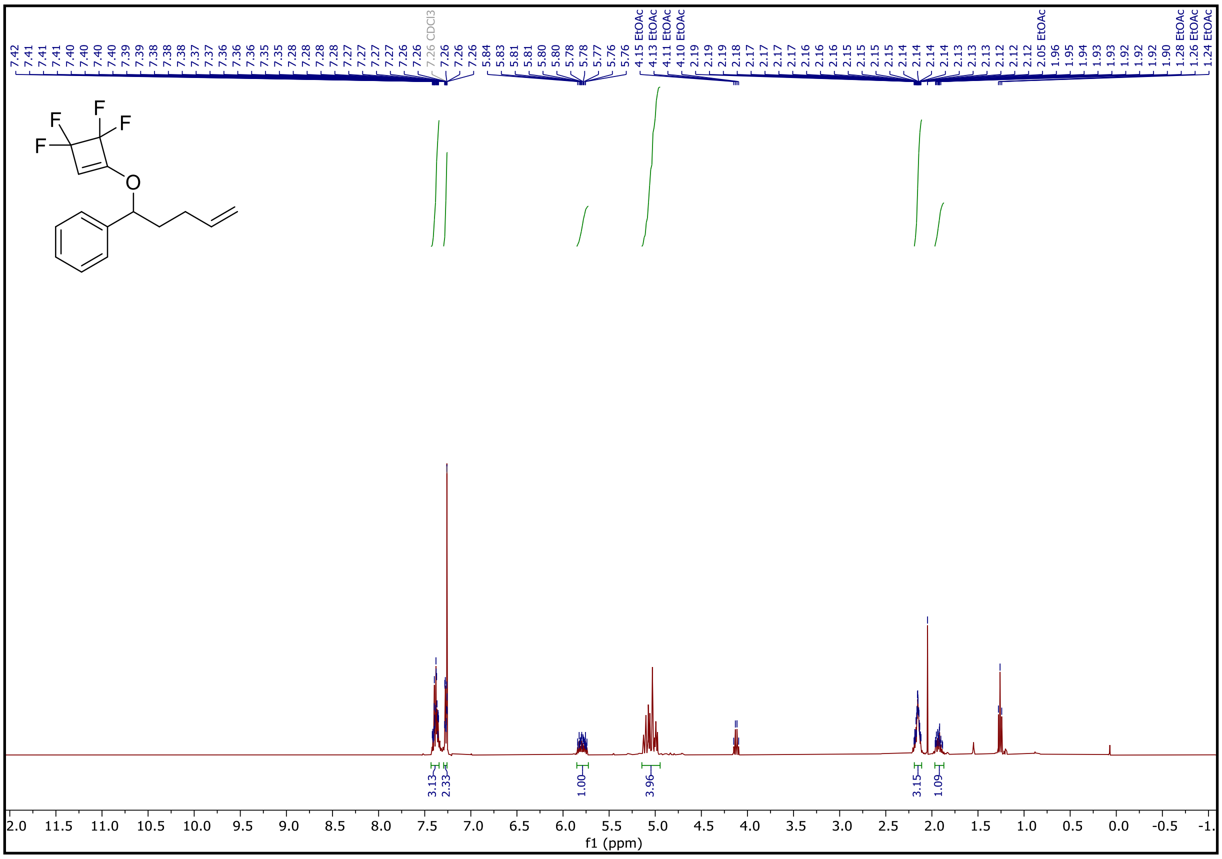

Figure S77: ^{1}H NMR spectrum of tetrafluorocyclobutene **12b** ($CDCl_3$, 400 MHz)

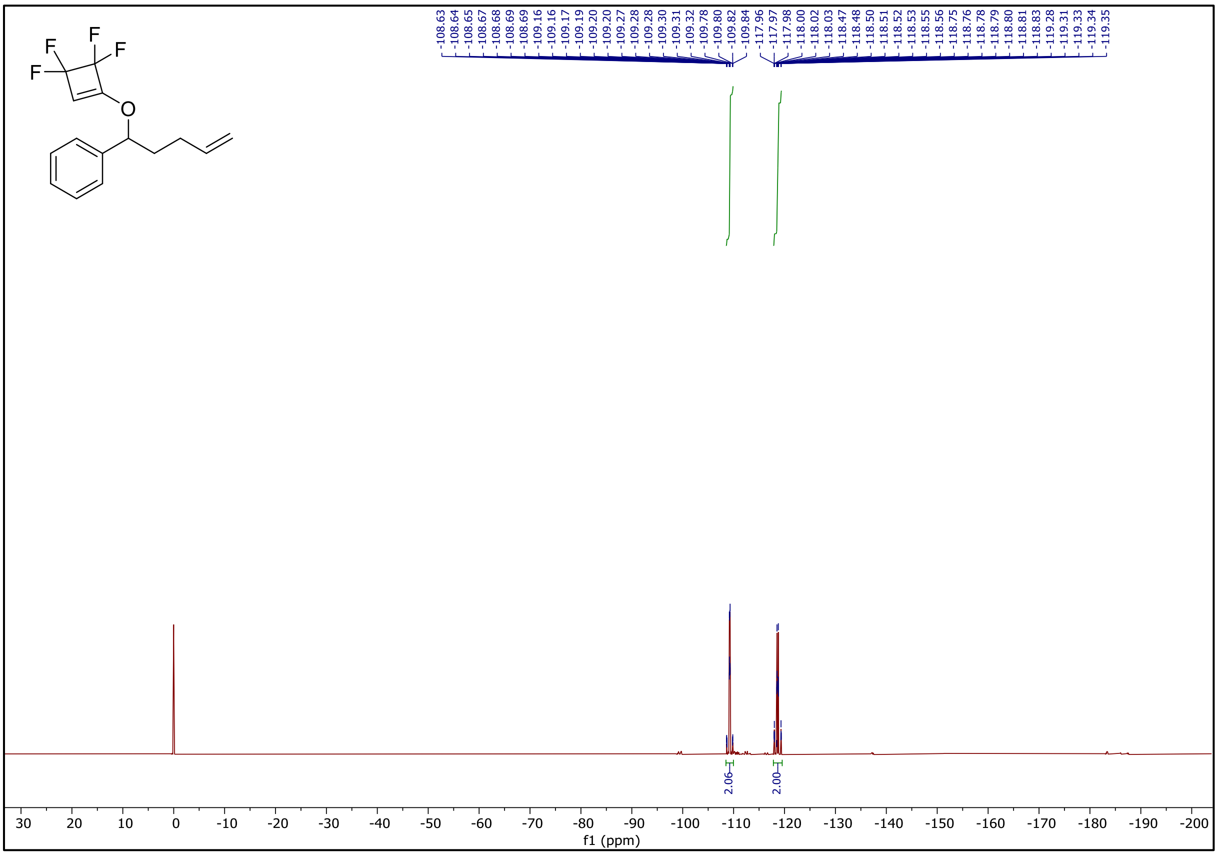

Figure S78: ^{19}F NMR spectrum of tetrafluorocyclobutene **12b** ($CDCl_3$, 376 MHz)

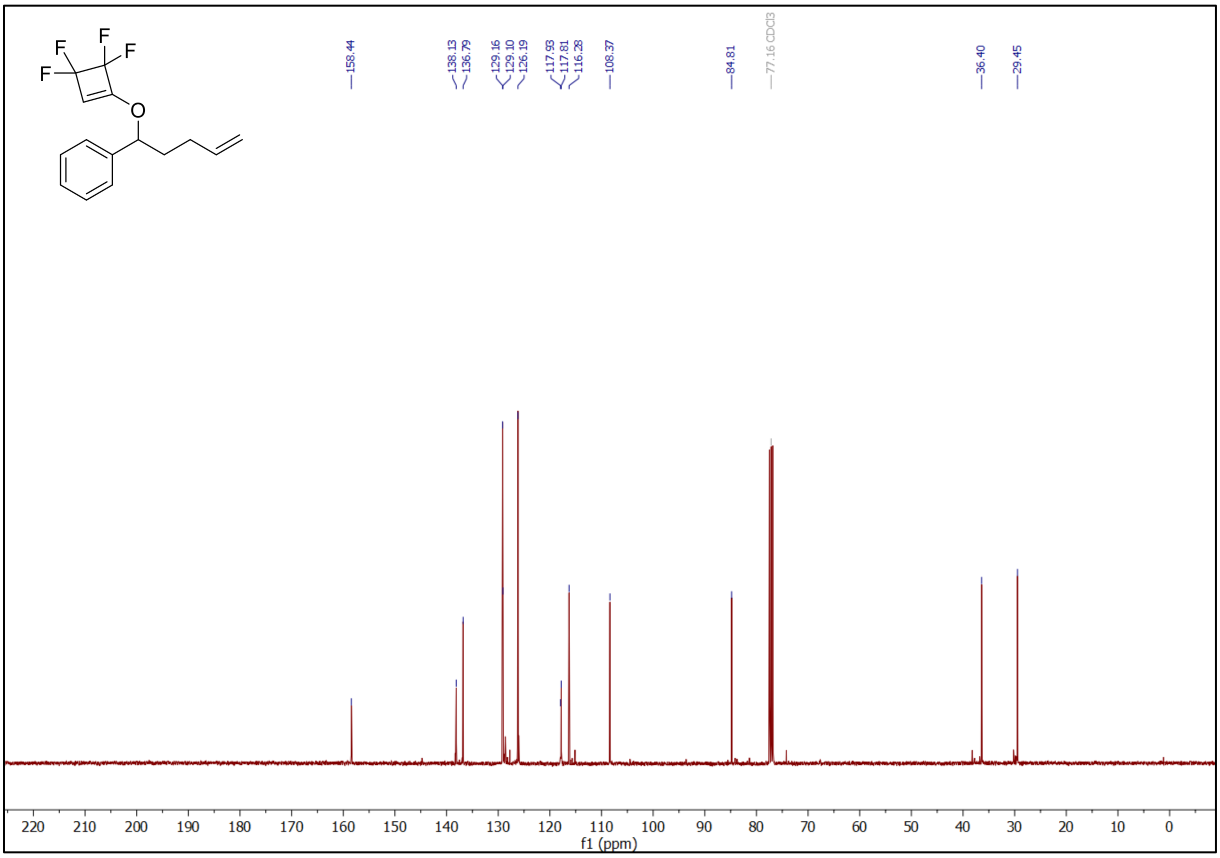

Figure S79: ^{13}C NMR spectrum of tetrafluorocyclobutene **12b** ($CDCl_3$, 101 MHz)

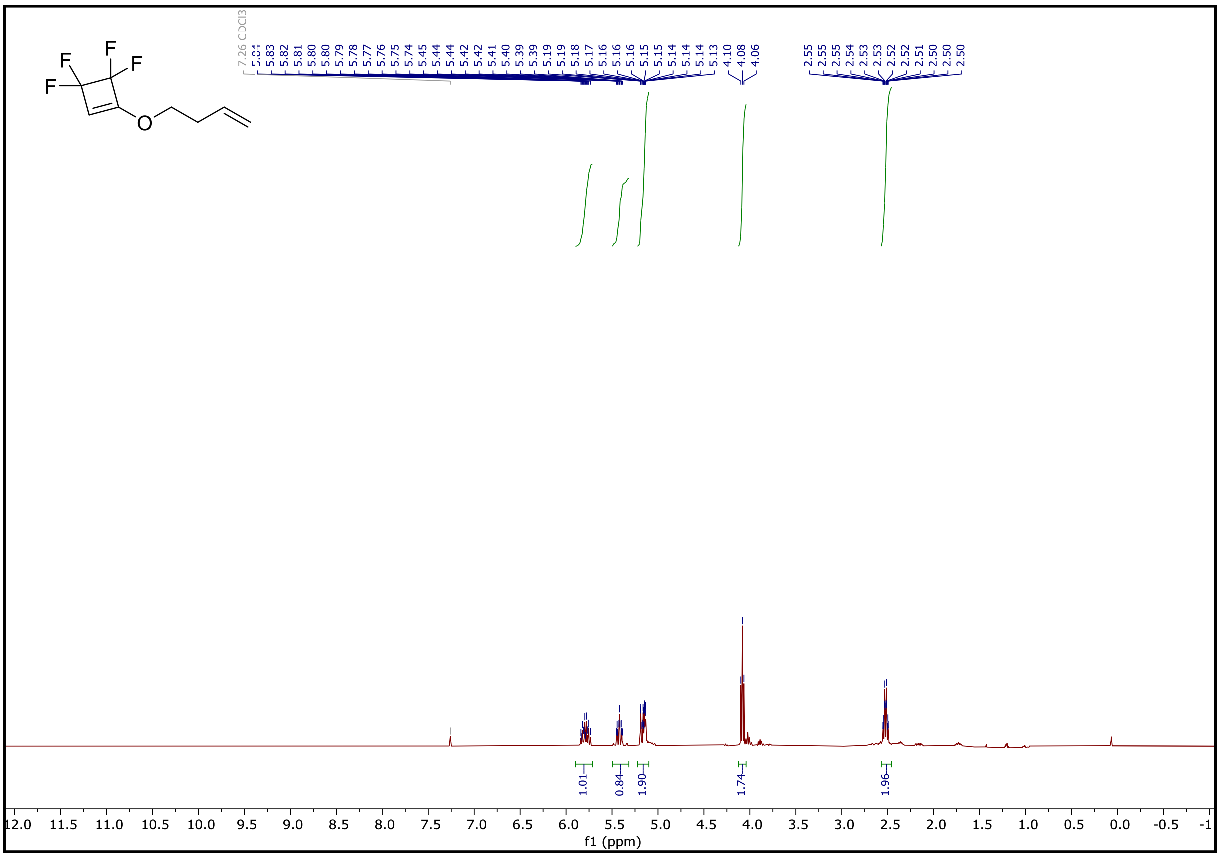

Figure S80: ^{1}H NMR spectrum of tetrafluorocyclobutene **3a** ($CDCl_3$, 400 MHz)

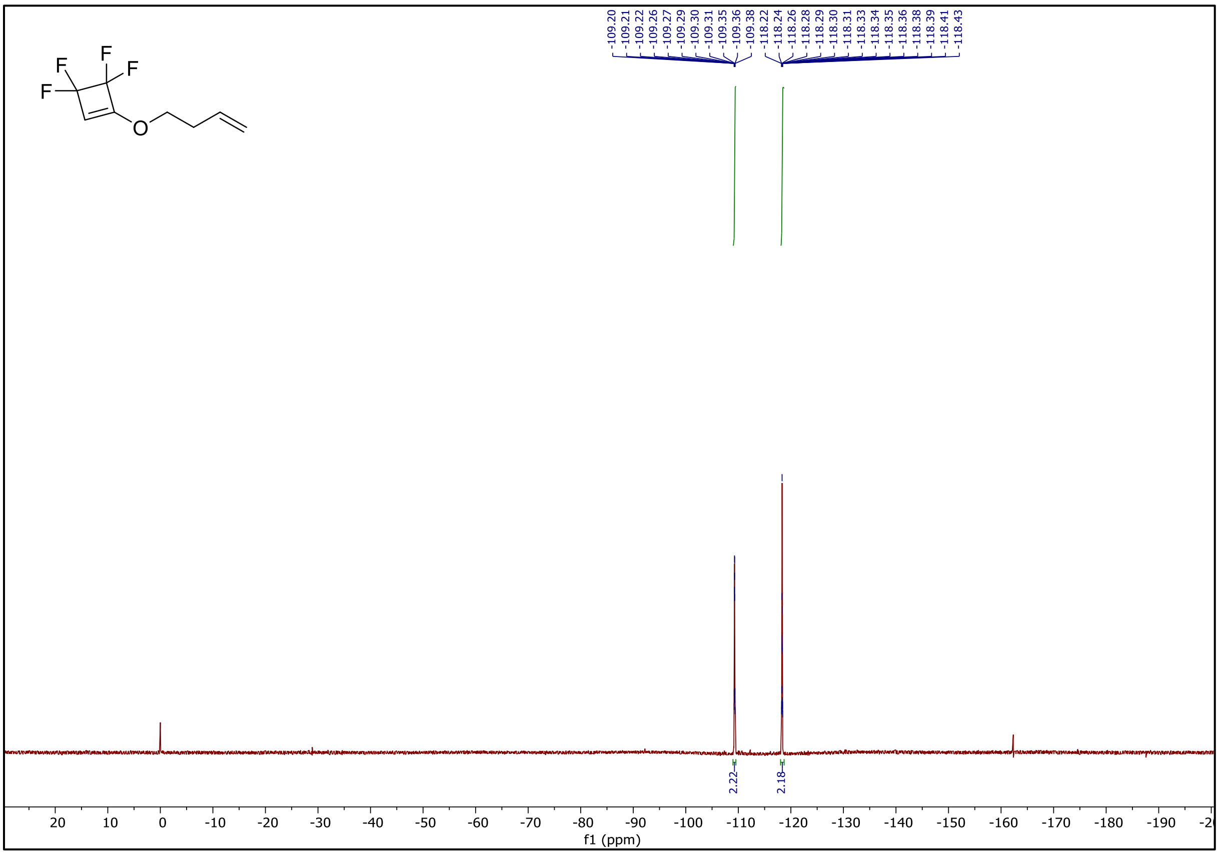

Figure S81: ^{19}F NMR spectrum of tetrafluorocyclobutene **3a** ($CDCl_3$, 282 MHz)

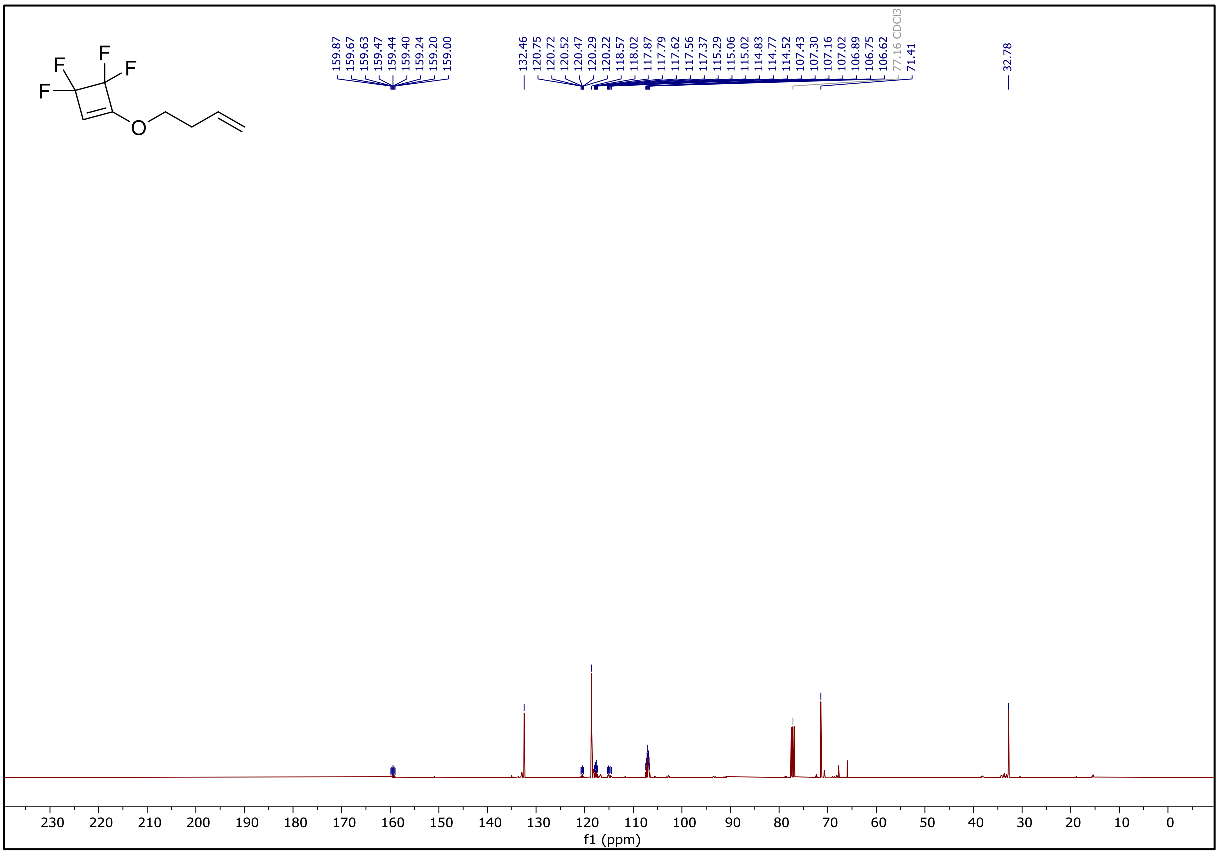

Figure S82: ^{13}C NMR spectrum of tetrafluorocyclobutene **3a** ($CDCl_3$, 101 MHz)

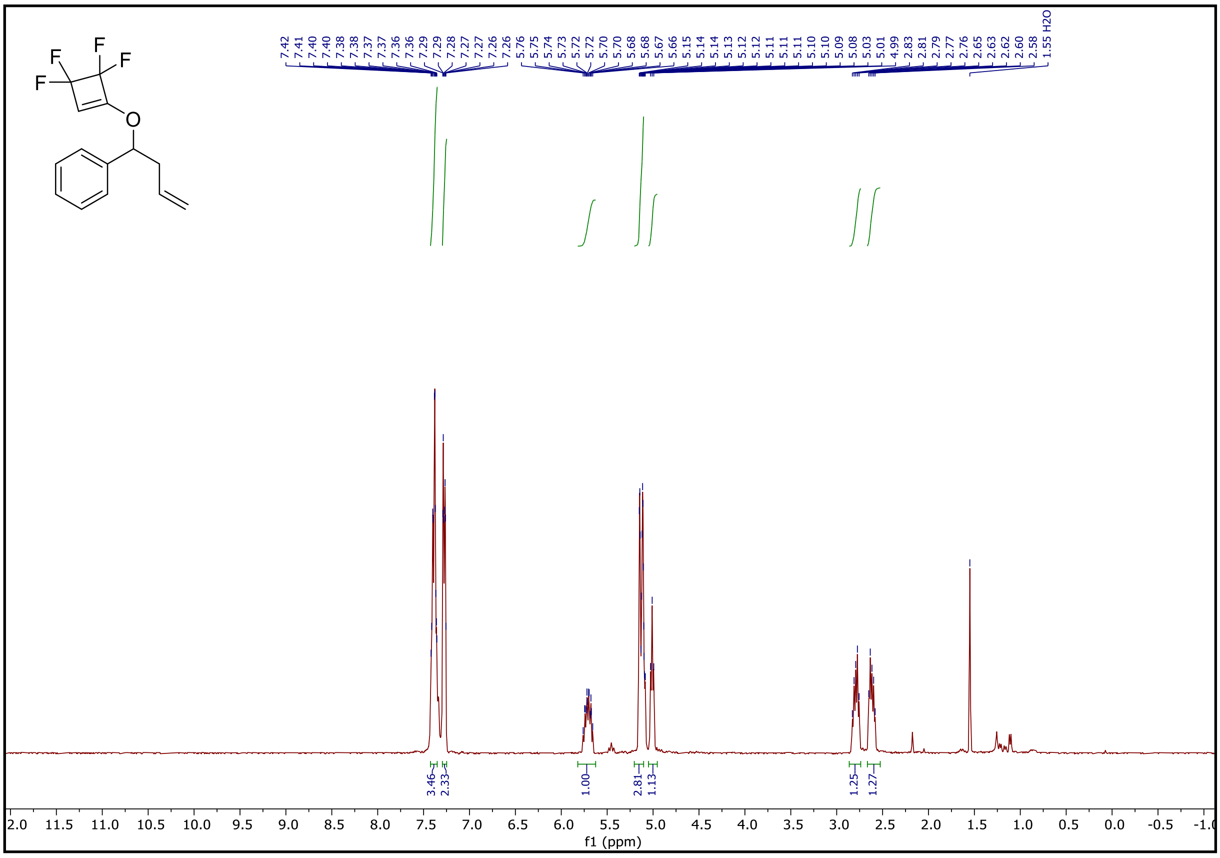

Figure S83: ^{1}H NMR spectrum of tetrafluorocyclobutene **3b** ($CDCl_3$, 400 MHz)

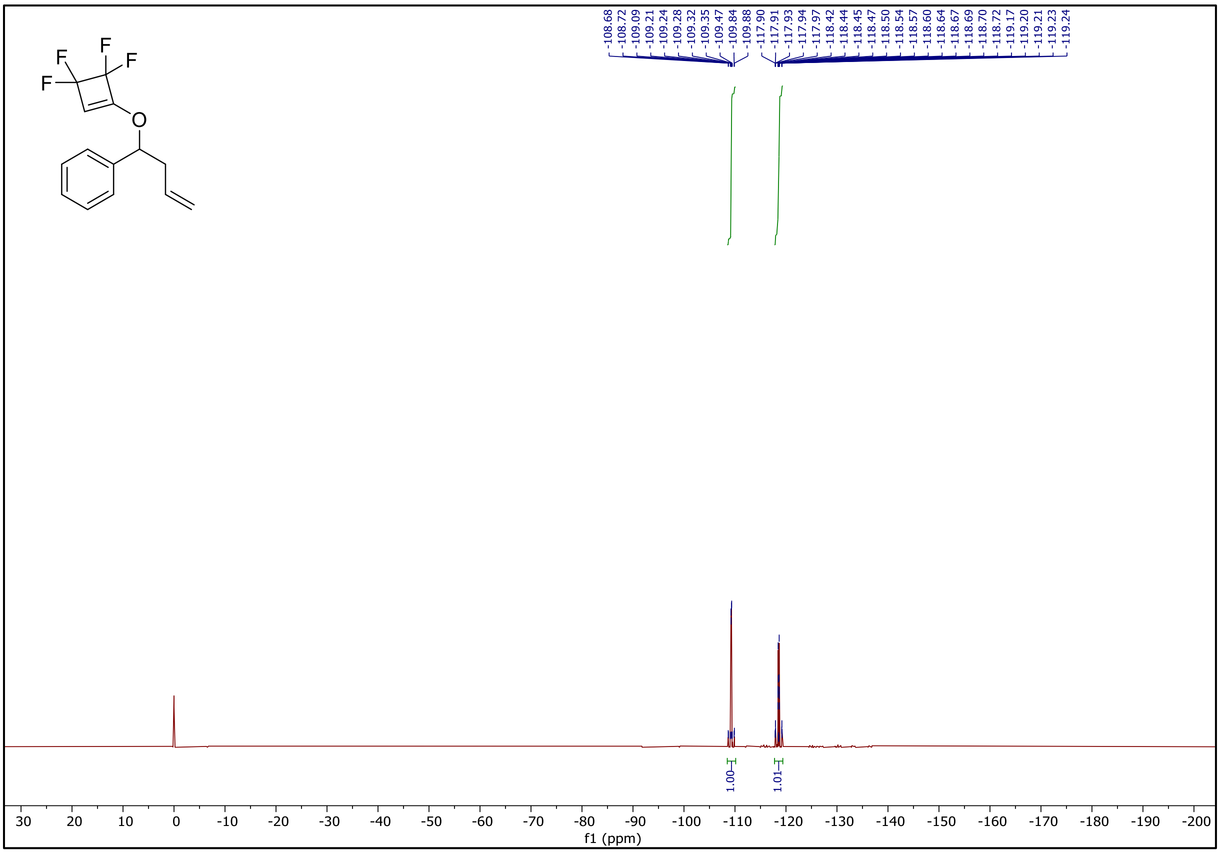

Figure S84: ^{19}F NMR spectrum of tetrafluorocyclobutene **3b** ($CDCl_3$, 282 MHz)

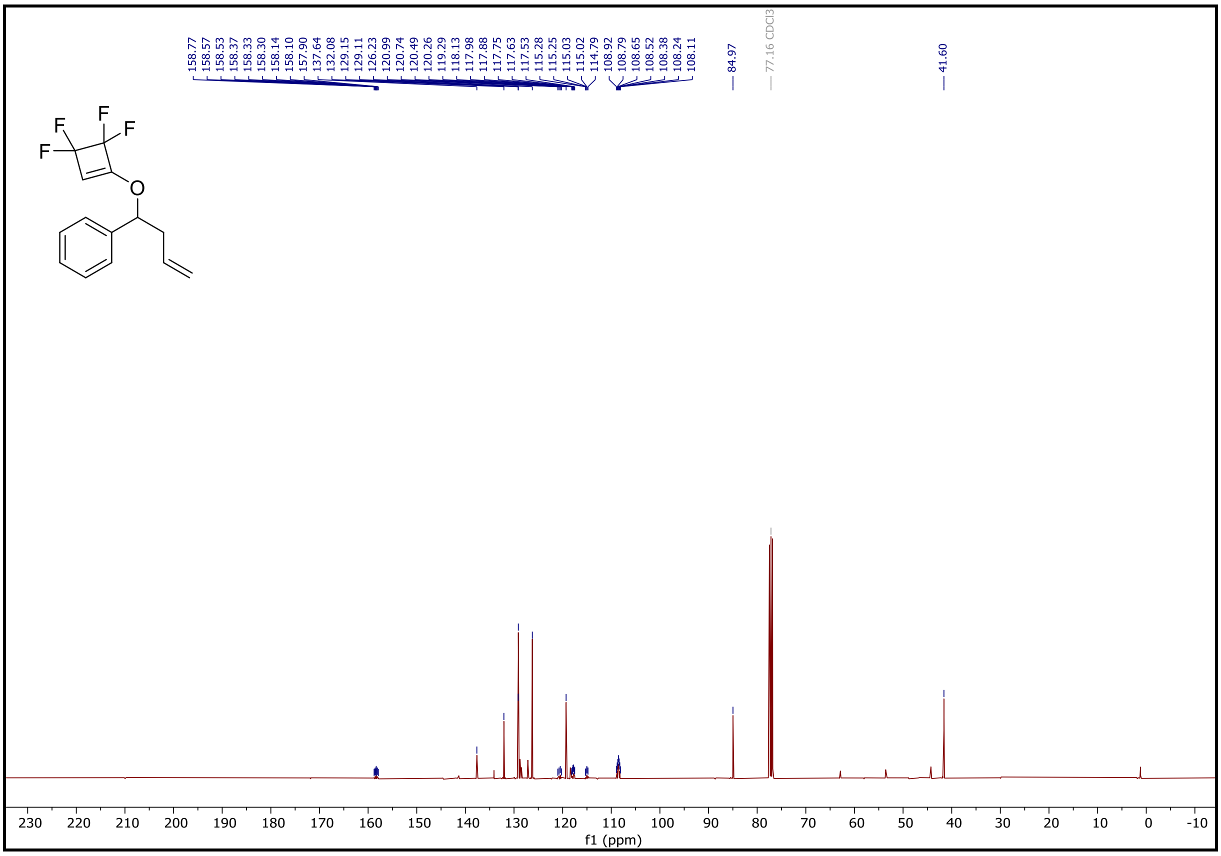

Figure S85: ^{13}C NMR spectrum of tetrafluorocyclobutene **3b** ($CDCl_3$, 101 MHz)

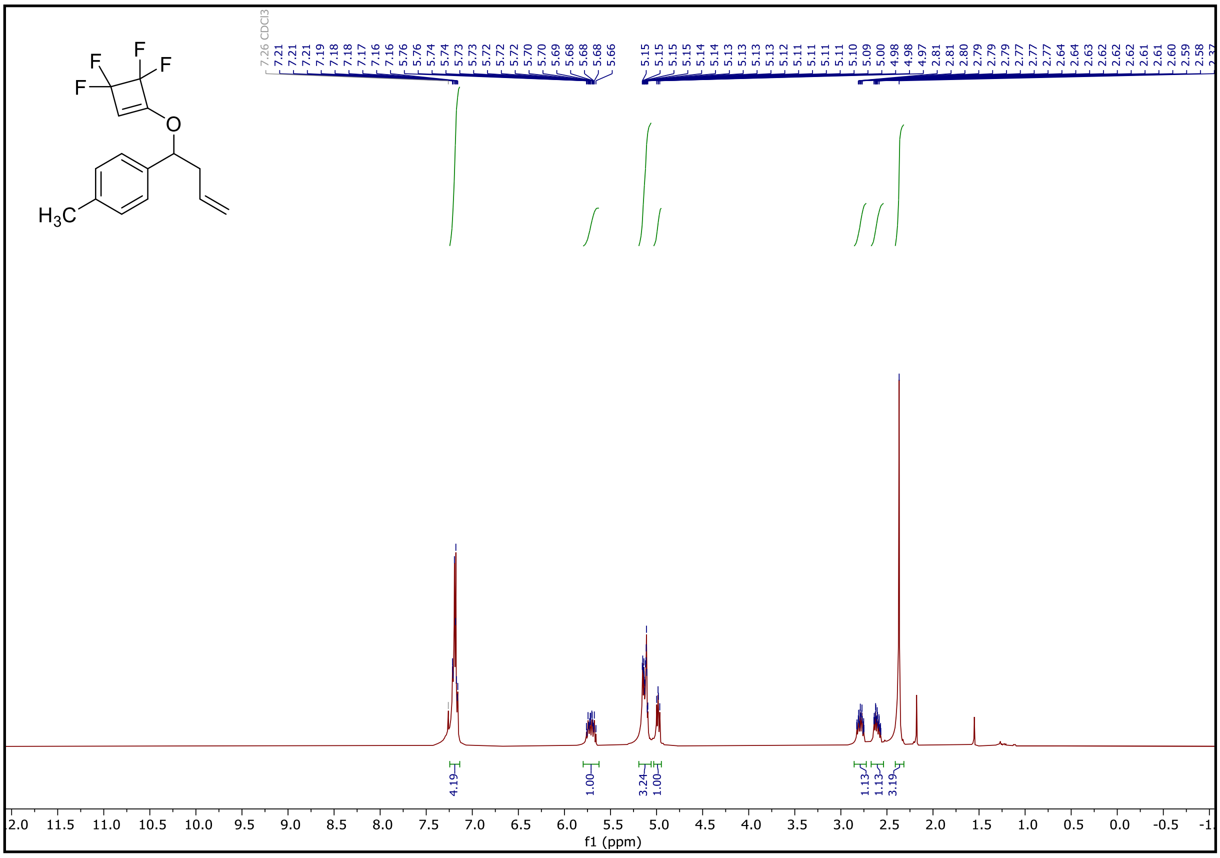

Figure S86: ^{1}H NMR spectrum of tetrafluorocyclobutene **3c** ($CDCl_3$, 400 MHz)

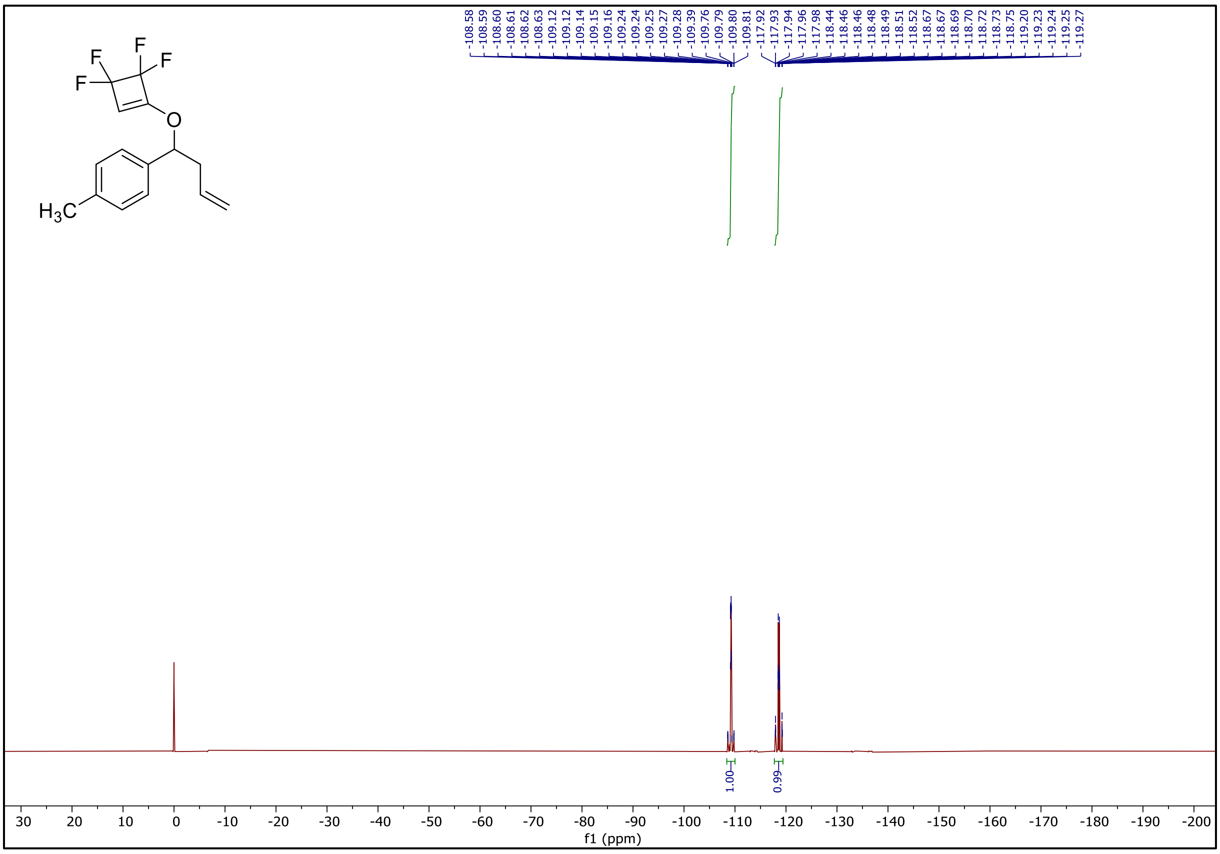

Figure S87: ^{19}F NMR spectrum of tetrafluorocyclobutene **3c** ($CDCl_3$, 376 MHz)

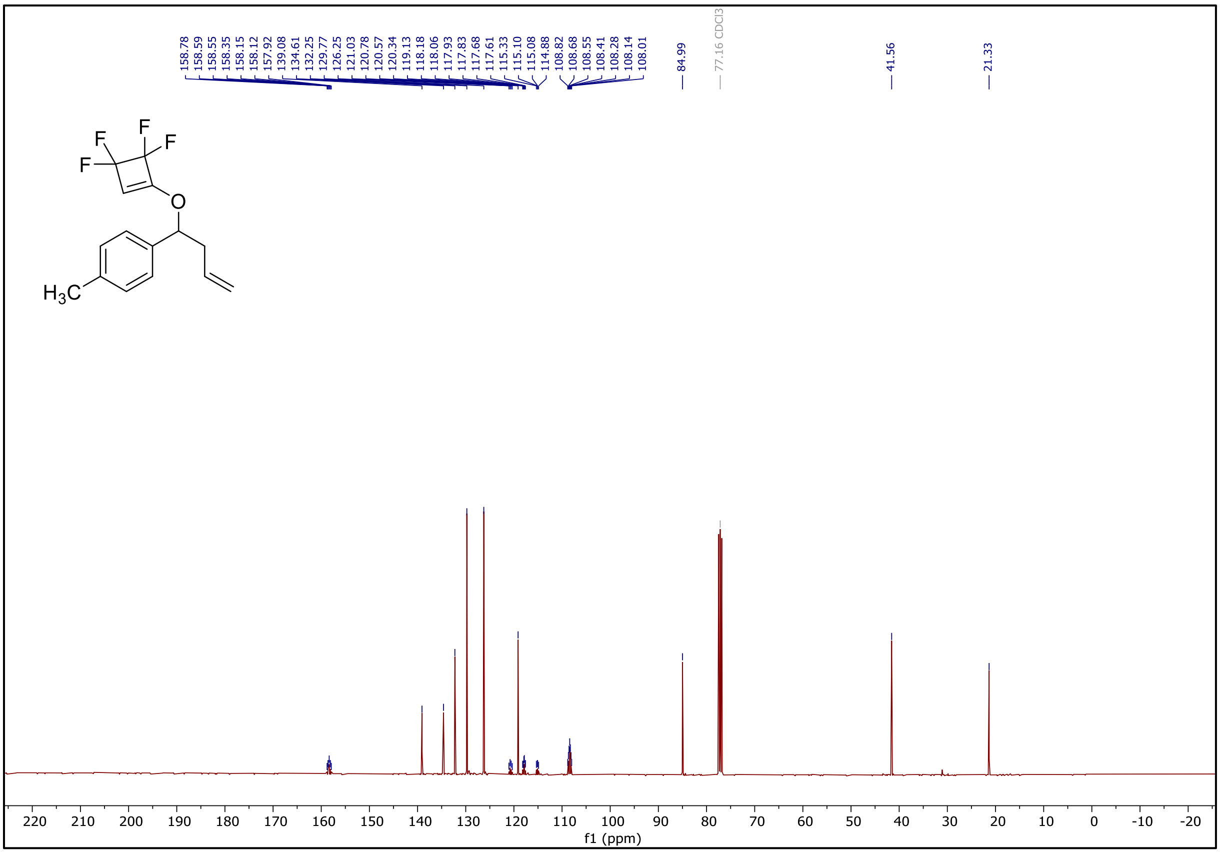

Figure S88: ^{13}C NMR spectrum of tetrafluorocyclobutene **3c** ($CDCl_3$, 101 MHz)

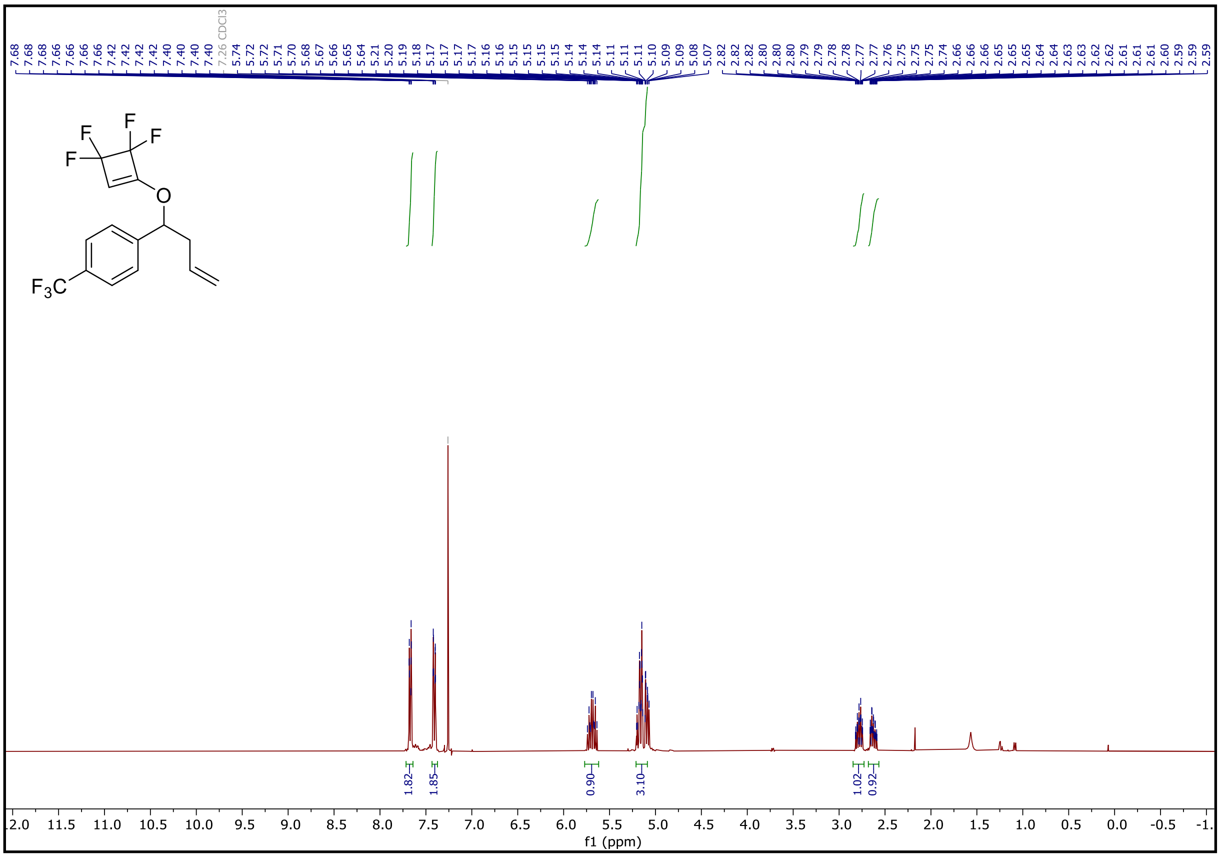

Figure S89: ^{1}H NMR spectrum of tetrafluorocyclobutene **3d** ($CDCl_3$, 400 MHz)

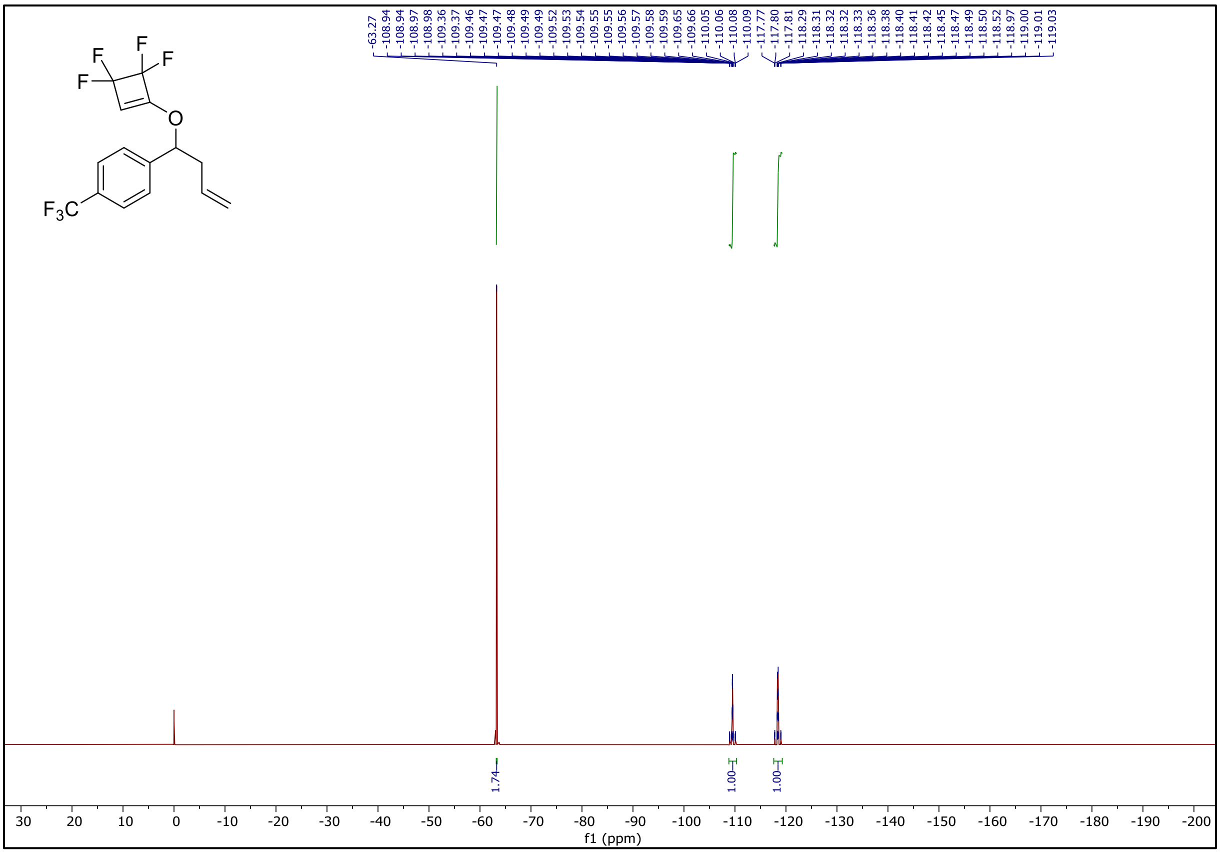

Figure S90: ^{19}F NMR spectrum of tetrafluorocyclobutene **3d** ($CDCl_3$, 376 MHz)

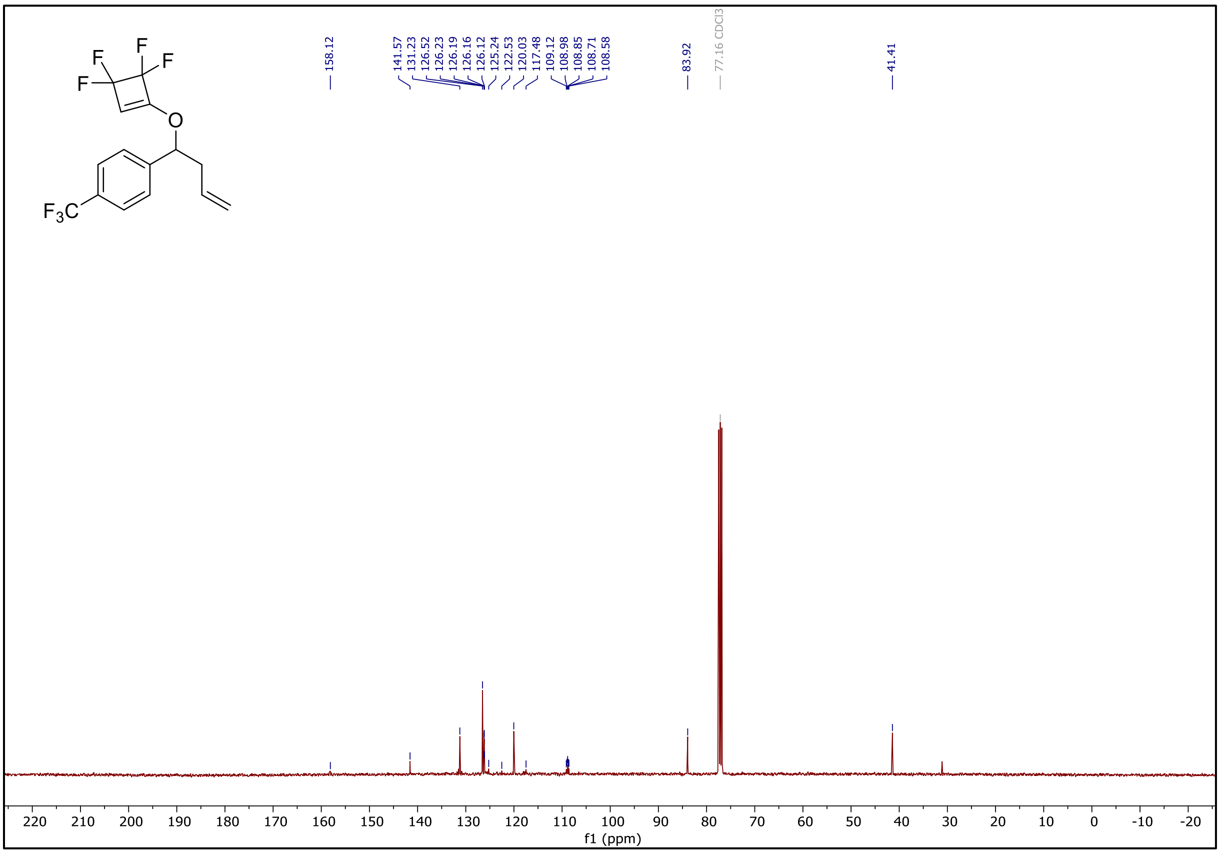

Figure S91: ^{13}C NMR spectrum of tetrafluorocyclobutene **3d** ($CDCl_3$, 101 MHz)

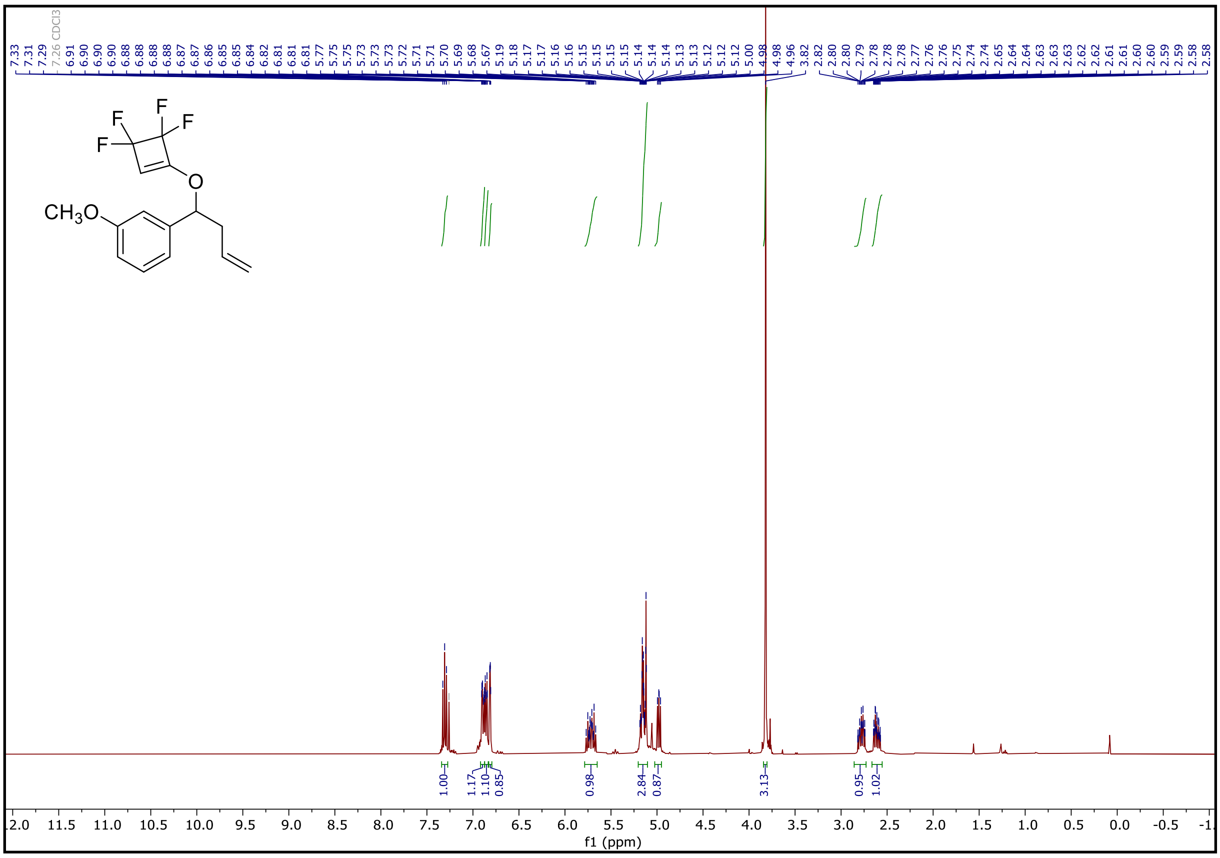

Figure S92: ^{1}H NMR spectrum of tetrafluorocyclobutene **3e** ($CDCl_3$, 400 MHz)

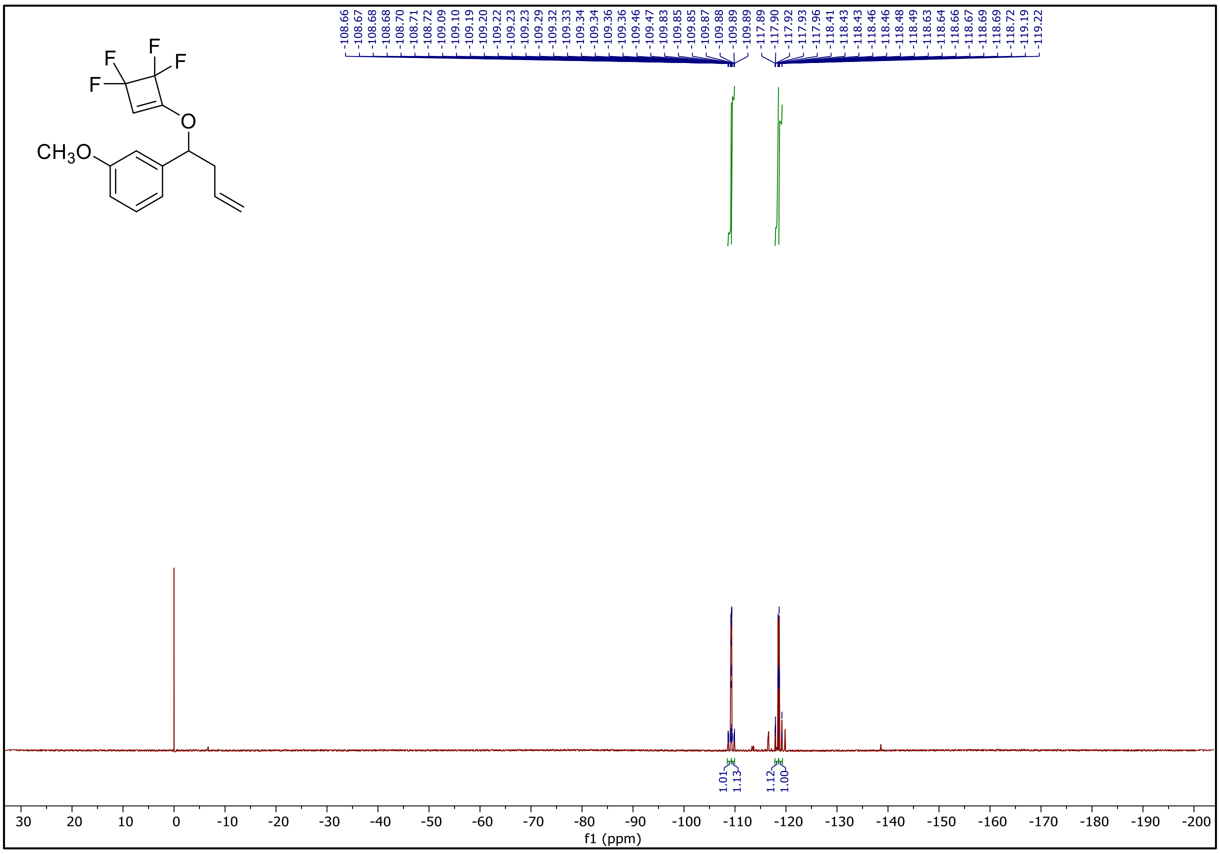

Figure S93: ^{19}F NMR spectrum of tetrafluorocyclobutene **3e** ($CDCl_3$, 376 MHz)

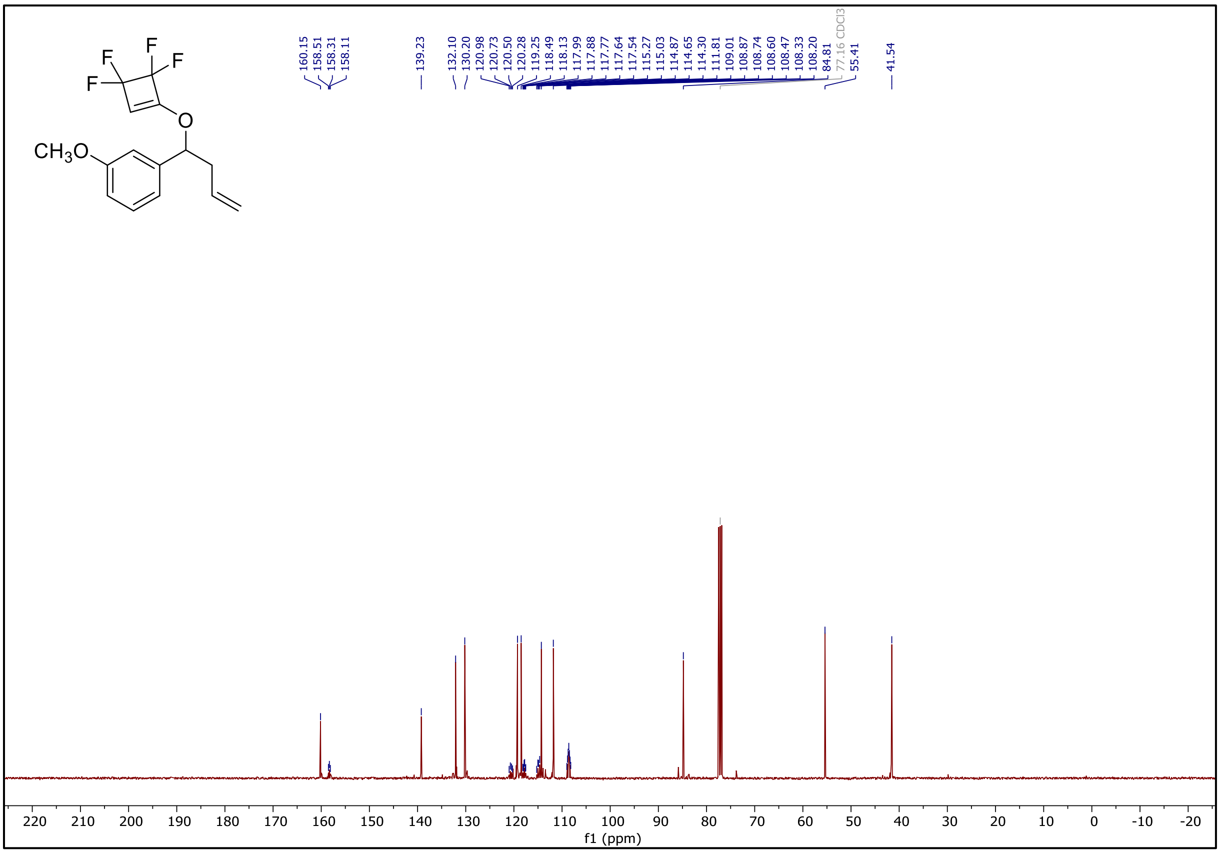

Figure S94: ^{13}C NMR spectrum of tetrafluorocyclobutene **3e** ($CDCl_3$, 101 MHz)

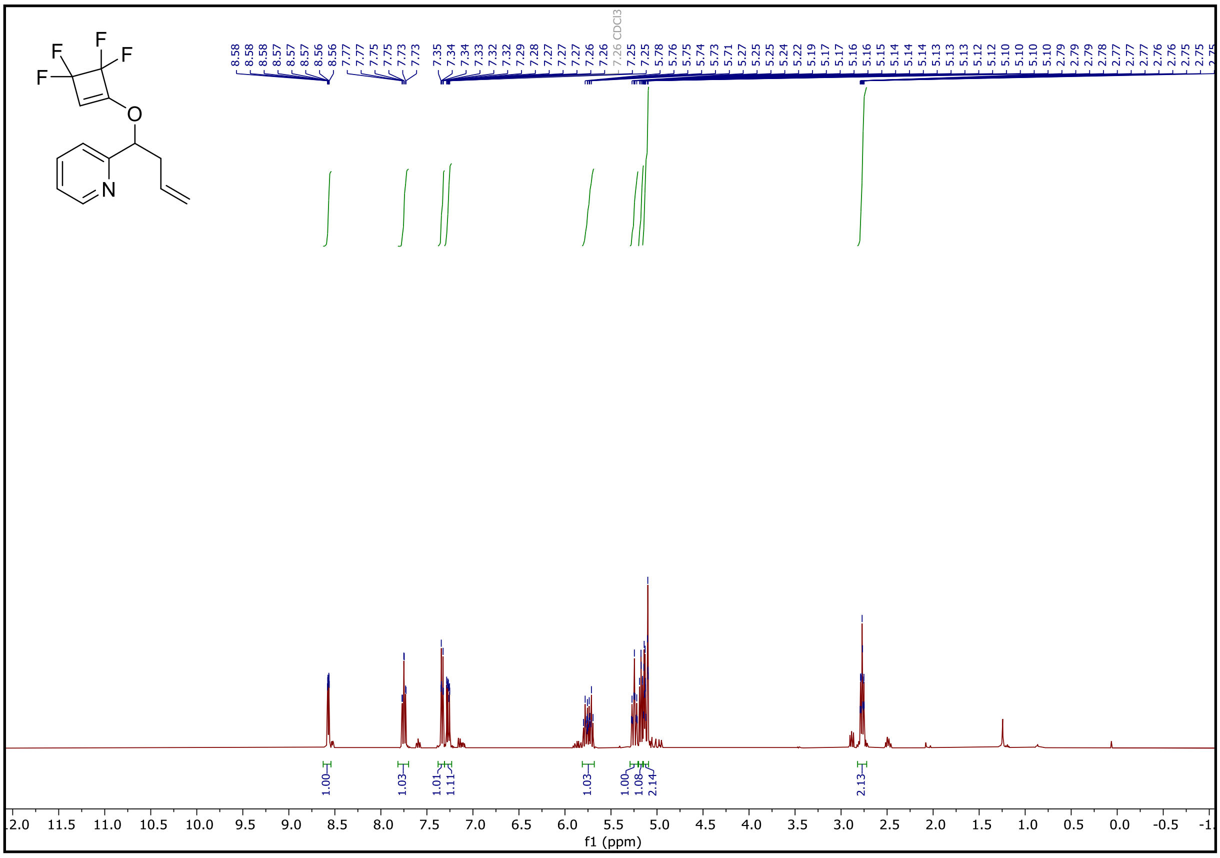

Figure S95: ^{1}H NMR spectrum of tetrafluorocyclobutene **3f** ($CDCl_3$, 400 MHz)

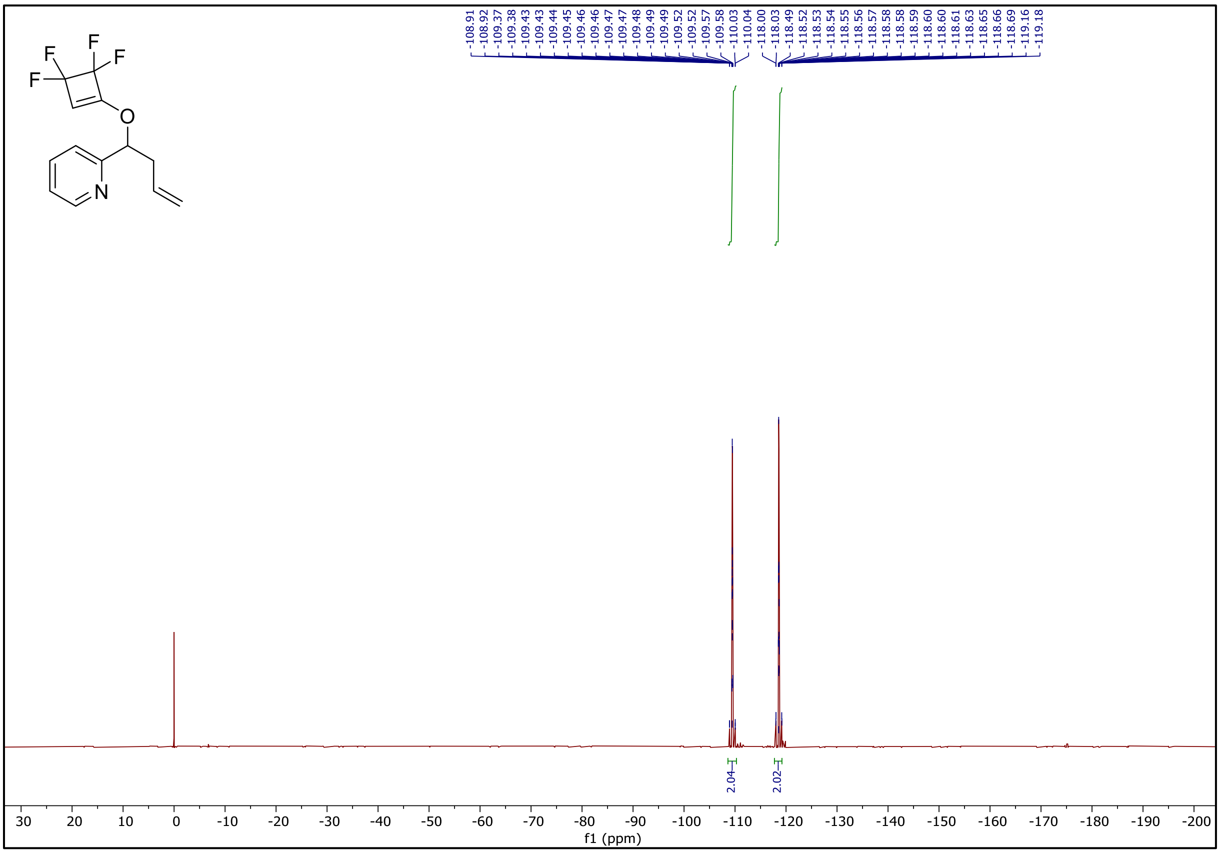

Figure S96: ^{19}F NMR spectrum of tetrafluorocyclobutene **3f** ($CDCl_3$, 376 MHz)

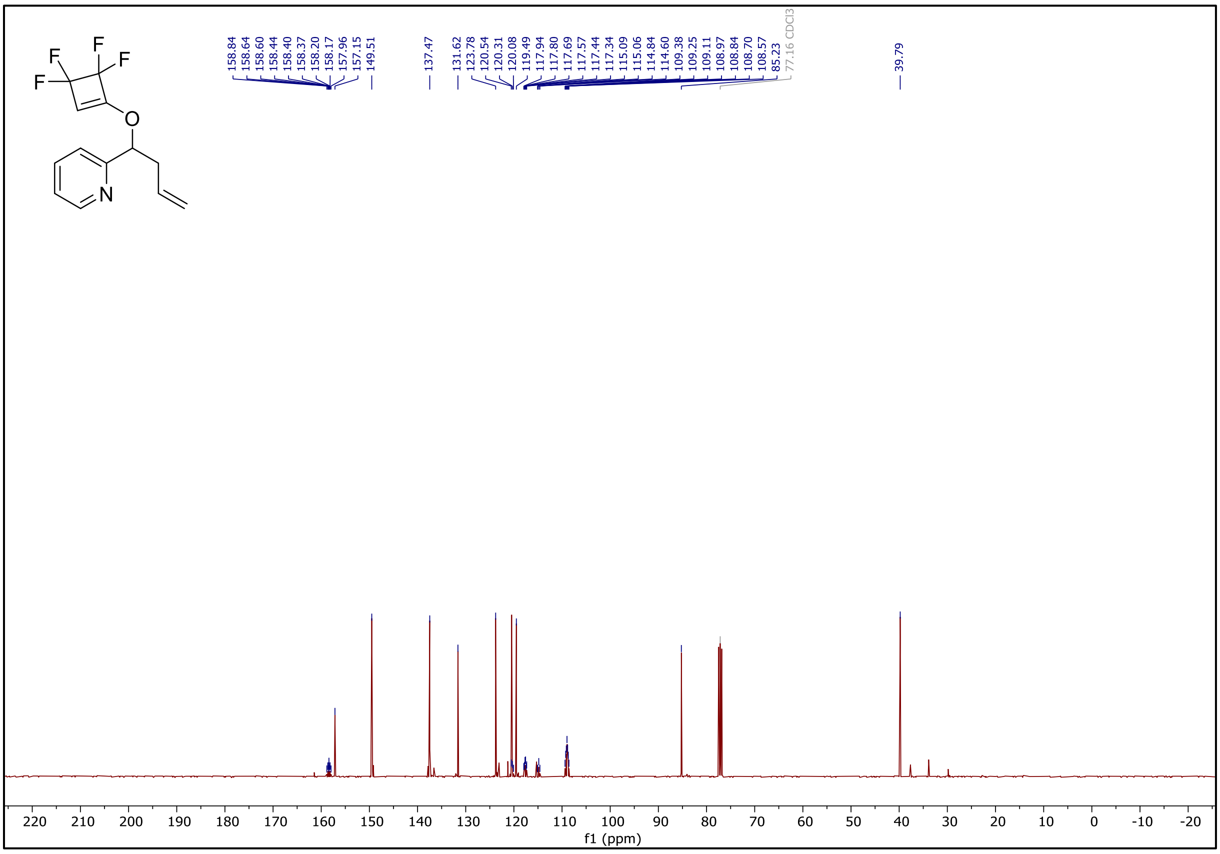

Figure S97: ^{13}C NMR spectrum of tetrafluorocyclobutene **3f** ($CDCl_3$, 101 MHz)

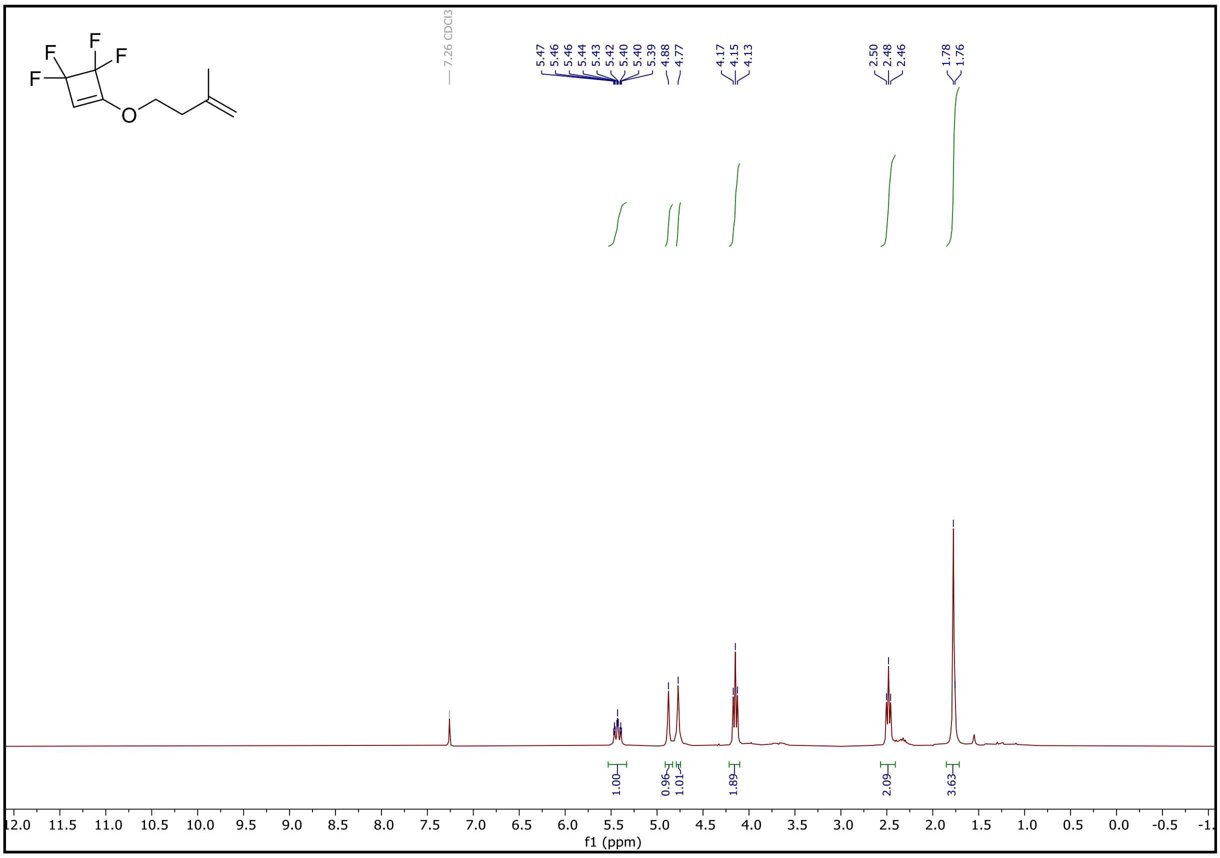

Figure S98: ^{1}H NMR spectrum of tetrafluorocyclobutene **3g** ($CDCl_3$, 400 MHz)

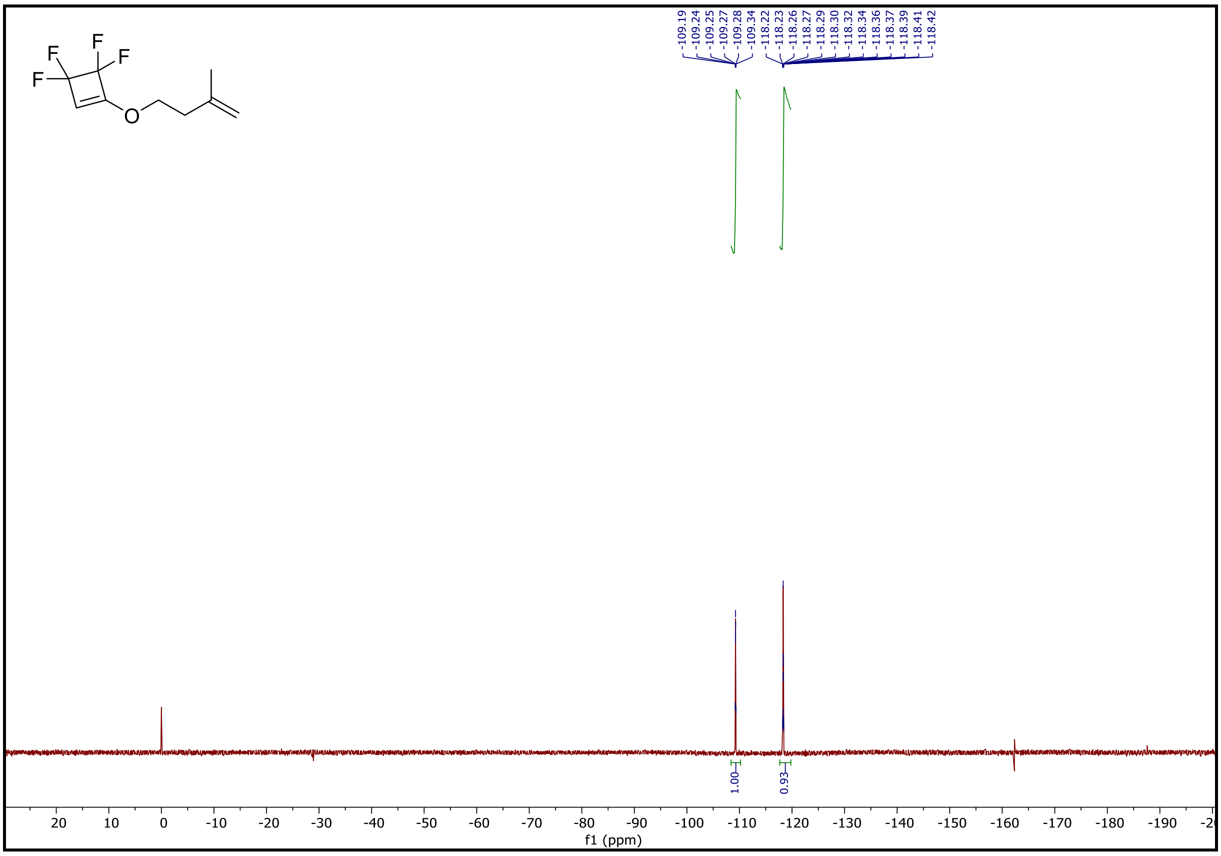

Figure S99: ^{19}F NMR spectrum of tetrafluorocyclobutene **3g** ($CDCl_3$, 282 MHz)

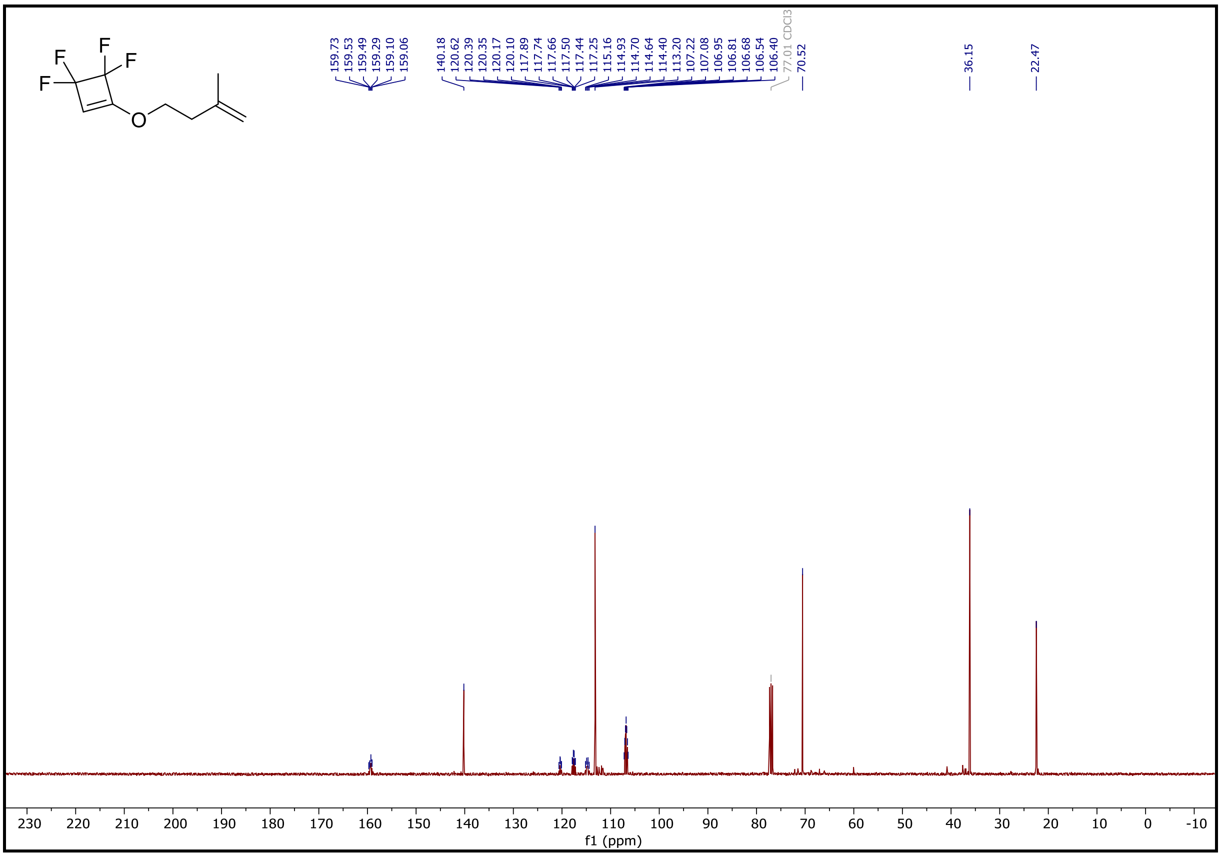

Figure S100: ^{13}C NMR spectrum of tetrafluorocyclobutene **3g** ($CDCl_3$, 101 MHz)

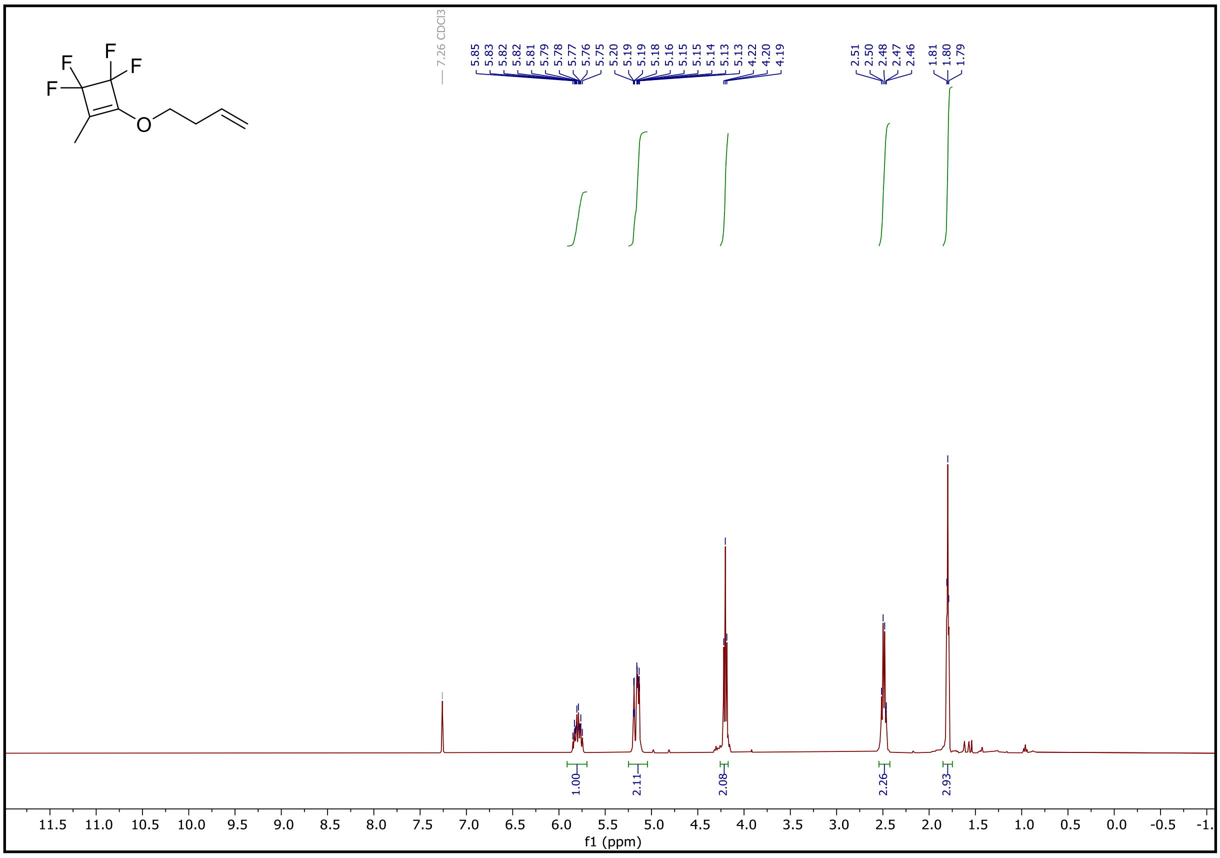

Figure S101: ^{1}H NMR spectrum of tetrafluorocyclobutene **15a** ($CDCl_3$, 400 MHz)

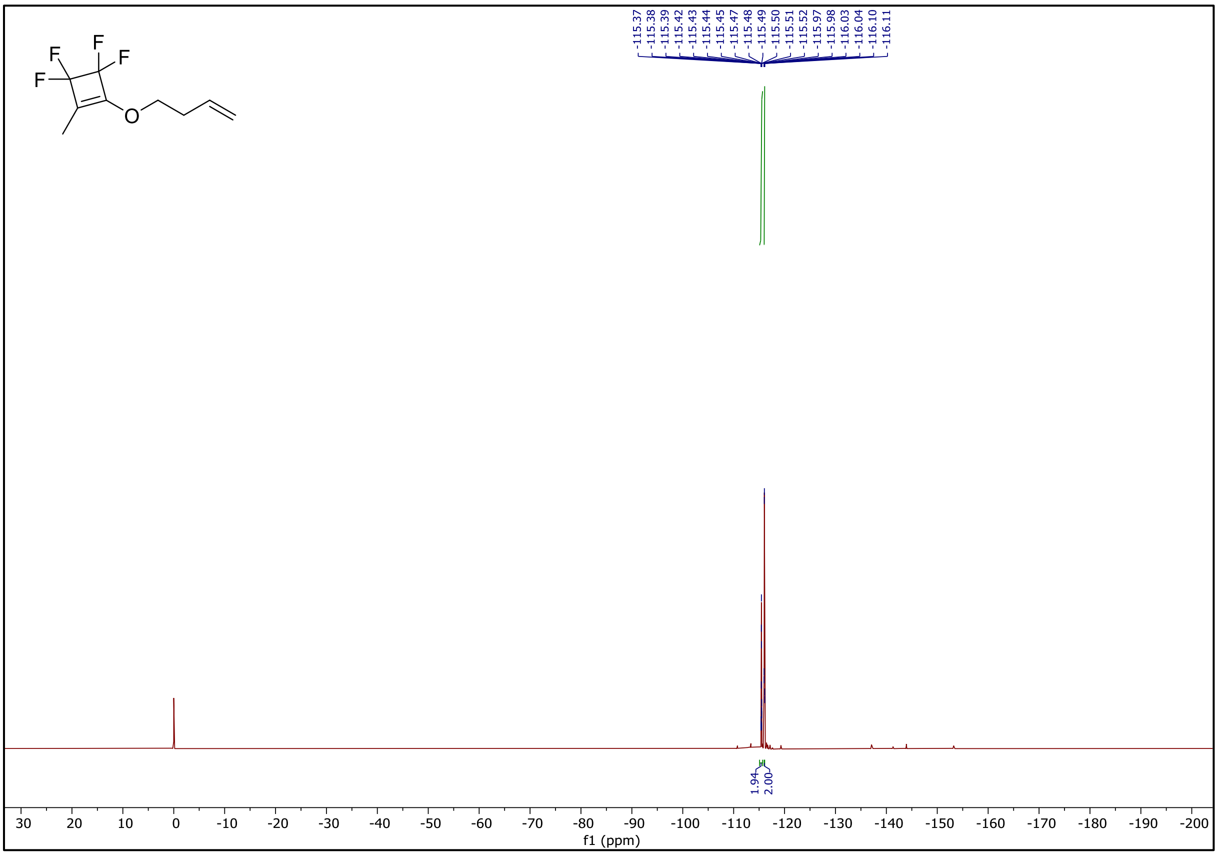

Figure S102: ^{19}F NMR spectrum of tetrafluorocyclobutene **15a** ($CDCl_3$, 376 MHz)

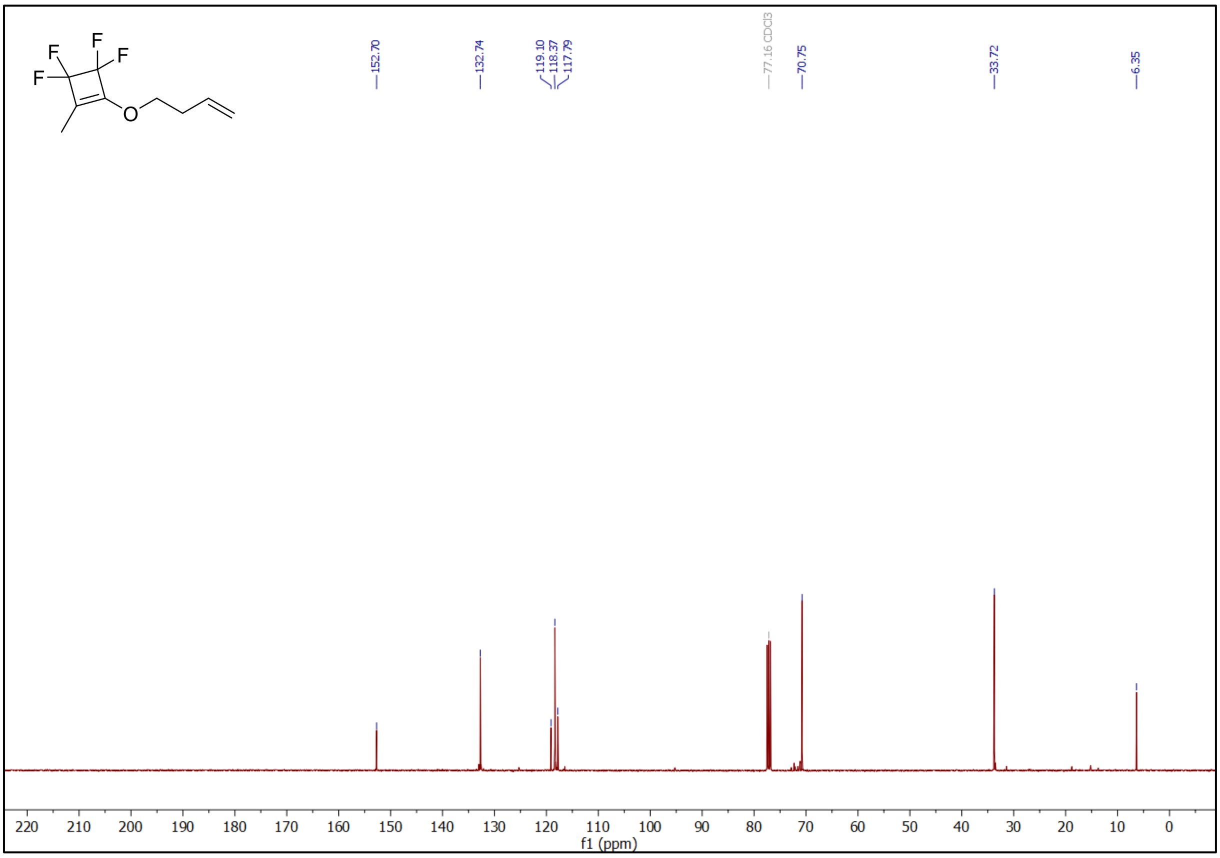

Figure S103: ^{13}C NMR spectrum of tetrafluorocyclobutene **15a** ($CDCl_3$,101 MHz,^{1}H and ^{19}F decoupling)

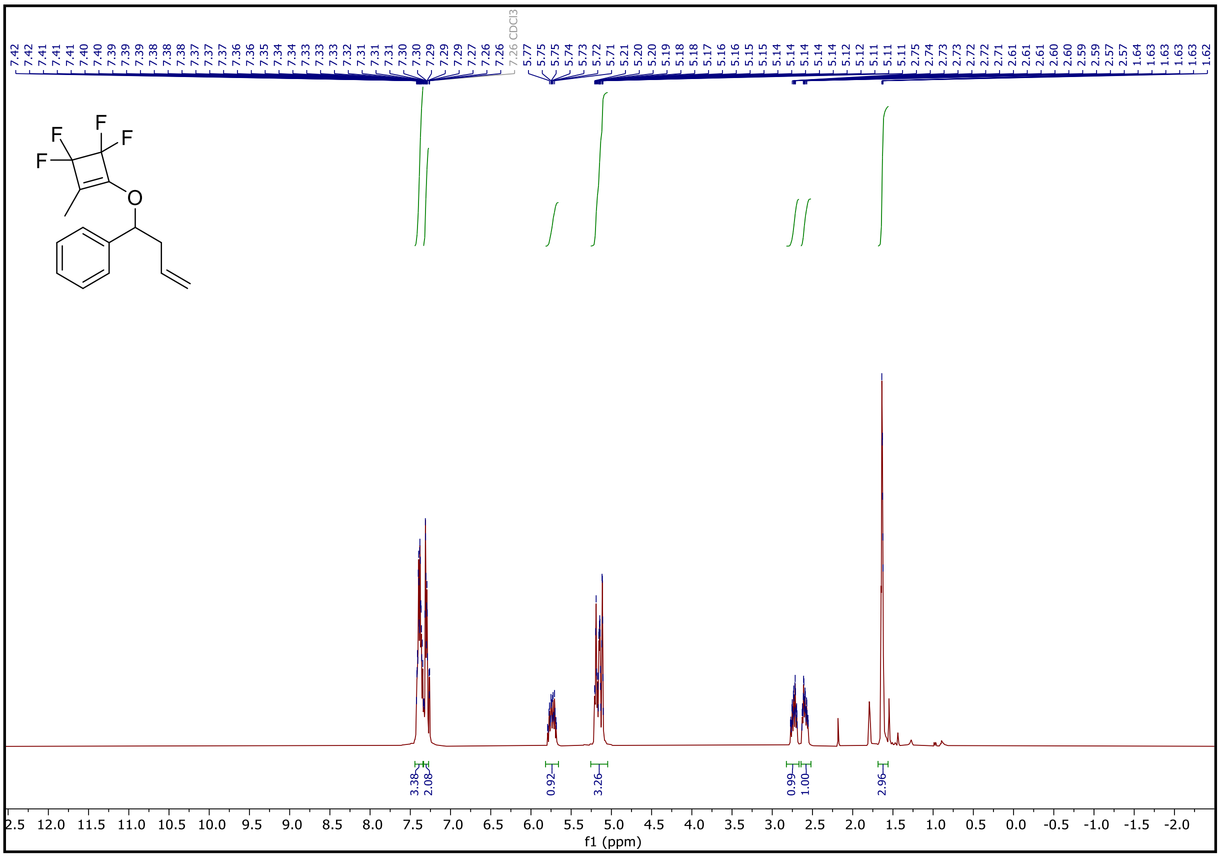

Figure S104: ^{1}H NMR spectrum of tetrafluorocyclobutene **15b** ($CDCl_3$, 400 MHz)

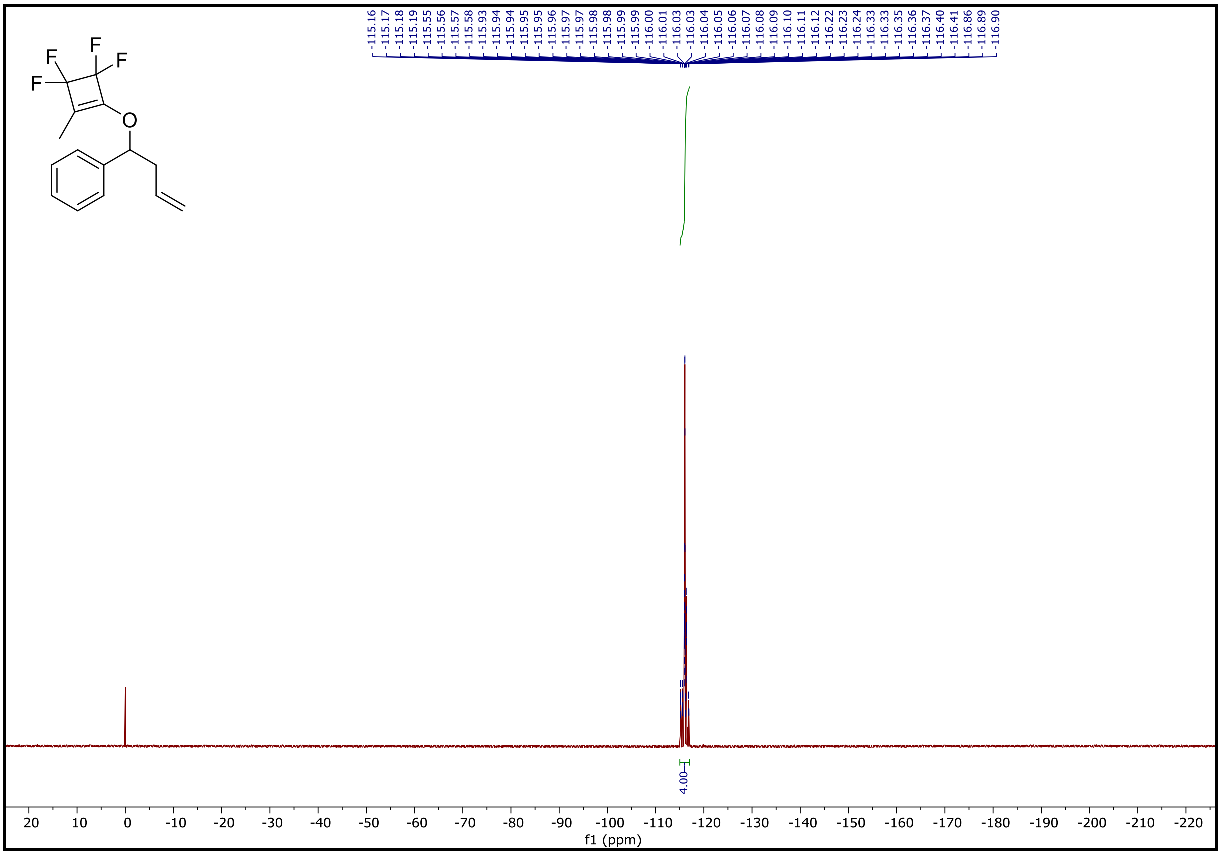

Figure S105: ^{19}F NMR spectrum of tetrafluorocyclobutene **15b** ($CDCl_3$, 376 MHz)

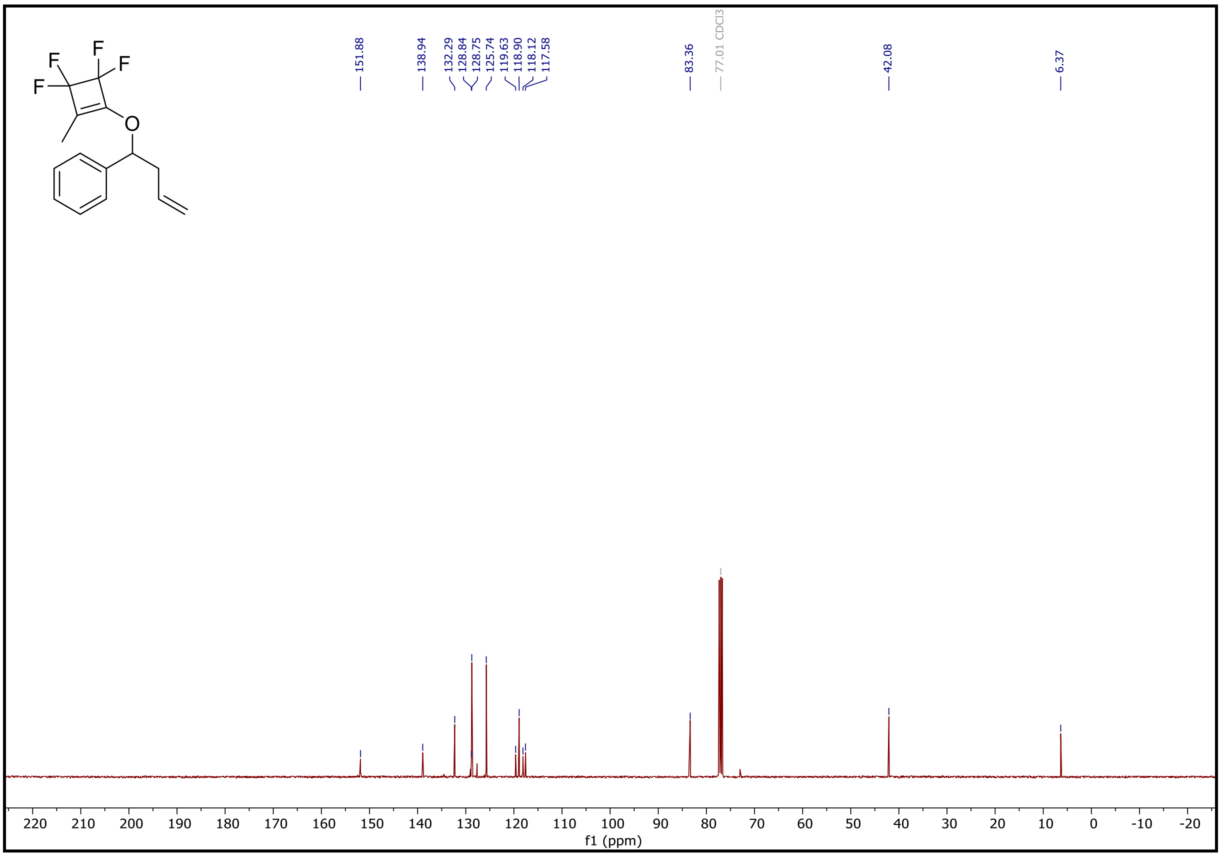

Figure S106: ^{13}C NMR spectrum of tetrafluorocyclobutene **15b** ($CDCl_3$,101 MHz,^{1}H and ^{19}F decoupling)

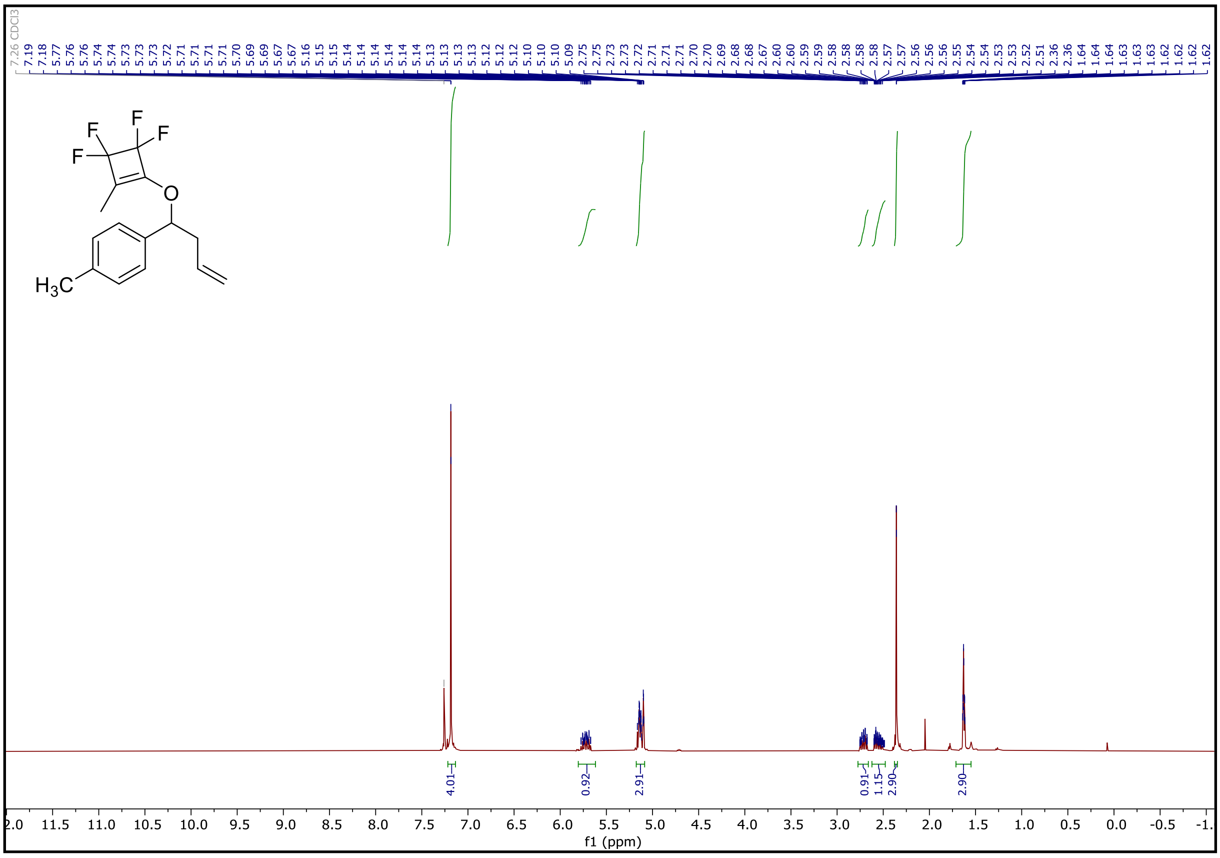

Figure S107: ^{1}H NMR spectrum of tetrafluorocyclobutene **15c** ($CDCl_3$, 400 MHz)

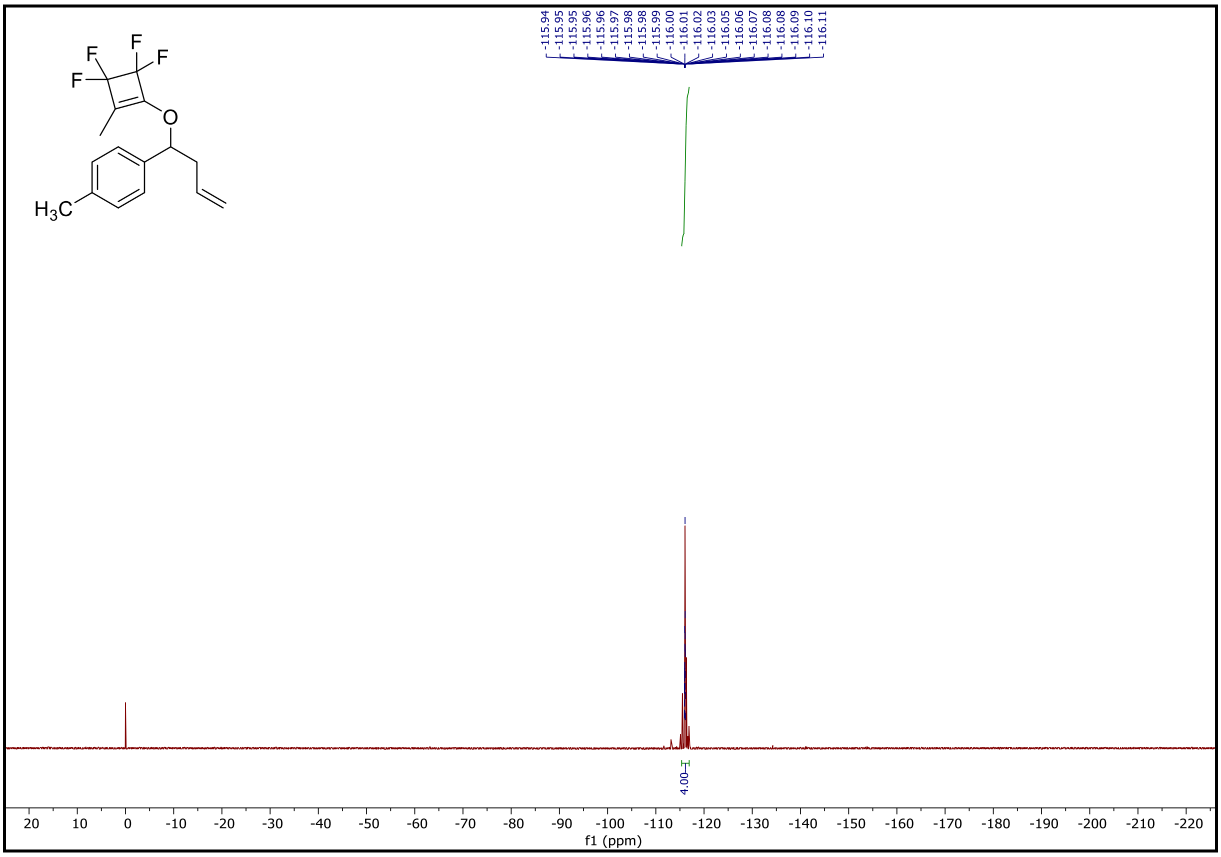

Figure S108: ^{19}F NMR spectrum of tetrafluorocyclobutene **15c** ($CDCl_3$, 376 MHz)

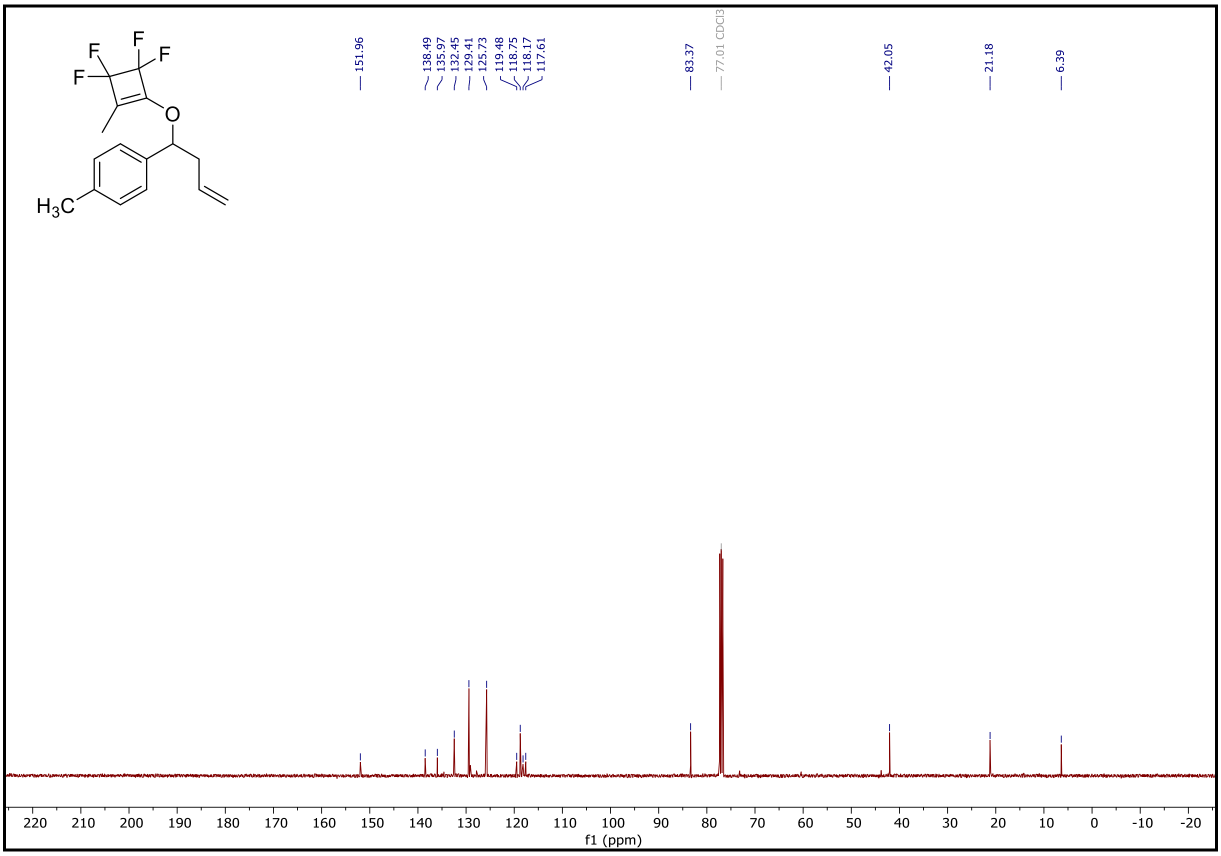

Figure S109: ^{13}C NMR spectrum of tetrafluorocyclobutene **15c** ($CDCl_3$,101 MHz,^{1}H and ^{19}F decoupling)

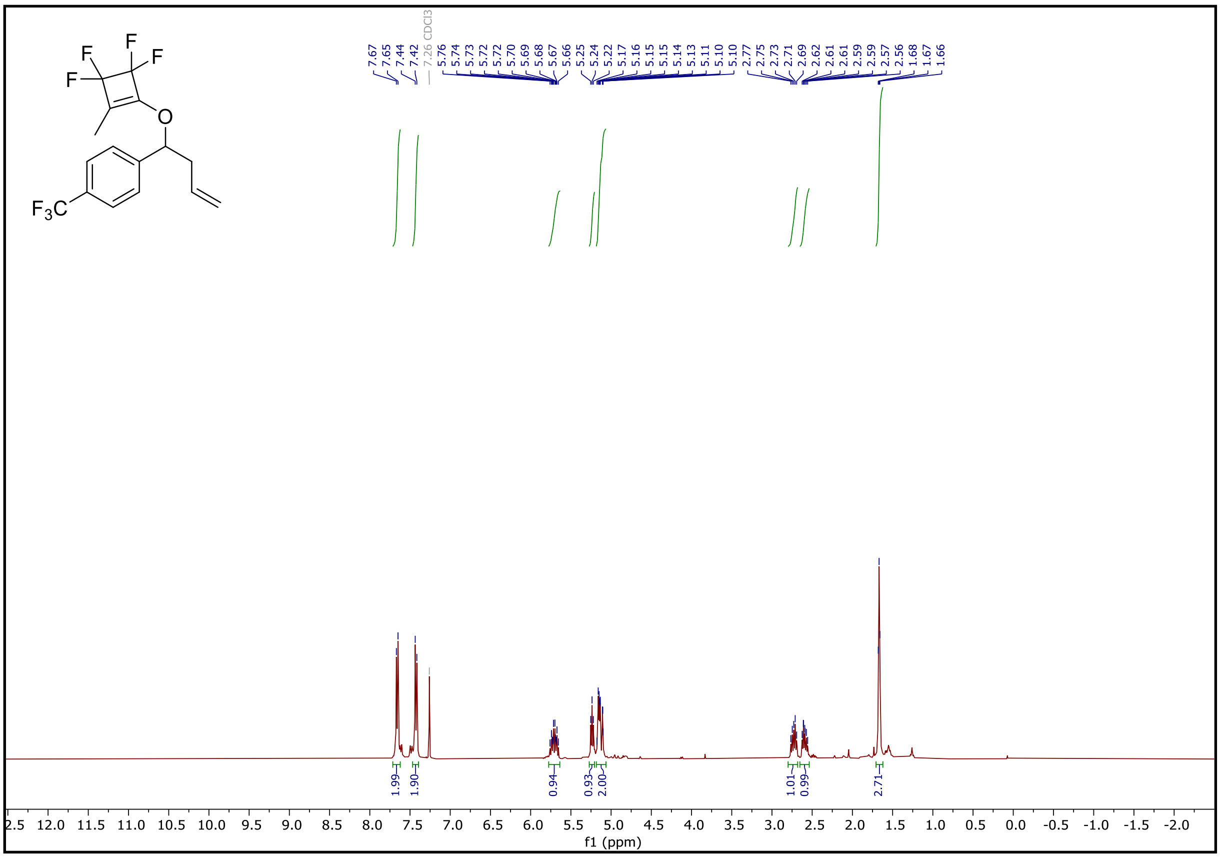

Figure S110: ^{1}H NMR spectrum of tetrafluorocyclobutene **15d** ($CDCl_3$, 400 MHz)

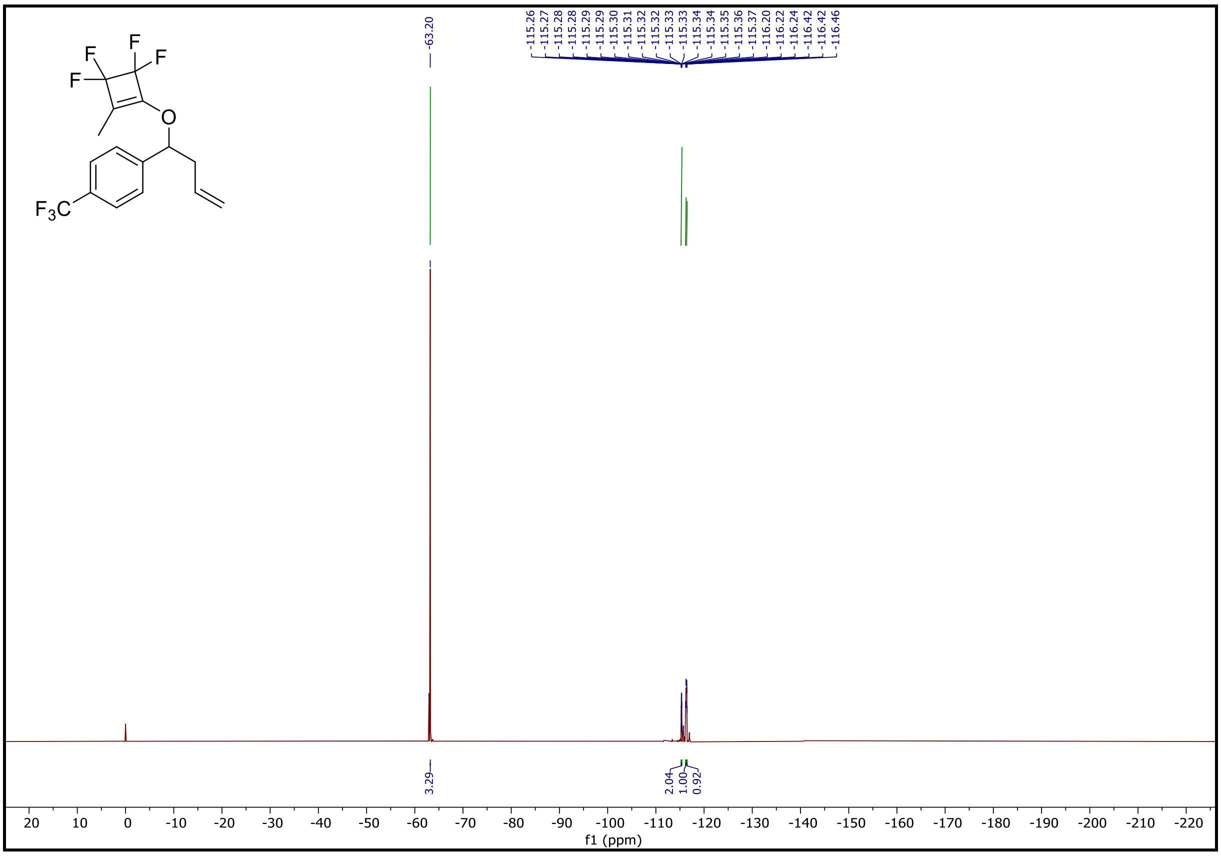

Figure S111: ^{19}F NMR spectrum of tetrafluorocyclobutene **15d** ($CDCl_3$, 376 MHz)

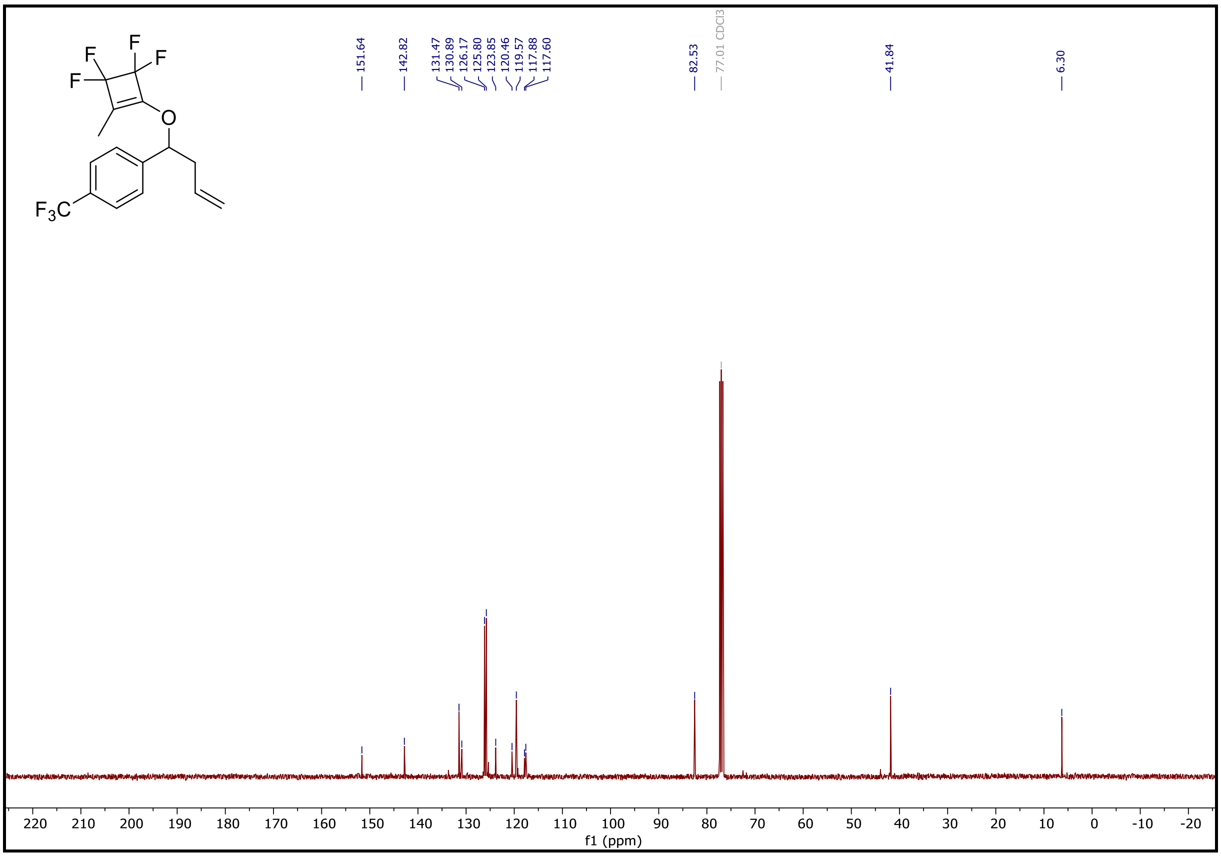

Figure S112: ^{13}C NMR spectrum of tetrafluorocyclobutene **15d** ($CDCl_3$,101 MHz,^1H and ^{19}F decoupling)

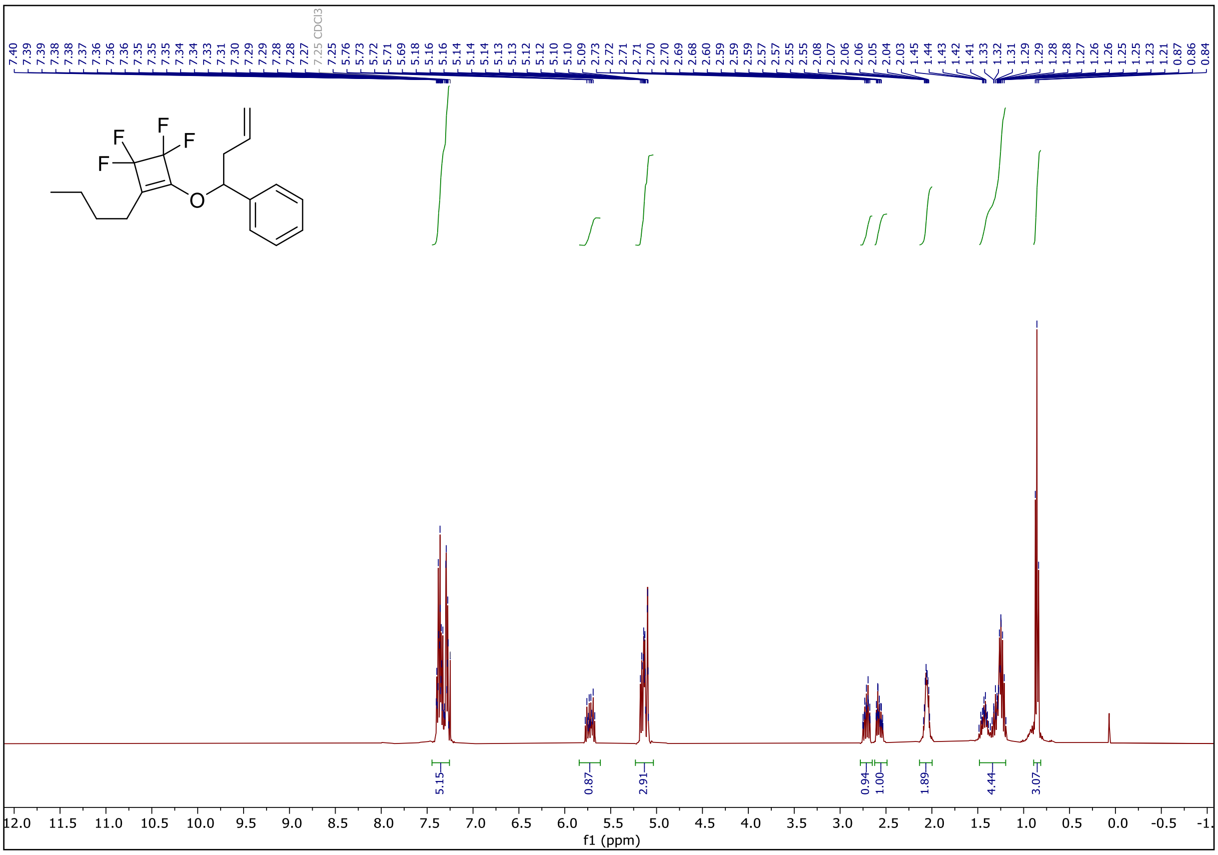

Figure S113: ^{1}H NMR spectrum of tetrafluorocyclobutene **15e** ($CDCl_3$, 400 MHz)

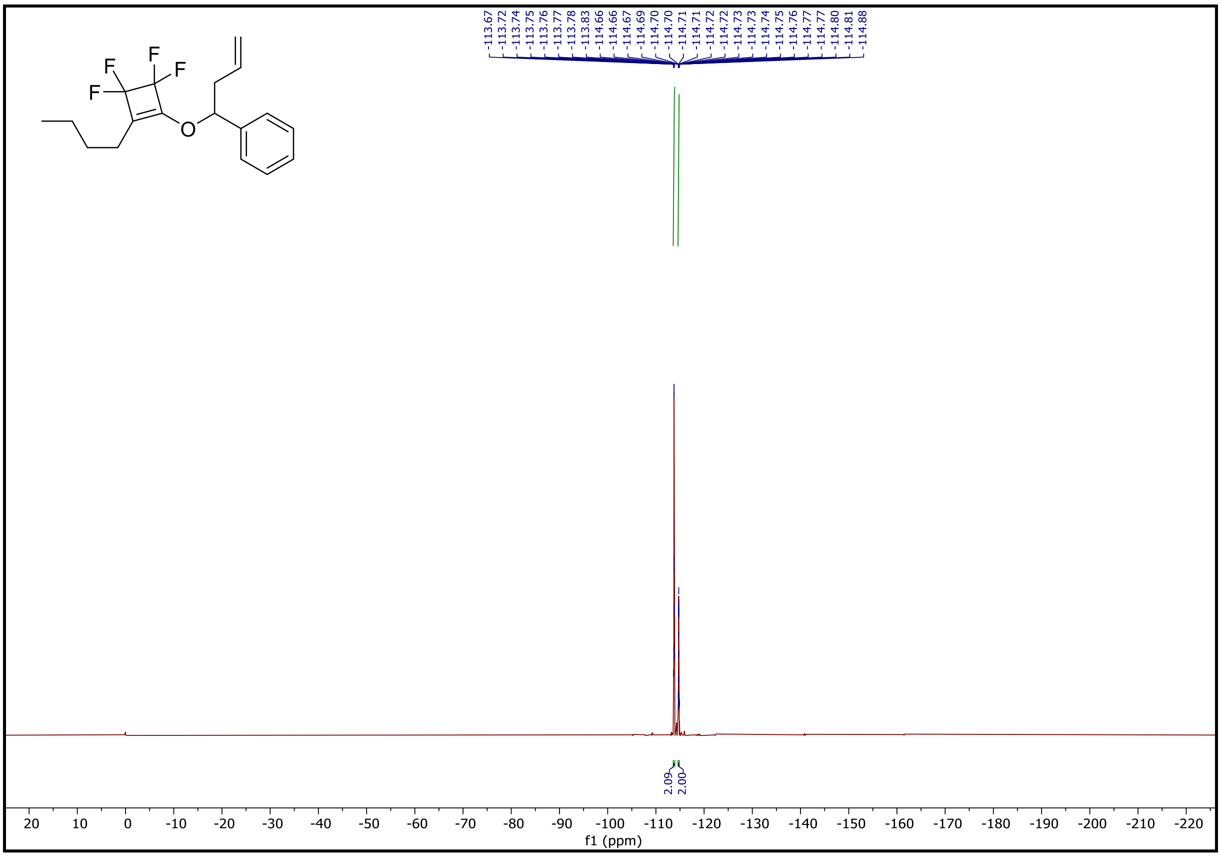

Figure S114: ^{19}F NMR spectrum of tetrafluorocyclobutene **15e** ($CDCl_3$, 376 MHz)

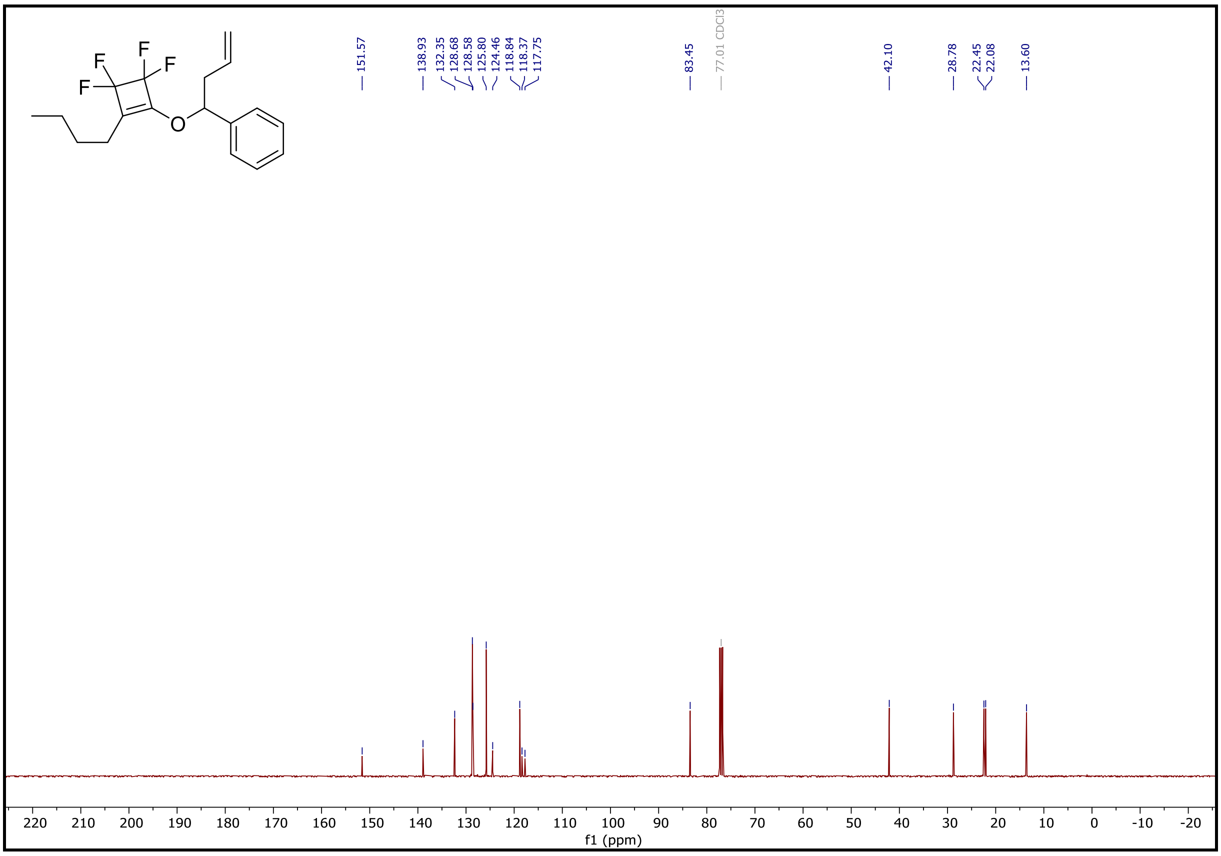

Figure S115: ^{13}C NMR spectrum of tetrafluorocyclobutene **15e** ($CDCl_3$,101 MHz,^{1}H and ^{19}F decoupling)

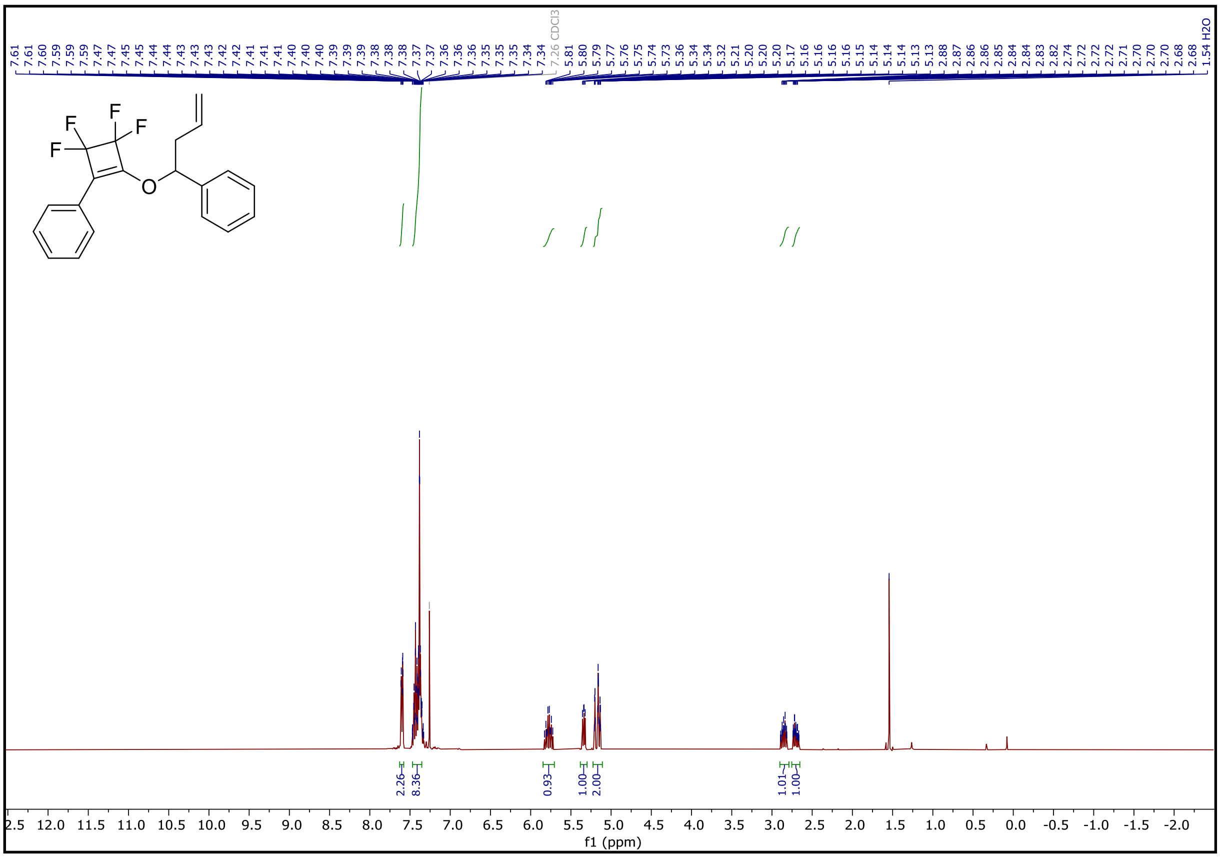

Figure S116: ^{1}H NMR spectrum of tetrafluorocyclobutene **15f** ($CDCl_3$, 400 MHz)

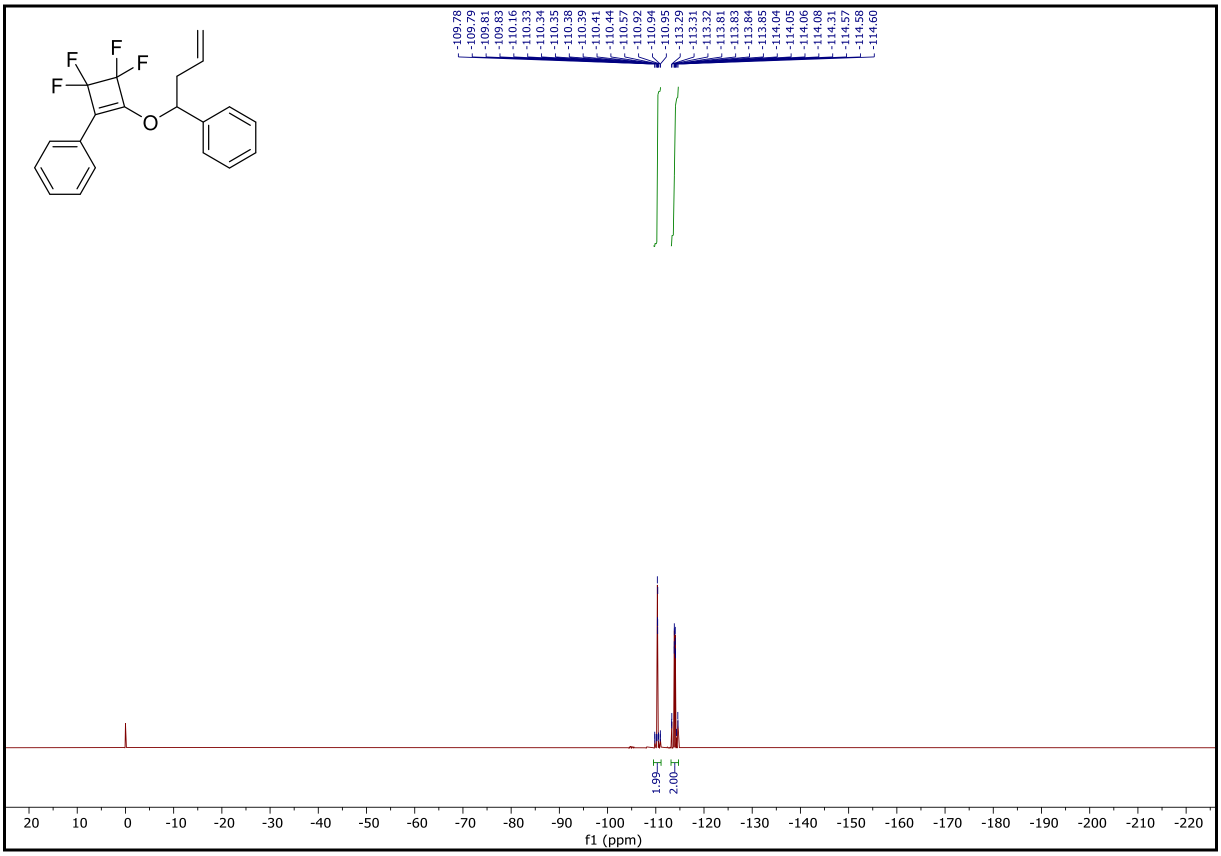

Figure S117: ^{19}F NMR spectrum of tetrafluorocyclobutene **15f** ($CDCl_3$, 376 MHz)

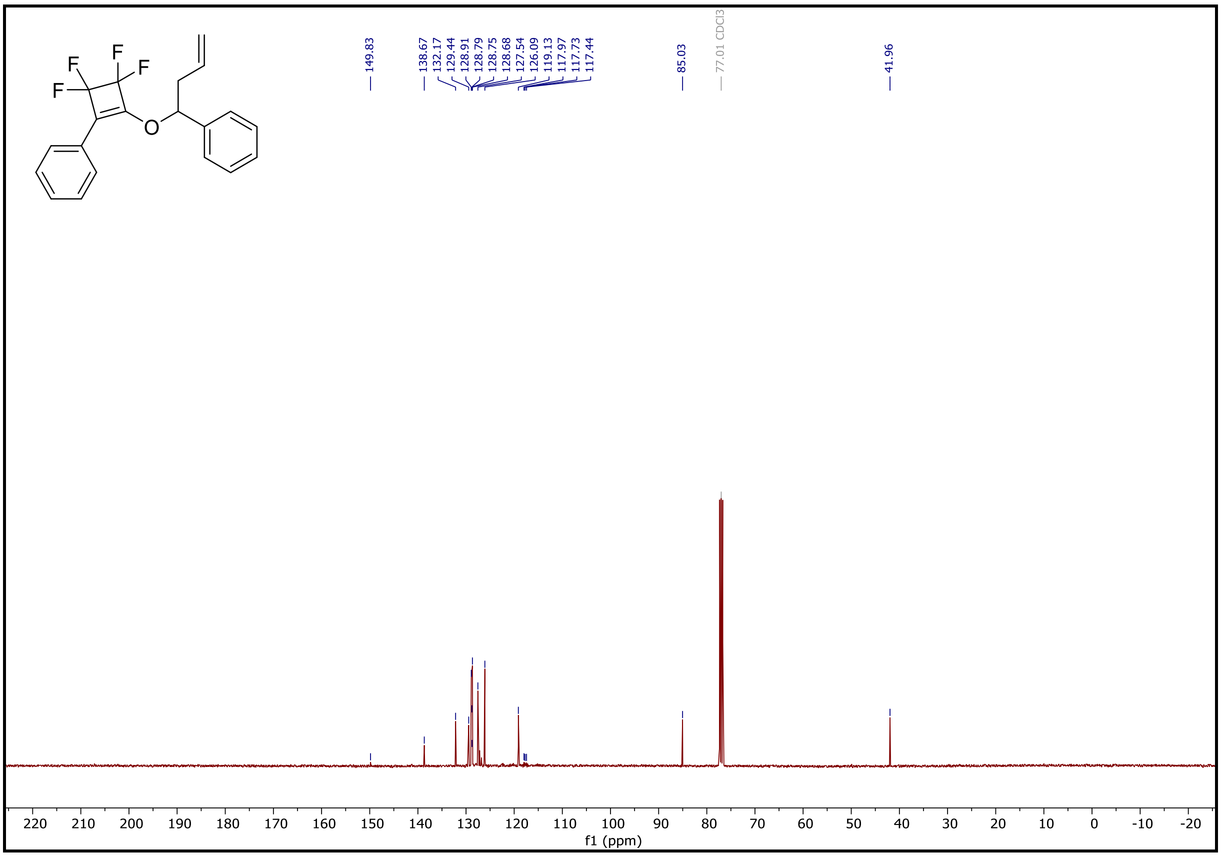

Figure S118: ^{13}C NMR spectrum of tetrafluorocyclobutene **15f** ($CDCl_3$,101 MHz,^{1}H and ^{19}F decoupling)

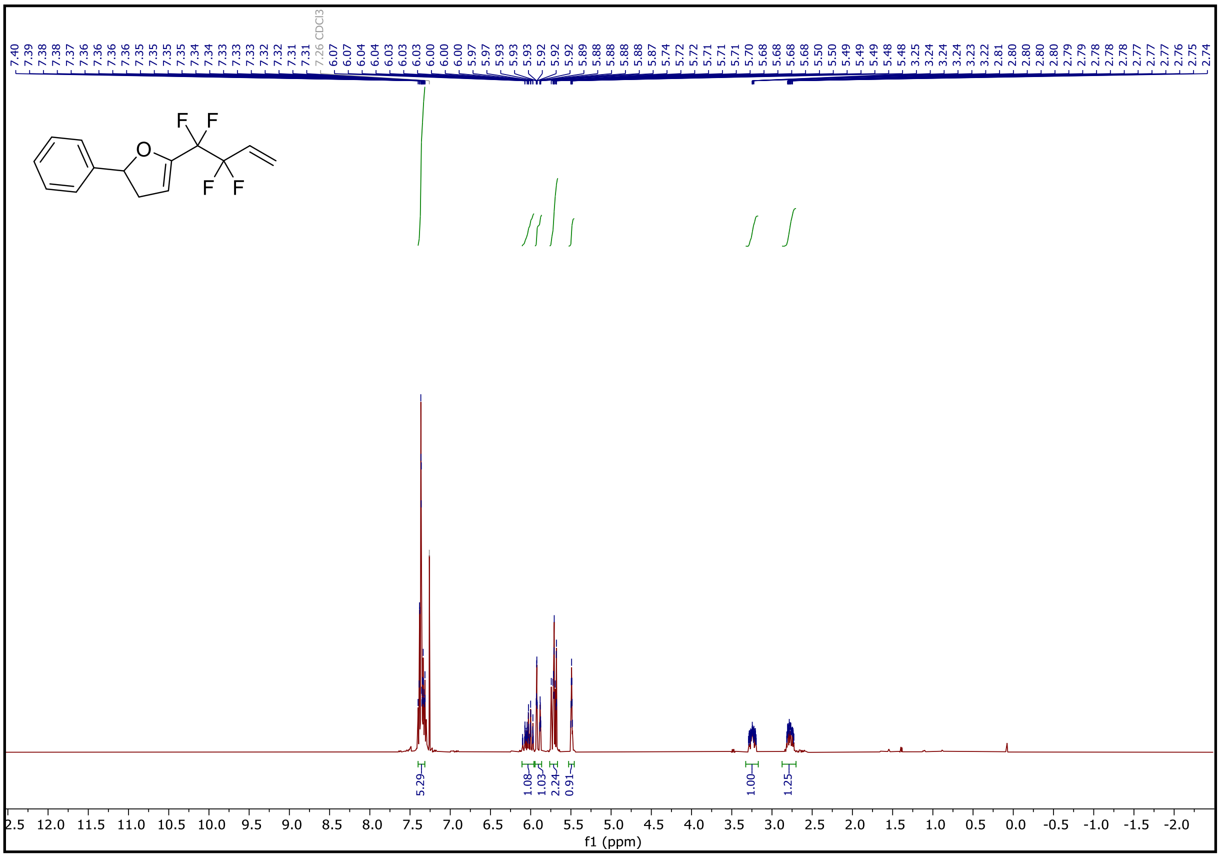

Figure S119: ^{1}H NMR spectrum of dihydrofuran **4b** ($CDCl_3$, 400 MHz)

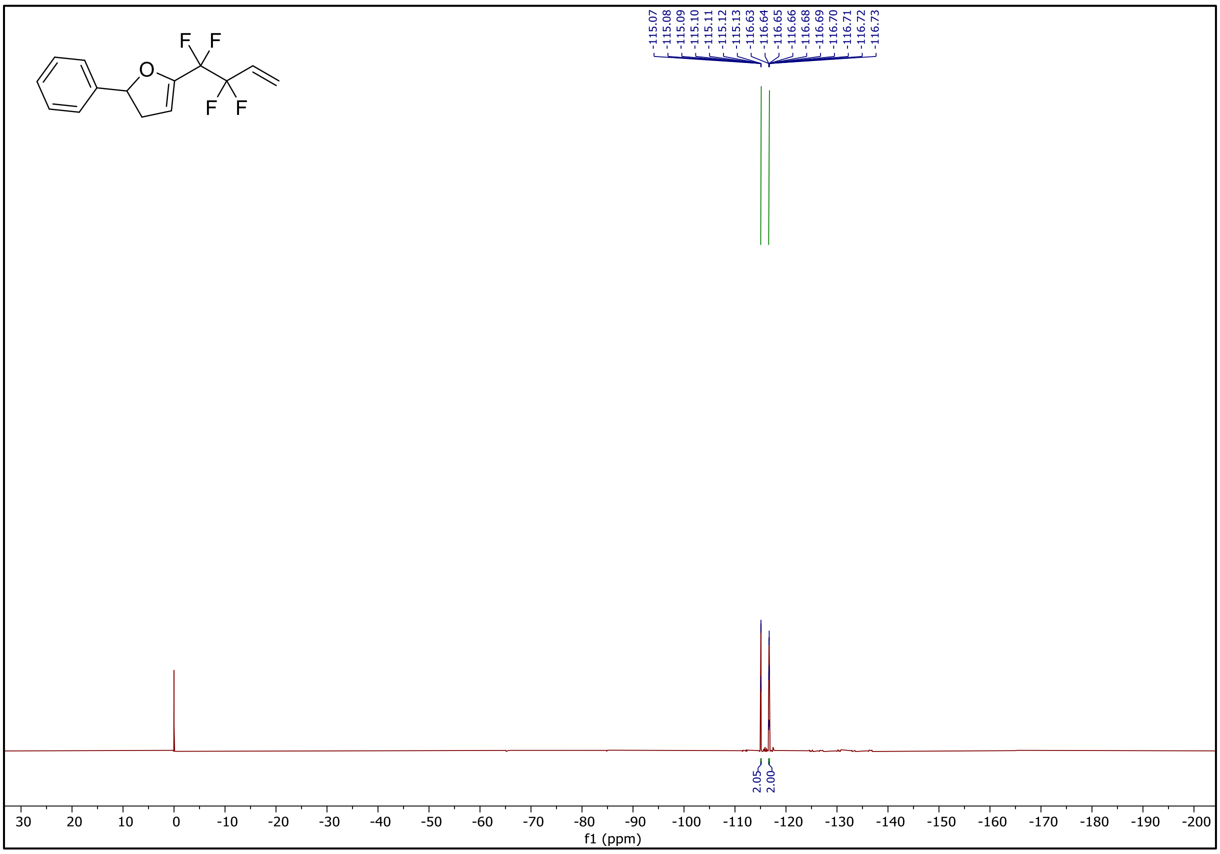

Figure S120: ^{19}F NMR spectrum of dihydrofuran **4b** ($CDCl_3$, 376 MHz)

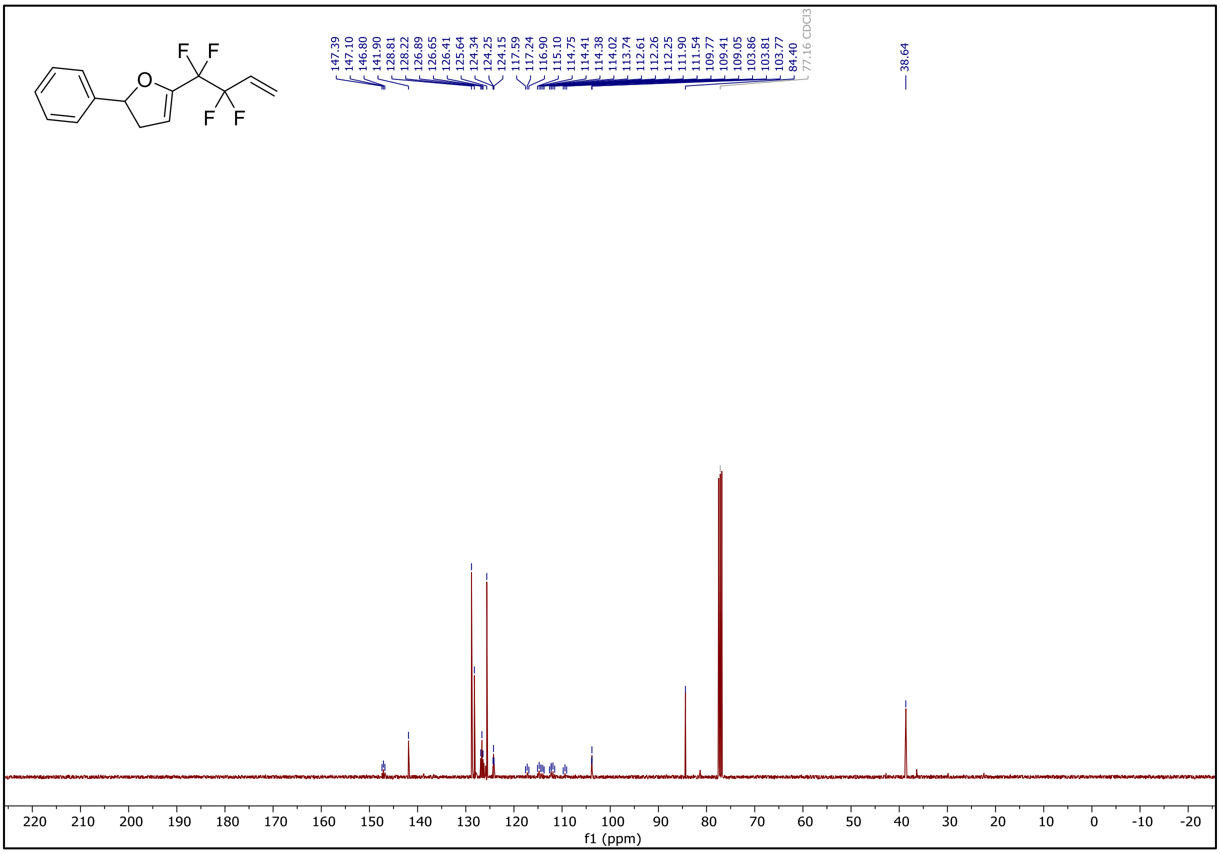

Figure S121: ^{13}C NMR spectrum of dihydrofuran **4b** ($CDCl_3$, 101 MHz)

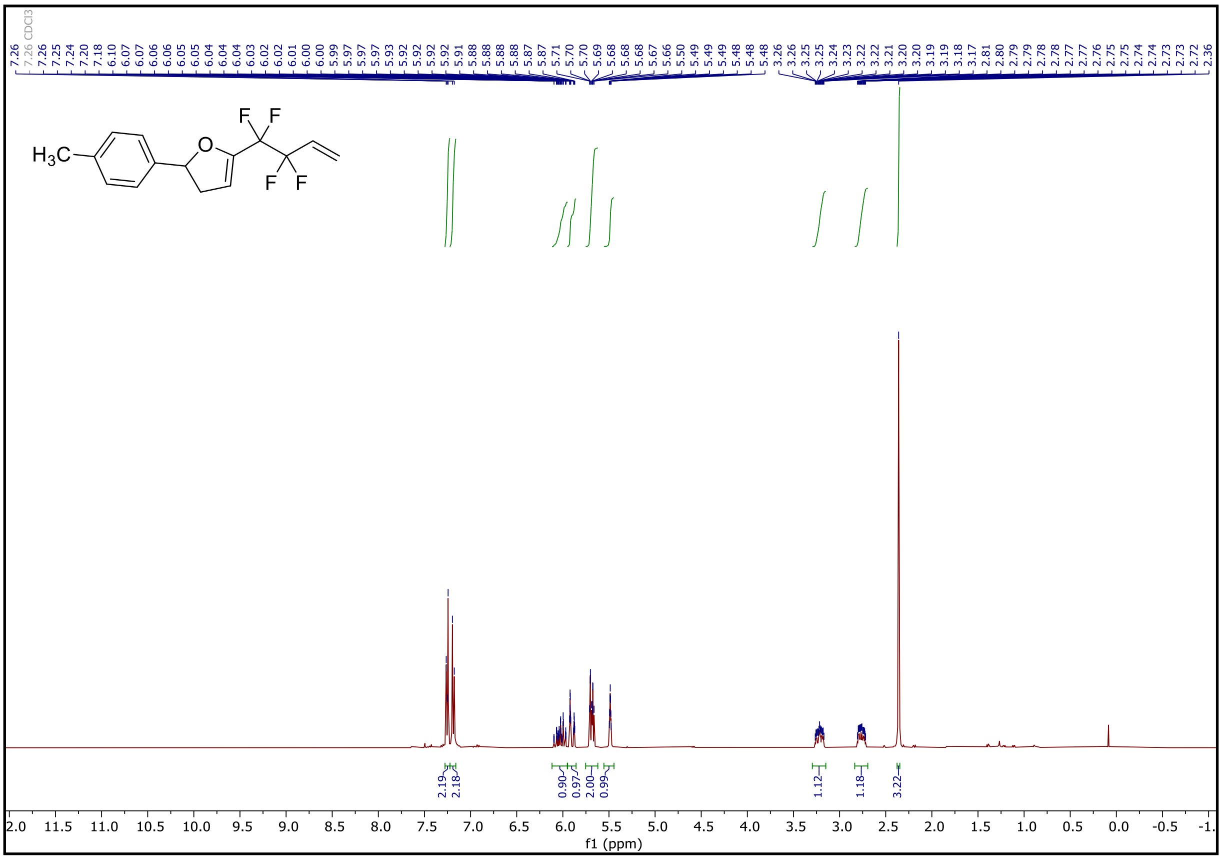

Figure S122: ^{1}H NMR spectrum of dihydrofuran **4c** ($CDCl_3$, 400 MHz)

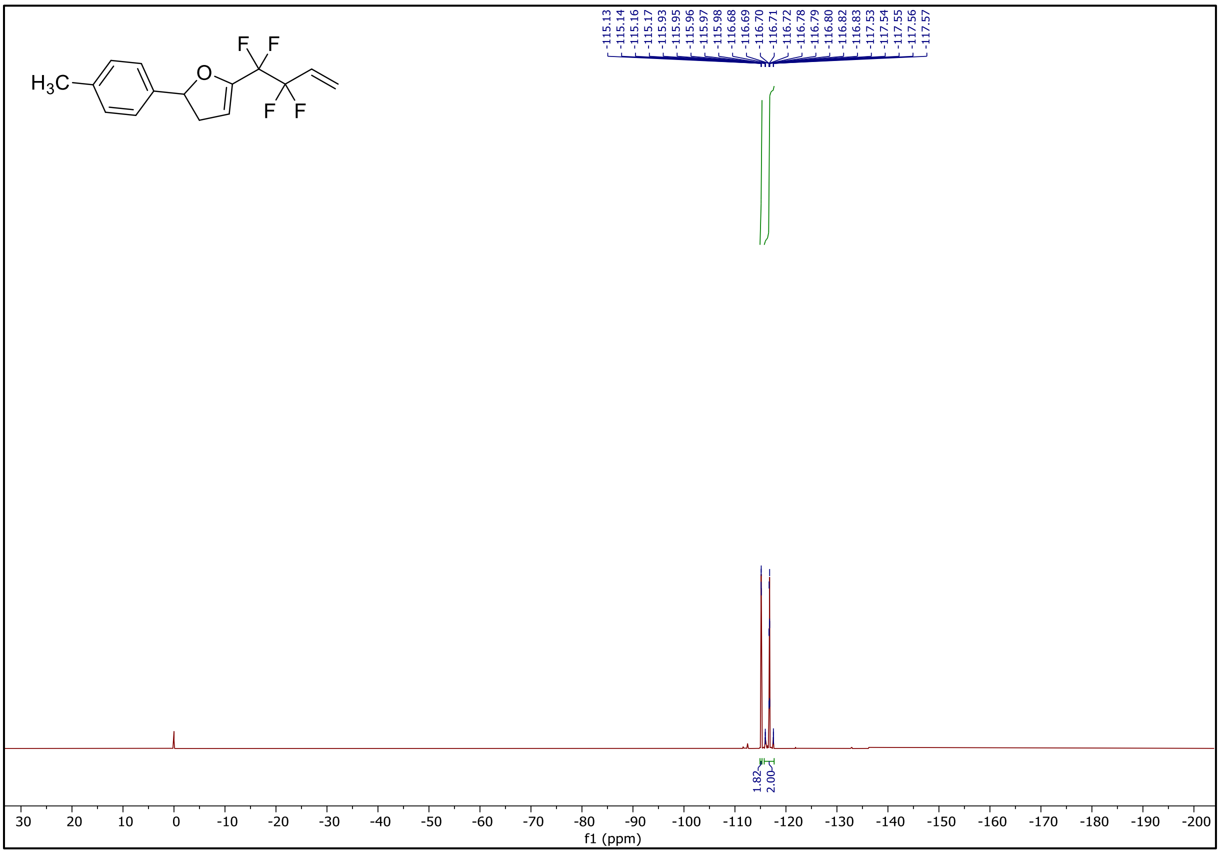

Figure S123: ^{19}F NMR spectrum of dihydrofuran **4c** ($CDCl_3$, 376 MHz)

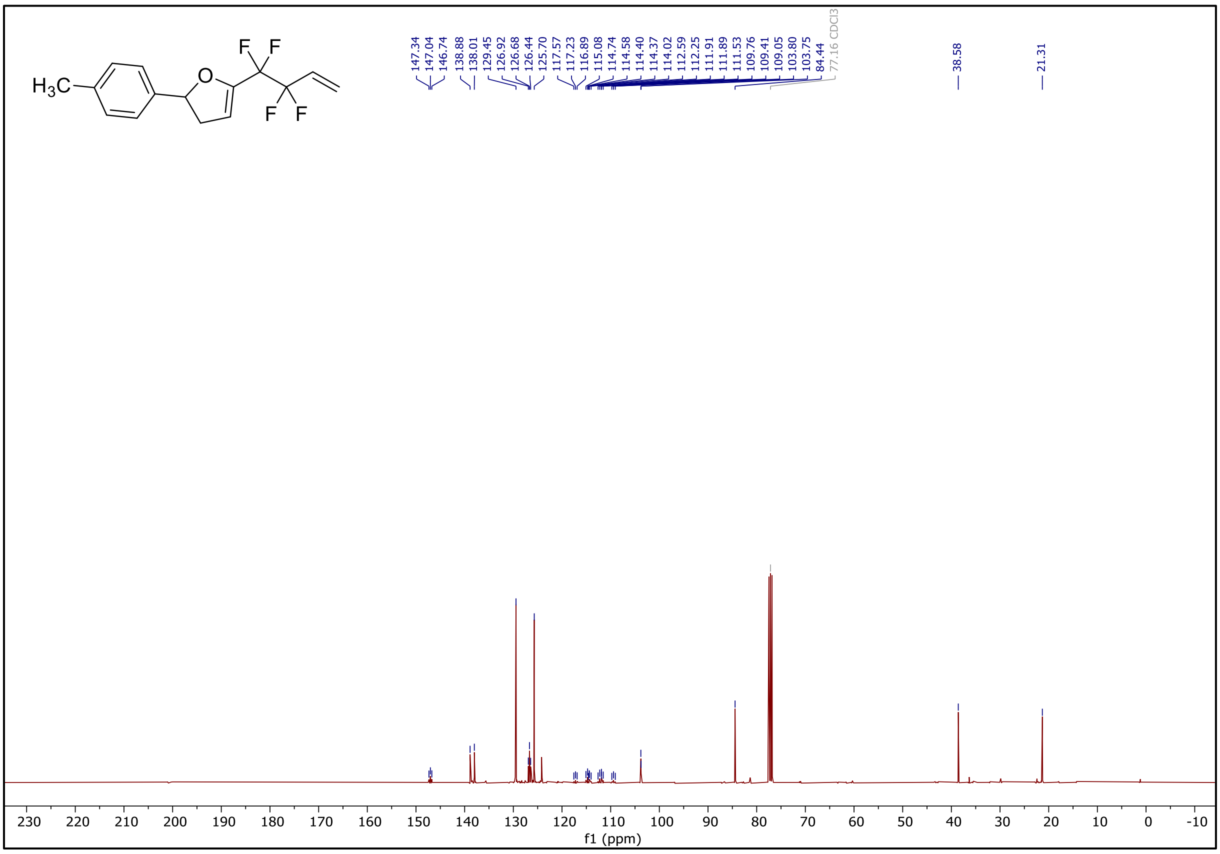

Figure S124: ^{13}C NMR spectrum of dihydrofuran **4c** ($CDCl_3$, 101 MHz)

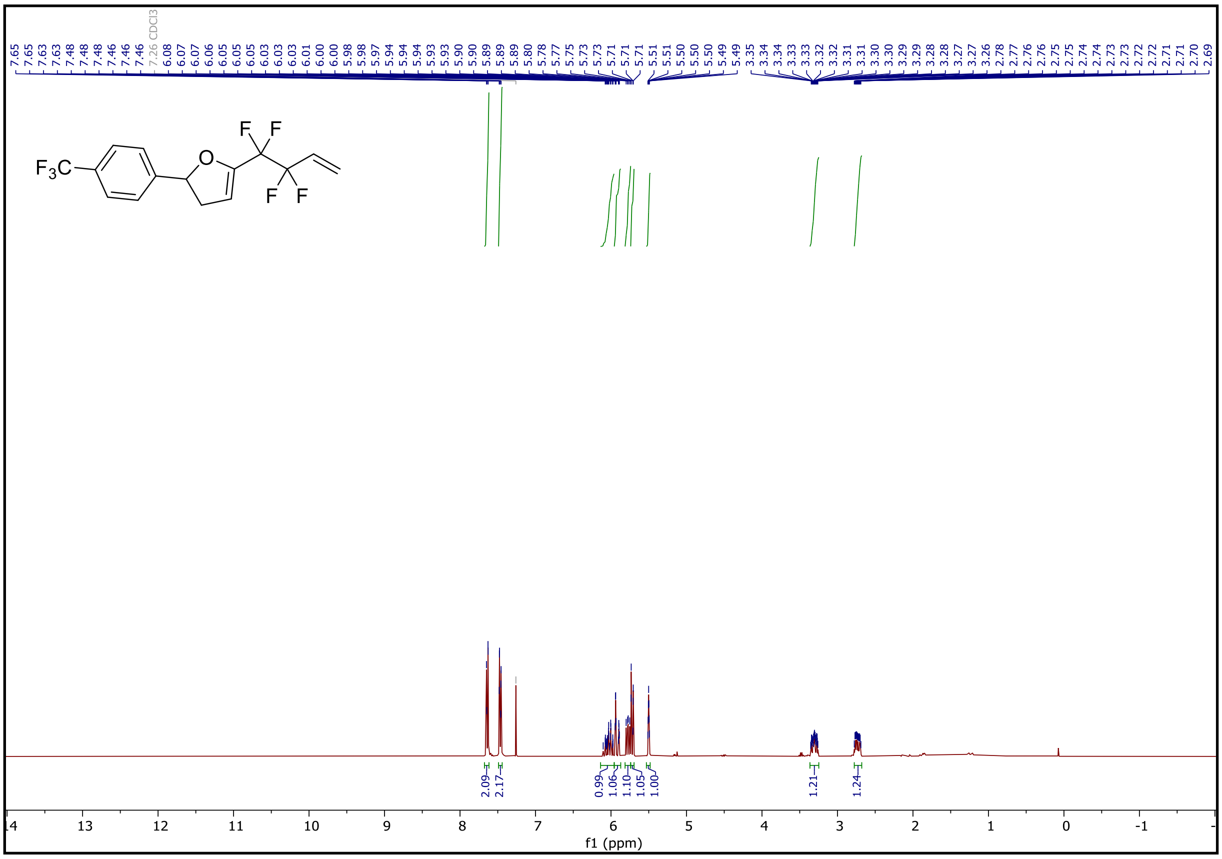

Figure S125: ^{1}H NMR spectrum of dihydrofuran **4d** ($CDCl_3$, 400 MHz)

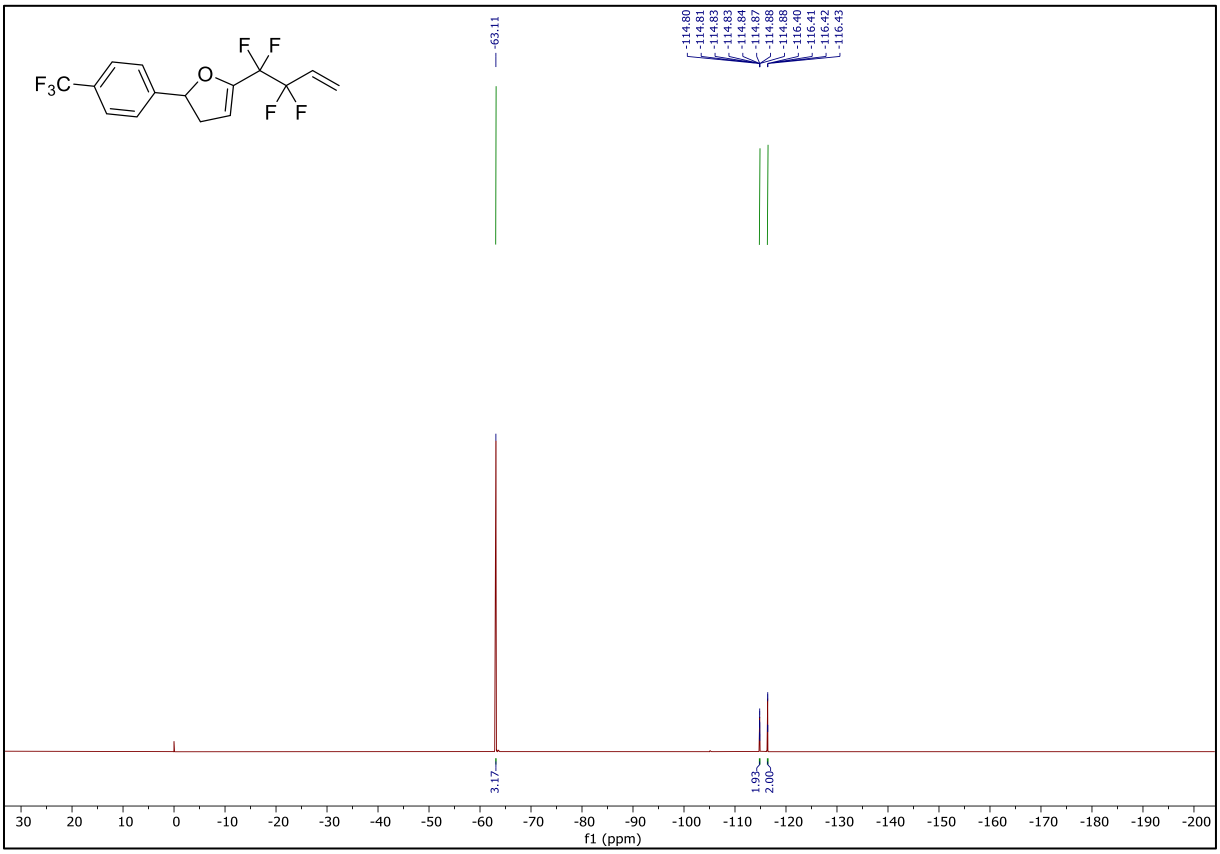

Figure S126: ^{19}F NMR spectrum of dihydrofuran **4d** ($CDCl_3$, 376 MHz)

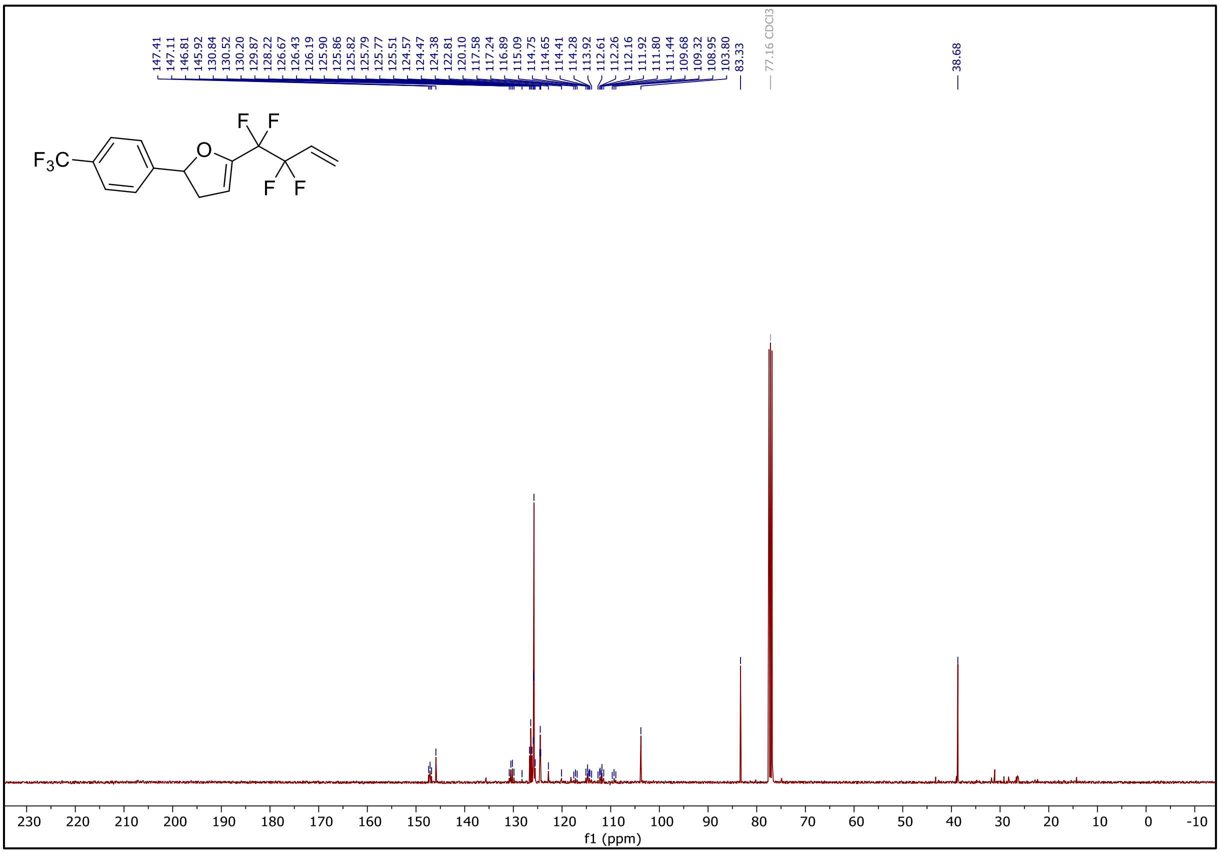

Figure S127: ^{13}C NMR spectrum of dihydrofuran **4d** ($CDCl_3$, 101 MHz)

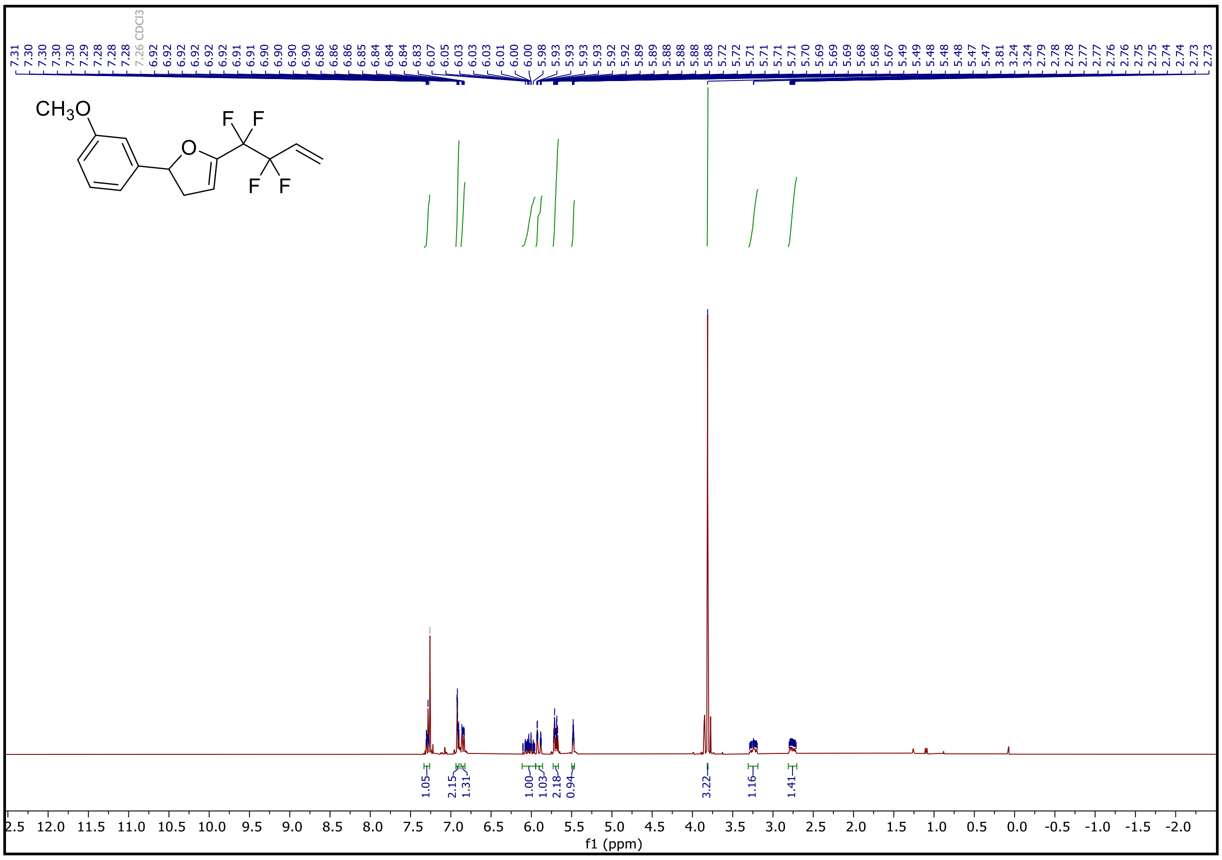

Figure S128: ^{1}H NMR spectrum of dihydrofuran **4e** ($CDCl_3$, 400 MHz)

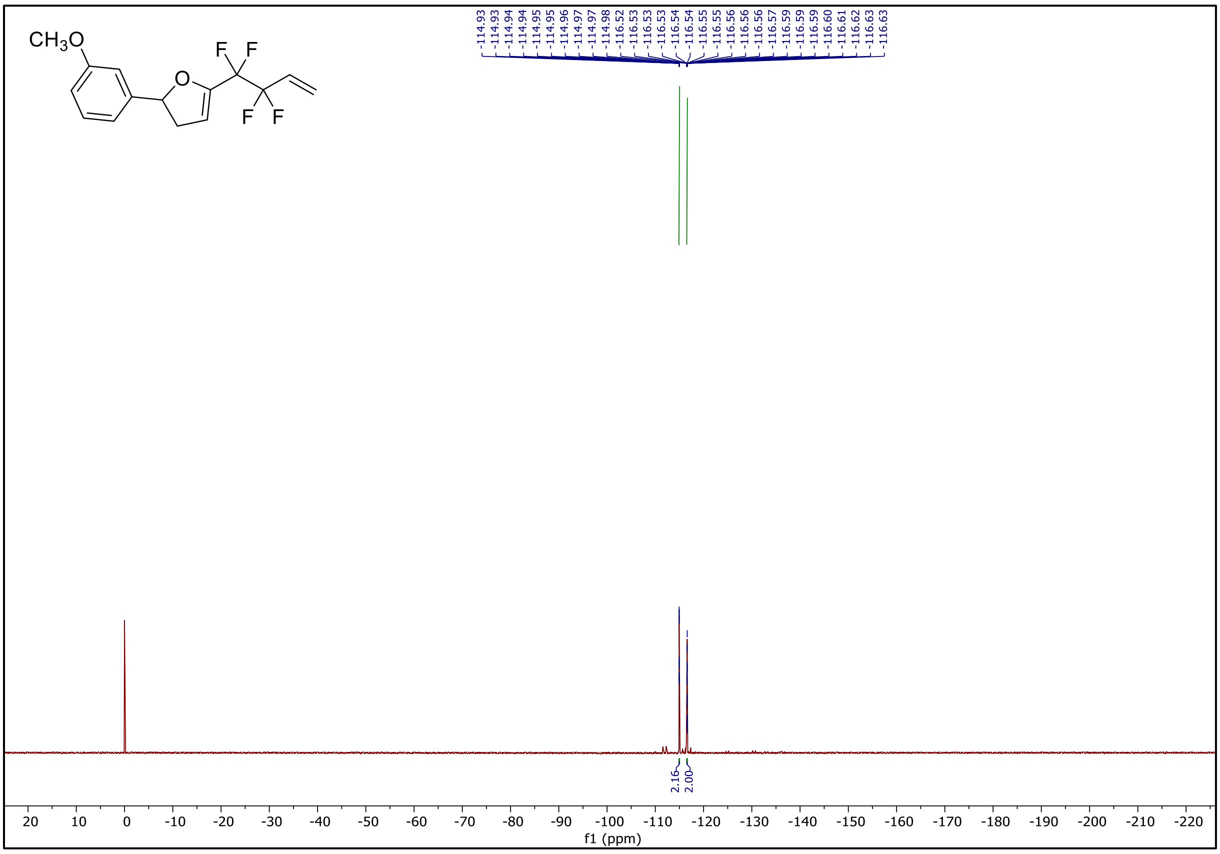

Figure S129: ^{19}F NMR spectrum of dihydrofuran **4e** ($CDCl_3$, 376 MHz)

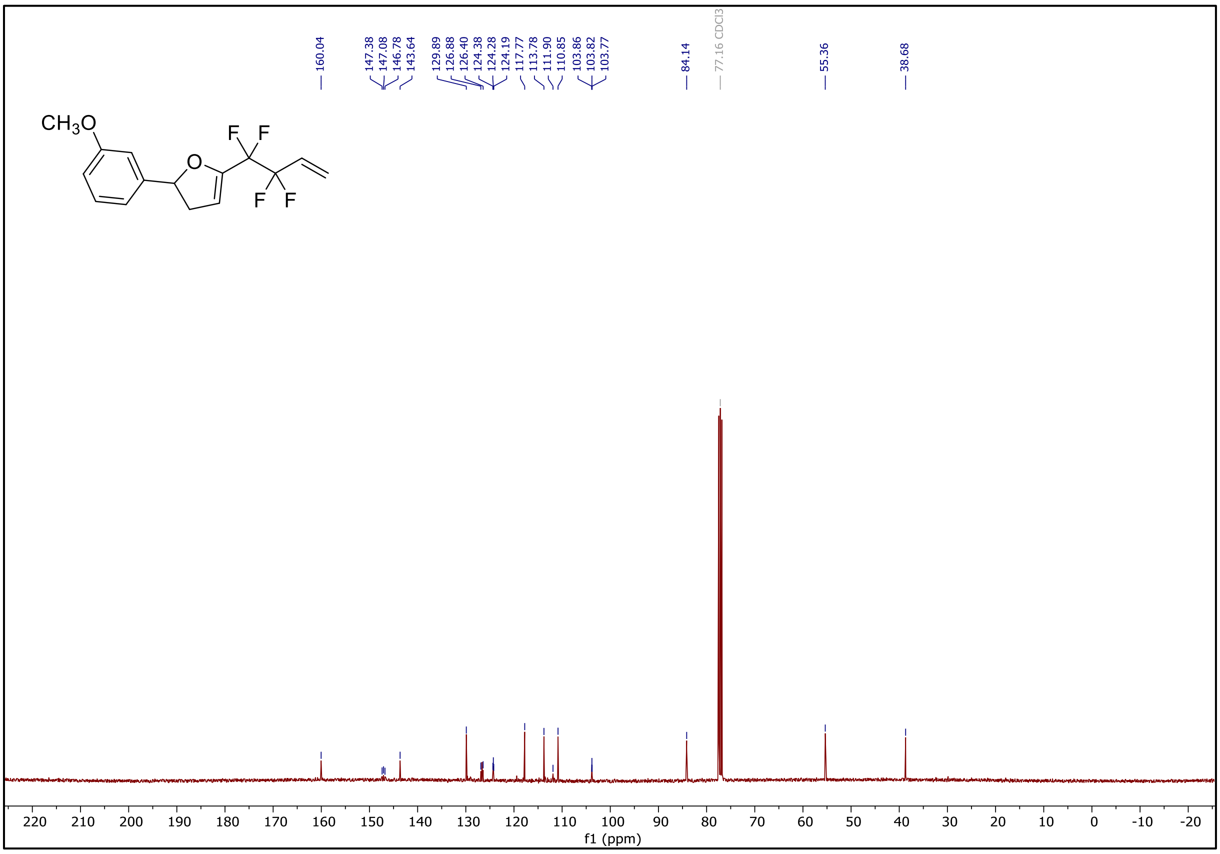

Figure S130: ^{13}C NMR spectrum of dihydrofuran **4e** ($CDCl_3$, 101 MHz)

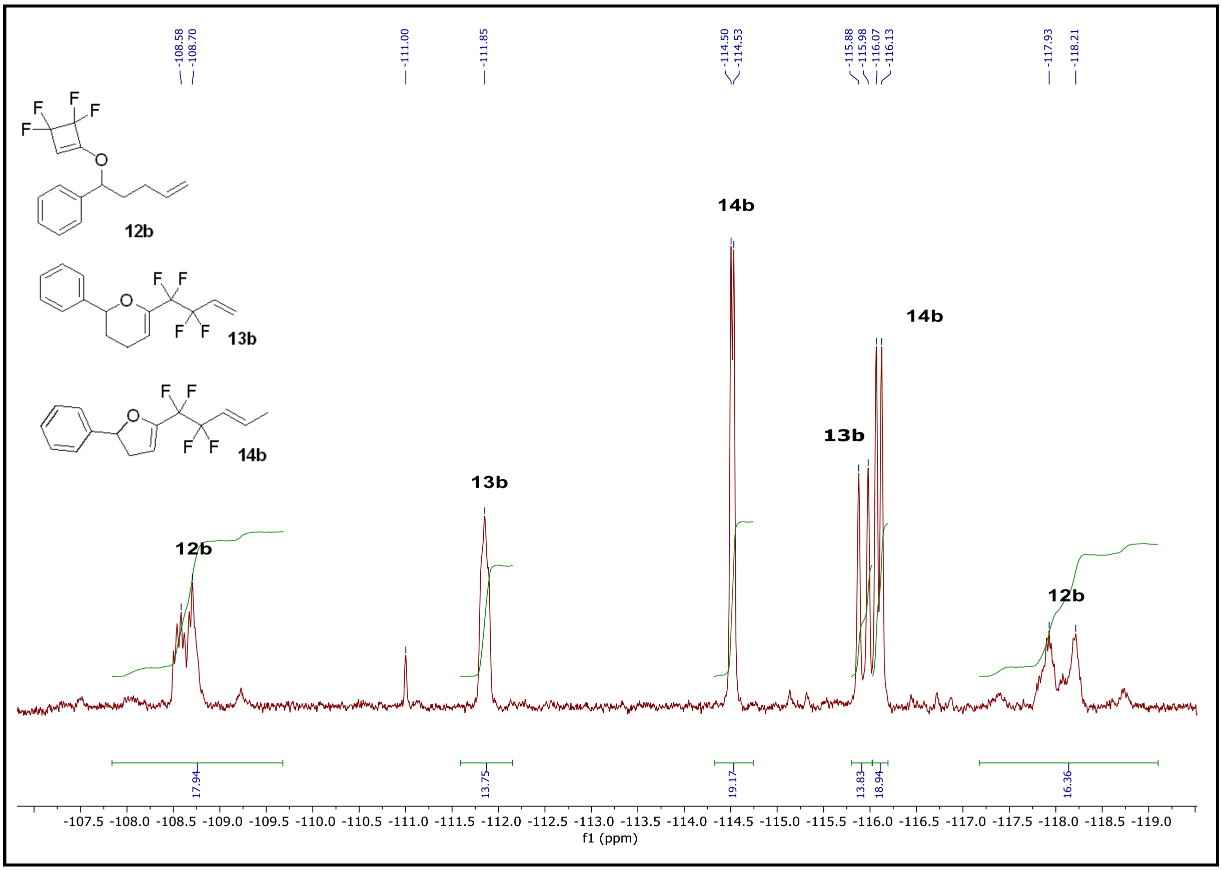

Figure S131: ^{19}F NMR spectrum of the mixture of dihydropyran **13b** and dihydrofuran **14b** ($CDCl_3$, 376 MHz)

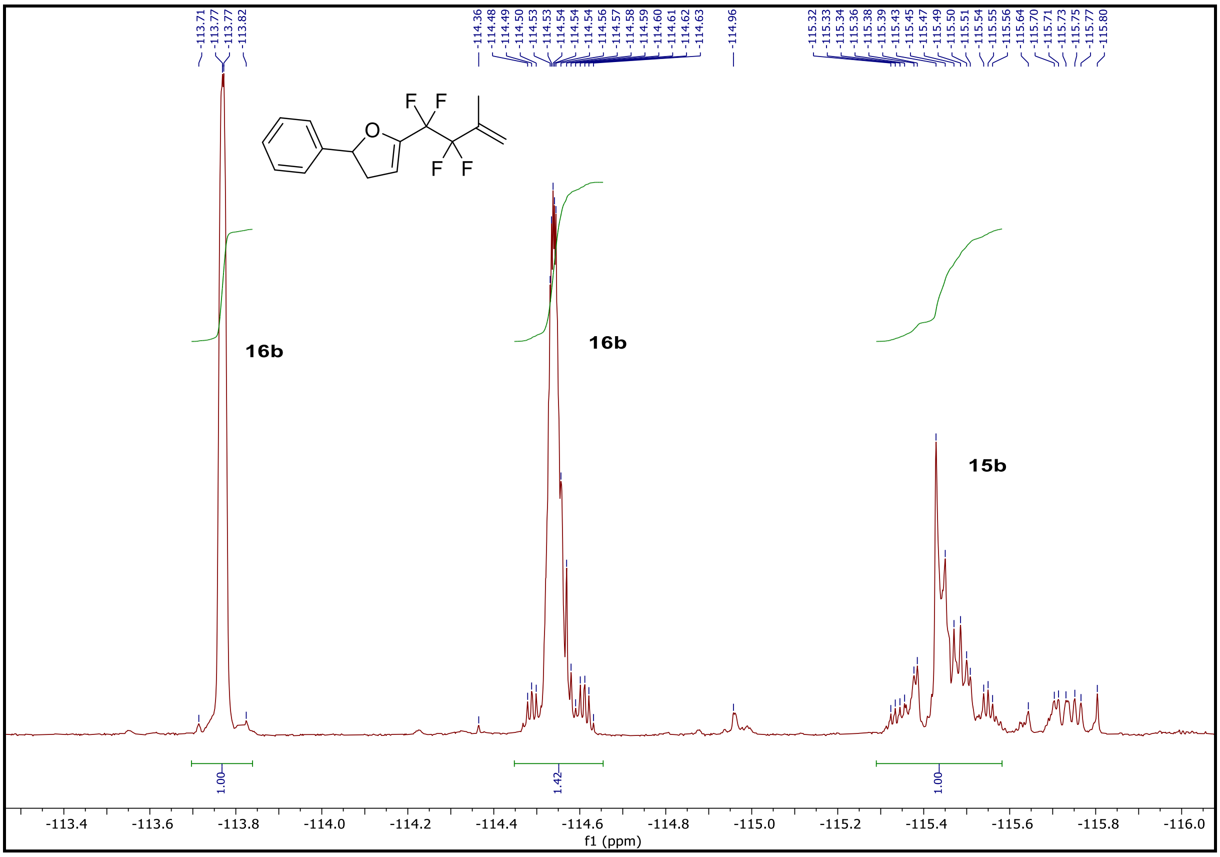

Figure S132: ^{19}F NMR spectrum of the mixture containing dihydrofuran **16b** ($CDCl_3$, 376 MHz)

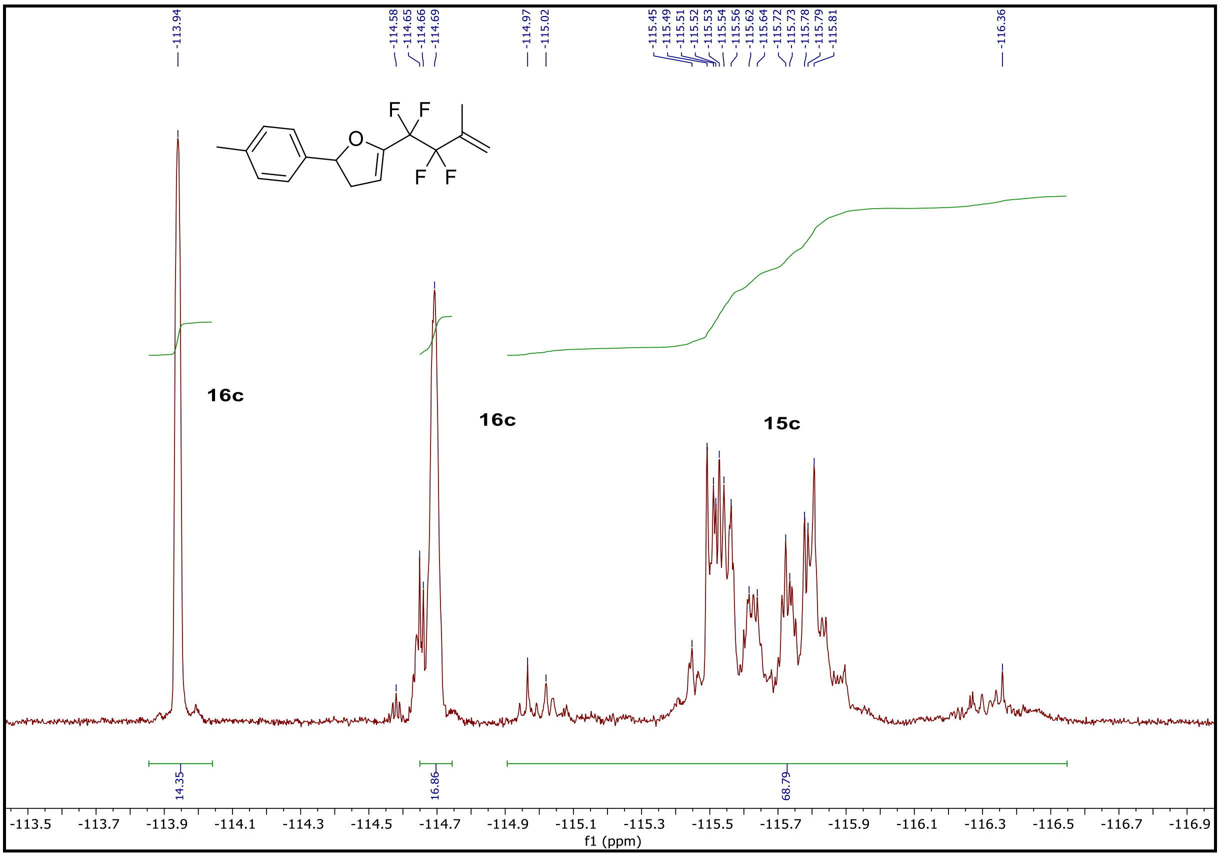

Figure S133: ^{19}F NMR spectrum of the mixture containing dihydrofuran **16c** ($CDCl_3$, 376 MHz)

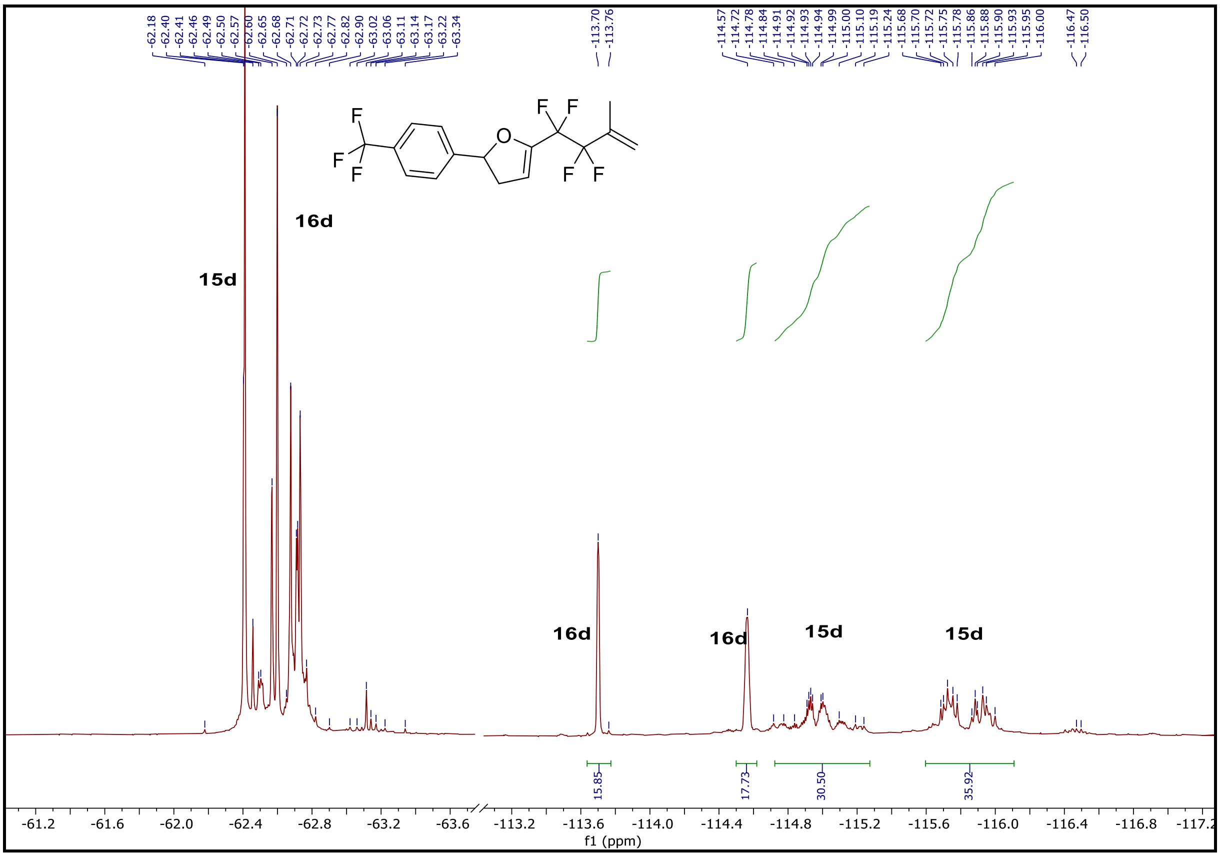

Figure S134: ^{19}F NMR spectrum of the mixture containing dihydrofuran **16d** ($CDCl_3$, 376 MHz)
